# Reversed reactivity of anilines with alkynes in the rhodium-catalysed C–H activation/carbonylation tandem

**DOI:** 10.1038/ncomms9591

**Published:** 2015-10-21

**Authors:** Siba P. Midya, Manoj K. Sahoo, Vinod G. Landge, P. R. Rajamohanan, Ekambaram Balaraman

**Affiliations:** 1 Catalysis Division, CSIR-National Chemical Laboratory (CSIR-NCL), Dr Homi Bhabha Road, Pune 411008, India; 2 Academy of Scientific and Innovative Research (AcSIR), New Delhi 110 025, India; 3 Central NMR Facility, CSIR-National Chemical Laboratory (CSIR-NCL), Dr Homi Bhabha Road, Pune 411008, India

## Abstract

Development of multicatalytic approach consisting of two or more mechanistically distinct catalytic steps using a single-site catalyst for rapid and straightforward access of structurally complex molecules under eco-benign conditions has significance in contemporary science. We have developed herein a rhodium-catalysed C–H activation strategy which uses an unprotected anilines and an electron-deficient alkynes to C–C bonded products as a potential intermediate in contrast to the archetypical C–N bonded products with high levels of regioselectivity. This is followed by carbonylation of C–H bond activated intermediate and subsequent annulation into quinolines has been described. This rhodium-catalysed auto-tandem reaction operates under mild, environmentally benign conditions using water as the solvent and CO surrogates as the carbonyl source with the concomitant generation of hydrogen gas. The strategy may facilitate the development of new synthetic protocols for the efficient and sustainable production of chemicals in an atom-economic way from simple, abundant starting materials.

## Abstract (web summary)

C-H activation reactions allow transformations on relatively unfunctionalised organic molecules. Here, the authors describe rhodium-catalysed C-H bond activation of unprotected anilines with alkynes leading to C-C bond formation (rather than the more usual C-N), and subsequent annulation to form quinolines.

## Graphical abstract

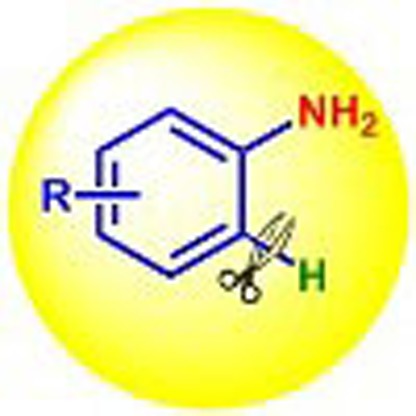

New chemical approaches that enable rapid and straightforward access to synthetically important molecules by sequential transformations, which enhances the synthetic efficiency is one of the prime focuses in chemical science. Many enzymes are often capable of selectively processing a single substrate through a multiple sequential reactions and are often an inspiration for the development of new synthetic transformations^1^. Recently, ‘auto-tandem catalysis', a strategy of association of two or more fundamentally different, that is, mechanistically distinct reactions promoted by a single-site catalyst, all of which occur under the same reaction conditions in a concurrent manner, is very attractive and highly desirable in organic synthesis^2^,^3^,^4^. In such tandem processes, potentially difficult work-up and product associated with the isolation and purification of the intermediates in multistep sequences can be avoided and the generation of chemical waste is also minimized. Development of such auto-tandem catalytic processes and a practically viable catalytic system is very challenging and sporadically mentioned due to the difficulty in the optimization of the separated processes independently^5^.

More recent, transition-metal-catalysed C–H bond activation approach has provided a direct and an atom-economical synthetic strategy to achieve structurally complex molecules from simple, pre-functionalised substrates and this has implication in developing more efficient synthetic methodologies^6^,^7^,^8^,^9^,^10^,^11^. The pre-installed directing group assisted *ortho*-C–H activation has been explored by several research groups. In recent times, a rare examples of challenging *meta*-C–H activation using the template strategy is also reported^12^,^13^. However, transition-metal-catalysed direct oxidative coupling between simple arenes and activated alkenes via the non-directed C–H bond activation strategy (electrophilic metallation) is of great interest and reported by research groups of Fujiwara^14^,^15^, Glorious^16^,^17^, Yu^18^,^19^ and others^20^,^21^. A gold-catalysed ethynylation of ‘deactivated' arenes with electron-deficient alkynes is also documented^22^. Despite using pre-functionalised substrates for the C–H bond activation event, a regio- and stereoselectivity and use of excess arenes for improved reactivity towards transition metals are of potential concern. Hence, selective C–H functionalization of arenes through the non-directed C–H activation strategy is highly challenging and synthetically demanding.

On the other hand, anilines serve as versatile precursors for the synthesis of *N*-heterocyclic scaffolds. In general, an aniline undergoes the Micheal-type 1,4-conjugate addition reaction with an electron-deficient alkyne and leads to usual carbon–nitrogen bonded product^23^. Transition-metal-catalysed hydroamination of alkynes (a formal addition of a N–H bond across a carbon–carbon multiple bond) for the synthesis of organo-nitrogen molecules is well documented (Fig. 1a)^24^,^25^. However, transition-metal-catalysed C–H bond activation of unprotected or ‘directing group-free' amines is extremely rare^26^,^27^,^28^,^29^,^30^,^31^. Typically it proceeds via the formation of a five- or a six-membered metallacyclic intermediate and notably, in all such reports, either benzylic amines or *ortho*-aryl/vinyl anilines were used as the effective directing groups^26^,^27^,^28^,^29^,^30^. Recently, transition-metal-catalysed oxidative C–H activation/annulation of anilines with alkynes to indole derivatives using molecular oxygen as an oxidant is also reported (Fig. 1b)^32^,^33^. The reaction proceeded through either *in situ* formation of NH(OAc) or the Michael-type intermediate. However, to the best of our knowledge, there is no example of non-directed catalytic C–H bond activation of unprotected primary anilines^34^,^35^.

Here we report a rhodium-catalysed C–H bond activation of unprotected (electron-rich primary) anilines with electron-deficient alkynes leading to formation of carbon–carbon bonded products as a potential intermediate in contrast to the archetypical carbon–nitrogen bonded products with high regio control. This is followed by carbonylation of C–H bond activated intermediate using CO surrogates and subsequent annulation reaction into the synthetically versatile 3-substituted quinolines is described (Fig. 1c). This rhodium-catalysed auto-tandem reaction operates under mild, environmentally benign conditions using water as a solvent and paraformaldehyde as a carbonyl source with the concomitant generation of hydrogen gas.

## Results

### Rhodium-catalysed C-H bond activation of anilines

**Figure 2:**
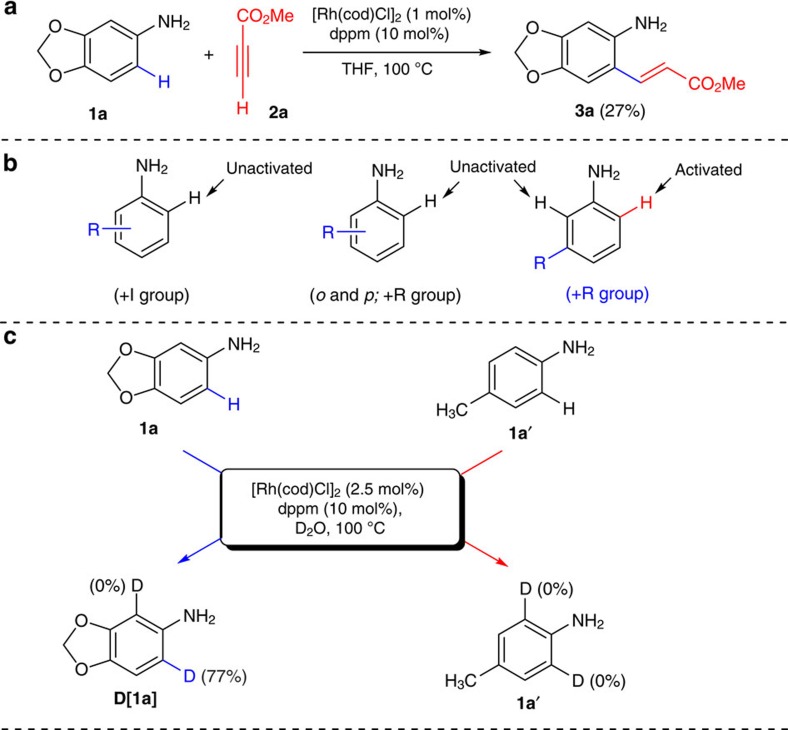
Rhodium-catalysed C–H bond activation of unprotected anilines. (**a**) Rhodium-catalysed C–H bond activation of anilines with alkynes leading to the formation of carbon–carbon bonded product. (**b**) Effect of various substituents in the non-directed C–H bond activation of anilines. (**c**) Rhodium-catalysed regioselective *ortho*-deuteration of anilines.

3,4-(Methylenedioxy)aniline (**1a**) and methyl propiolate (**2a**) were selected as a benchmark substrates for the non-directed C–H bond activation strategy. Treatment of 3,4-(methylenedioxy)aniline **1a** with methyl propiolate **2a** in the presence of catalytic amount of rhodium catalyst resulted in 27% yield of C–H bond activated product, ethyl-3-(6-aminobenzo[*d*][1,3]dioxol-5-yl)acrylate (**3a**) with the recovery of unreacted **1a** in 49% (Fig. 2a). To gain more insight into this unobserved reactivity of anilines with alkynes, H/D exchange experiment was performed (Fig. 2c). When 3,4-(methylenedioxy)aniline (**1a**) and *p*-toluidine (**1a′**) were separately treated with D_2_O in the presence of Rh(I)/dppm (1,1-bis(diphenylphosphino)methane) catalytic system, 77% of **1a** was selectively deuterated to give **[D]1a** with high regio control (Supplementary Figs 1 and 2) and whereas no H/D exchange was observed for **1a′**. These results imply that the carbon–carbon bond formation might begin with the *ortho*-C–H activation of **1a** and can be proceeded via the electrophilic metalation (that is, non-directed strategy)^16^,^17^,^18^,^19^,^22^, and a resonance effect of substituents (+R group) is responsible for the regioselective C–H activation of anilines.

### C-H bond activation/carbonylation tandem

Recently, Gulías *et al*.^36^ reported a rhodium-catalysed [5+1] cycloaddition of *ortho*-vinylphenols with carbon monoxide to lead a coumarin derivatives. Inspired by this result, we were encouraged to explore the possibility of utilization of the carbon–carbon bonded product (*ortho*-vinylanilines) for further transformation. Thus, we planned to integrate rhodium-catalysed non-directed C–H activation strategy with the carbonylation reaction in an auto-tandem manner^37^.

### Development and scope

After an extensive evaluation of combination of metal–ligand complexes, carbonyl sources, solvents and temperature (Table 1; Supplementary Tables 1–6), we have optimized the finest reaction conditions for this auto-tandem catalysis. For example, the treatment of 3,4-(methylenedioxy)aniline (**1a**) with methyl propiolate (**2a**, 1.1 equiv.) in the presence of catalytic amount of [Rh(cod)Cl]_2_:dppm (1:10 mol%) in tetrahydrofuran at 100 °C under 3 atm of CO for 12 h cleanly produced **4a** in 49% yield with the recovery of the unreacted **1a** in 42% (Table 1, entry 1). The reaction operates with double C–H bond activation under mild conditions with the formation of water as the by-product. To our delight, the same reaction was conducted using water as a solvent and yielded **4a** in 46% (Table 1, entry 2) with the concomitant generation of hydrogen gas. The liberation of molecular hydrogen was qualitatively analysed by gas chromatography (Supplementary Fig. 96).

Interestingly, when aqueous formaldehyde was used as a carbon monoxide surrogate^38^,^39^,^40^ in our auto-tandem approach, it showed a slight improvement in the product yield (Table 1, entry 3). Remarkably, excellent yields (up to 87%) were obtained when paraformaldehyde was used as a carbonyl source in CH_3_CN (Table 1, entry 6). Notably, addition of trace amount of water accelerated the reaction^41^ and smoothly yielded the desired product (Table 1, entries 4–6). These results highlight the importance of water as a reaction medium, which may increase the solubility of paraformaldehyde and thus provide a slow release of CO gas available for the carbonylation reaction. Hence, water was used as an optimal solvent for this one-pot operation to afford a green synthetic protocol to the 3-substituted quinolines^42^ (1 mol% of [Rh] yielded **3a** in 93% and 2.5% mol% of [Rh] yielded **3a** in 95%; entries 7–8). However, a three-component reaction of an aniline, an alkyne and an (aromatic) aldehyde to yield 2-substituted quinoline derivatives is a matured transformation in organic synthesis via typical Micheal-type 1,4-conjugate addition^42^,^43^. A multicomponent reaction of electron-deficient alkynes with amines and formaldehyde leads to polysubstituted pyrimidine derivatives were reported by Jiang and his co-workers and such type of product was not detected in our system^44^. It is very important to note that, in our reaction, paraformaldehyde and/or formalin solution were used as a carbonyl source (without using poisonous CO gas) and the reaction operates in an auto-tandem manner. The ‘auto-tandem' catalysis consisting of a non-directed C–H bond activation of free anilines leads to C–C bonded intermediate, generation of carbon monoxide from CO surrogates, and carbonylation of C–H activated product followed by annulation reaction to yield 3-substituted quinolines with the generation of hydrogen gas. Indeed, there are no reports describing the direct syntheses of biologically important 3-substituted quinoline derivatives from simple, feedstock chemicals and usually accessed in a multistep synthetic procedures^45^.

**Figure 3:**
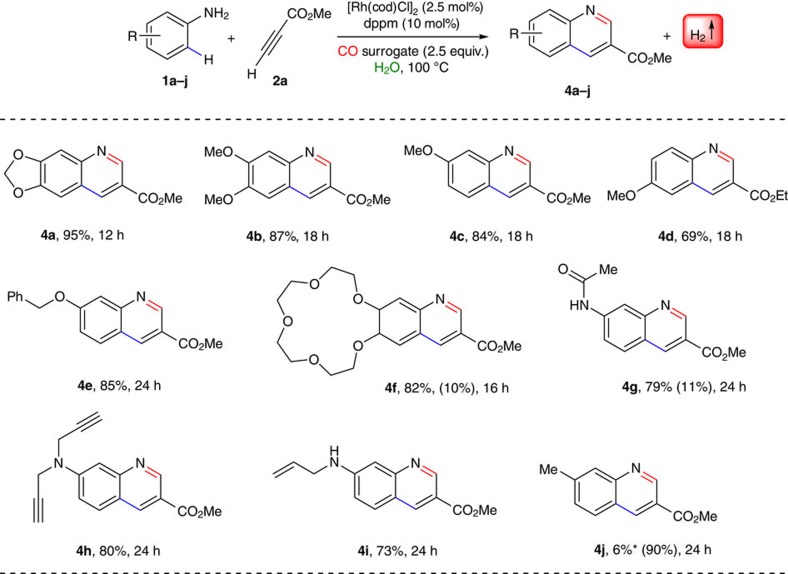
Scope of anilines. Reaction conditions: anilines **1a–j** (0.25 mmol), methyl propiolate **2a** (0.275 mmol), [Rh(cod)Cl]_2_ (2.5 mol%), dppm (10 mol%), (HCHO)_*n*_ (2.5 equiv.) and 250 μl of H_2_O were heated at 100 °C under closed viol for specified time and depicted yields are isolated yields (yields in parentheses are recovery of the starting material). *Yield based on ^1^H NMR of crude reaction mixture using toluene as an internal standard (using 1 mol% of the rhodium catalyst).

With the optimized reaction conditions in hand, we evaluated the substrate scope of the reaction with respect to the arene component and probed the generality of the auto-tandem process by generating a library of 3-substituted quinolines. As depicted in Fig. 3, variety of electron-rich anilines was found to be the good commodity for this auto-tandem reaction. This is attributed to the electronic nature of the substituents (+R effect), and may facilitate the electrophilic metallation step much easier (Fig. 2b). Thus, a tool box of electron-rich anilines (alkoxy, crown-ether type, amide, alkyl, *N*-allyl and *N*-propargyl) were reacted smoothly with **2a** via C–H bond activation and yielded the C–C coupled product as a potential intermediate (in contrast to the archetypical C–N bonded products) and followed by subsequent carbonylation using CO surrogates and annulation reaction to the 3-substituted quinolines in good to excellent yields (up to 95% isolated yield). The reaction is highly regio- and chemoselective and water and hydrogen gas is the only by-products. Remarkably, both allylic and propargylic groups were well tolerated under our reaction conditions. Alkyl substituted aniline (**4j**) suffered from suppressed reactivity and observed poorer yield (∼6% by ^1^H NMR). We presume this lower reactivity of aniline to be a consequence of decreased arene electron density: less *η*^2^ coordination and more Lewis basic coordination to the metal centre.

**Figure 4:**
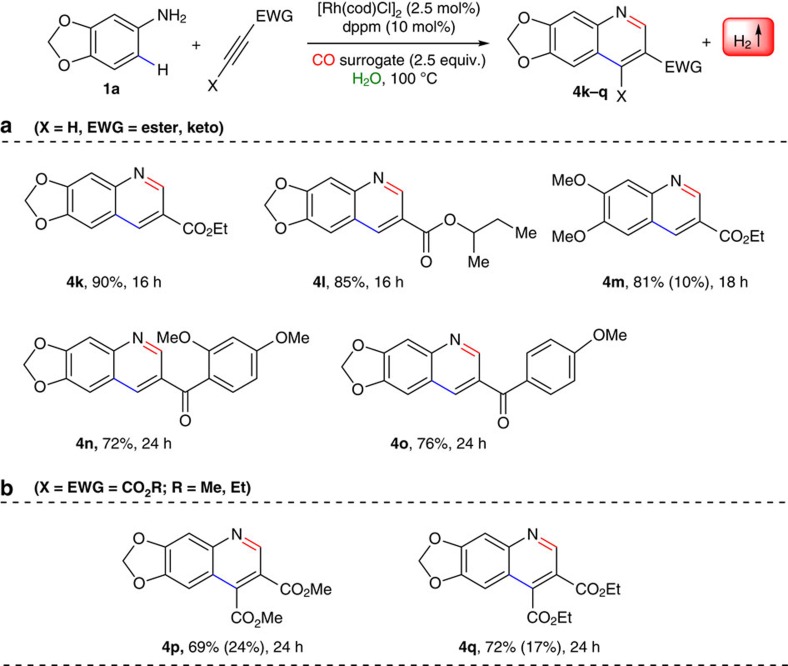
Scope of alkynes. Reaction conditions: 0.25 mmol of 3,4-(methylenedioxy)aniline (**1a**) or 3,4-domethoxyaniline (**1m**), alkynes (0.275 mmol), [Rh(cod)Cl]_2_ (2.5 mol%), dppm (10 mol%), (HCHO)_*n*_ (2.5 equiv.) and 250 μl of H_2_O were heated at 100 °C under closed viol for specified time and depicted yields are isolated yields (yields in parentheses are recovery of the starting material).

**Figure 5:**
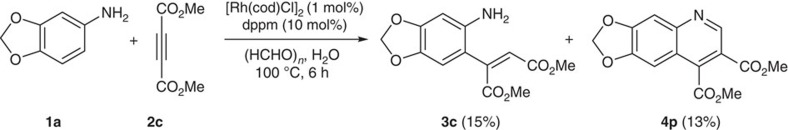
Evidence for the formation of C–C bonded intermediate. Reaction of **1a** with DMAD (dimethyl acetylenedicarboxylate) **2c**.

Subsequently, we have also investigated the substrate scope with regard to the alkynes (Fig. 4). Both carboxyl ester (entries **4k–4m**) and keto substituted (**4n–4o**) terminal alkynes gave expected quinoline derivatives in excellent yields (up to 90% isolated yields). To our delight, dialkyl dicarboxylates smoothly underwent intermolecular annulation with aniline (**1a**) and paraformaldehyde as a carbonyl source in water leading to the corresponding 3,4-disubstitutedquinoline derivatives (**4p–4q)** in good yields. Thus various electron-deficient alkynes including internal and terminal alkynylesters, linear and branched alkynylesters, and aryl ketoalkynes were shown good reactivity and yielded the desired 3- and 3,4-substituted quinolines in good to excellent yields with high regio- and chemioselectivity under mild, environmentally benign conditions with the liberation of molecular hydrogen. Importantly, in the reaction of 3,4-(methylenedioxy)aniline (**1a**) with dimethyl acetylenedicarboxylate under standard reaction conditions with a shortened reaction time (6 h) yielded a mixture of the intermediate **3c** (15%) and the desired product **4p** with the yield of 13% (Fig. 5). This result evidently proved that the auto-tandem approach proceeds through the C–C bonded intermediate.

**Figure 6:**
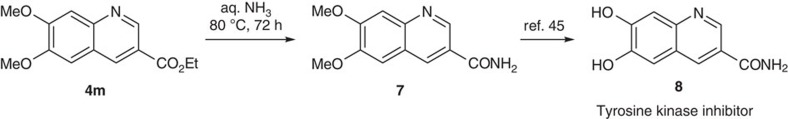
Synthesis of **8**, a tyrosine kinase inhibitor. Reaction conditions: 0.25 mmol of **4m** (synthesized by our auto-tandem strategy) and 4 ml of aq. NH_3_ were heated at 80 °C under closed viol for 72 h.

We have also designed a three-step synthetic protocol for 6,7-dihydroxyquinoline-3-carboxamide (**8**), a tyrosine kinase inhibitors (Fig. 6); which resulted in a 54% (overall) yield using our stragety^45^.

### Carbonylation of *ortho*-vinylanilines using CO surrogates

**Figure 7:**
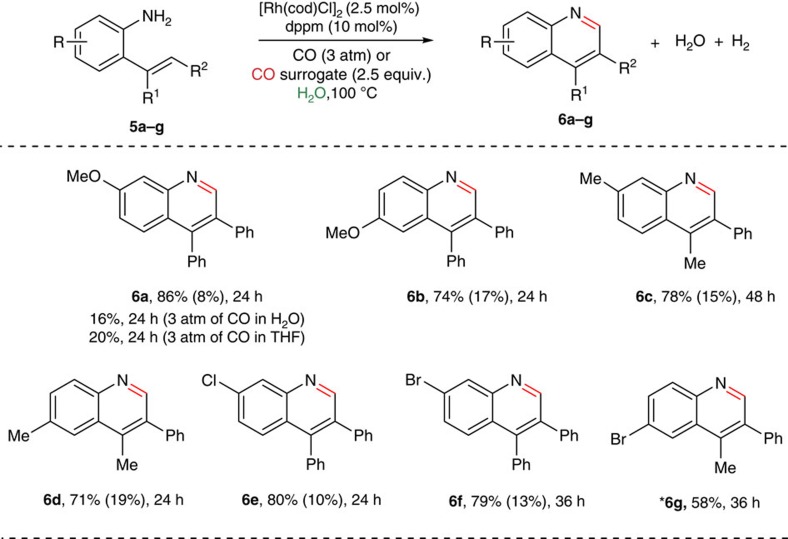
Rhodium-catalysed carbonannulation of *ortho*-vinylanilines using CO (surrogate) in water. Reaction conditions: *ortho*-vinylanilines **5a–g** (0.25 mmol), [Rh(cod)Cl]_2_ (2.5 mol%), dppm. (10 mol%), (HCHO)_*n*_ (3 equiv.) and 250 μl of H_2_O were heated at 100 °C under closed viol for specified time and the yields in parenthesis are recovery of unreacted starting material. *Yields based on ^1^H NMR of the reaction mixture (using 1 mol% of the rhodium catalyst).

In addition to the one-pot operation, we speculated on the possibility where conditions could be developed to convert various *ortho*-vinylanilines to the corresponding quinoline derivatives via sequential carbonylation and intramolecular imination reactions using CO surrogates as the carbonyl source (Fig. 7). However, performing carbonylation reactions both in industry and academia without the use of carbon monoxide (gasesous form, highly toxic, flammable and need of special high pressure equipment) is highly desired^39^,^40^,^46^. As described previously, we were hopeful with the Rh(I)/dppm catalytic system and CO surrogates in water as a carbonyl source to accomplish the carbonylation reaction of *ortho*-vinylanilines. This is followed by the trapping of aldehydic intermediate by the amine counterpart may lead to 3,4-disubstituted quinolines in one-pot operation. Alper *et al*.^47^ reported Pd-catalysed oxidative cyclocarbonylation of *ortho*-vinylanilines to 2(1*H*)-quinolinones. Indeed, as far as we know, carbonylation of *ortho*-vinylanilines using CO surrogates has never been reported. Thus, treatment of (*E*)-2-(1,2-diphenylvinyl)-5-methoxyaniline, **5a** (0.25 mmol) with paraformaldehyde (0.75 mmol) at 100 °C for 24 h with a catalytic amount of [Rh(cod)Cl]_2_ (2.5 mol%) and dppm (10 mol%), using water as the solvent, resulted 7-methoxy-3,4-diphenylquinoline in 82% isolated yield (**6a**) with the concomitant generation of hydrogen gas. Notably, the same reaction under 3 atm pressure of carbon monoxide yielded **6a** in 16% (water as the only by-product). The reaction is general and a variety of *ortho*-vinylanilines were compatible with this transformation. Thus, electron-donating groups proceeded smoothly to provide the corresponding carboannulated products **6a–6b** (up to 86% isolated yield), wherein electron-withdrawing groups (*p*-NO_2_ and *m*-CF_3_) were found to be ineffective under standard conditions. This may be attributed to more Lewis basic coordination to the rhodium centre. However, it is noteworthy that halide substituents (**5e–5f**) were well tolerated and yielded the desired products **6e–6f** (80% of **6e** and 79% of **6f**, respectively), as this is advantageous for further synthetic elaborations with transition-metal catalysis thereby broadening the diversity of the products.

### Mechanistic investigation

**Figure 8:**
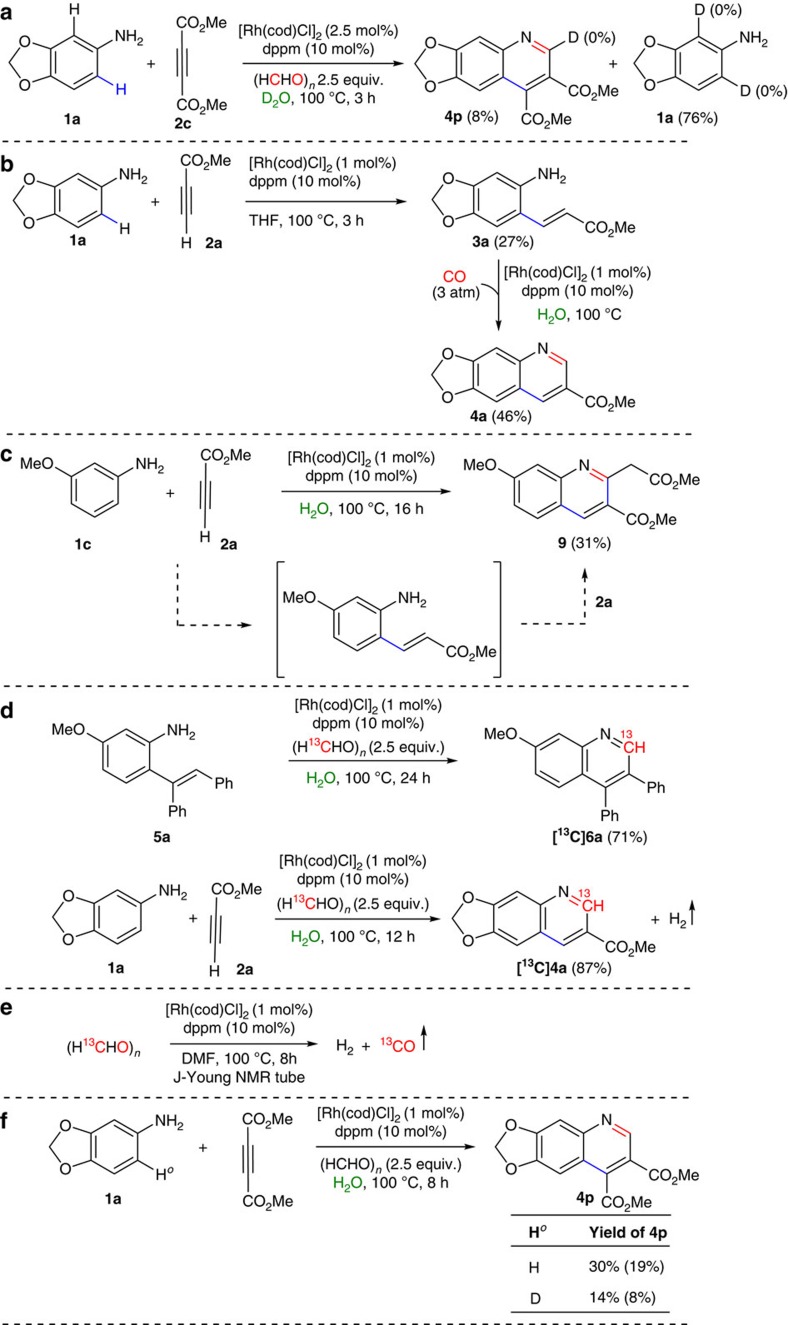
Mechanistic investigation. (**a**) Irreversibility of regioselective C–H bond activation of **1a**. (**b**) Isolation of intermediates (C–C cross-coupled product) followed by carbonylation reaction. (**c**) Reaction of **1c** with methyl propiolate (**2a**) in the absence of CO source. (**d**) Rhodium-catalysed auto-tandem reaction with labelled compounds. (**e**) Rhodium-catalysed CO formation from paraformaldehyde. (**f**) Kinetic isotopic experiments. (Yields in parentheses are isolated yields).

To gain insight into the reaction mechanism, a series of control experiments, and deuterium-labelling studies were performed (Fig. 8).

Deuterium-labelling studies clearly confirmed the *ortho*-C(*sp*^2^)–H bond cleavage of the aniline is irreversible. Thus, the reaction of **1a** with **2c** using the standard reaction conditions in D_2_O was stopped before completion (3 h). Compounds **1a** and **4p** were isolated and their deuterium content was analysed by ^1^H NMR. With both recovered compounds, no deuterium incorporation was observed suggesting that electrophilic metalation is irreversible in presence of **2c**. However, it was observed that the cyclometalation of **1a** is reversible in the absence of **2c** (Fig. 2c). Under standard reaction conditions, the C–C bonded intermediate methyl-3-(6-aminobenzo[d][1,3]dioxol-5-yl)acrylate (**3a**) was isolated in 27% from the reaction of 3,4-(methylenedioxy)aniline (**1a**) with methyl propiolate **2a** (Fig. 8b). Further, treatment of methyl-3-(6-aminobenzo[d][1,3]dioxol-5-yl)acrylate (**3a**) with a catalytic amount of [Rh] under 3 atm of carbon monoxide cleanly produced **4a** in 46% isolated yield. Notably, reaction of 3-methoxyaniline (**1c**) and methyl propiolate **2a** with a catalytic amount of [Rh] in the absence of carbonyl source with the prolonged time yielded, methyl 7-methoxy-2-(2-methoxy-2-oxoethyl)quinoline-3-carboxylate (**8**). This result strongly support that this auto-tandem catalysis is proceeding via the C–C bond formation (Fig. 8c; Supplementary Fig. 98). On the basis of the experimental details, other plausible pathways such as reaction via formation of –NHCHO, Michael addition intermediate, imine intermediate and [3,3] rearrangement have been discarded completely (Supplementary Methods). To understand the carbonylation process involving CO surrogates several labelling experiments were performed using ^13^C-labelled paraformaldehyde. Indeed, labelling experiments unambiguously illustrated the formation of carbon monoxide from paraformaldehyde and therefore, utilized as a carbonyl source. Thus, under standard reaction conditions, using ^13^C-paraformaldehyde as a carbonyl source both *ortho*-vinylaniline ((*E*)-2-(1,2-diphenylvinyl)-5-methoxyaniline (**5a)** and methyl-3-(6-aminobenzo[*d*][1,3]dioxol-5-yl)acrylate (**3a**) (*in situ* formation by the reaction of 3,4-(methylenedioxy)aniline, **1a** with methyl propiolate, **2a**) predominantly yielded the corresponding ^13^C-labelled quinoline derivatives (Fig. 8d). In addition, the reactivity of ^13^C-paraformaldehyde in the presence of the rhodium catalyst under standard condition was investigated (Fig. 8e). Indeed, after 8 h, the formation of ^13^C-labelled carbon monoxide and dihydrogen was qualitatively analysed on gas chromatography (GC) and GC–mass spectrometry (GC–MS) studies, and thus demonstrating the slow release of CO. However, in the presence of water as a reaction medium ^13^CO_2_ and H_2_ were detected on GC and GC–MS (Supplementary Figs 95 and 96). This is probably due to water–gas shift reaction^48^.

**Figure 9:**
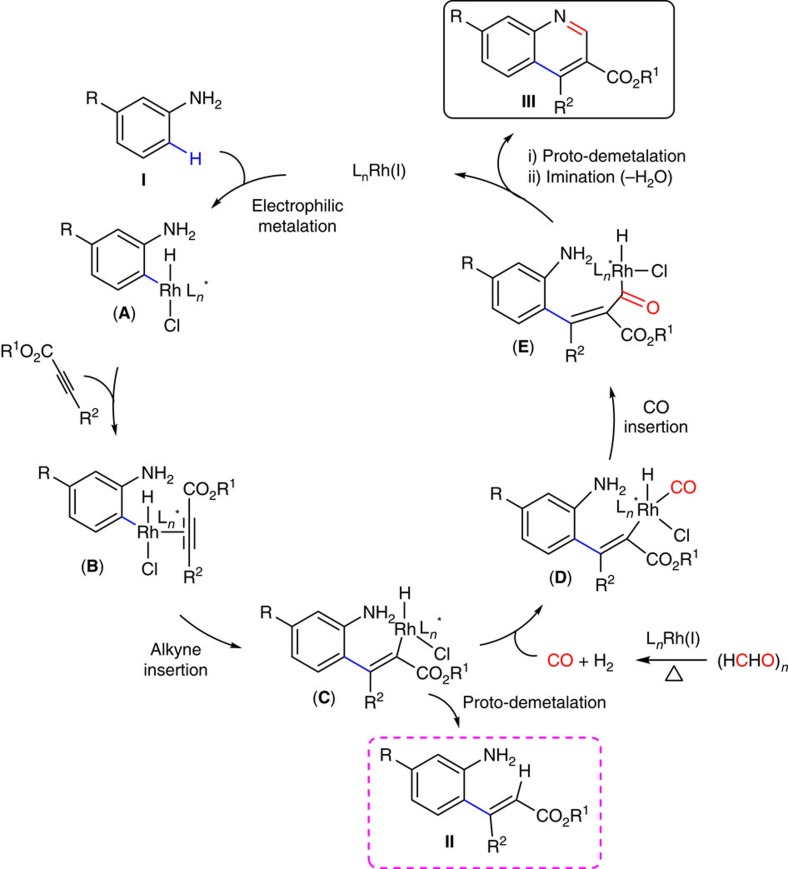
Mechanistic rationale for the rhodium-catalysed auto-tandem construction of quinoline. Proposed catalytic cycles.

On the basis of the above experimental findings and literature the precedent, we have proposed a plausible mechanism for the auto-tandem catalysis consisting of three mechanically distinct reactions, such as C–C cross-coupling via non-directed C–H activation, CO generation from CO surrogates and sequential carbonylation followed by annulation reaction catalysed by the single-site rhodium catalyst (Fig. 9). The electrophilic metalation of aniline can provide the *ortho*-C–H bond activated product **A** (Supplementary Fig. 97). Interaction of an alkyne with intermediate **A** can lead to intermediate **B** and subsequently alkyne insertion can lead to intermediate **C**. However, intermediate **C** can undergo proto-demetalation to provide the *ortho*-vinylaniline (**II**) in the absence of carbonyl source. Reaction of intermediate **C** with carbon monoxide (*in situ* generated from CO surrogates) can lead to **E** via intermediate **D**. This is followed by the proto-demetalation of intermediate **E** and intramolecular imination to provide the expected quinoline moiety **III**.

## Discussion

Reversed reactivity of an aniline with an electron-deficient alkyne in the rhodium catalysis to lead to the formation of C–C coupled product as a potential intermediate in contrast to the archetypical C–N bonded products is disclosed. The product from this complementary approach (non-directed C–H activation strategy of free anilines) is integrated with sequential carbonylation and annulation reaction in an auto-tandem manner to lead to the 3-substituted quinolines with high rigio- and chemioselectivity is reported. This auto-tandem reaction operates under mild, environmentally benign conditions using water as a solvent and CO surrogates as a carbonyl source with extremely good atom-efficiency (only H_2_ and H_2_O as by-products). Beyond the unique reactivity of this strategy, we anticipate that this auto-tandem catalysis will open a new avenue in the designing of new catalytic process for an efficient and a sustainable production of valuable targeted scaffolds from simple, feedstock chemicals in an atom-economic way.

## Methods

### General procedure for this rhodium-catalysed auto-tandem reaction

To a 10-ml clean, oven-dried screw cap reaction tube was added [Rh(cod)Cl]_2_ (2.5 mol%), dppm 1,1-bis(diphenylphosphino)methane) (10 mol%), an aniline (0.25 mmol), CO surrogate (paraformaldehyde) (0.75 mmol), an alkyne (0.275 mmol) and water (250 μl) under argon atm. The reaction mixture was kept for heating at 100 °C for a specified time. After cooling to room temperature, reaction mixture was diluted with water (6 ml) and extracted with ethyl acetate (3 × 5 ml). The resultant organic layer was dried over anhydrous Na_2_SO_4_ and the solvent was evaporated under reduced pressure. The crude mixture was purified by silica gel column chromatography (230–400 mesh size) using petroleum-ether/ethyl acetate as an eluting system.

### General procedure for rhodium-catalysed carbonylation of *ortho*-vinylanilines

To a 10-ml clean, oven-dried screw cap reaction tube was added [Rh(cod)Cl]_2_ (2.5 mol%), dppm (10 mol%), *ortho*-vinylaniline (**5a–g**) (0.25 mmol), paraformaldehyde (0.75 mmol, 2.5 equiv.) and water (250 μl) under argon atm. The reaction mixture was heated at 100 °C for specified hours. After cooling at room temperature, reaction mixture was diluted with water (6 ml) and extracted with ethyl acetate (3 × 5 ml). The combined organic layer was dried over anhydrous Na_2_SO_4_ and the solvent was evaporated. The crude product was purified by silica gel column chromatography (230–400 mesh size) using petroleum-ether/ethyl acetate as an eluent. All new compounds were fully characterized. For NMR and high-resolution mass spectrometry analysis in this article, see Supplementary Figs 3–93. General information, materials, synthesis and characterization of compounds in this article (**D[1a]**, **3a**, **3c**, **4a–4q**, **6a–6g** and **7–9**), and experimental part for mechanistic investigations see Supplementary Methods.

## Additional information

**How to cite this article:** Midya, S. P. *et al*. Reversed reactivity of anilines with alkynes in the rhodium-catalysed C–H activation/carbonylation tandem. *Nat. Commun.* 6:8591 doi: 10.1038/ncomms9591 (2015).

## Supplementary Material

Supplementary Information Supplementary Figures 1-98, Supplementary Tables 1-6, Supplementary Methods and Supplementary References

## Figures and Tables

**Figure 1:**
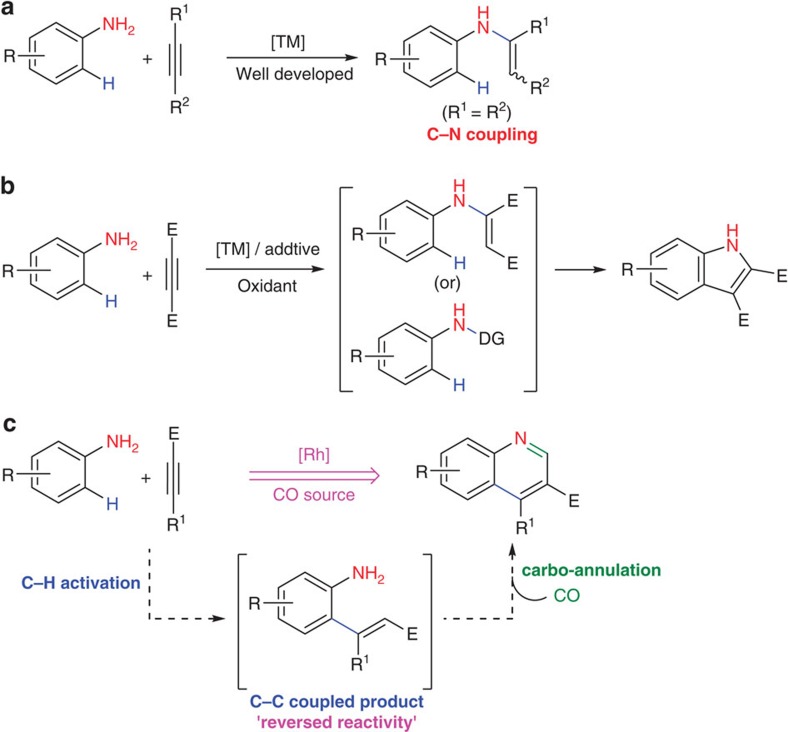
Reactivity of unprotected anilines with alkynes. (**a**) Transition-metal-catalysed hydroamination of alkynes (a formal addition of a N–H bond across a carbon–carbon multiple bond). (**b**) Previous work involving transition-metal (TM)-catalysed oxidative C–H bond activation/annulation of anilines with alkynes to indole derivatives (DG, directing group; E, electron-withdrawing group). (**c**) In this report, reversed reactivity of anilines with alkynes in the rhodium-catalysed C–H activation/carbonylation tandem to quinoline derivatives.

**Table 1:** Optimization of rhodium-catalysed C–H bond activation of anilines with alkynes and CO (surrogates).

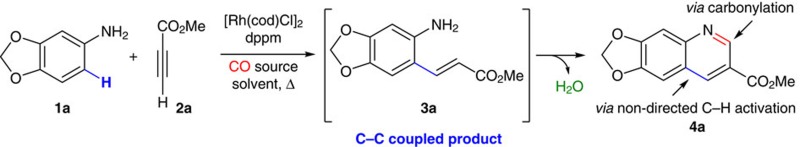			
**Entry**	**CO source**	**Solvent**	Yield (%)*
1	CO	THF	49 (42)
2	CO	H_2_O	46 (39)
3	aq. HCHO	—	52 (40)
4†	(HCHO)_*n*_	THF	62 (33)
5†	(HCHO)_*n*_	DMF	74 (19)
6†	(HCHO)_*n*_	CH_3_CN	87 (7)
7†	(HCHO)_*n*_	H_2_O	93
8†‡	(HCHO)_*n*_	H_2_O	95
9‡	CO	H_2_O	61 (32)

THF, tetrahydrofuran

Reaction conditions: 3,4-(methylenedioxy)aniline **1a** (0.1 mmol), methyl propiolate **2a** (0.11 mmol), [Rh(cod)Cl]_2_ (1 mol%), dppm (10 mol%), CO source (0.25 mmol in case of CO surrogates and/or 3 atm of CO) and 50 μl of solvent were heated at 100 °C in a closed viol for 12 h.

^*^Isolated yields and yields in parenthesis represent recovery of **1a**.

^†^A measure of 50 μl of solvent (10:1 mixture of solvent and water) and 8 h.

^‡^2.5 mol% of [Rh(cod)Cl]_2_ was used.
