# Supplementary material for: Reversed reactivity of anilines with alkynes in the rhodium-catalysed C–H activation/carbonylation tandem
Source: Nat Commun. 2015 Oct 21;6:8591. doi: 10.1038/ncomms9591 (PMC4639815; doi:10.1038/ncomms9591)
Supplement: Supplementary Information — Supplementary Figures 1-98, Supplementary Tables 1-6, Supplementary Methods and Supplementary References [file ncomms9591-s1.pdf]

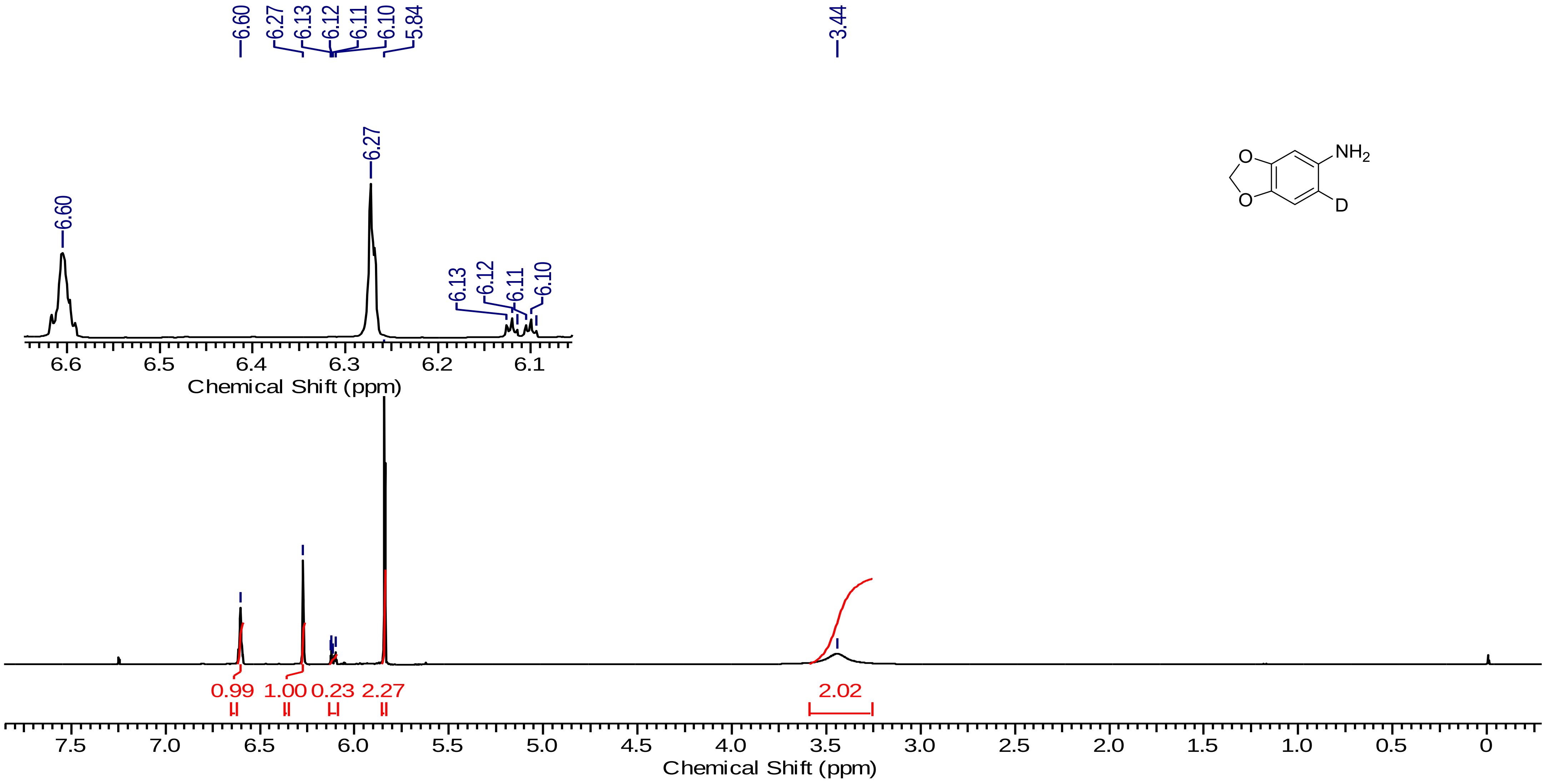

Supplementary Figure 1. ^{1}H NMR of **D[1a]**

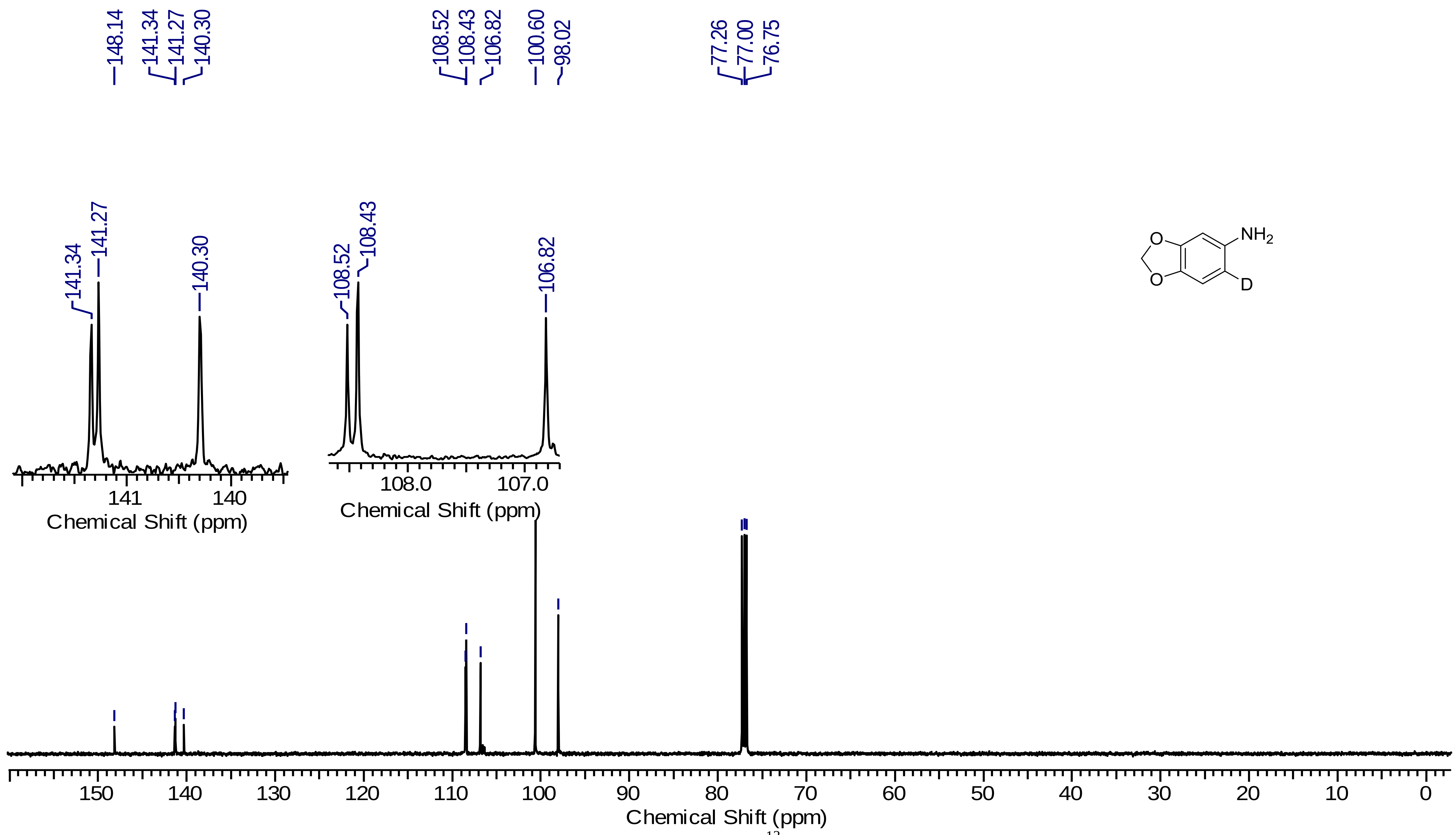

Supplementary Figure 2. ^{13}C NMR of **D[1a]**

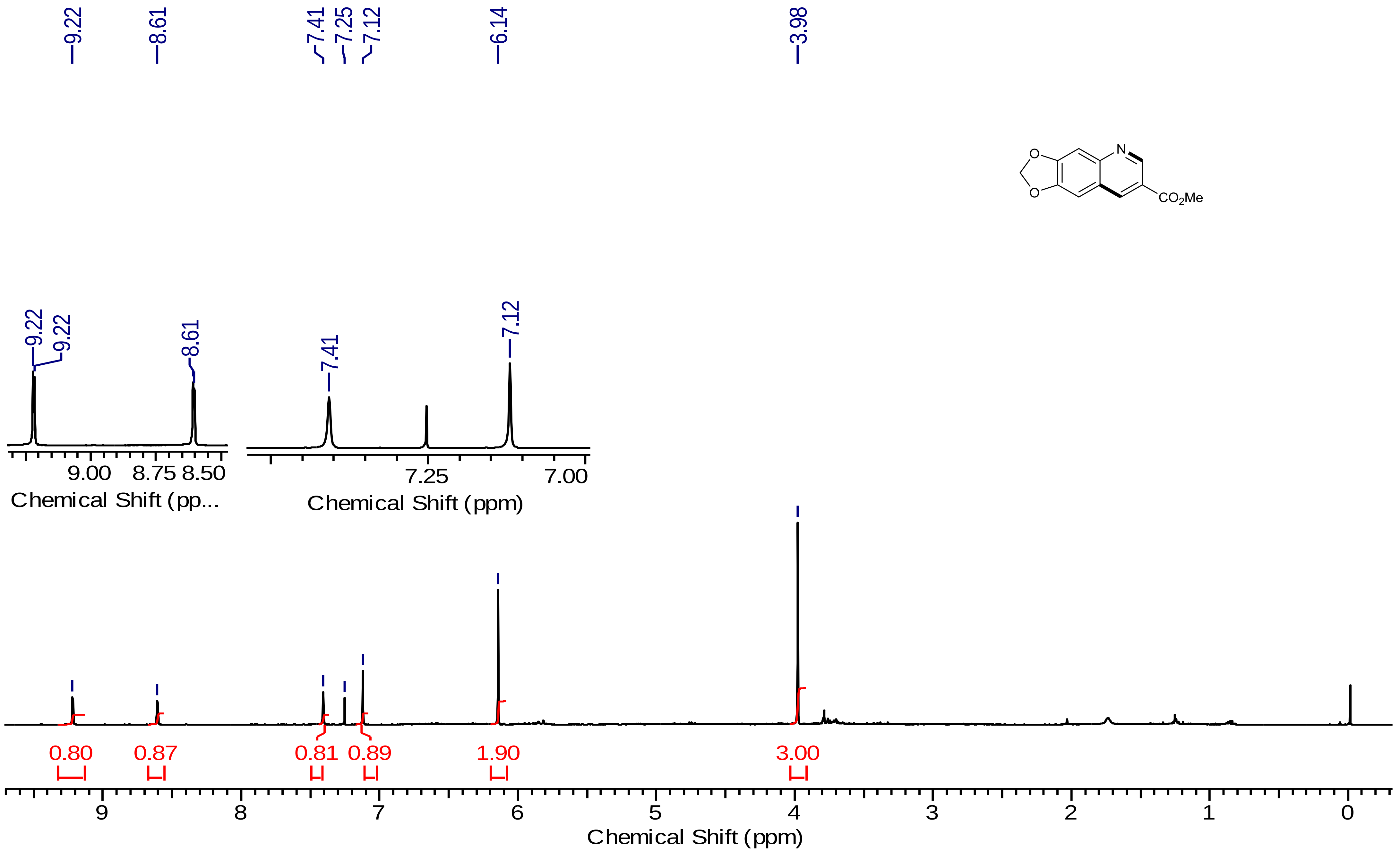

Supplementary Figure 3. ^{1}H NMR of **4a**

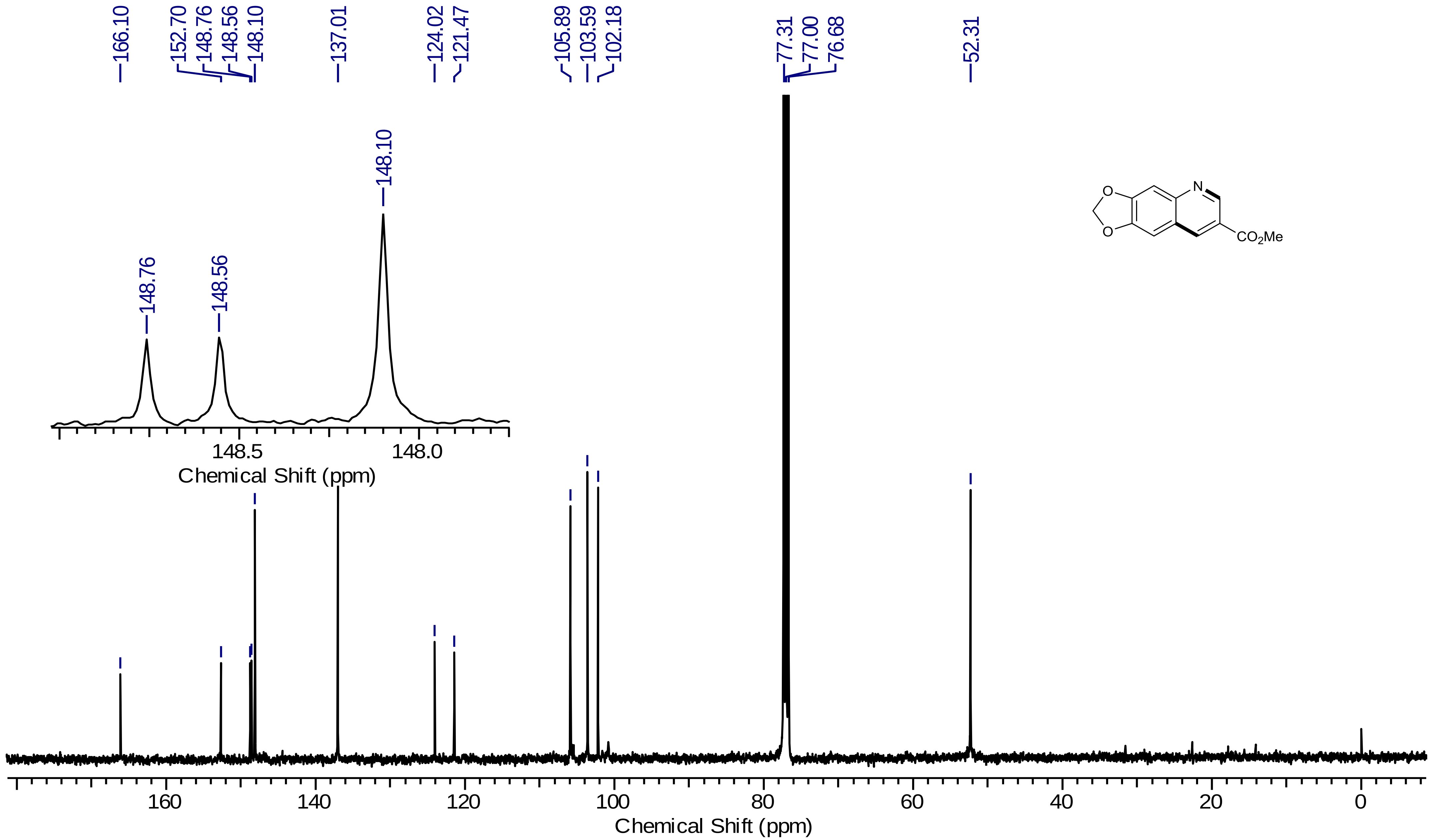

Supplementary Figure 4. ^{13}C NMR of **4a**

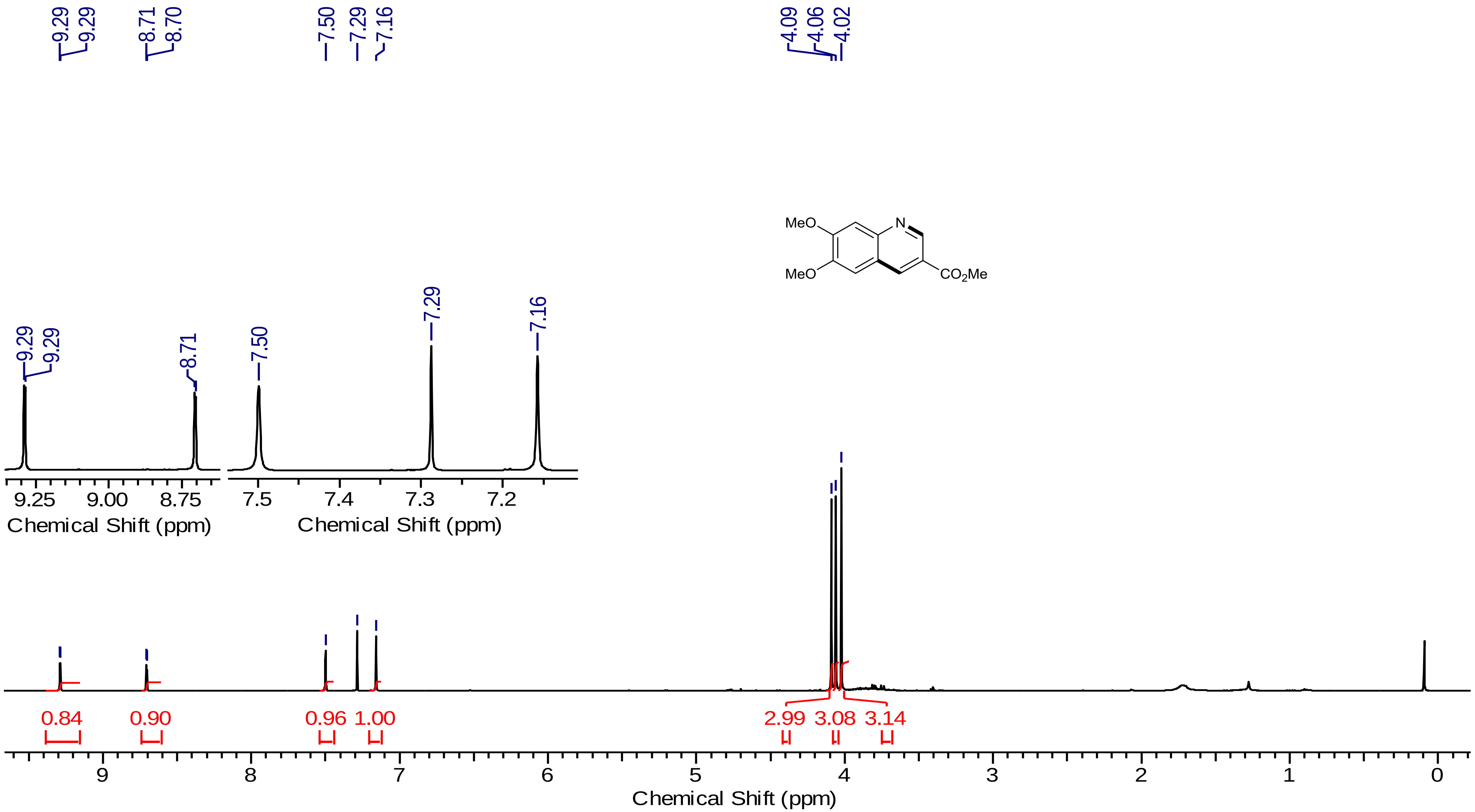

Supplementary Figure 5. ^{1}H NMR of **4b**

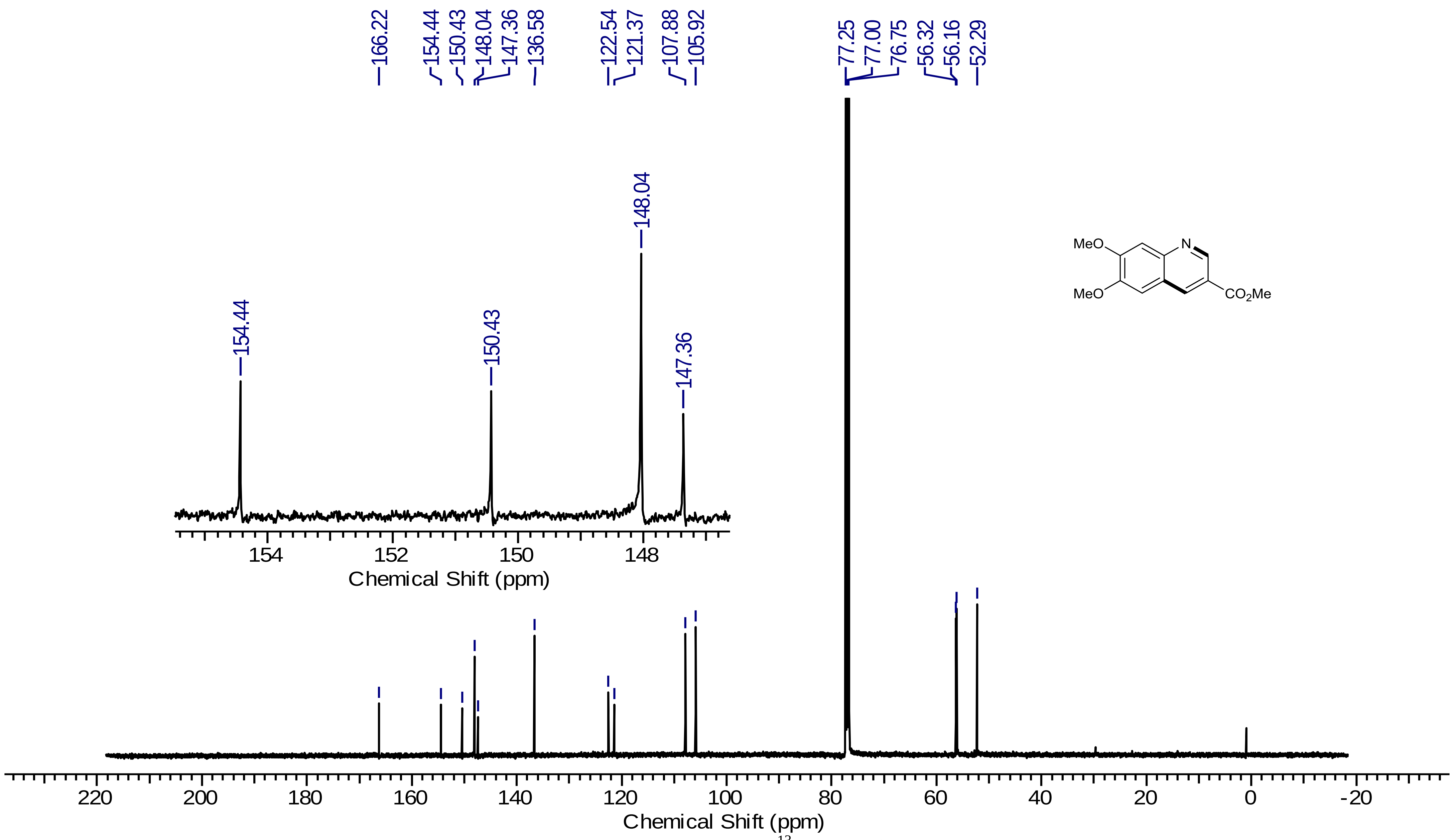

Supplementary Figure 6. ^{13}C NMR of **4b**

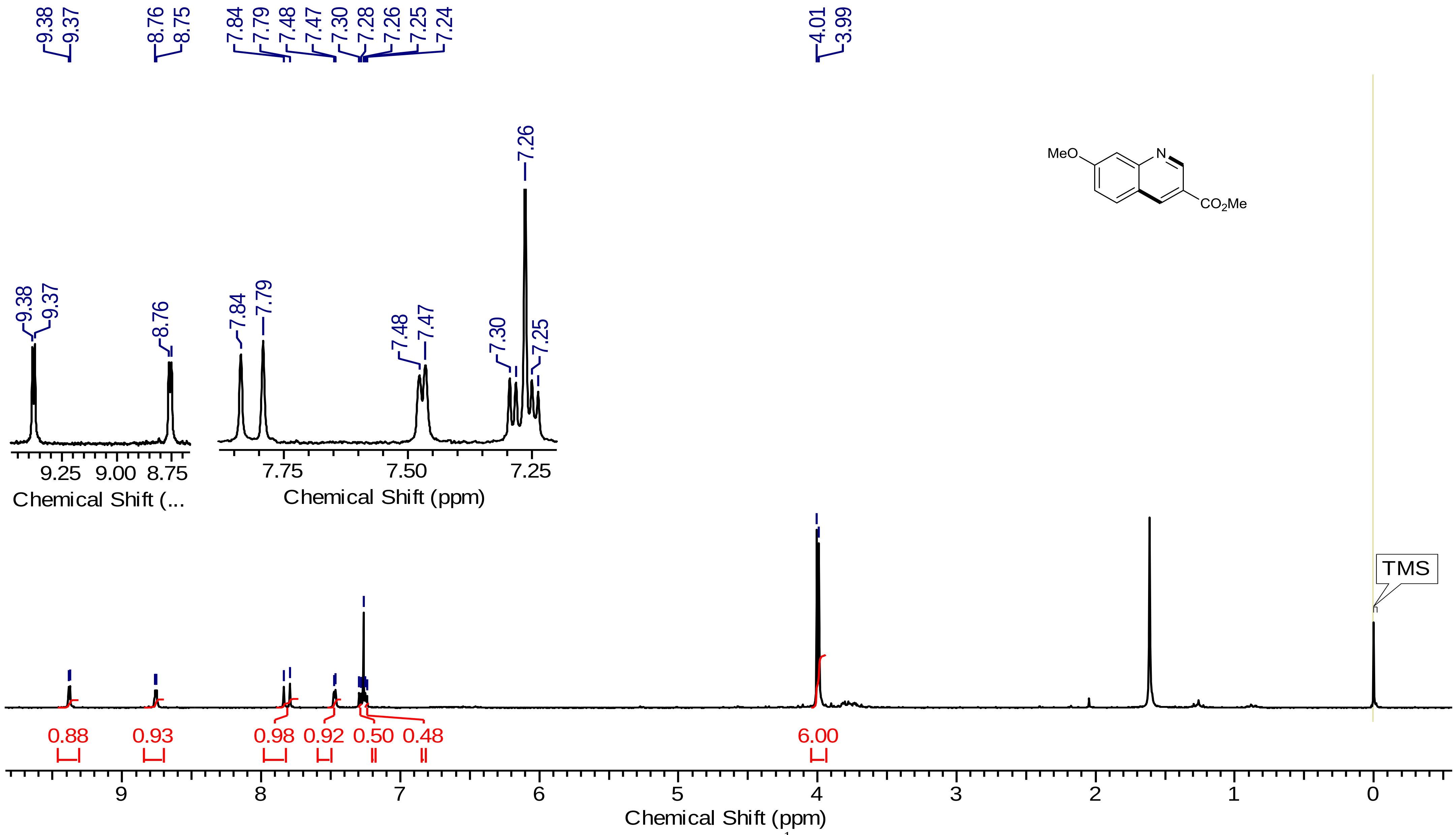

Supplementary Figure 7. ^{1}H NMR of **4c**

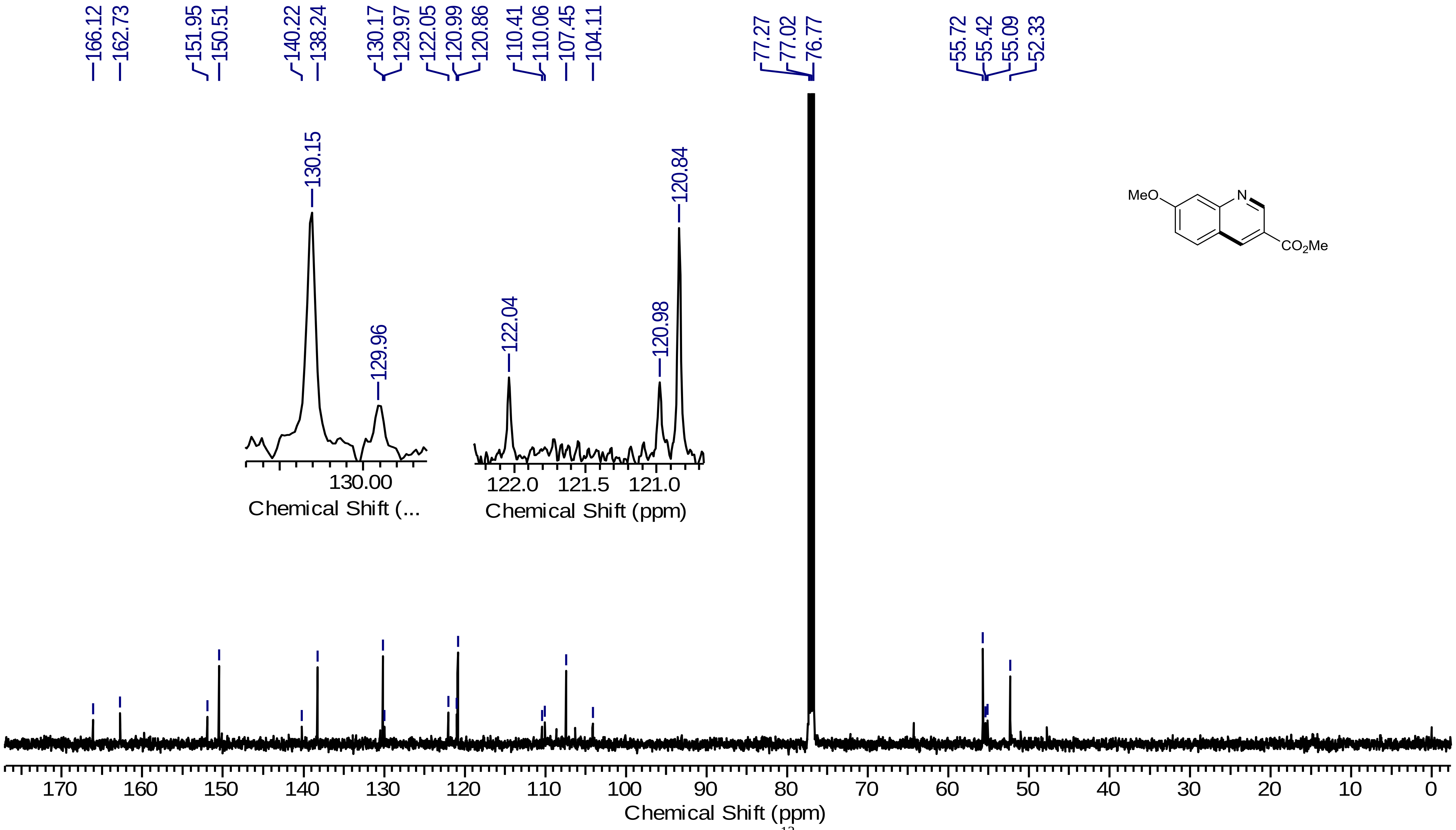

Supplementary Figure 8. ^{13}C NMR of **4c**

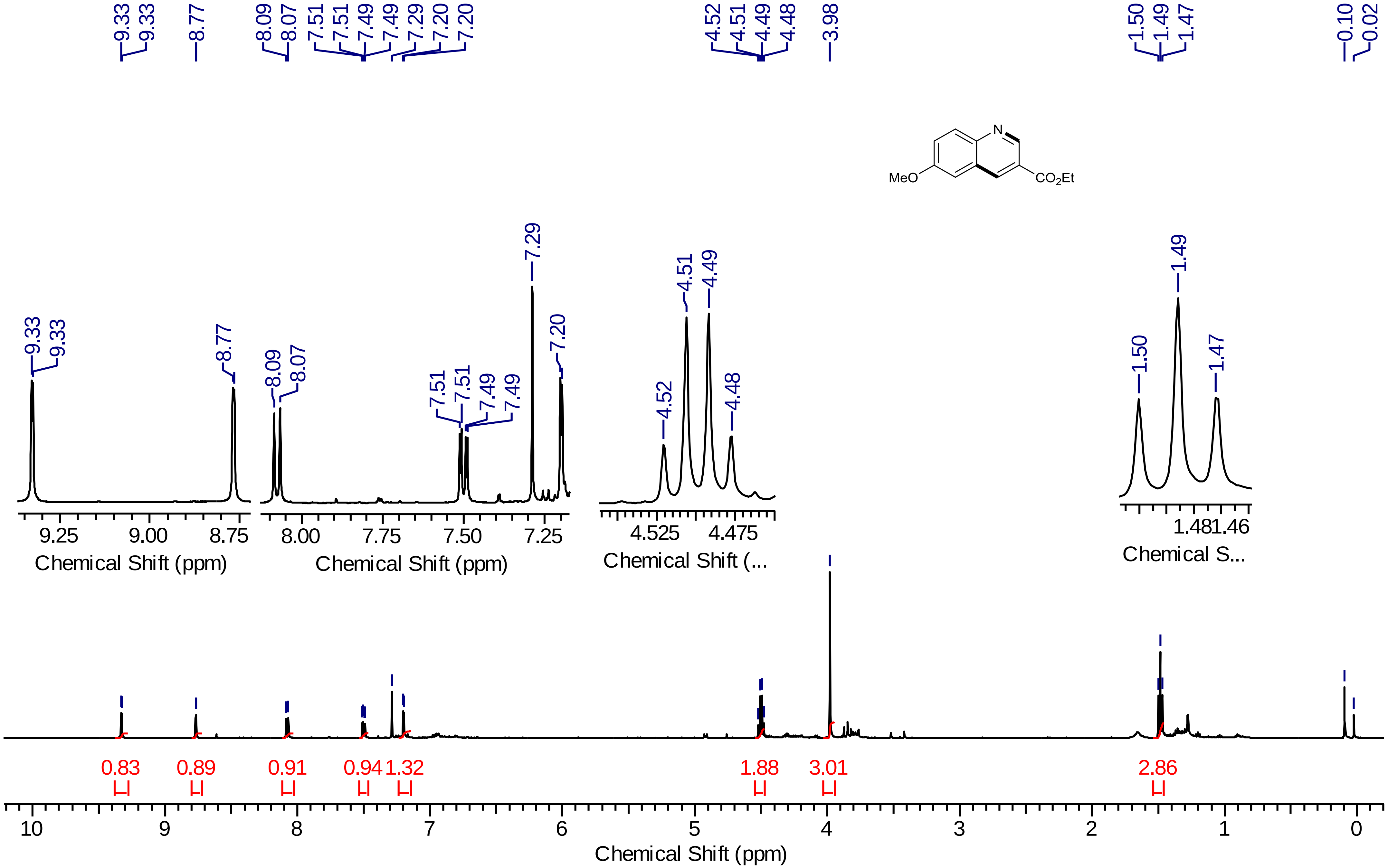

Supplementary Figure 9. ^{1}H NMR of **4d**

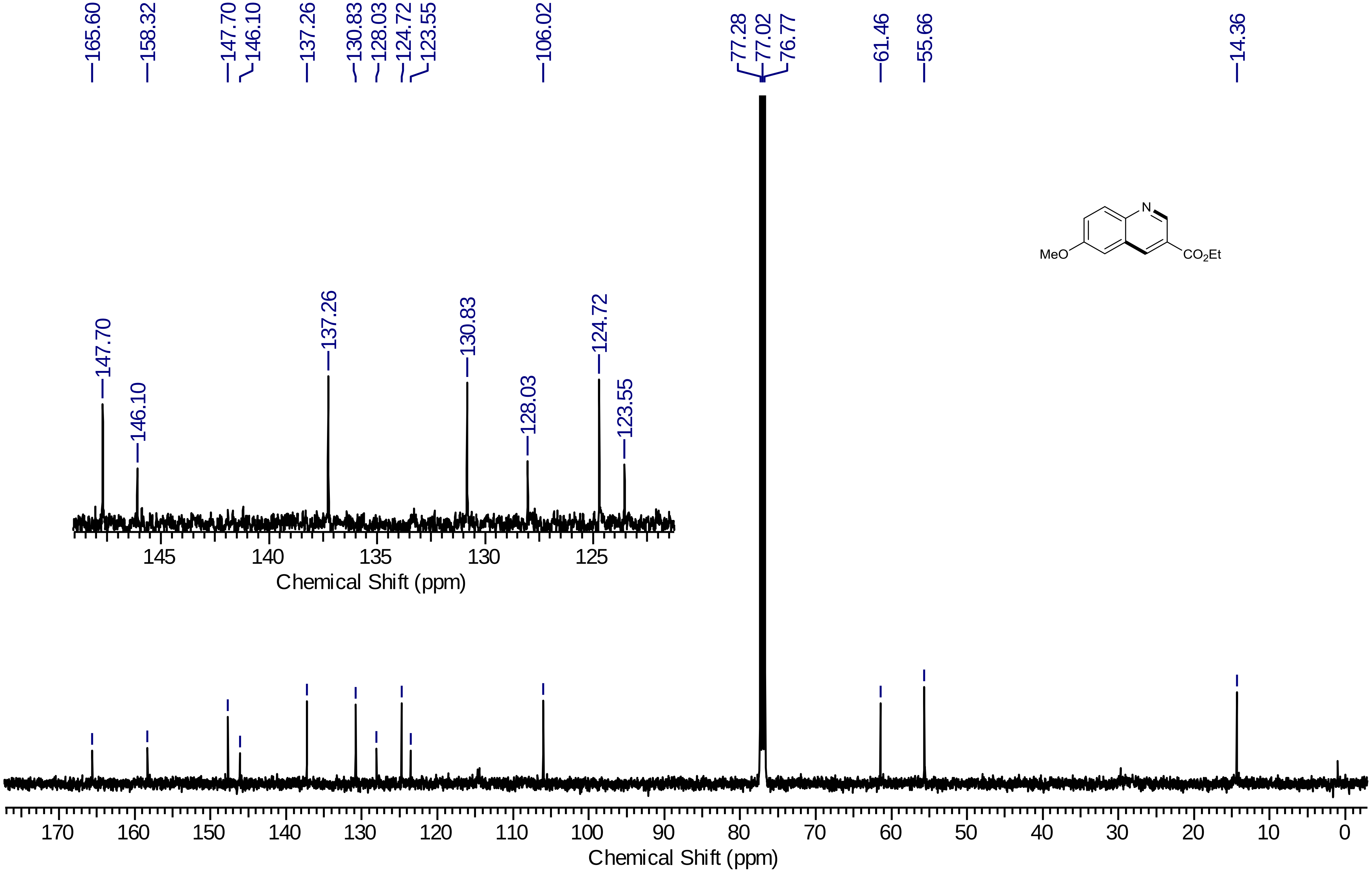

Supplementary Figure 10. ^{13}C NMR of **4d**

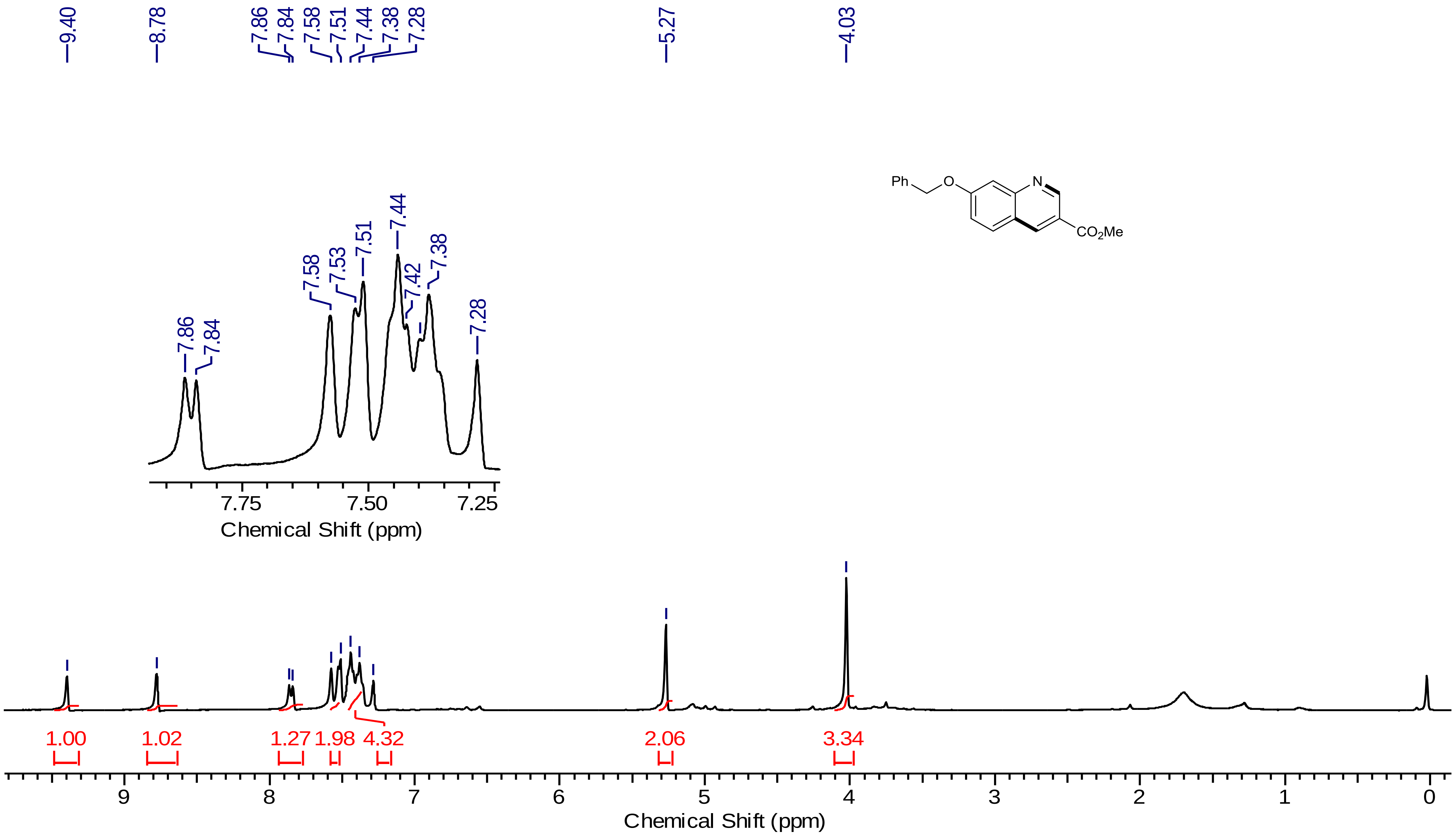

Supplementary Figure 11. ^{1}H NMR of **4e**

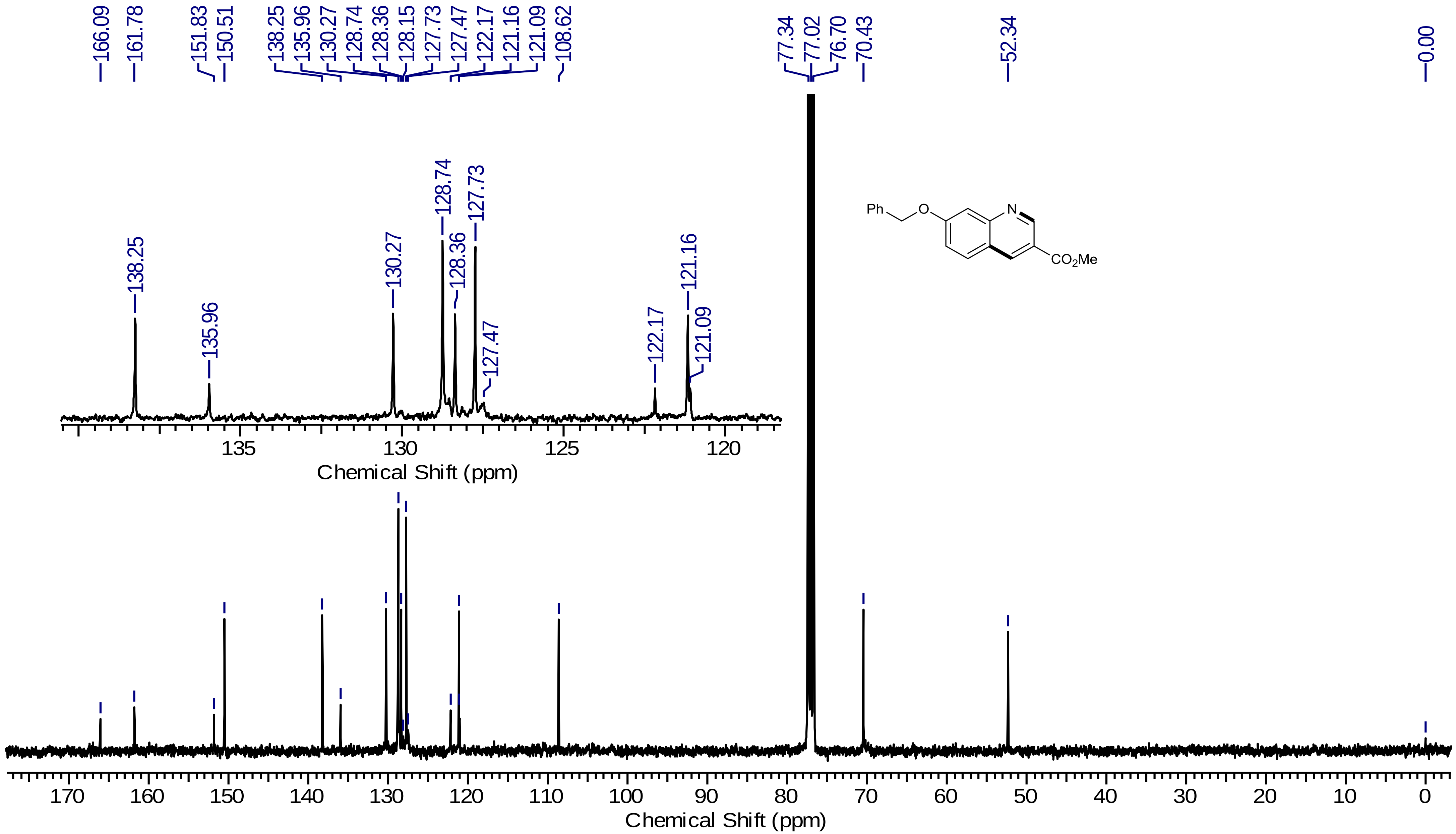

Supplementary Figure 12. ^{13}C NMR of **4e**

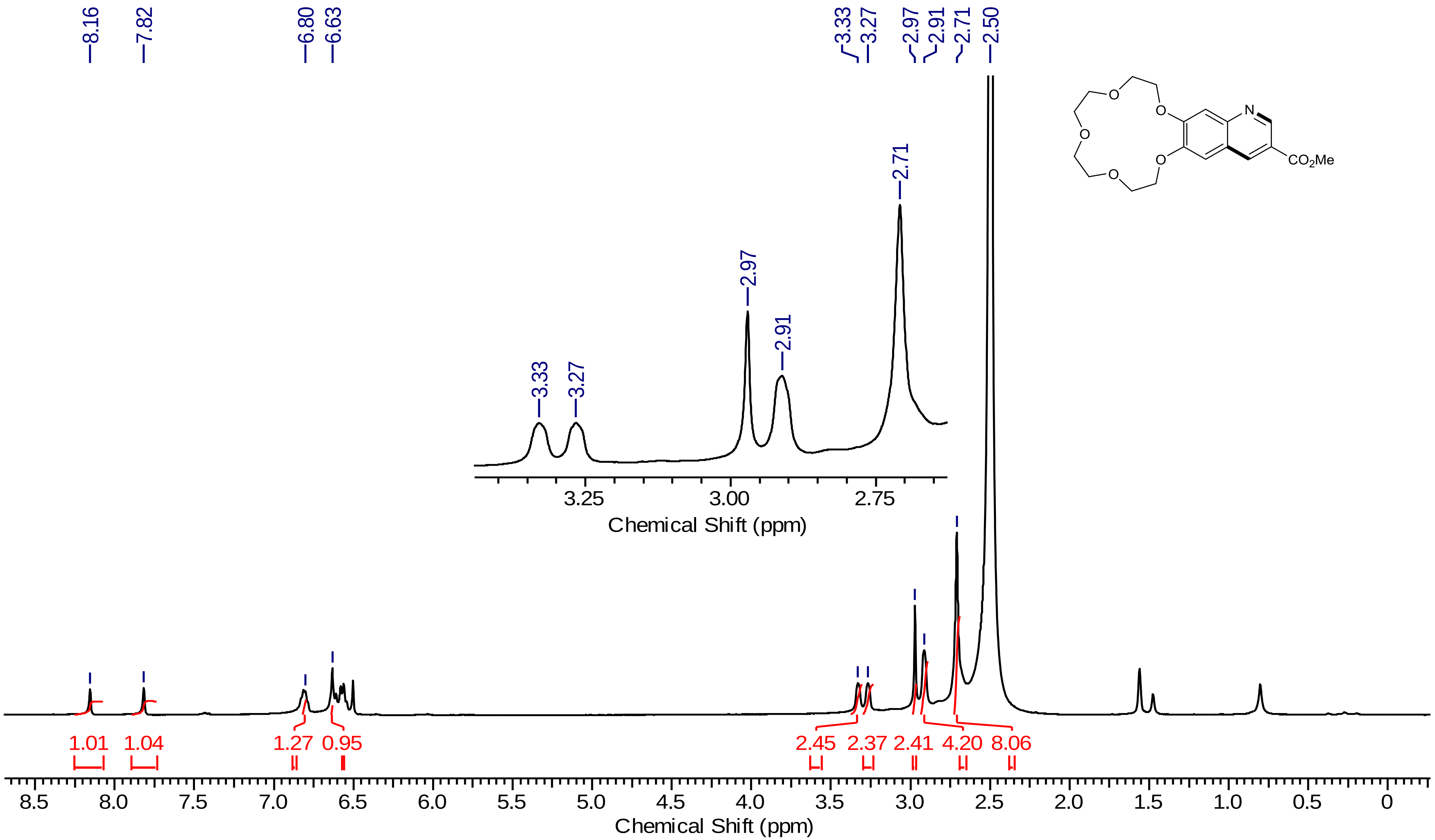

Supplementary Figure 13. ^{1}H NMR of **4f** (in DMSO-d_6)

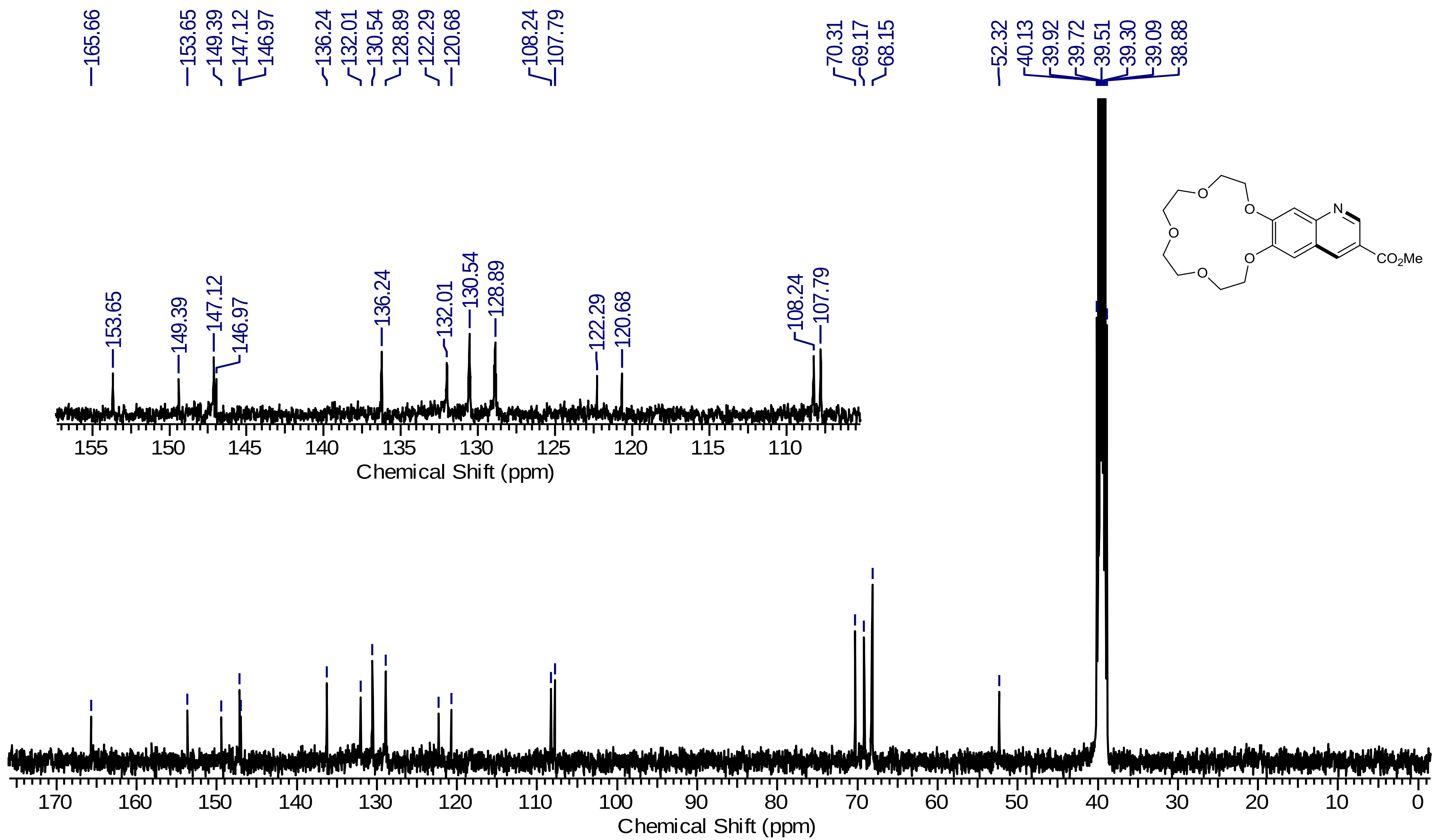

Supplementary Figure 14. ^{13}C NMR of **4f** (in DMSO-d_6)

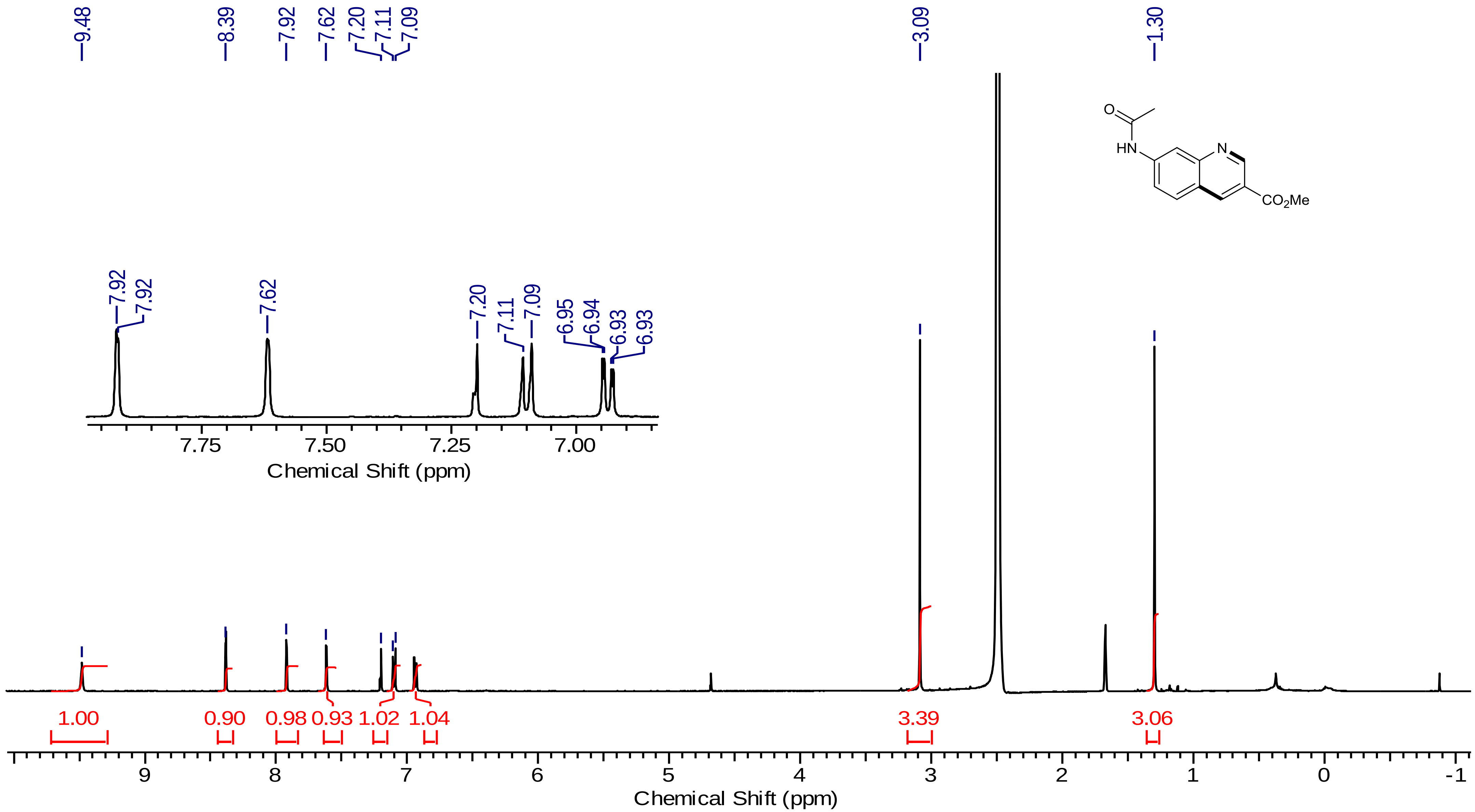

Supplementary Figure 15. ^{1}H NMR of **4g** (in 1:1 mixture of DMSO-d_6 + $CDCl_3$)

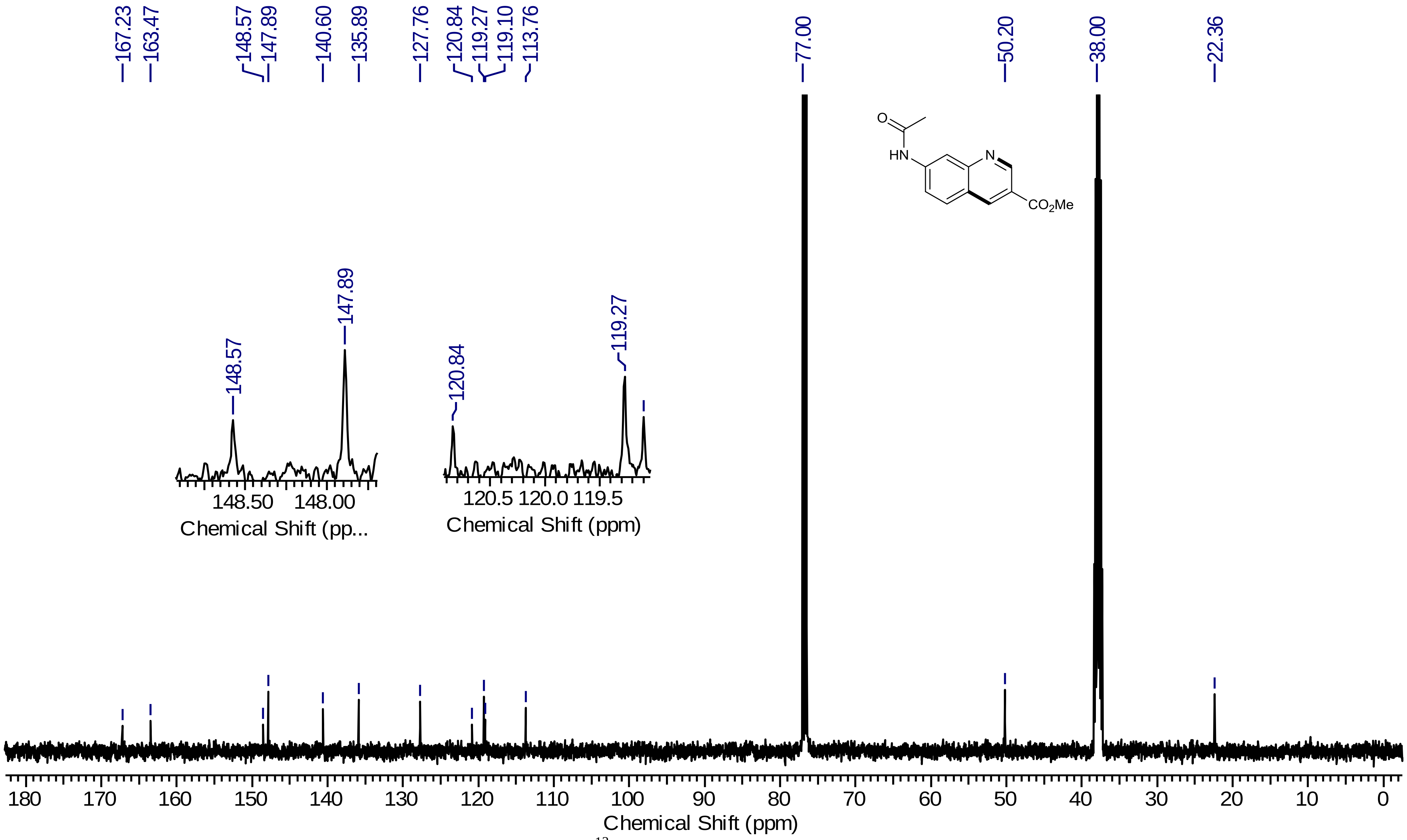

Supplementary Figure 16. ^{13}C NMR of **4g** (in 1:1 mixture of DMSO-d_6 + $CDCl_3$)

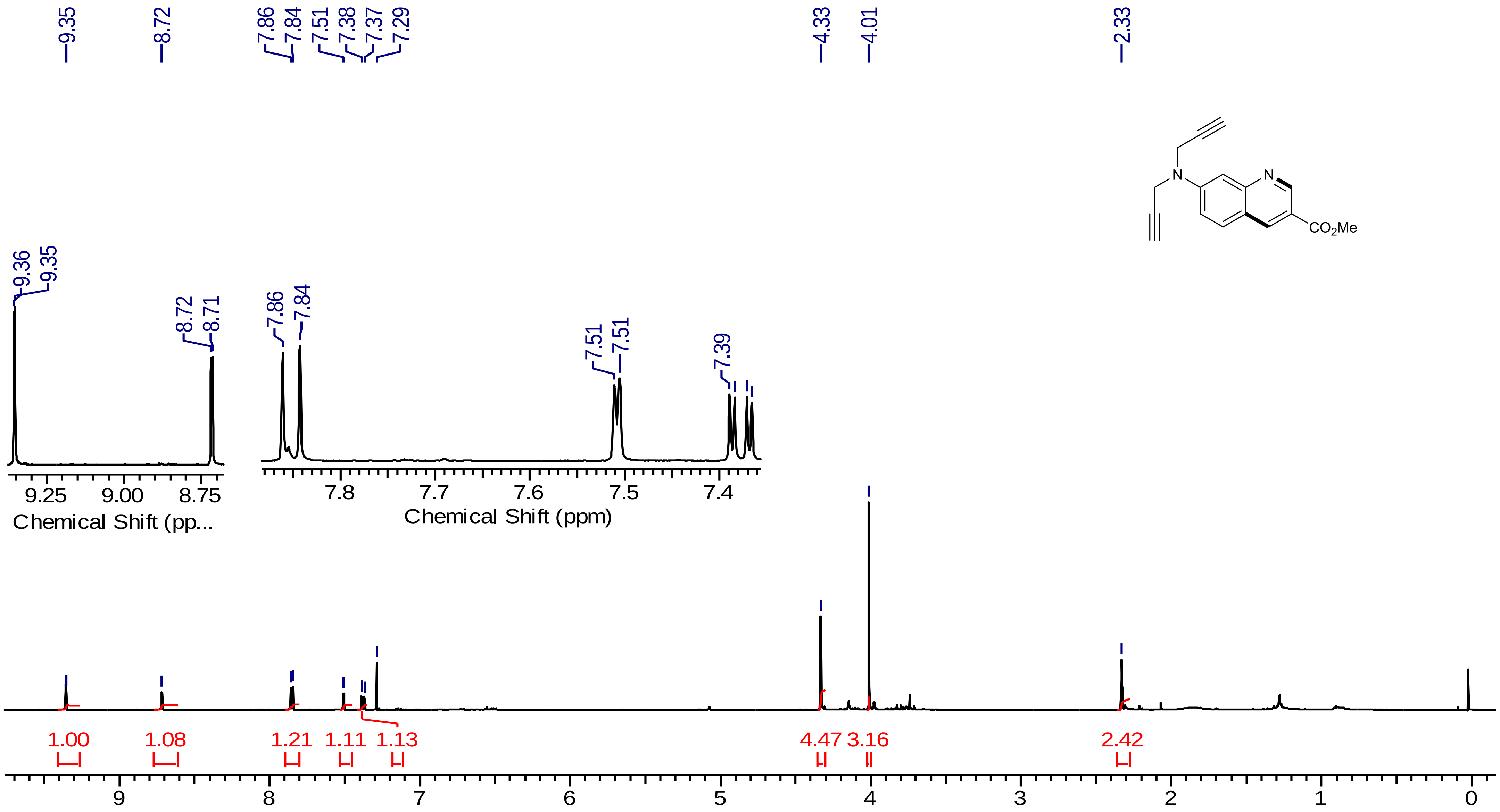

Supplementary Figure 17. ^{1}H NMR of **4h**

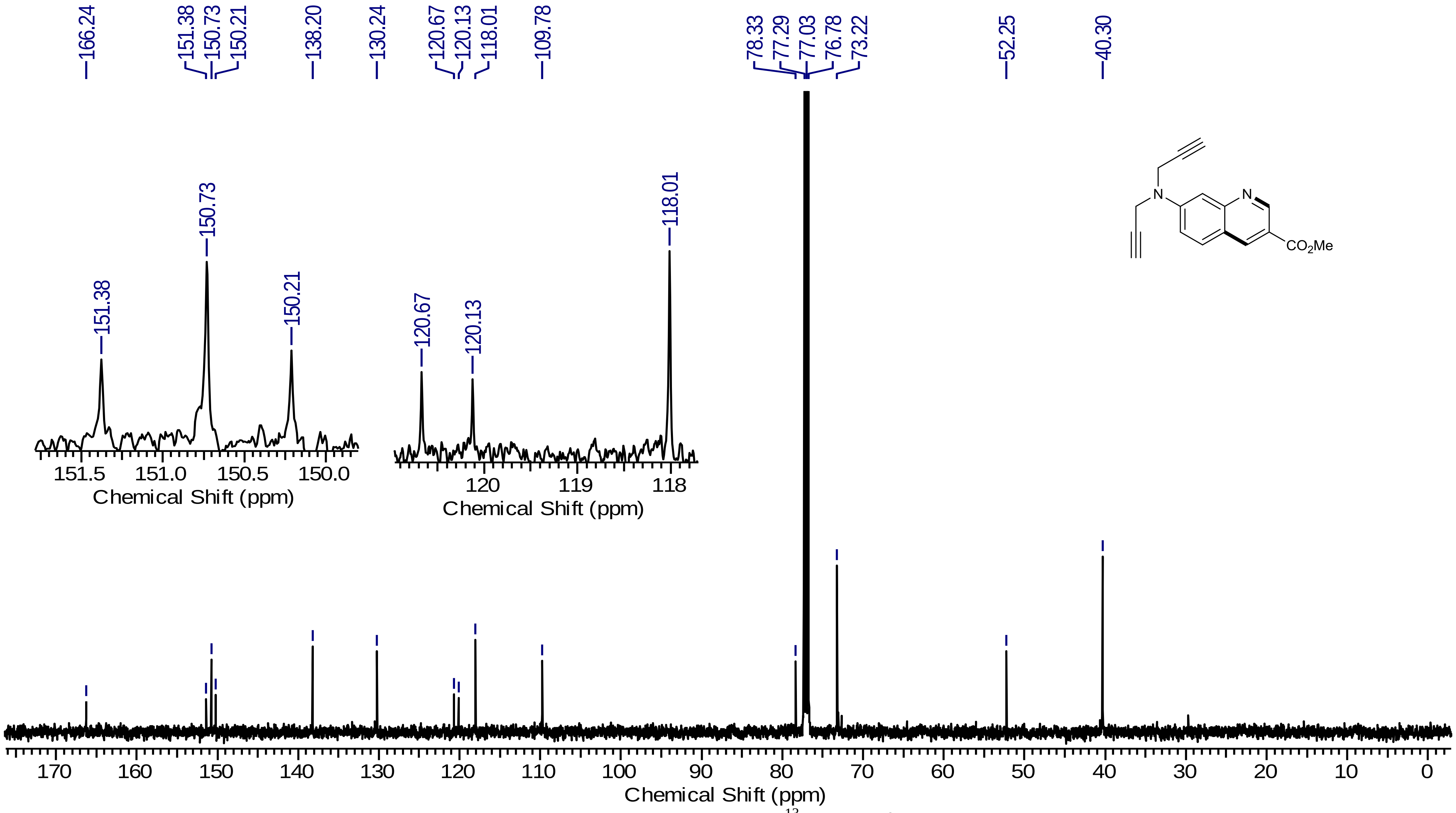

Supplementary Figure 18. ^{13}C NMR of **4h**

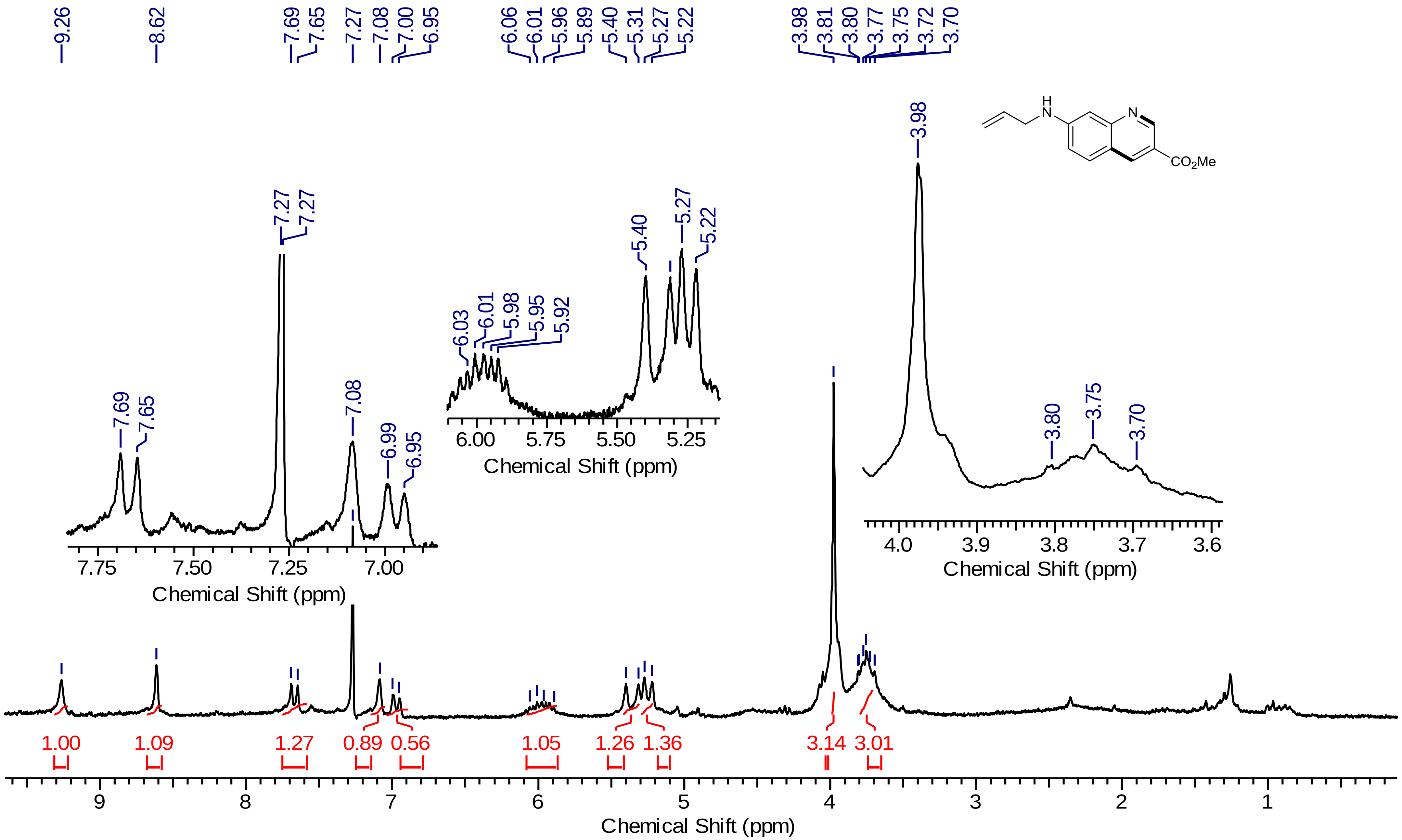

Supplementary Figure 19. ^{1}H NMR of **4i** (Due to poor solubility in $CDCl_3$ good proton NMR was not obtained)

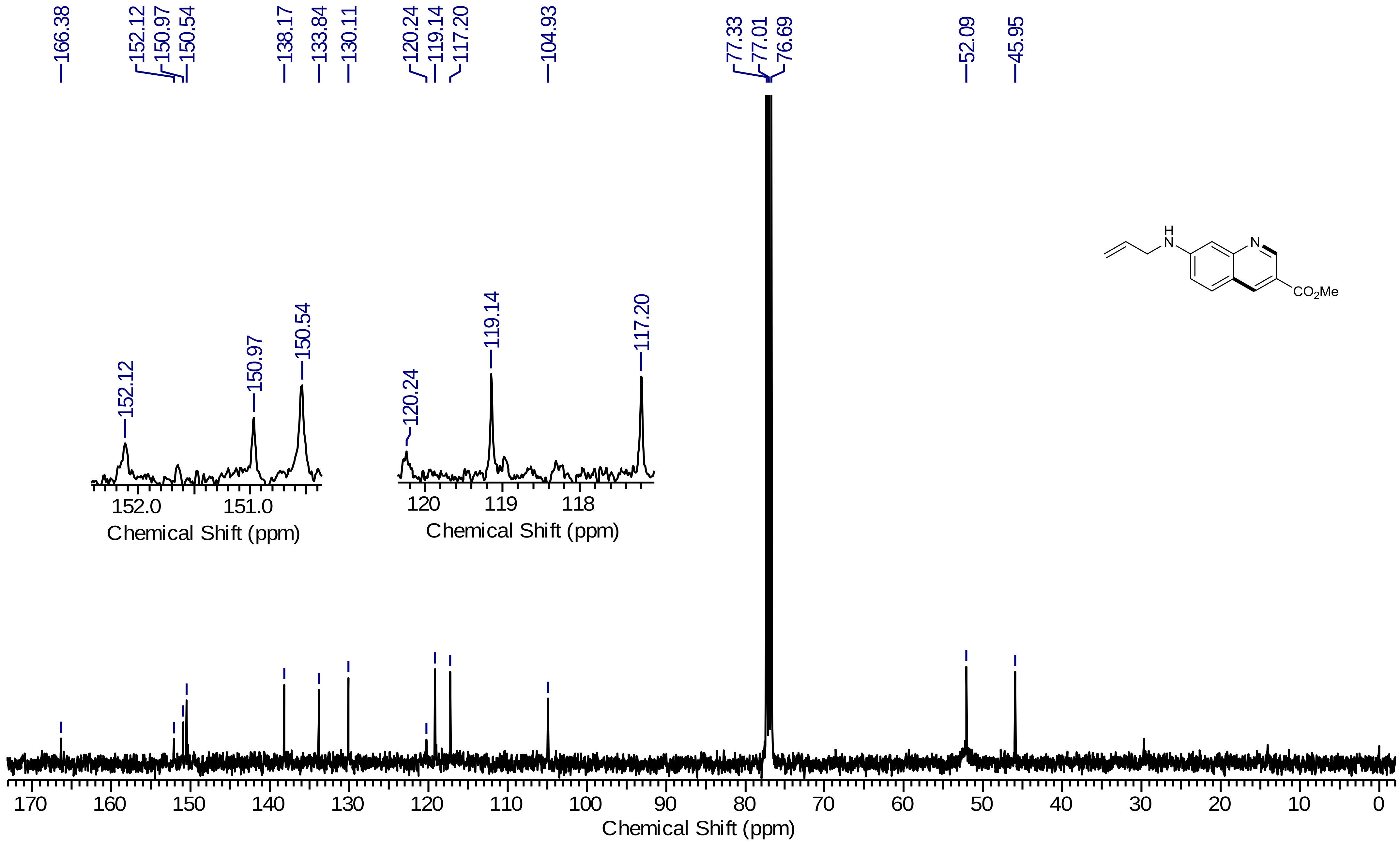

Supplementary Figure 20. ^{13}C NMR of **4i**

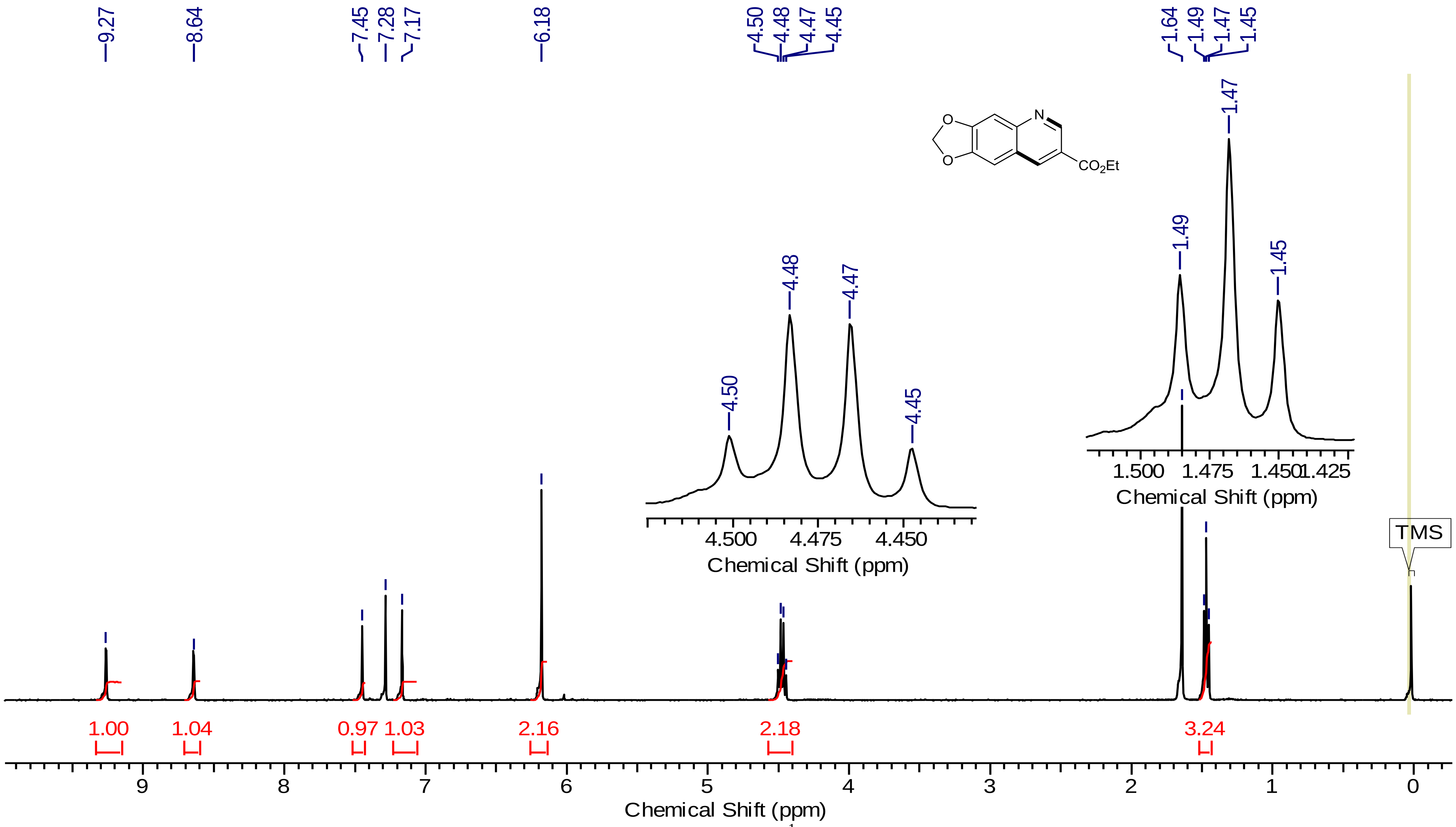

Supplementary Figure 21. ^{1}H NMR of **4k**

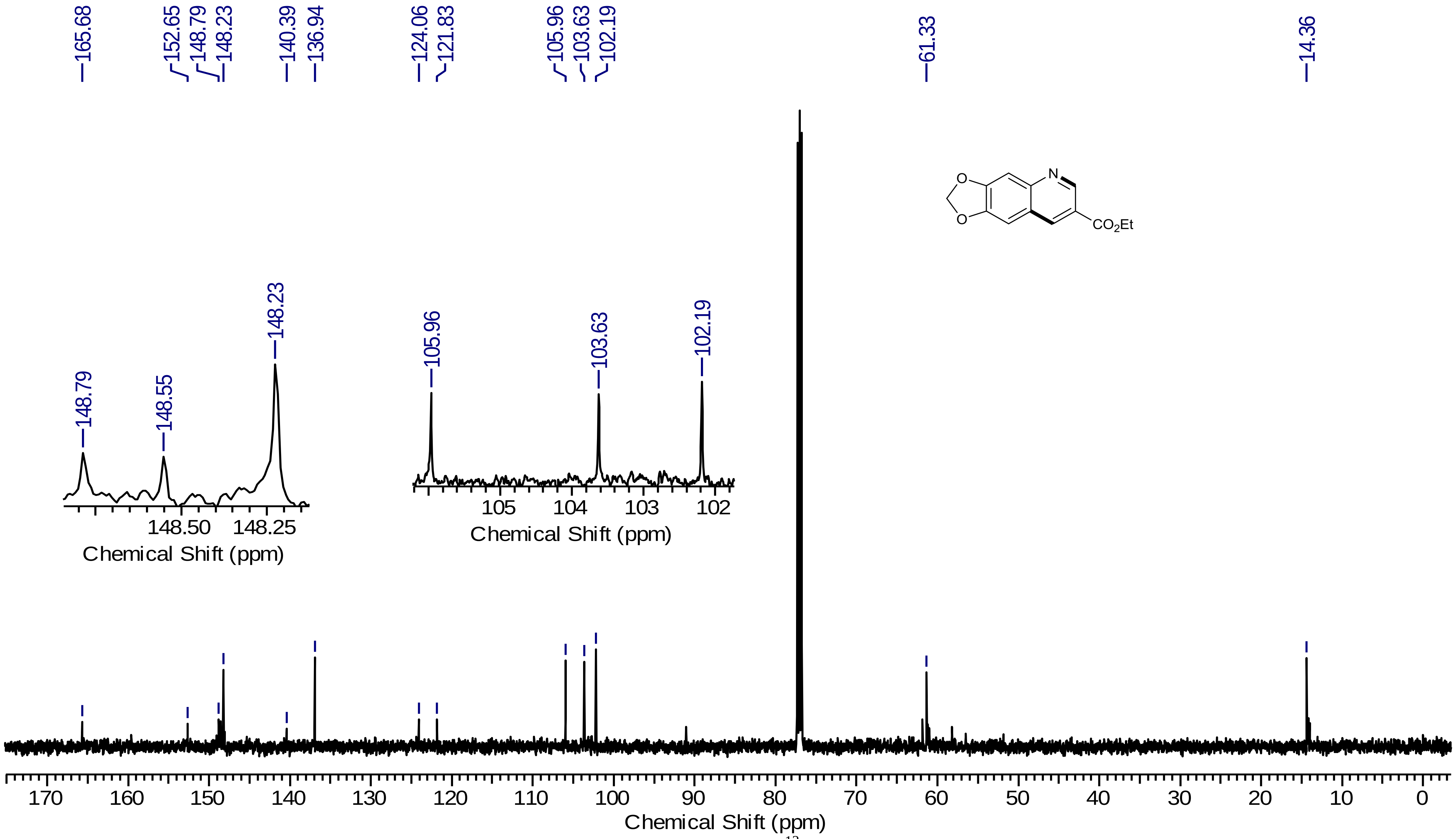

Supplementary Figure 22. ^{13}C NMR of **4k**

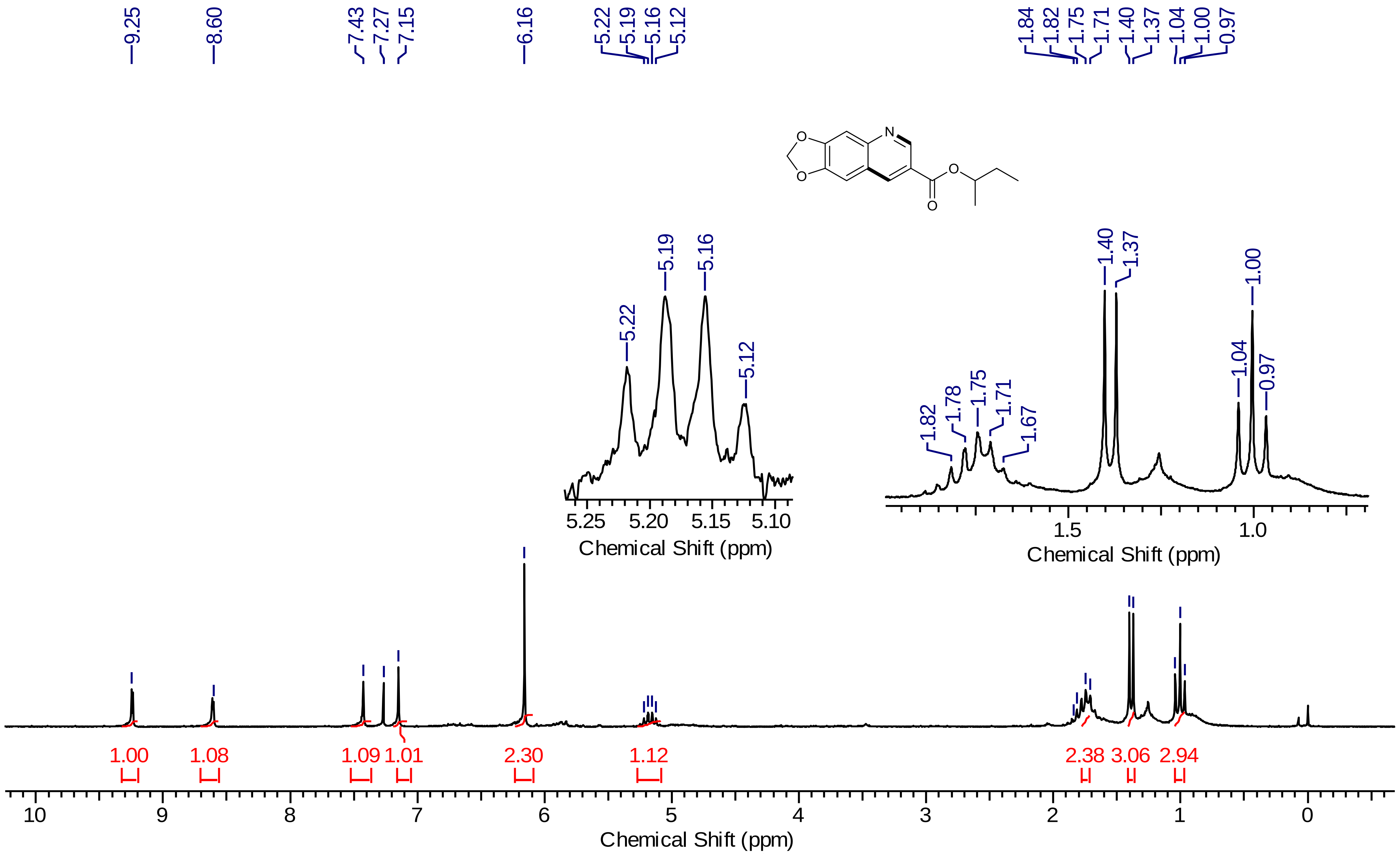

Supplementary Figure 23. ^{1}H NMR of **4l**

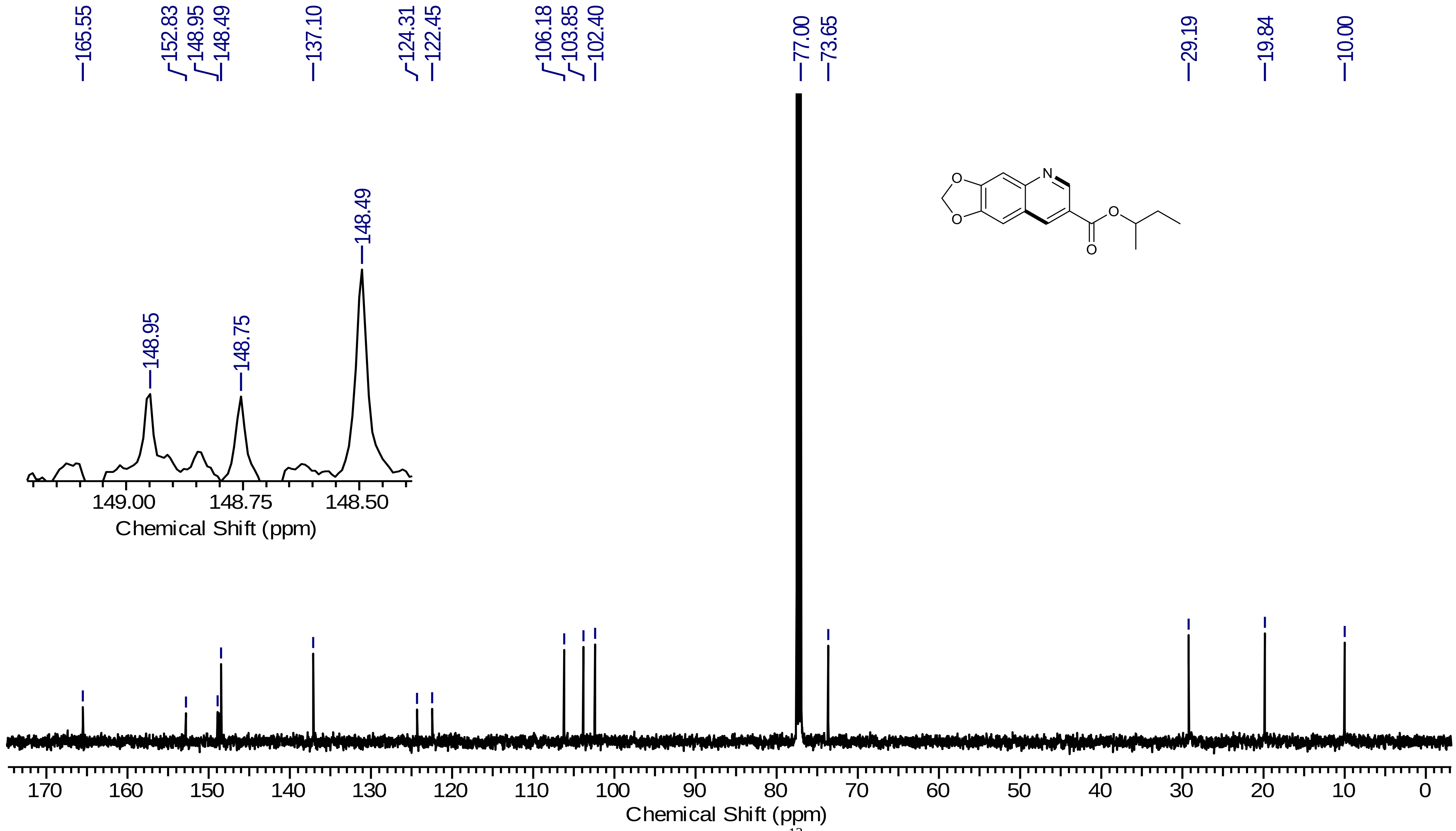

Supplementary Figure 24. ^{13}C NMR of **4l**

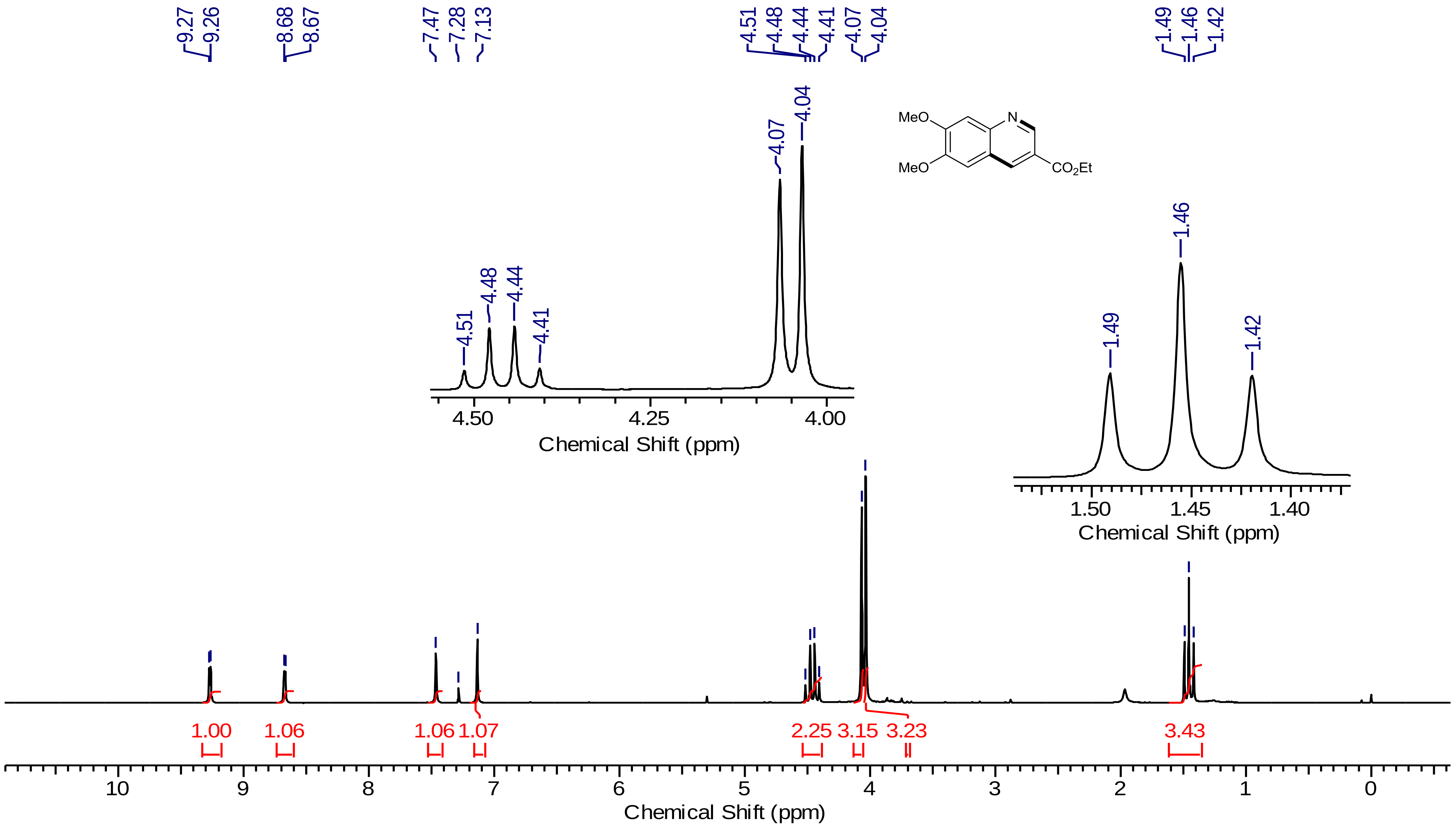

Supplementary Figure 25. ^{1}H NMR of **4m**

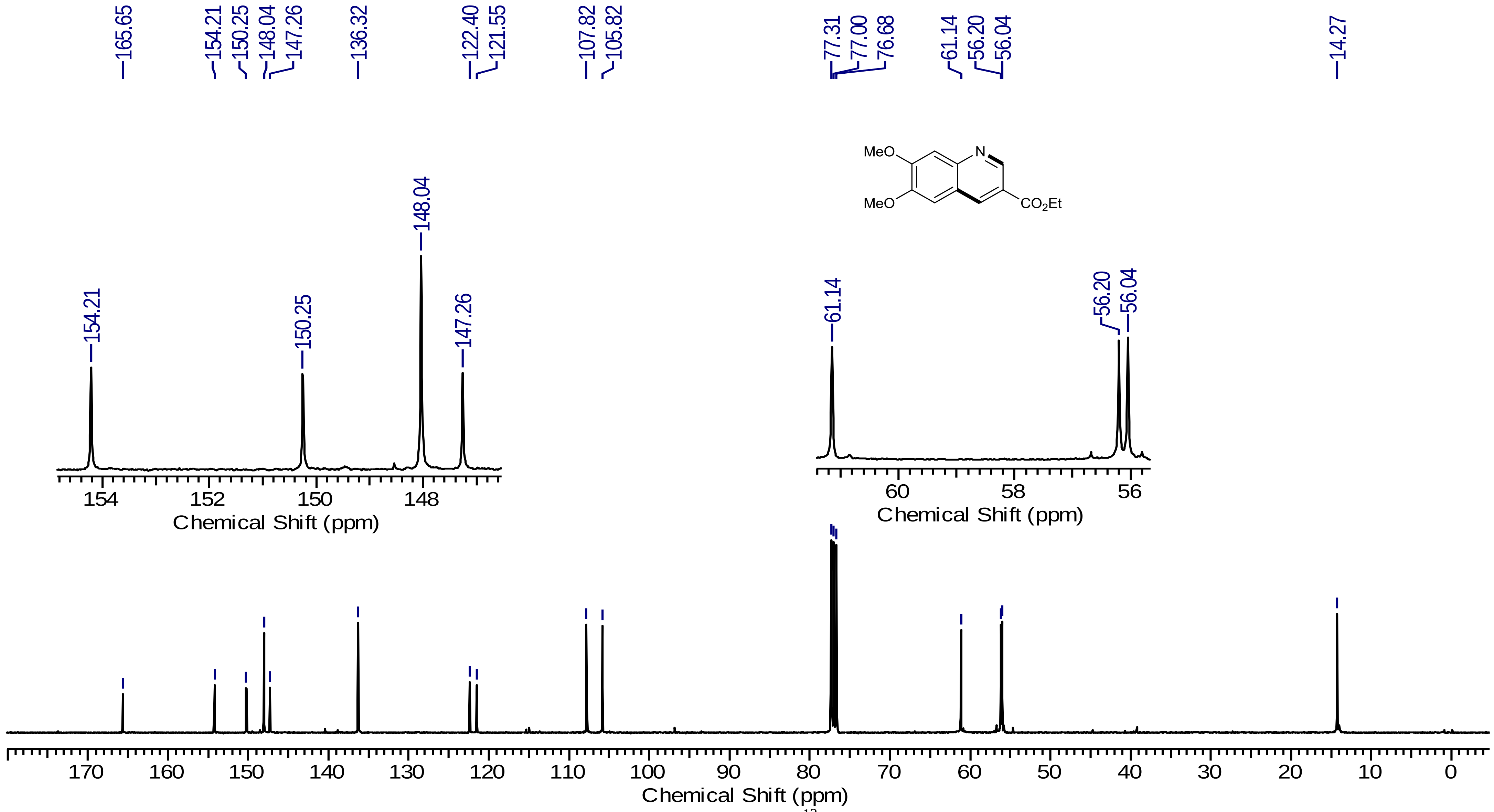

Supplementary Figure 26. ^{13}C NMR of **4m**

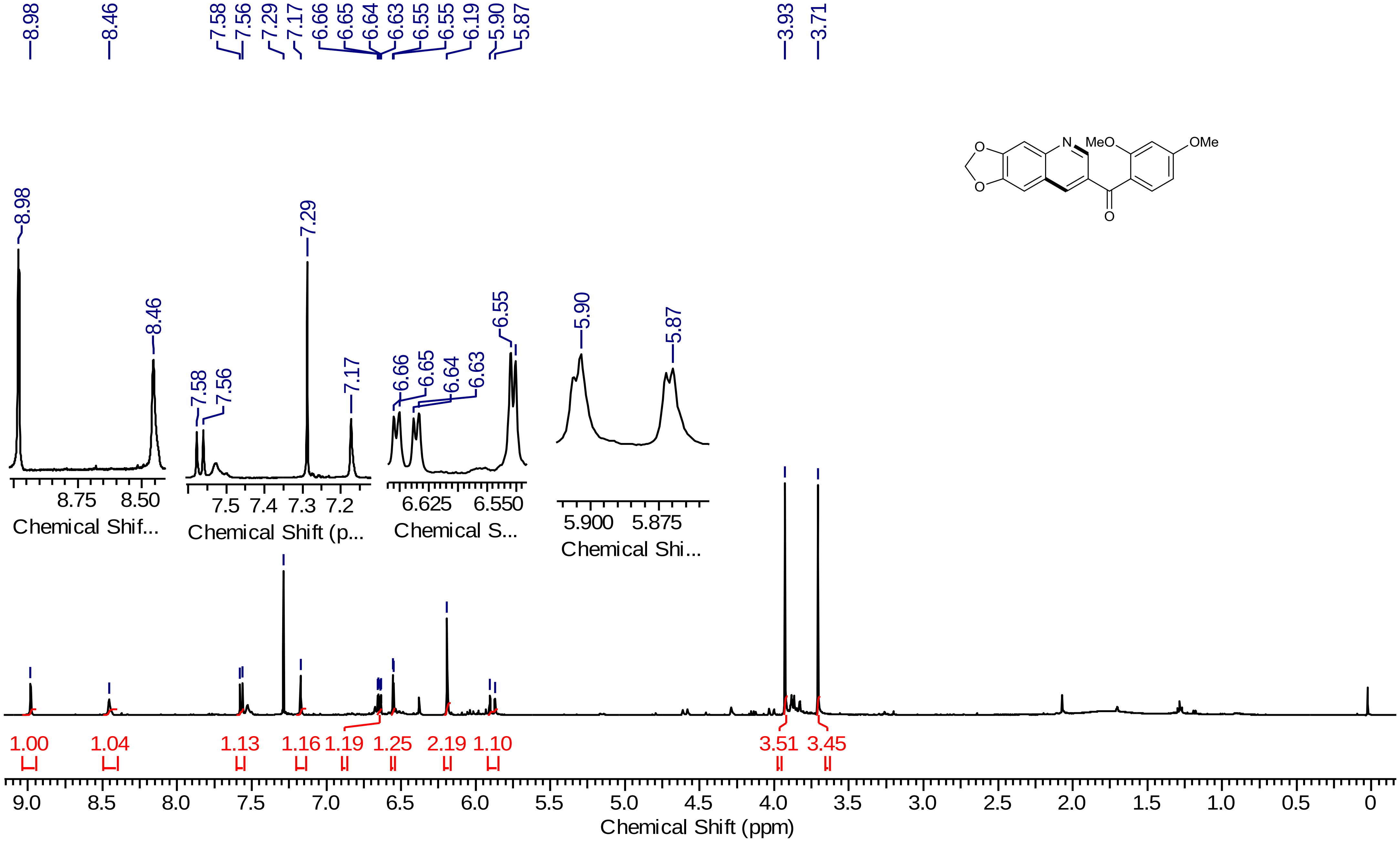

Supplementary Figure 27. ^{1}H NMR of **4n**

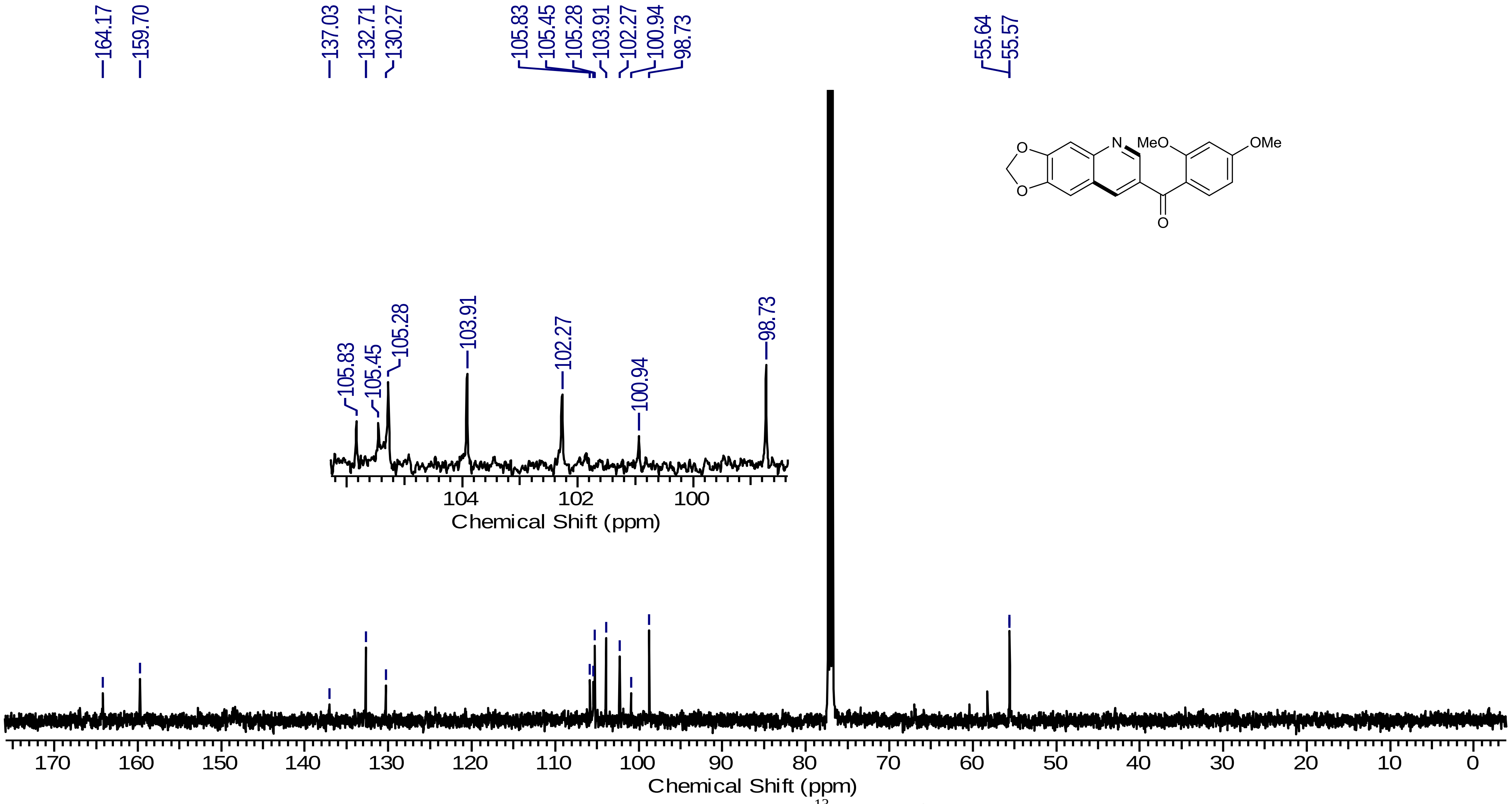

Supplementary Figure 28. ^{13}C NMR of **4n**

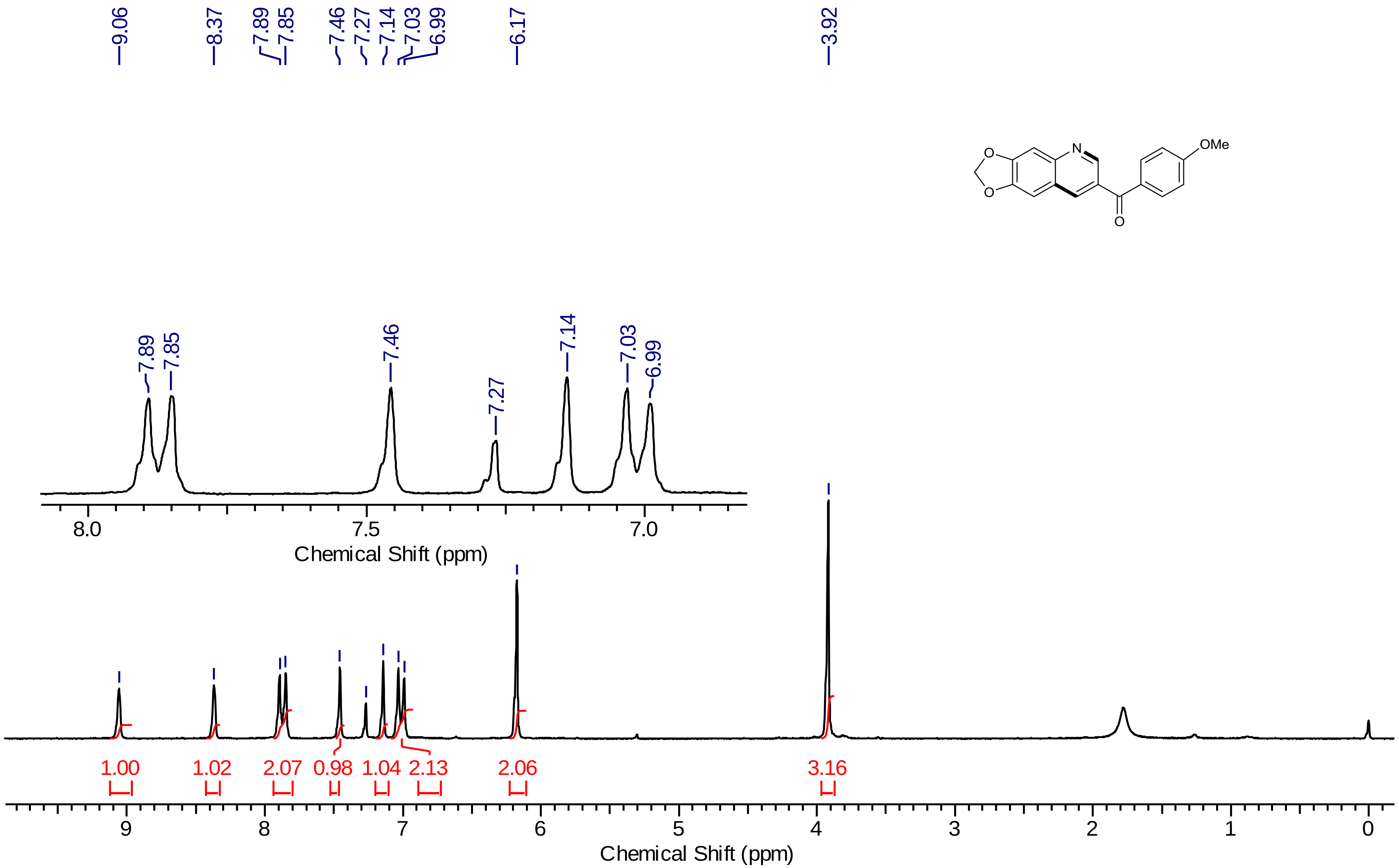

Supplementary Figure 29. ¹H NMR of **4o**

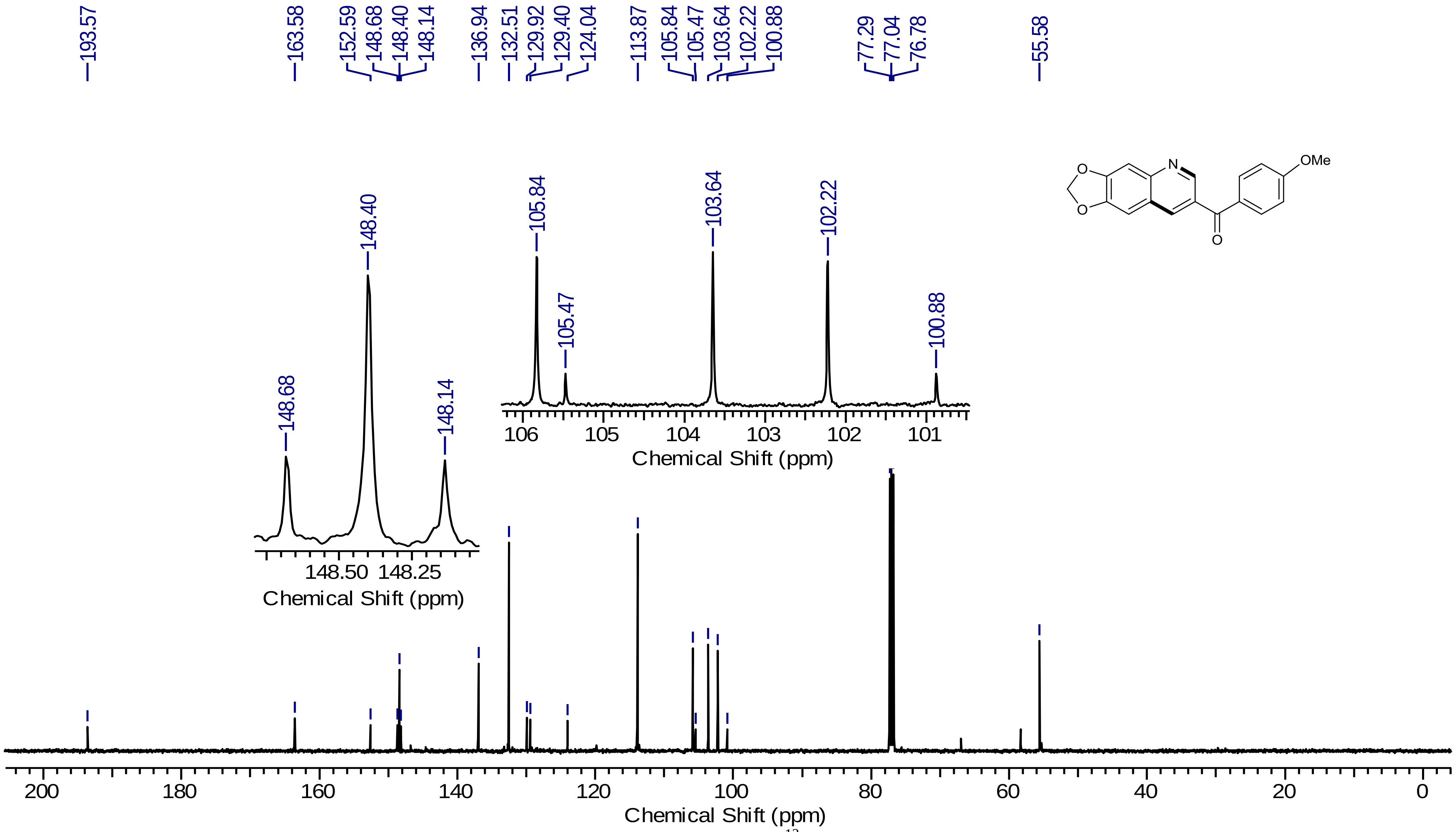

Supplementary Figure 30. ^{13}C NMR of **4o**

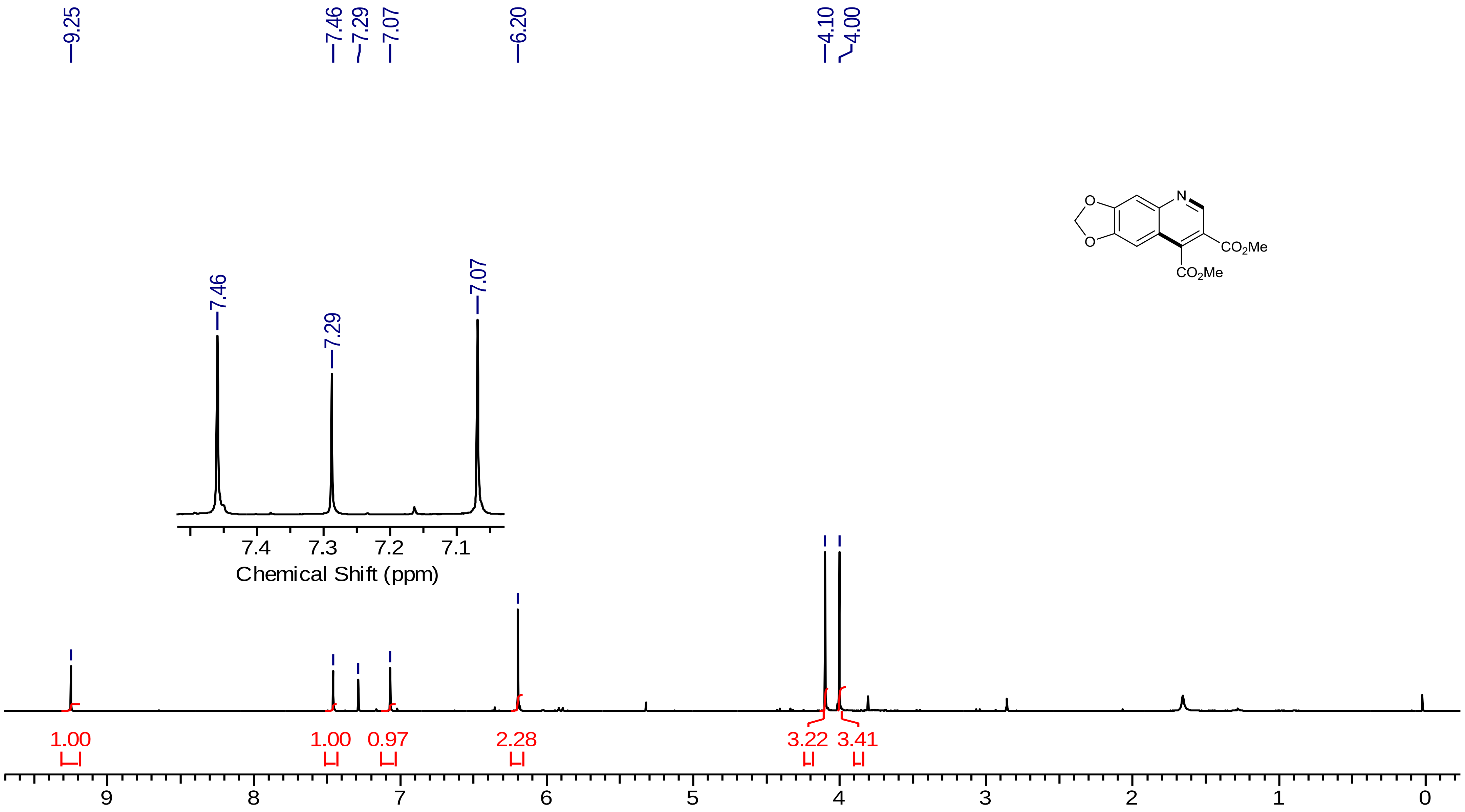

Supplementary Figure 31. ^{1}H NMR of **4p**

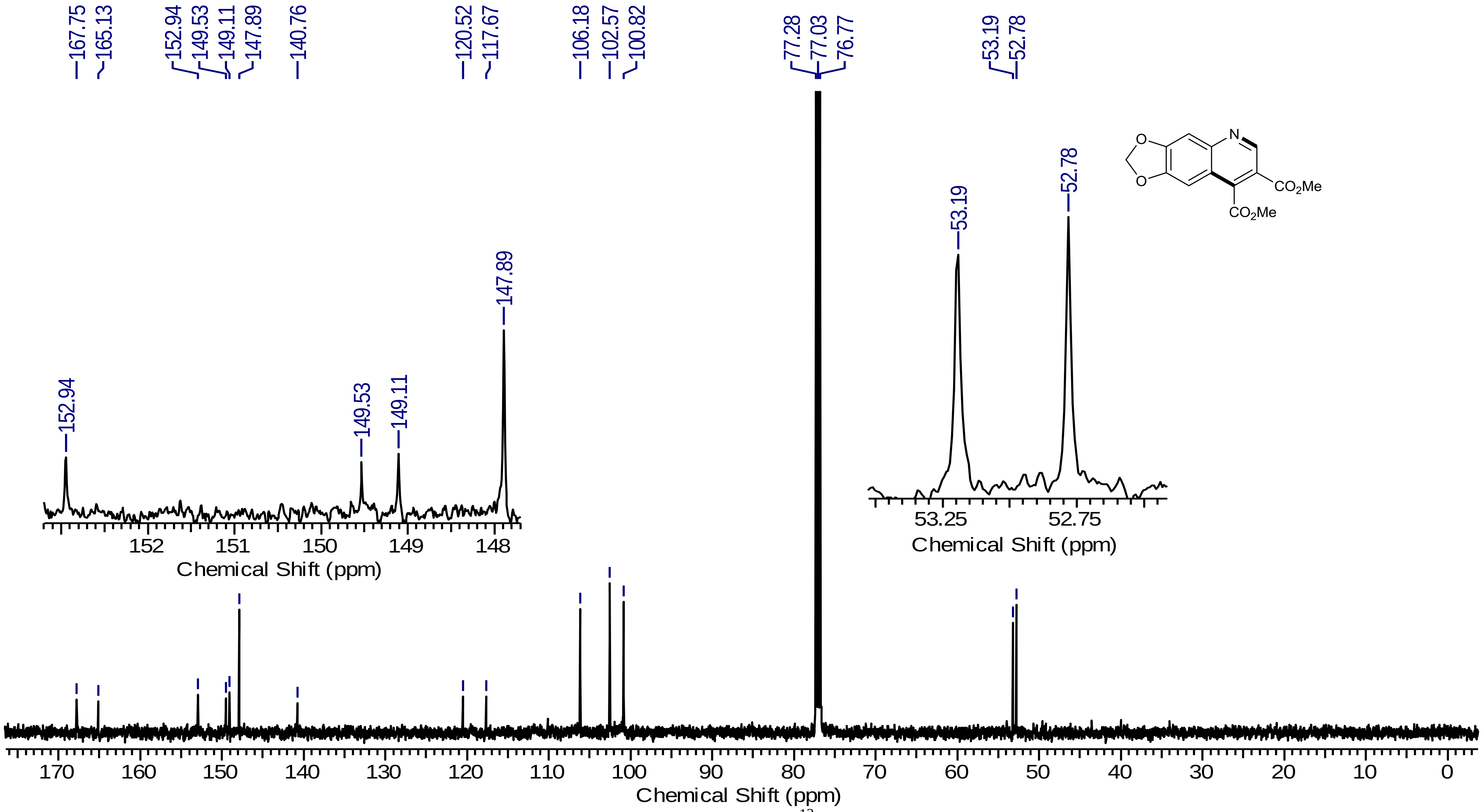

Supplementary Figure 32. ^{13}C NMR of **4p**

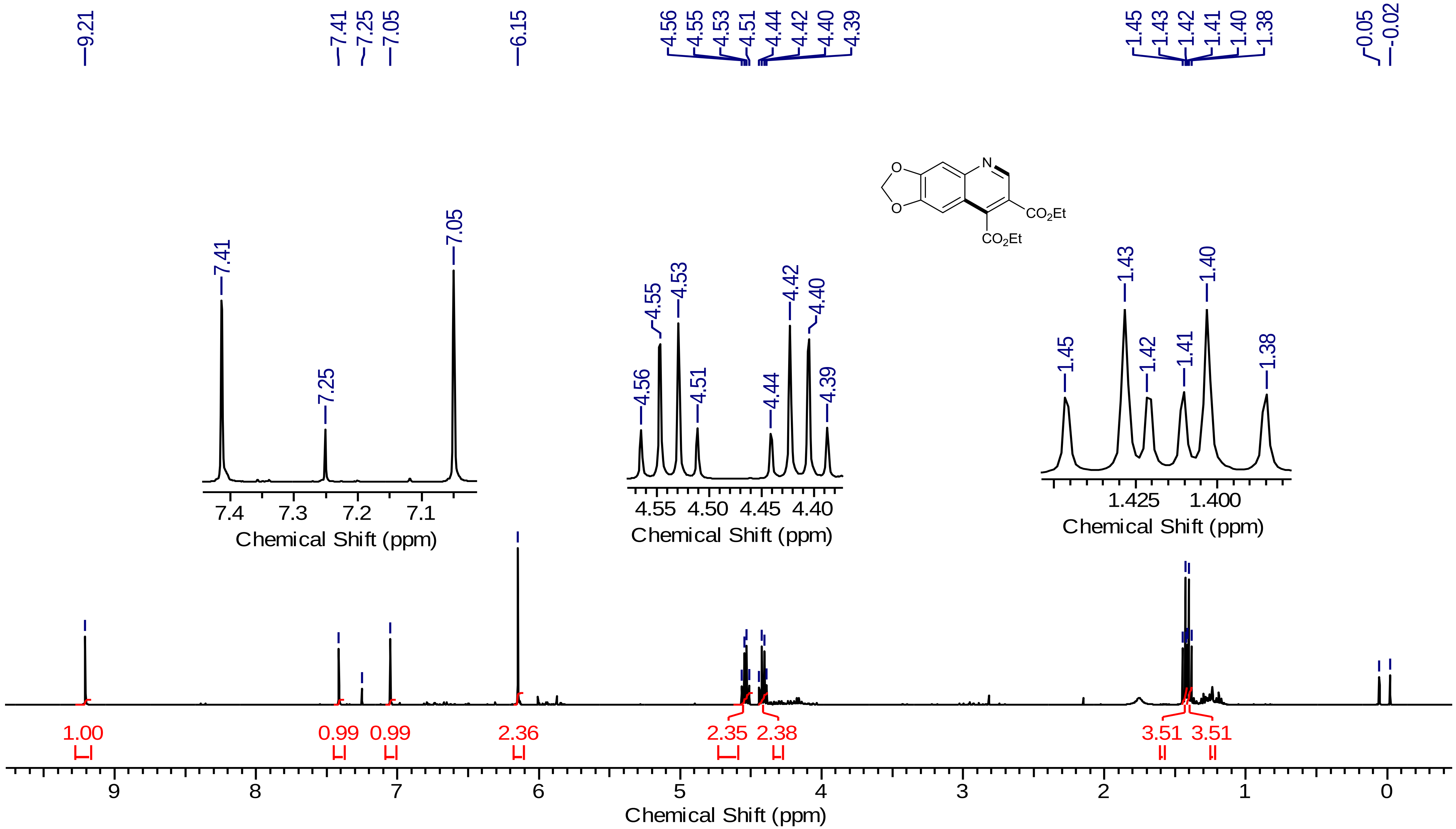

Supplementary Figure 33. ^{1}H NMR of **4q**

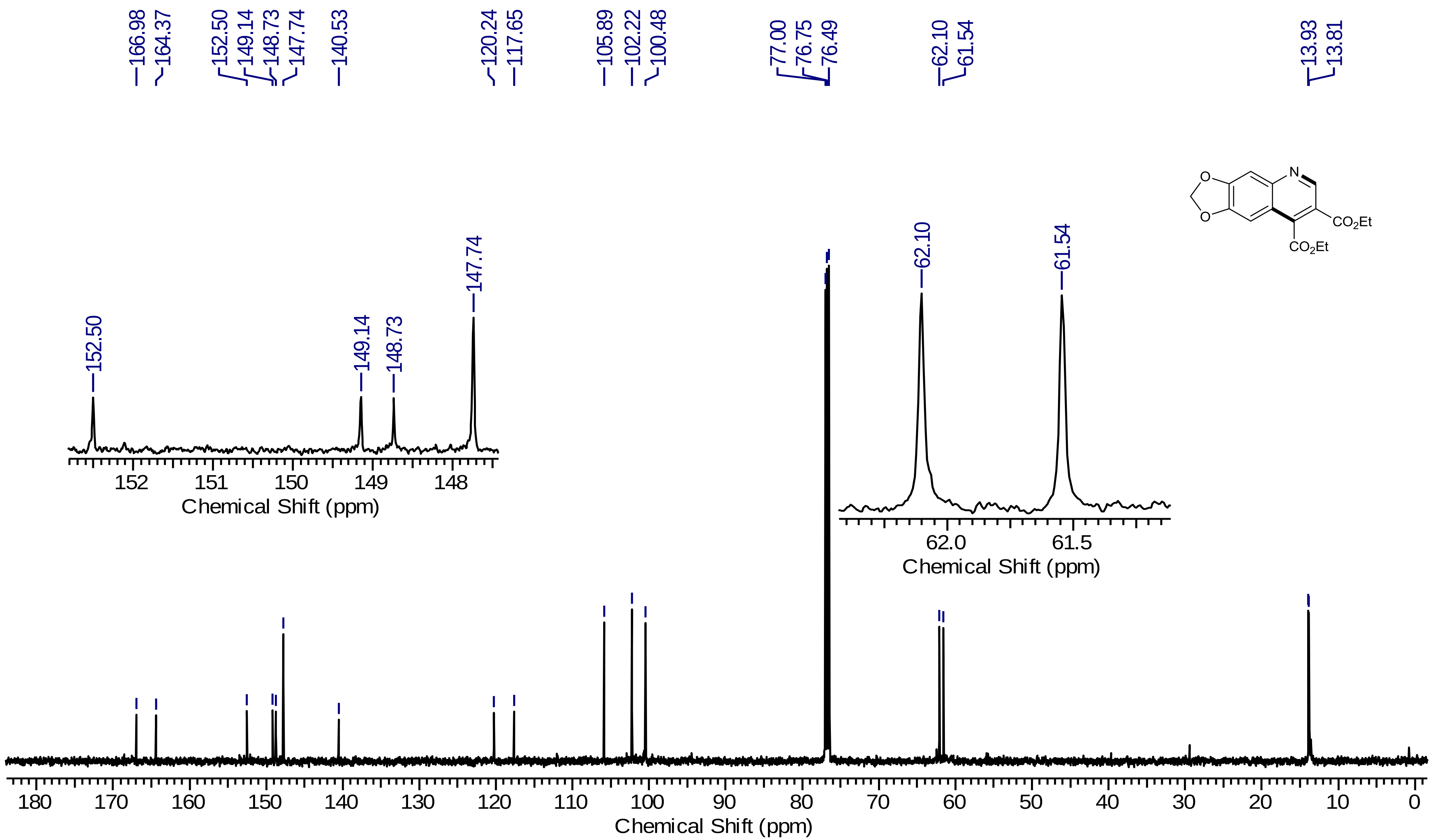

Supplementary Figure 34. ^{13}C NMR of **4q**

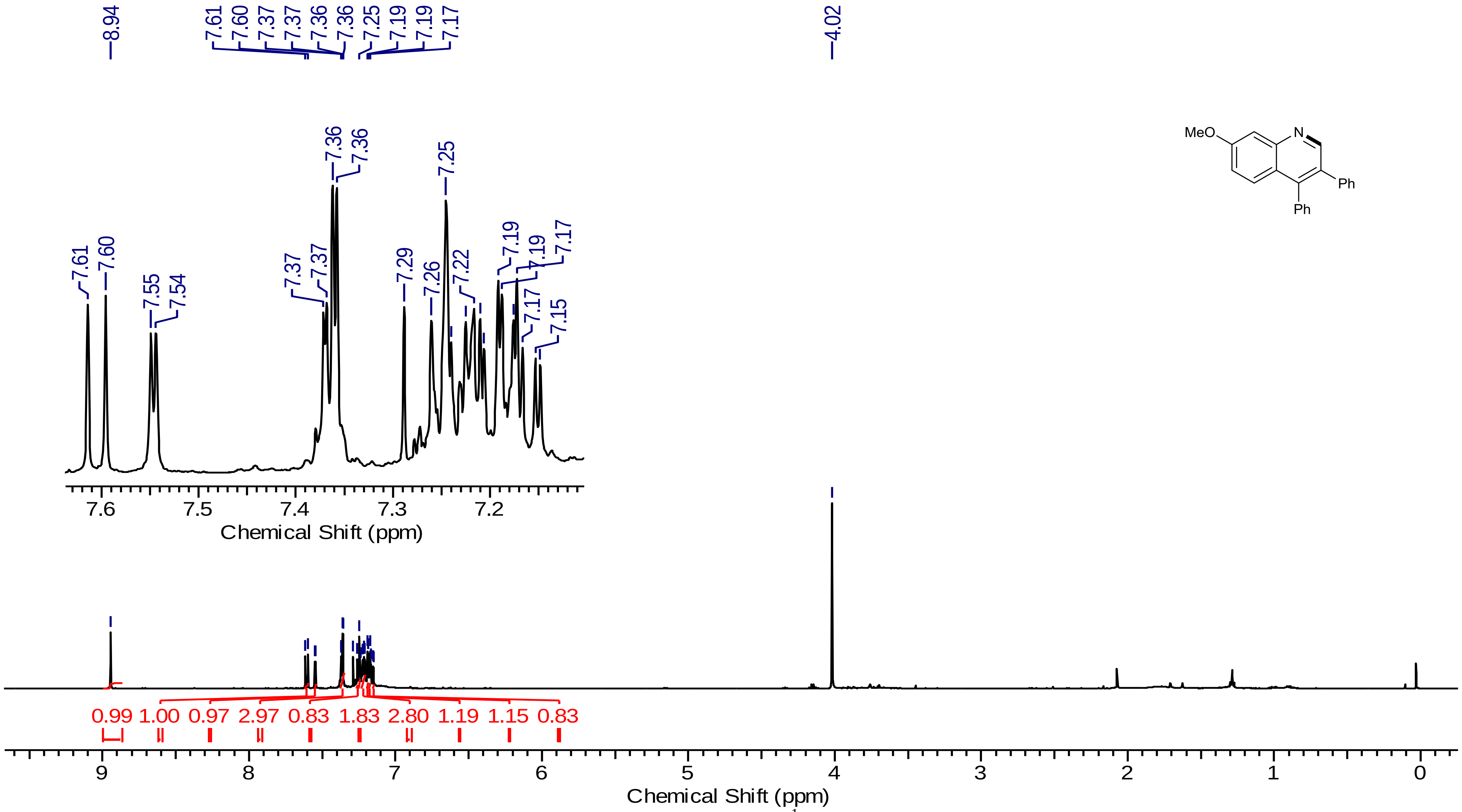

Supplementary Figure 35. ^{1}H NMR of **6a**

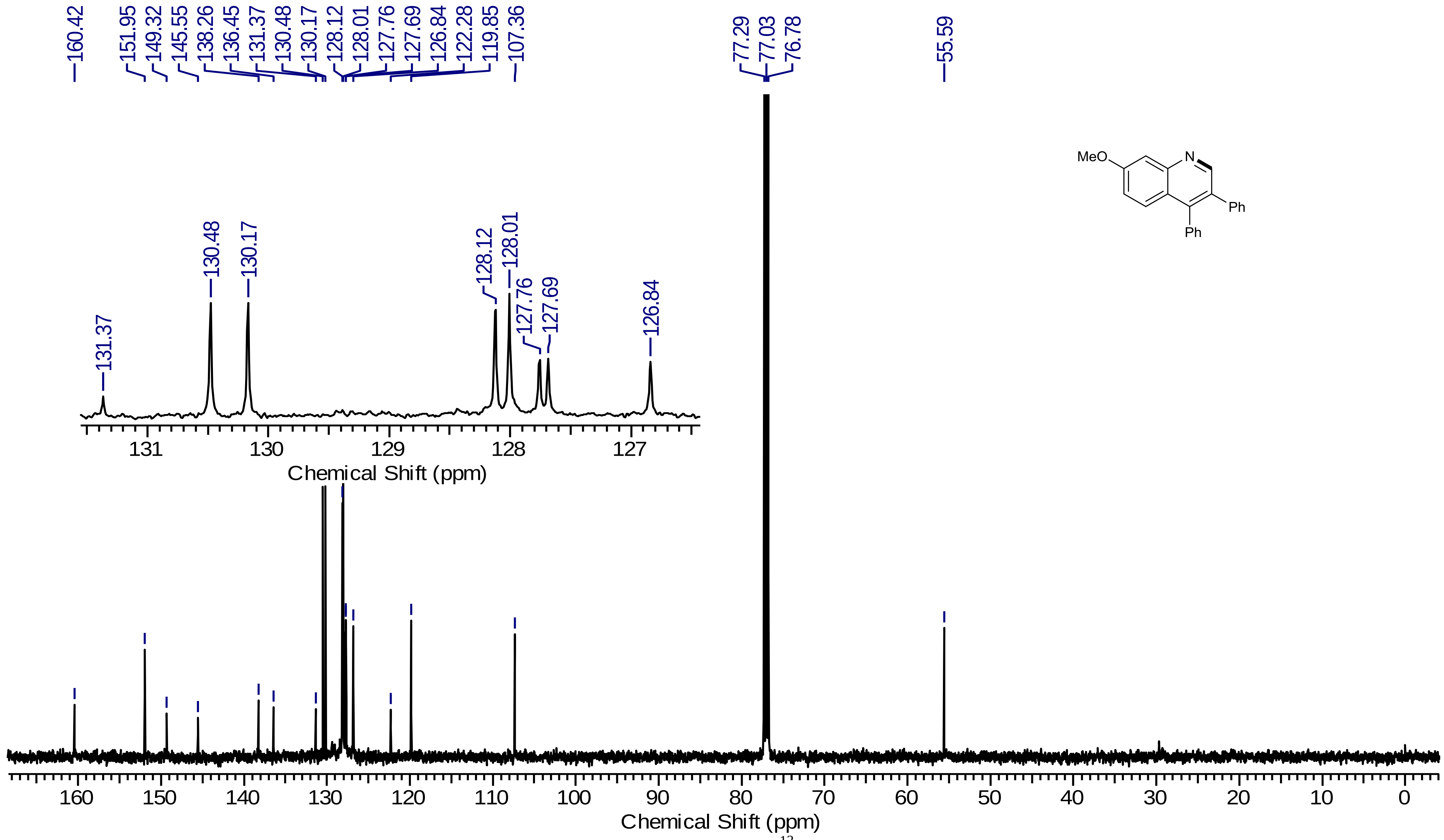

Supplementary Figure 36. ^{13}C NMR of **6a**

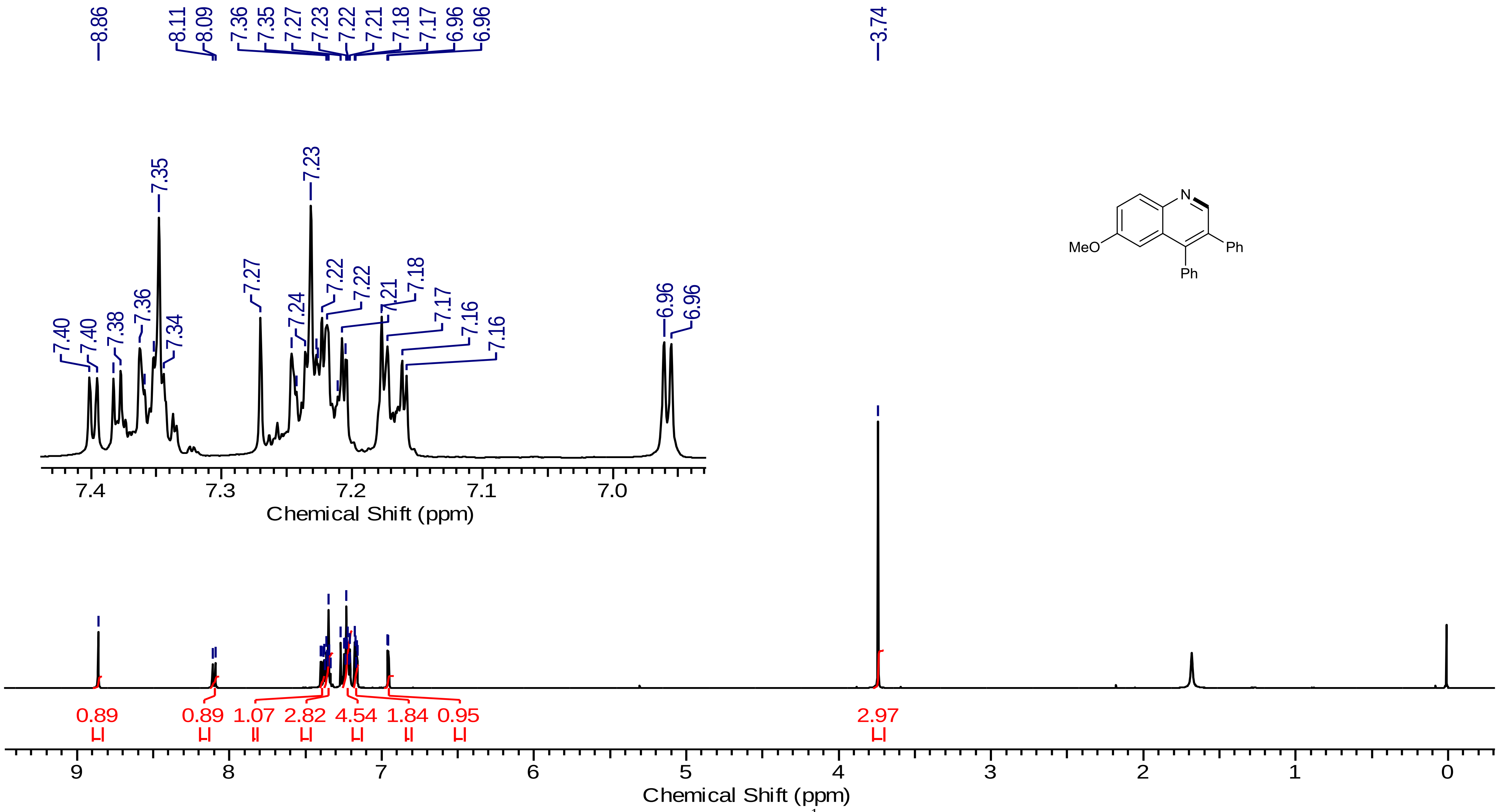

Supplementary Figure 37. ^{1}H NMR of **6b**

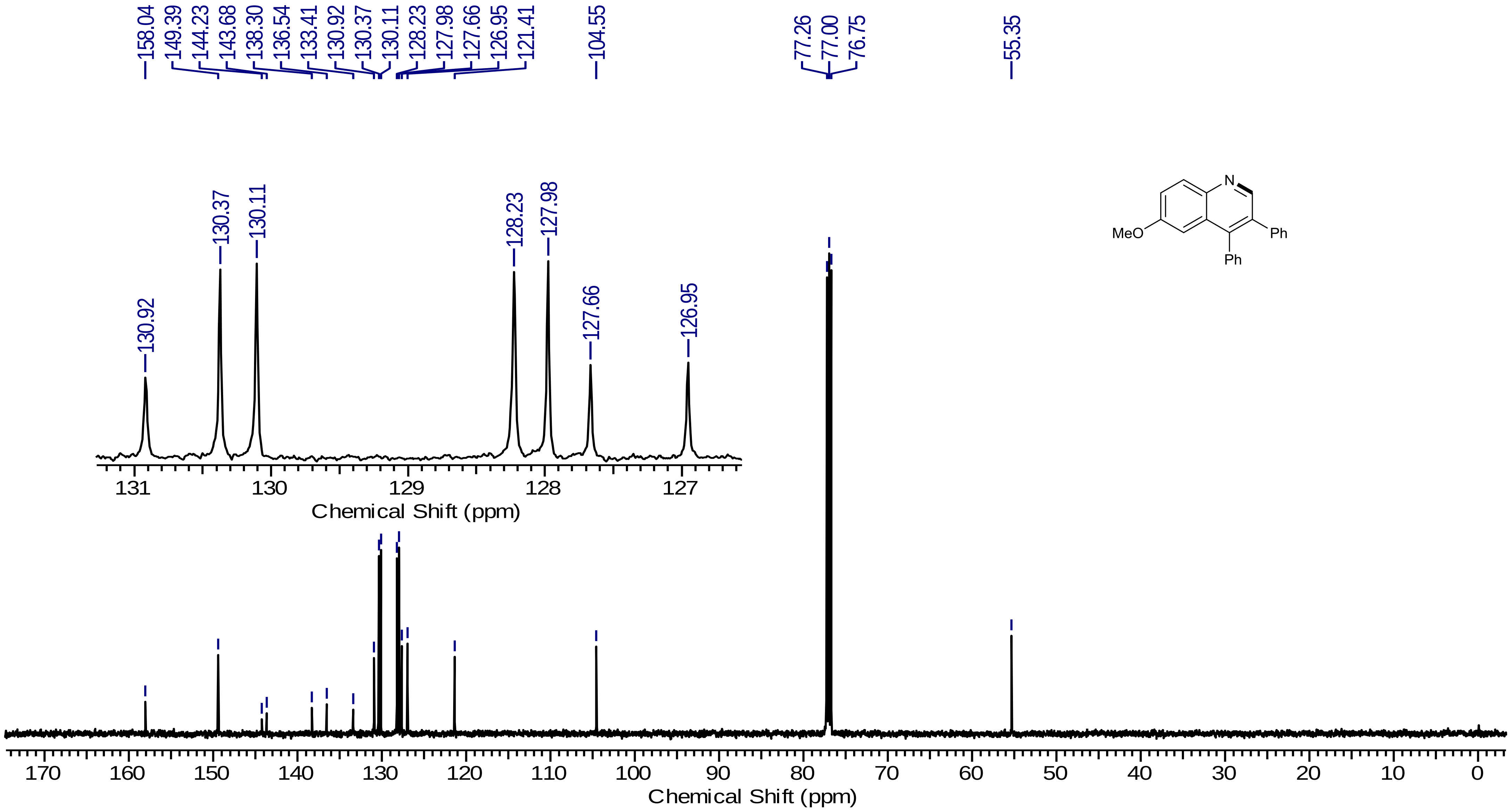

Supplementary Figure 38. ^{13}C NMR of **6b**

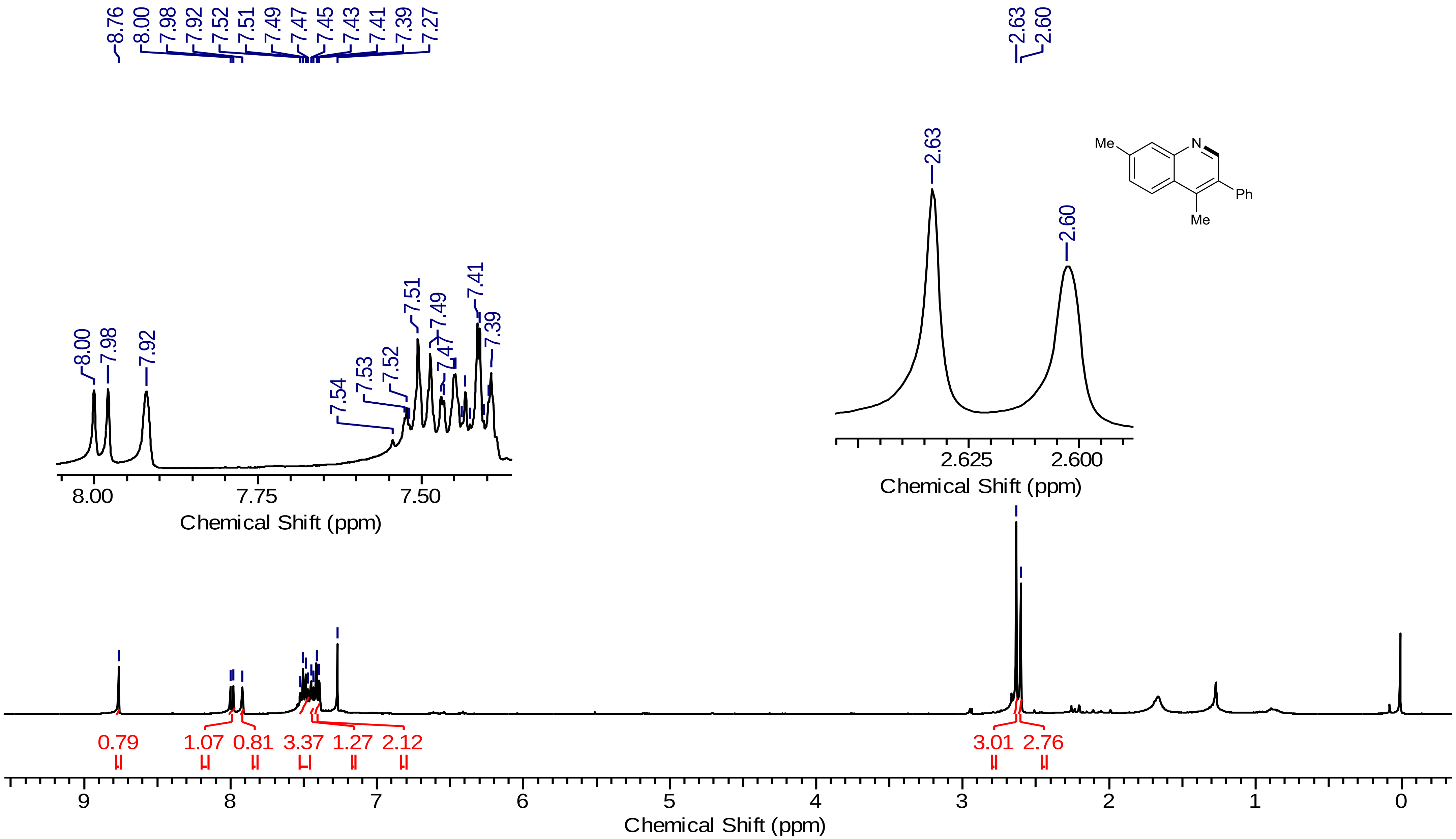

Supplementary Figure 39. ^{1}H NMR of **6c**

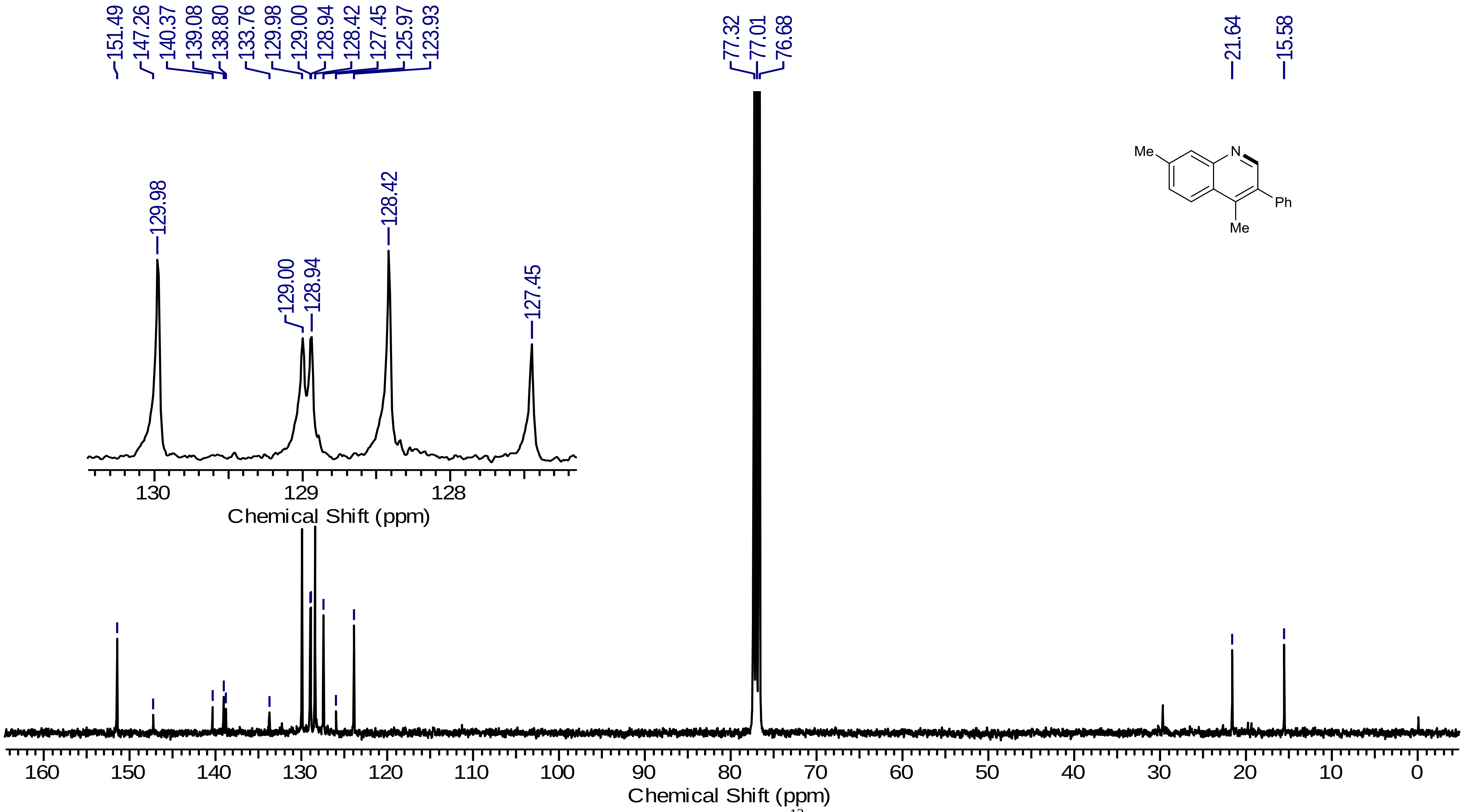

Supplementary Figure 40. ^{13}C NMR of **6c**

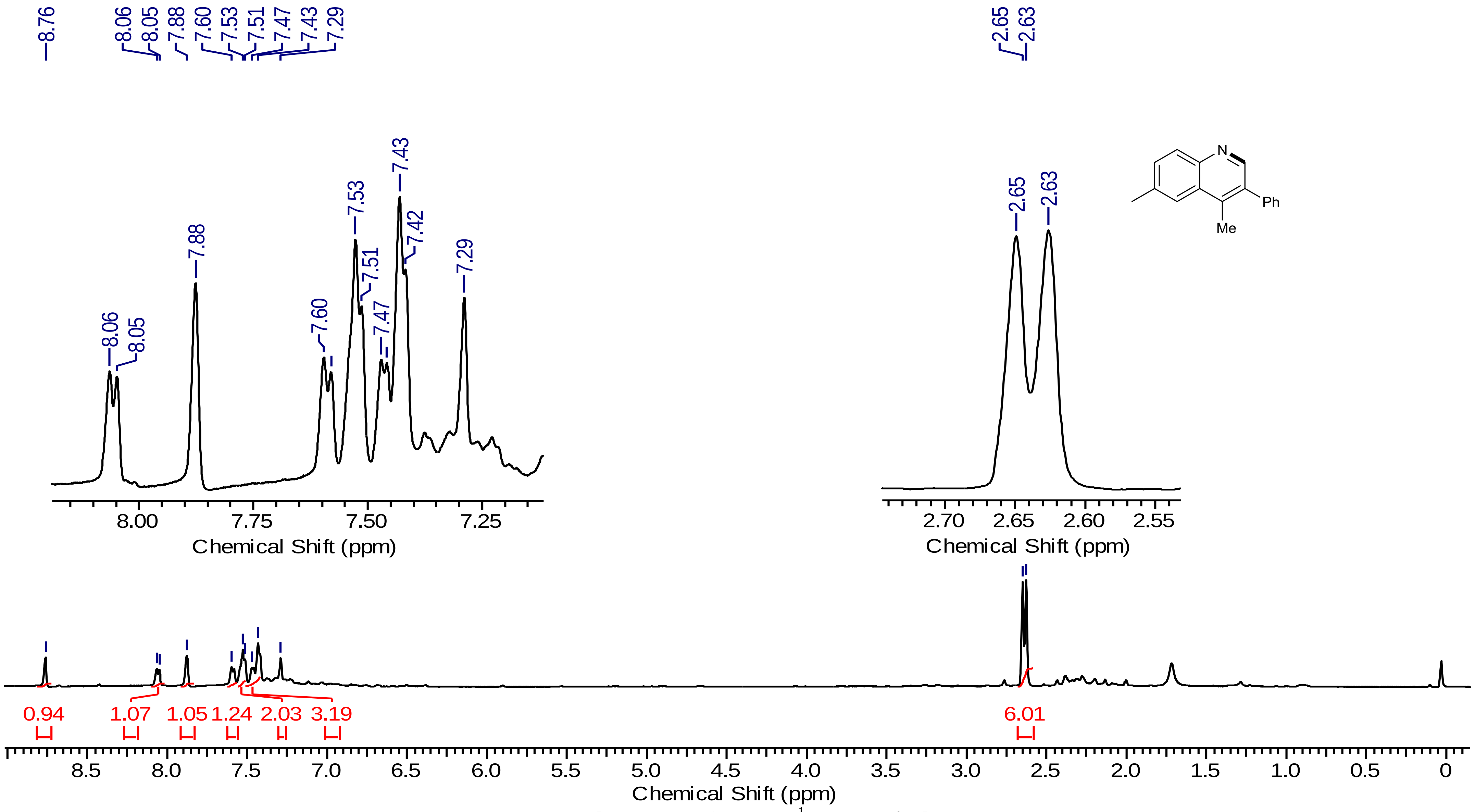

Supplementary Figure 41. ^{1}H NMR of **6d**

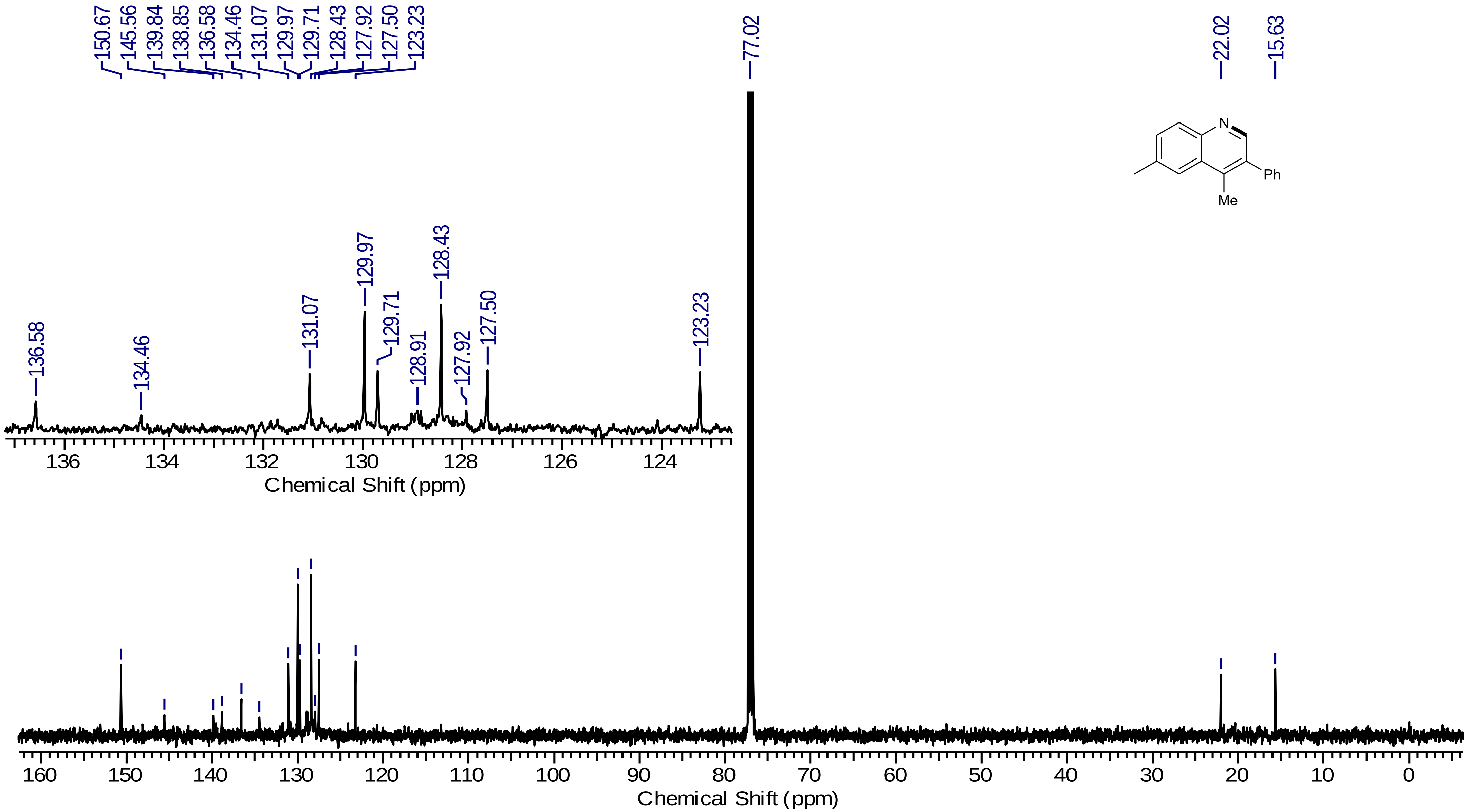

Supplementary Figure 42. ^{13}C NMR of **6d**

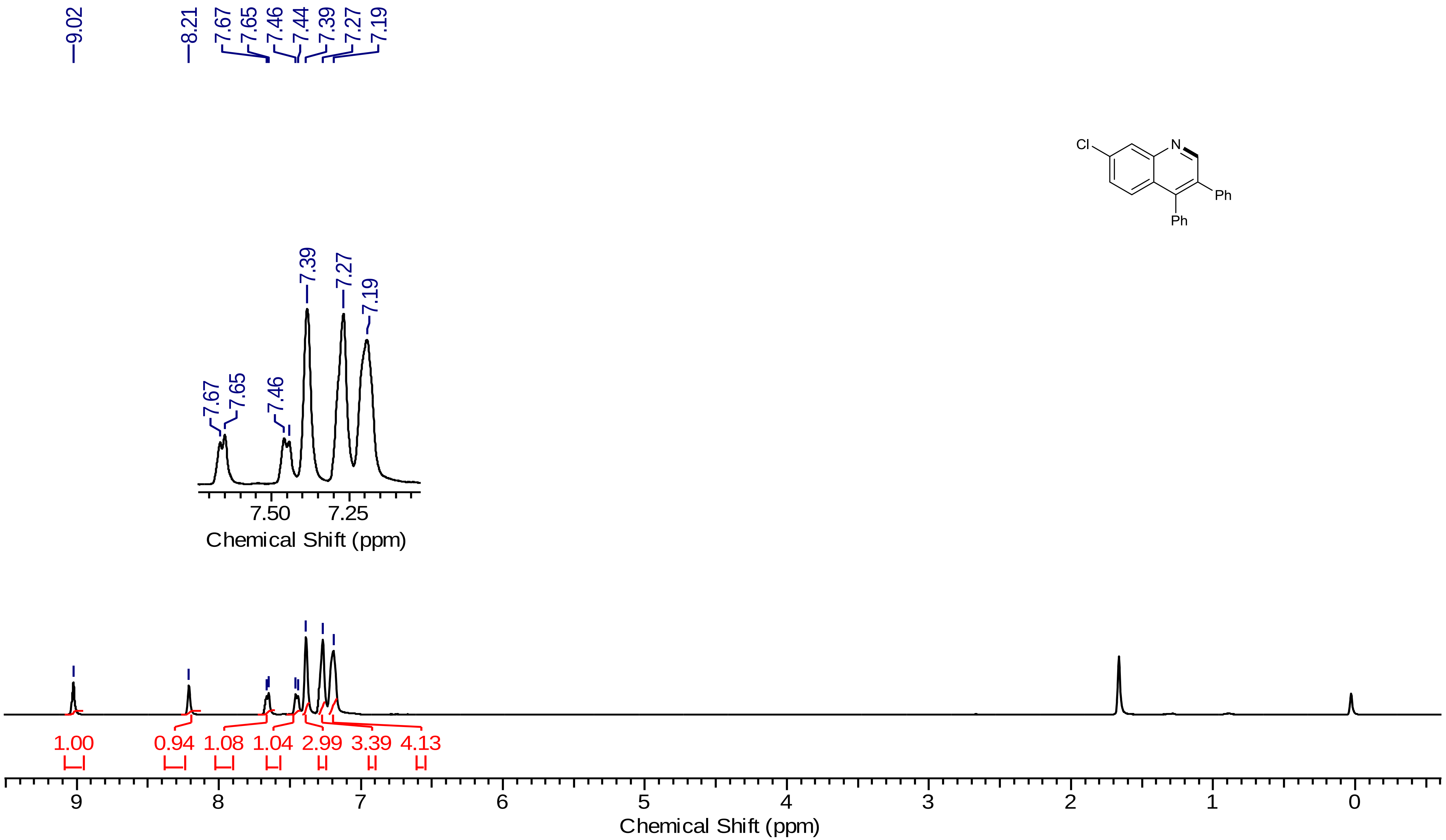

Supplementary Figure 43. ^{1}H NMR of **6e**

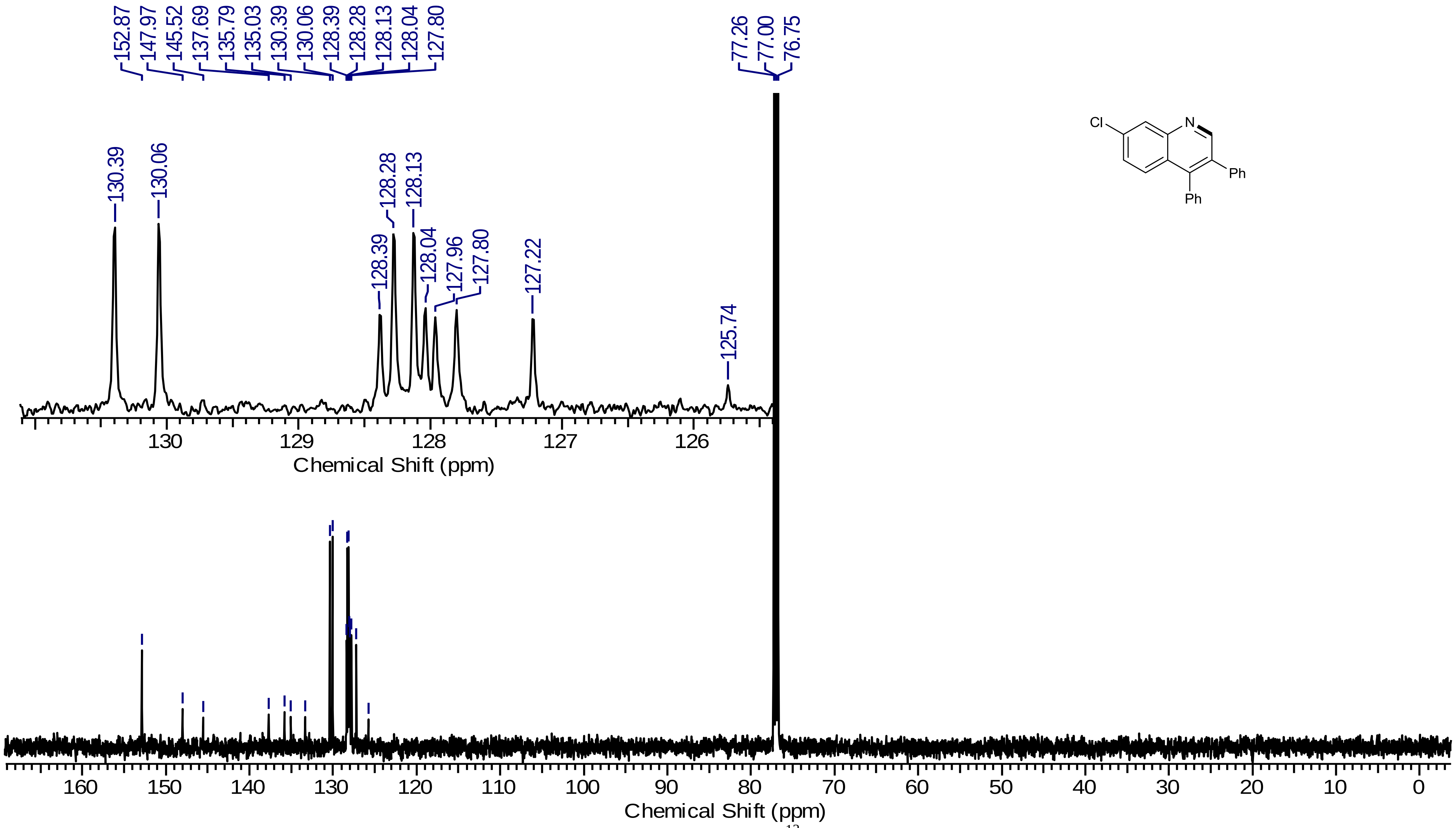

Supplementary Figure 44. ^{13}C NMR of **6e**

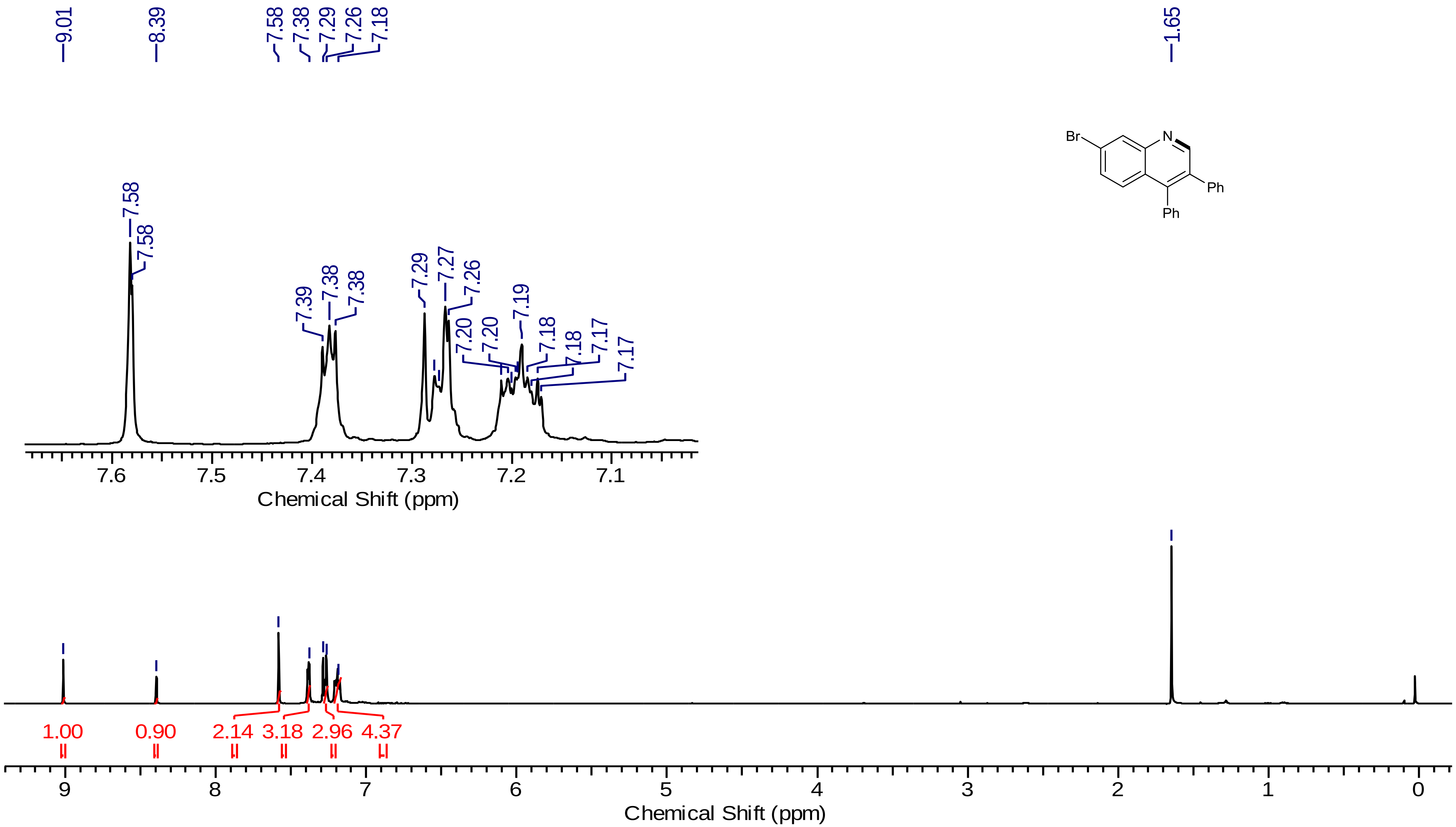

Supplementary Figure 45. ^{1}H NMR of **6f**

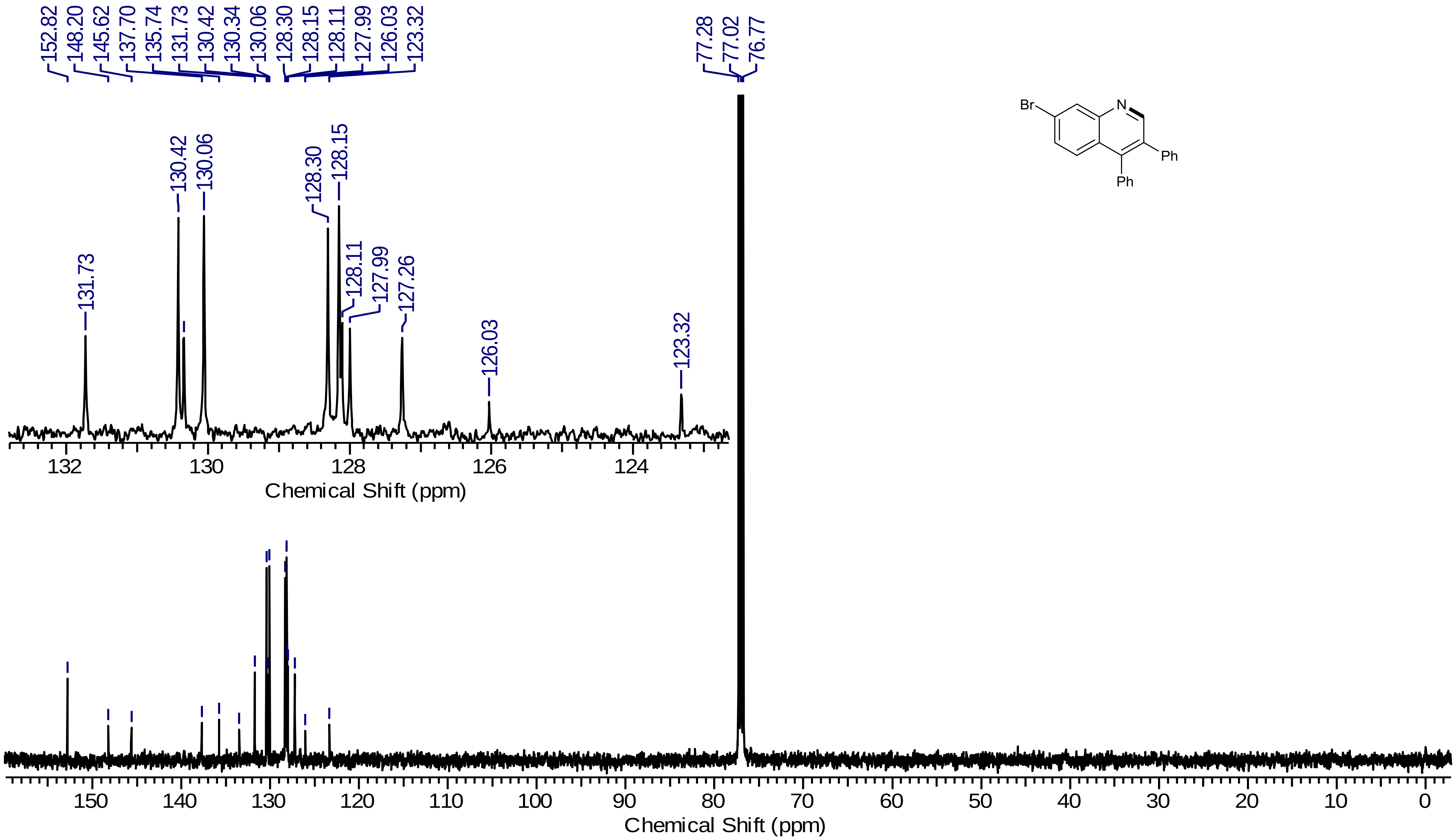

Supplementary Figure 46. ^{13}C NMR of **6f**

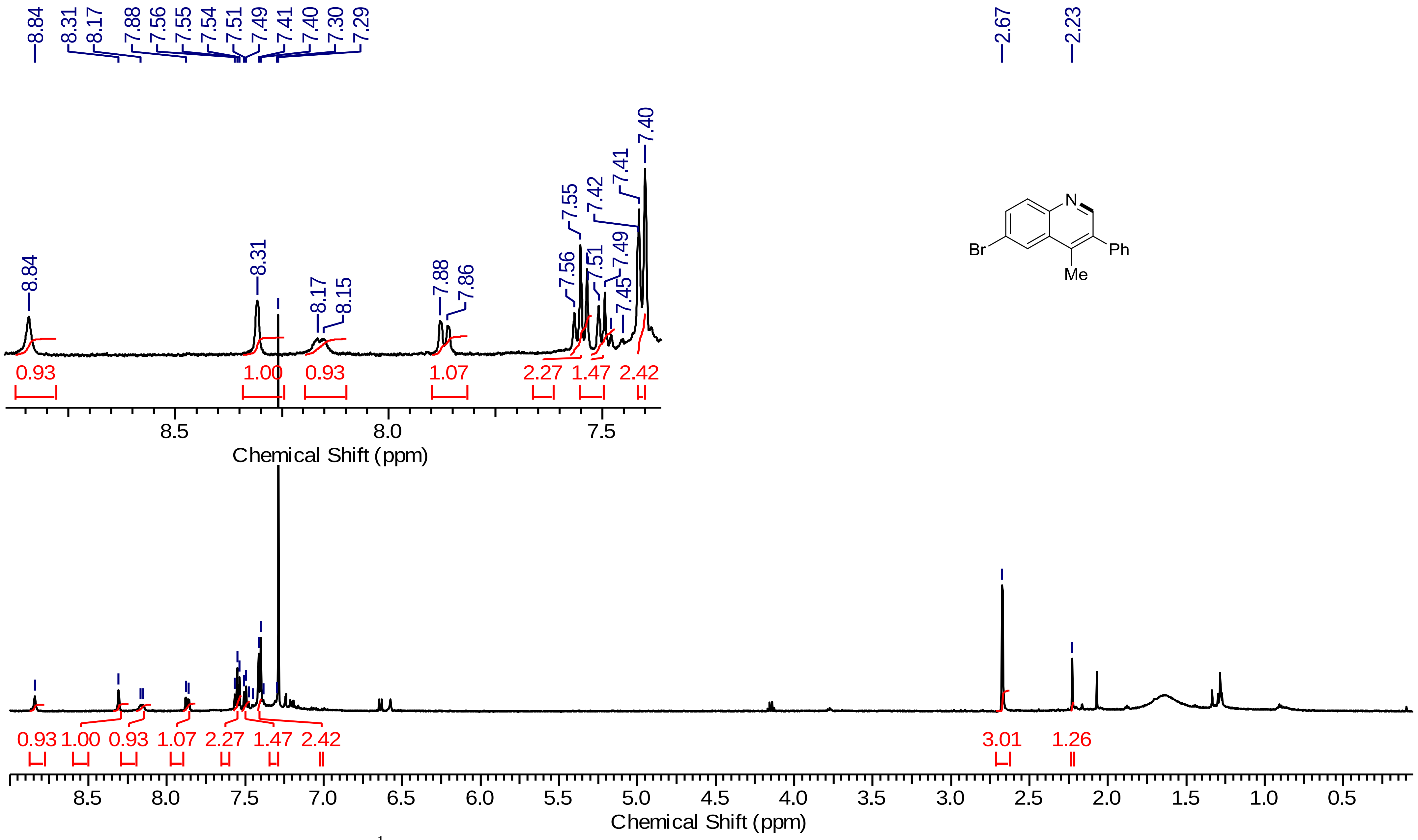

Supplementary Figure 47. ^{1}H NMR of **6g** (product identified from the reaction mixture which contains unreacted starting material 5g)

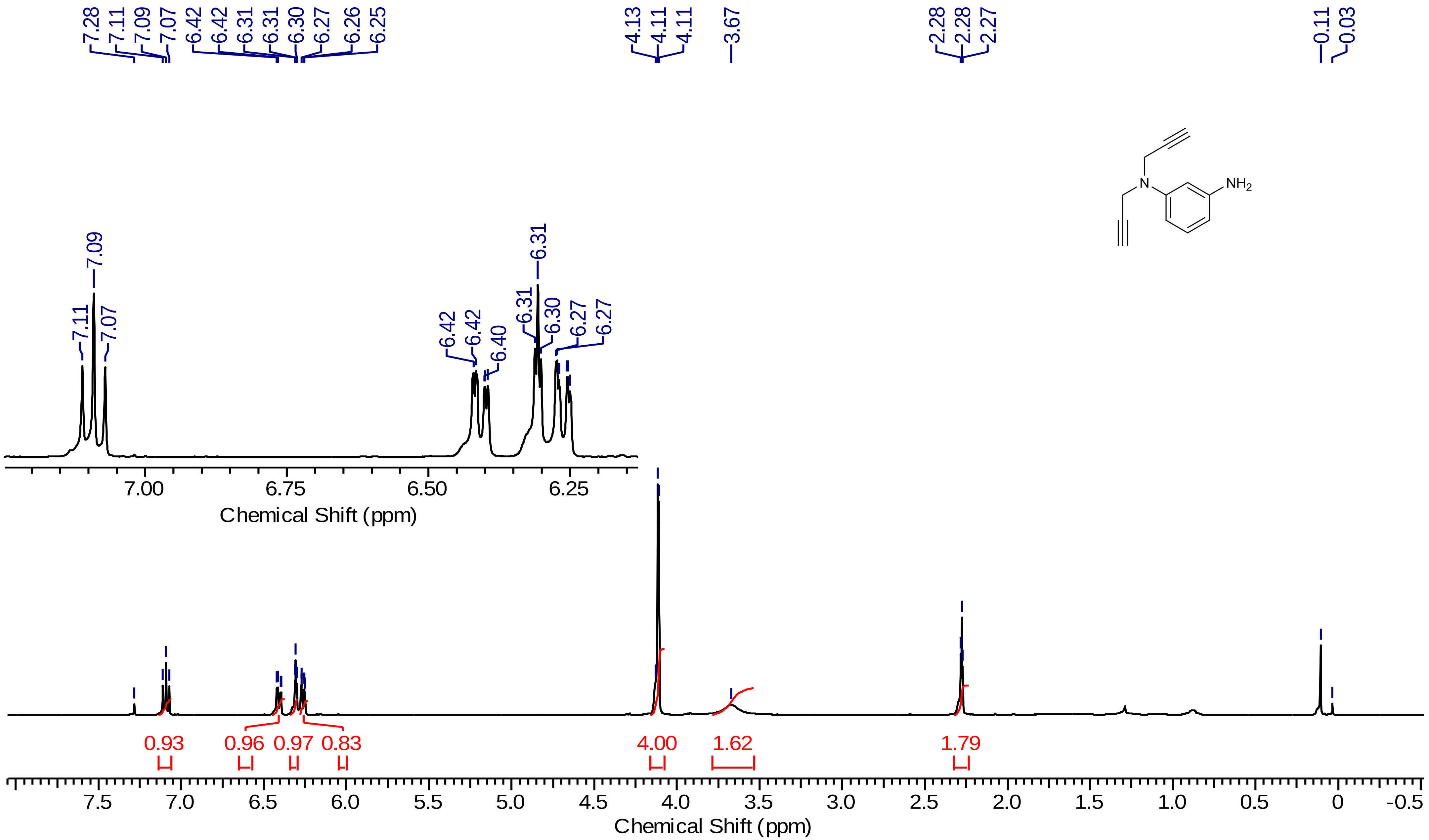

Supplementary Figure 48. ^{1}H NMR of **1h**

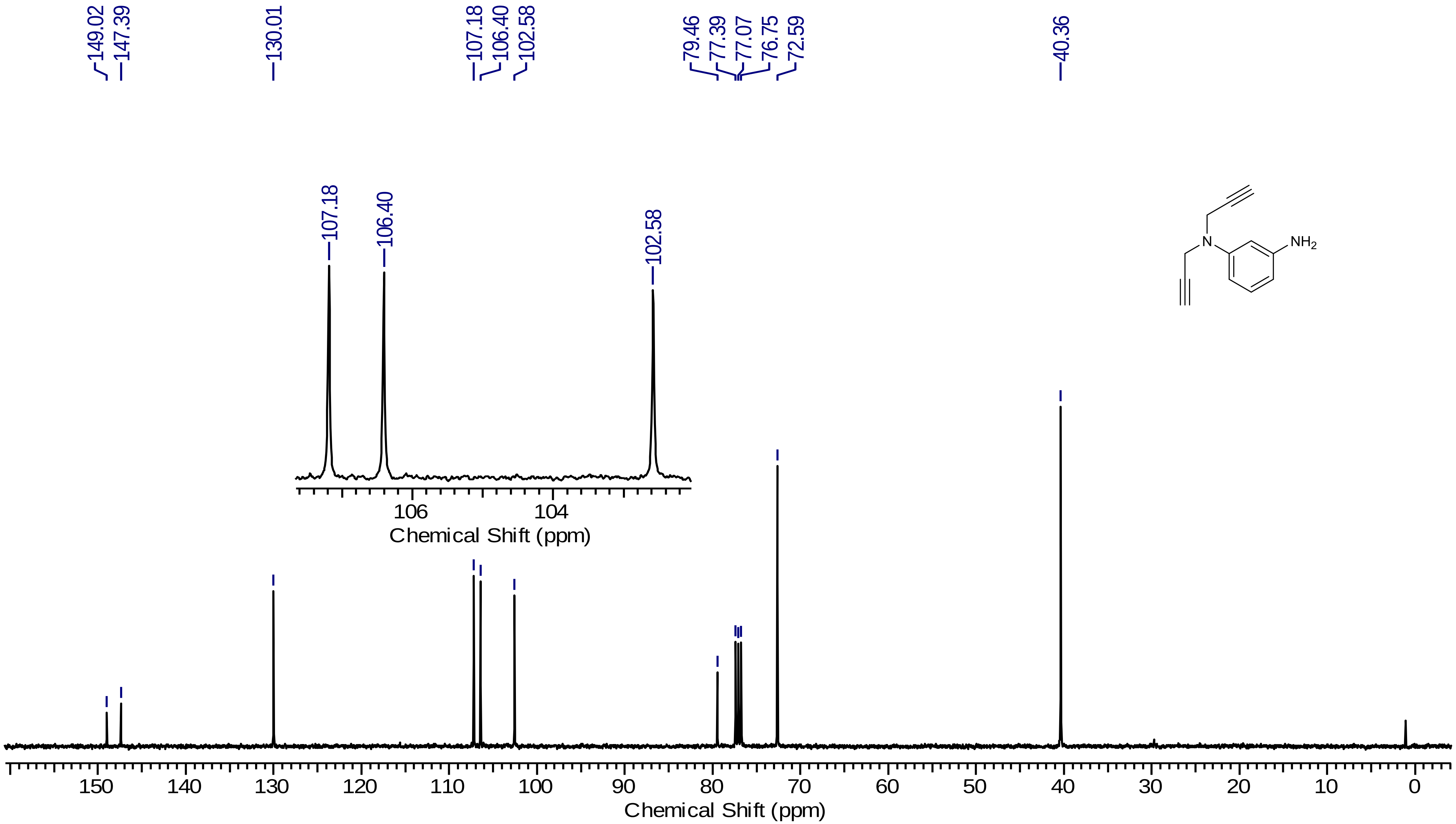

Supplementary Figure 49. ^{13}C NMR of **1h**

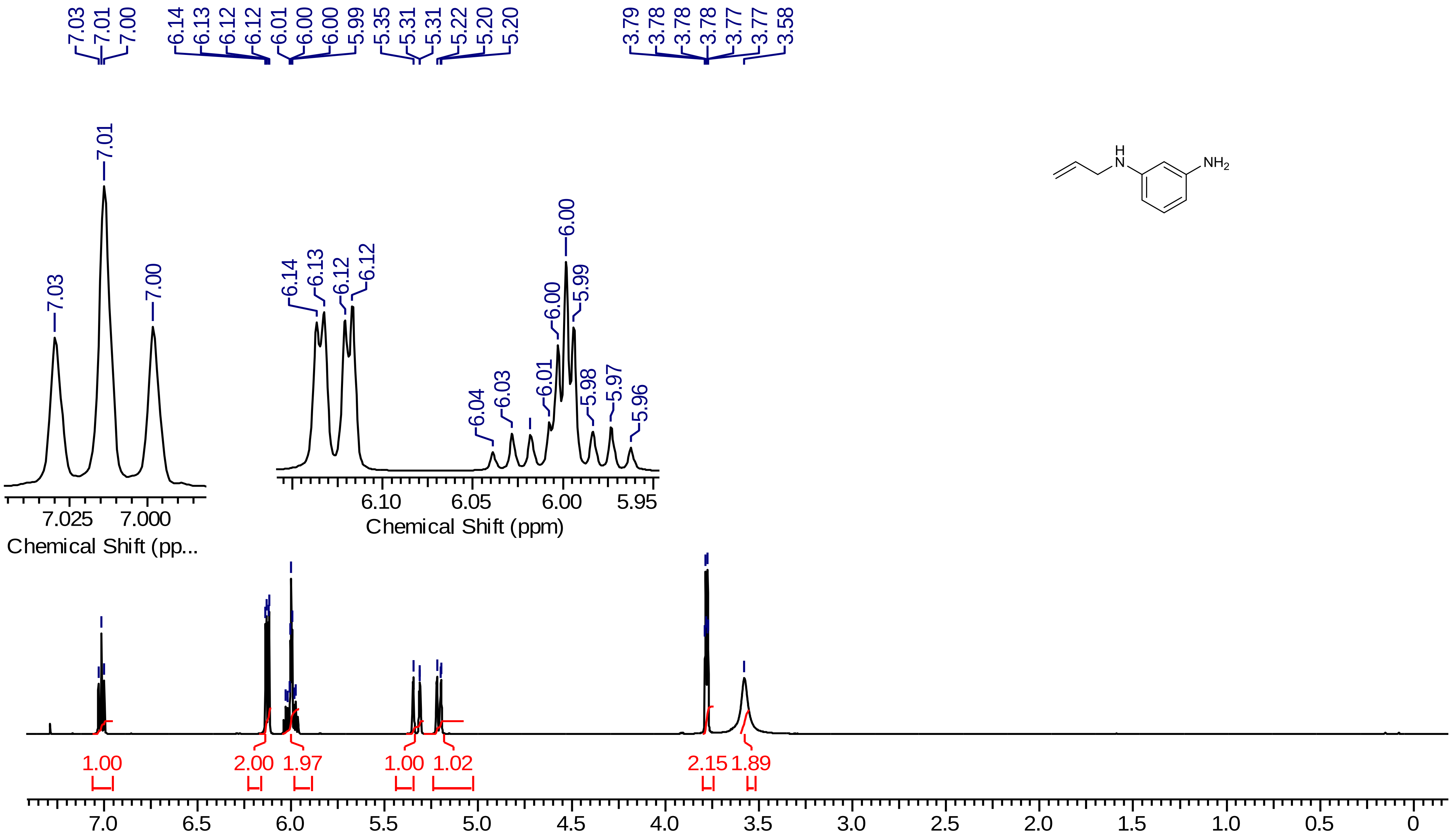

Supplementary Figure 50. ^{1}H NMR of **1i**

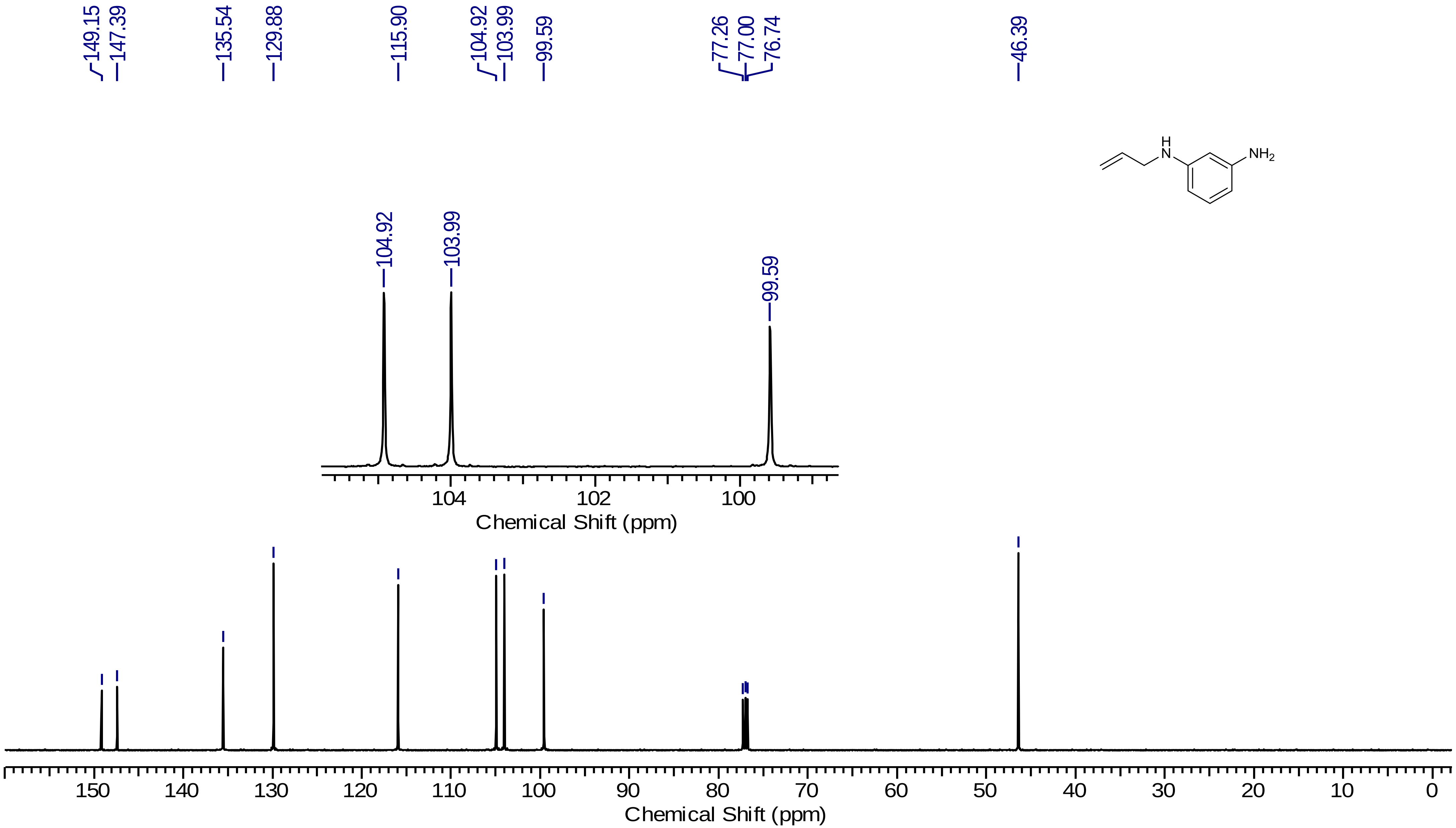

Supplementary Figure 51. ^{13}C NMR of **1i**

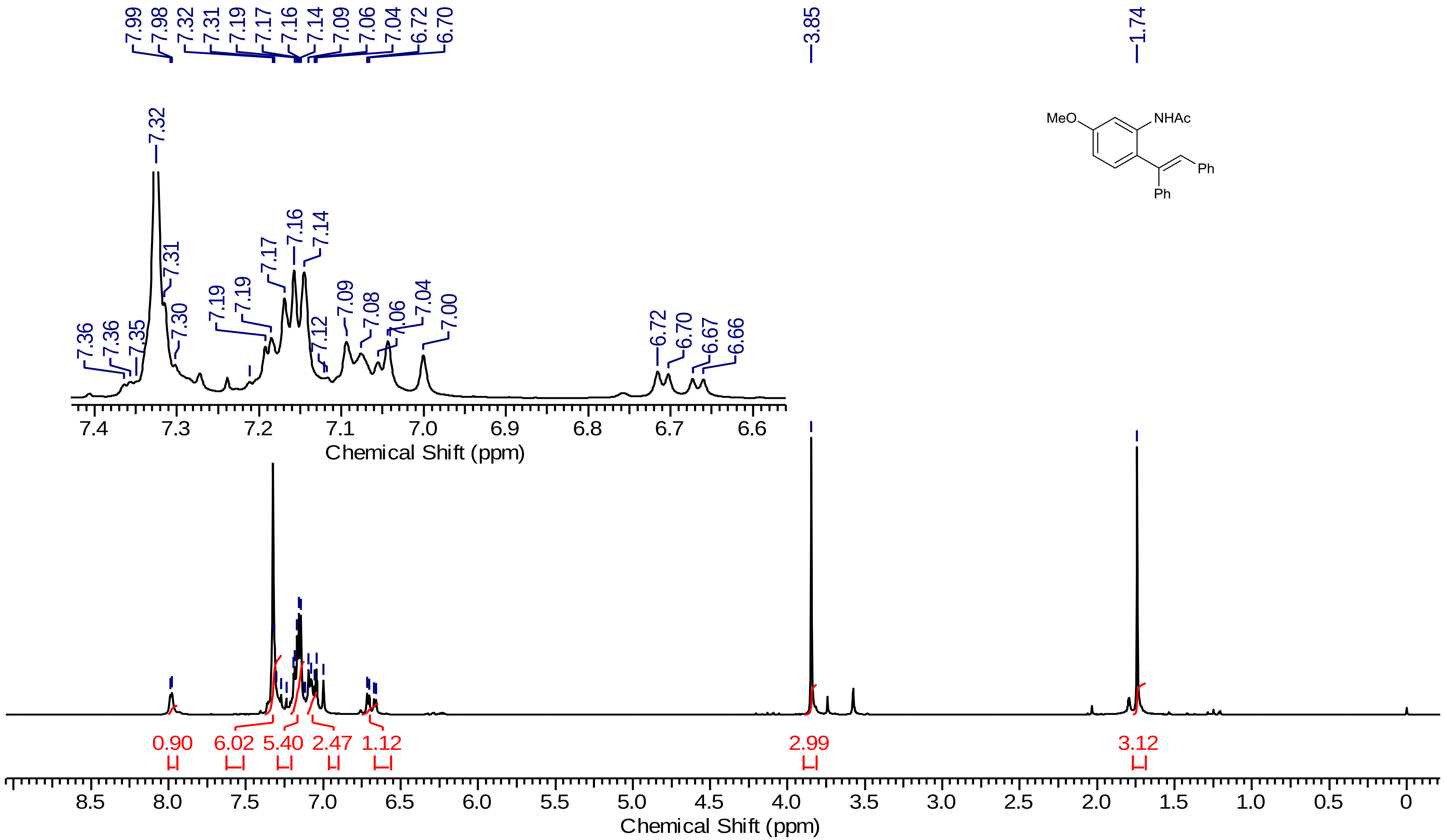

Supplementary Figure 52. ^{1}H NMR of **5a’**

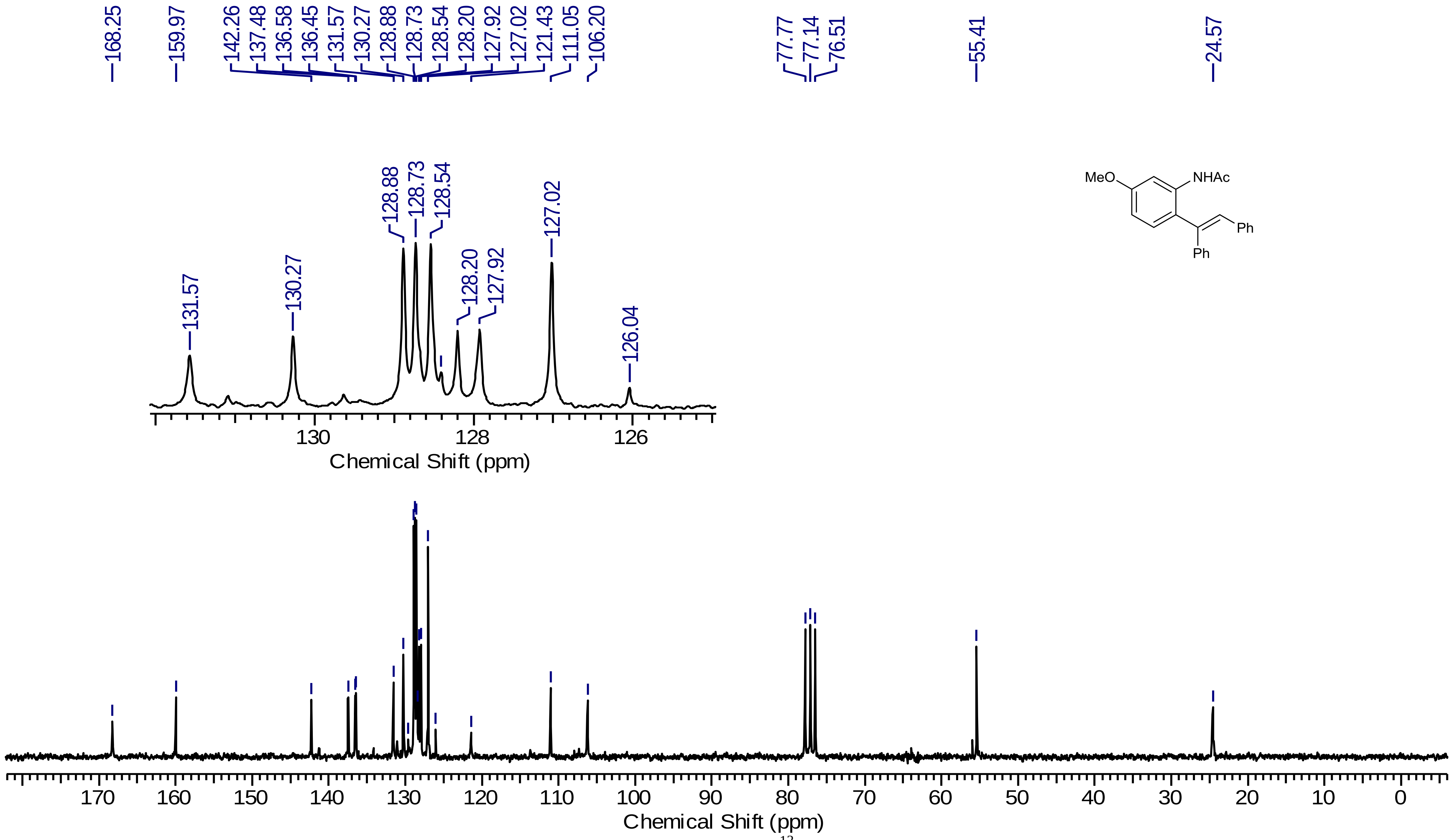

Supplementary Figure 53. ^{13}C NMR of **5a’**

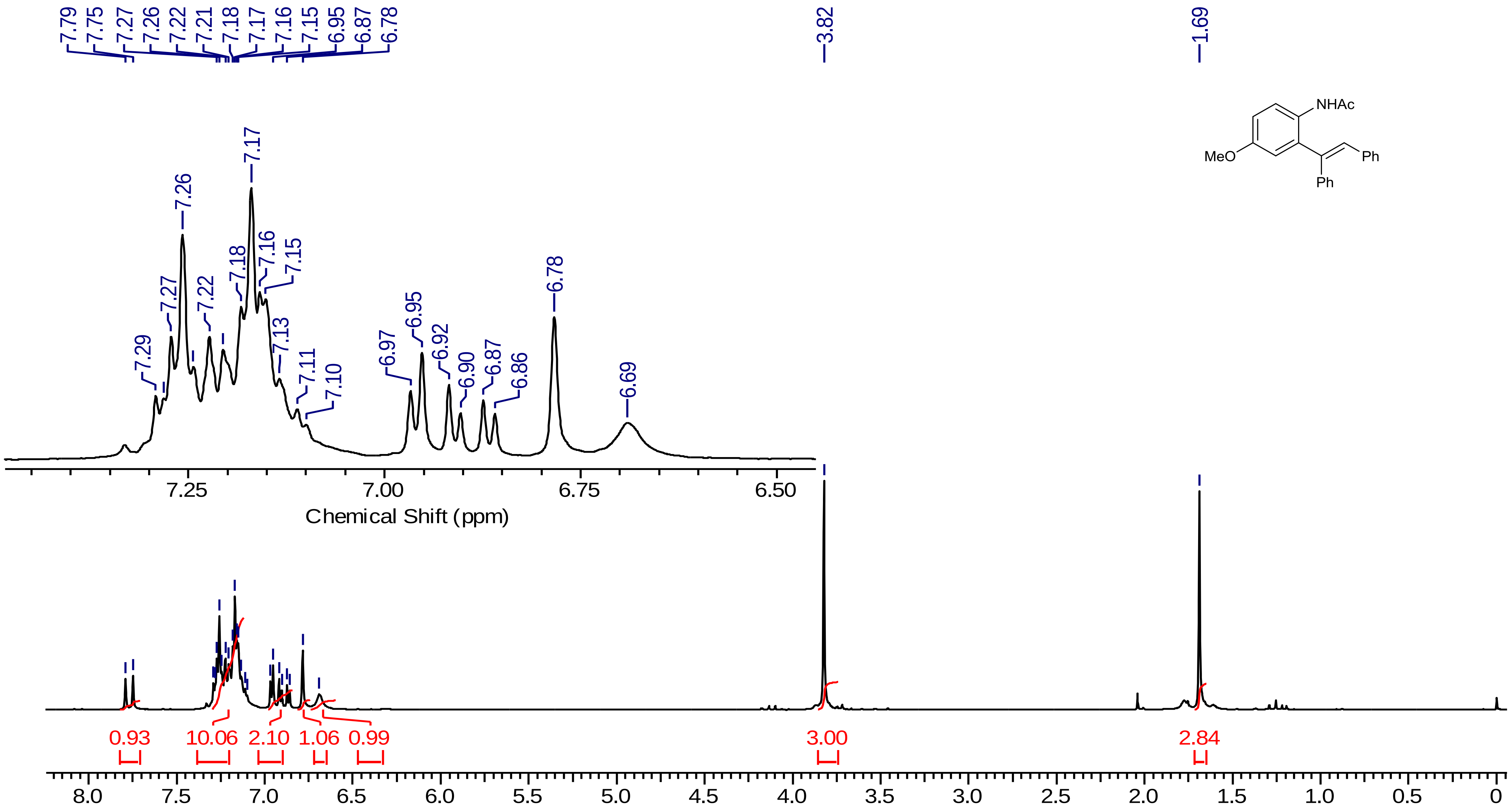

Supplementary Figure 54. ^{1}H NMR of **5b’**

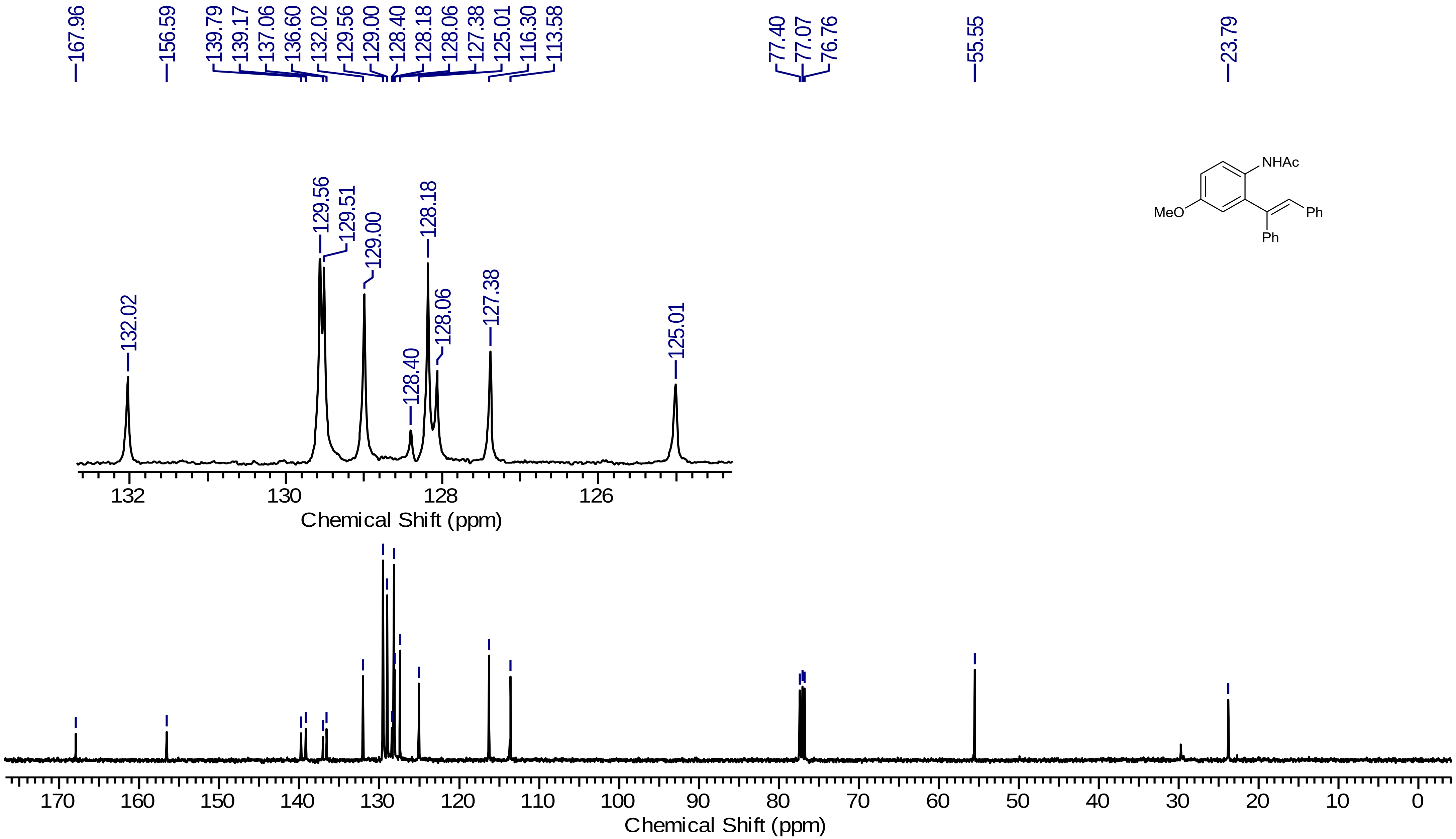

Supplementary Figure 55. ^{13}C NMR of **5b'**

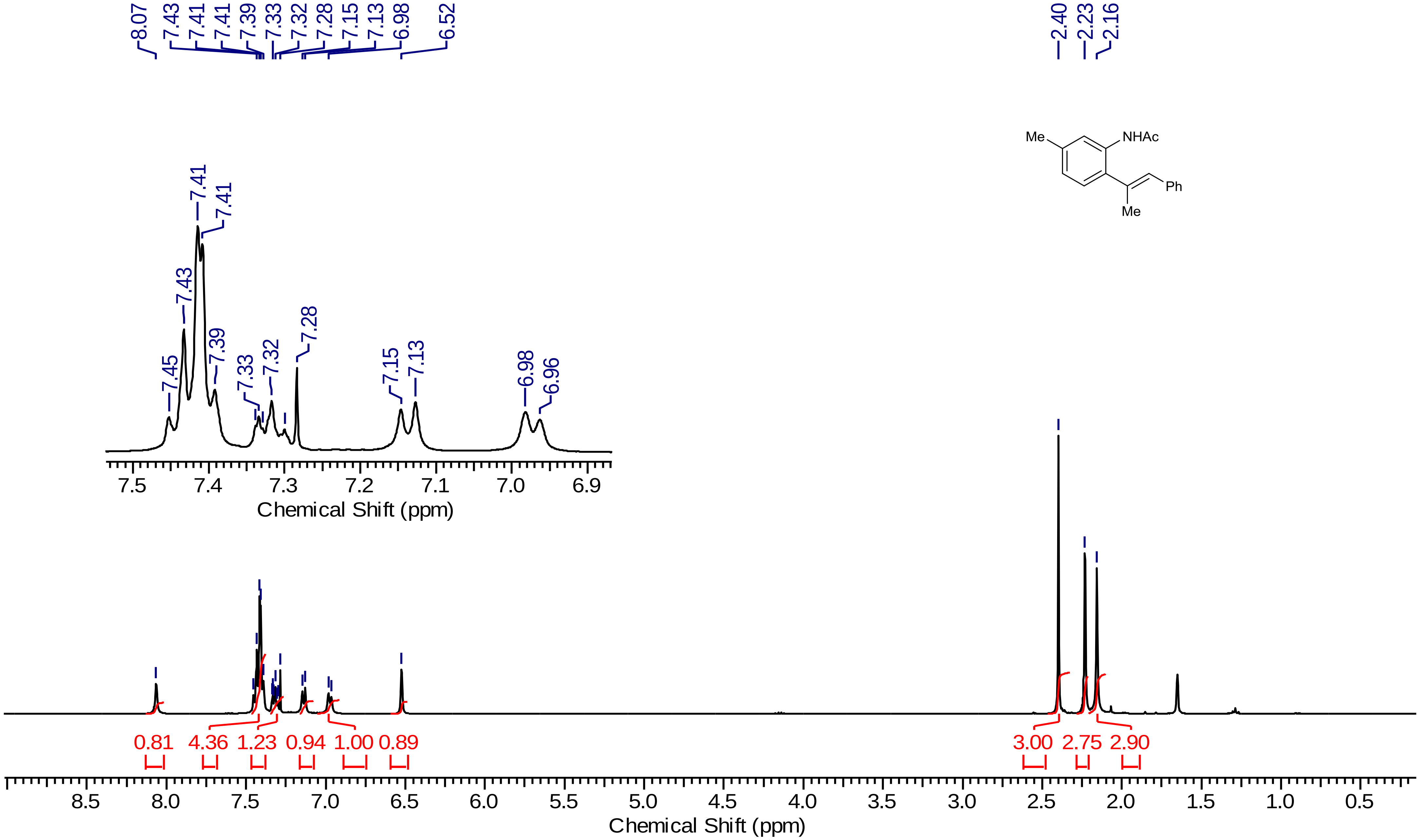

Supplementary Figure 56. ^{1}H NMR of **5c'**

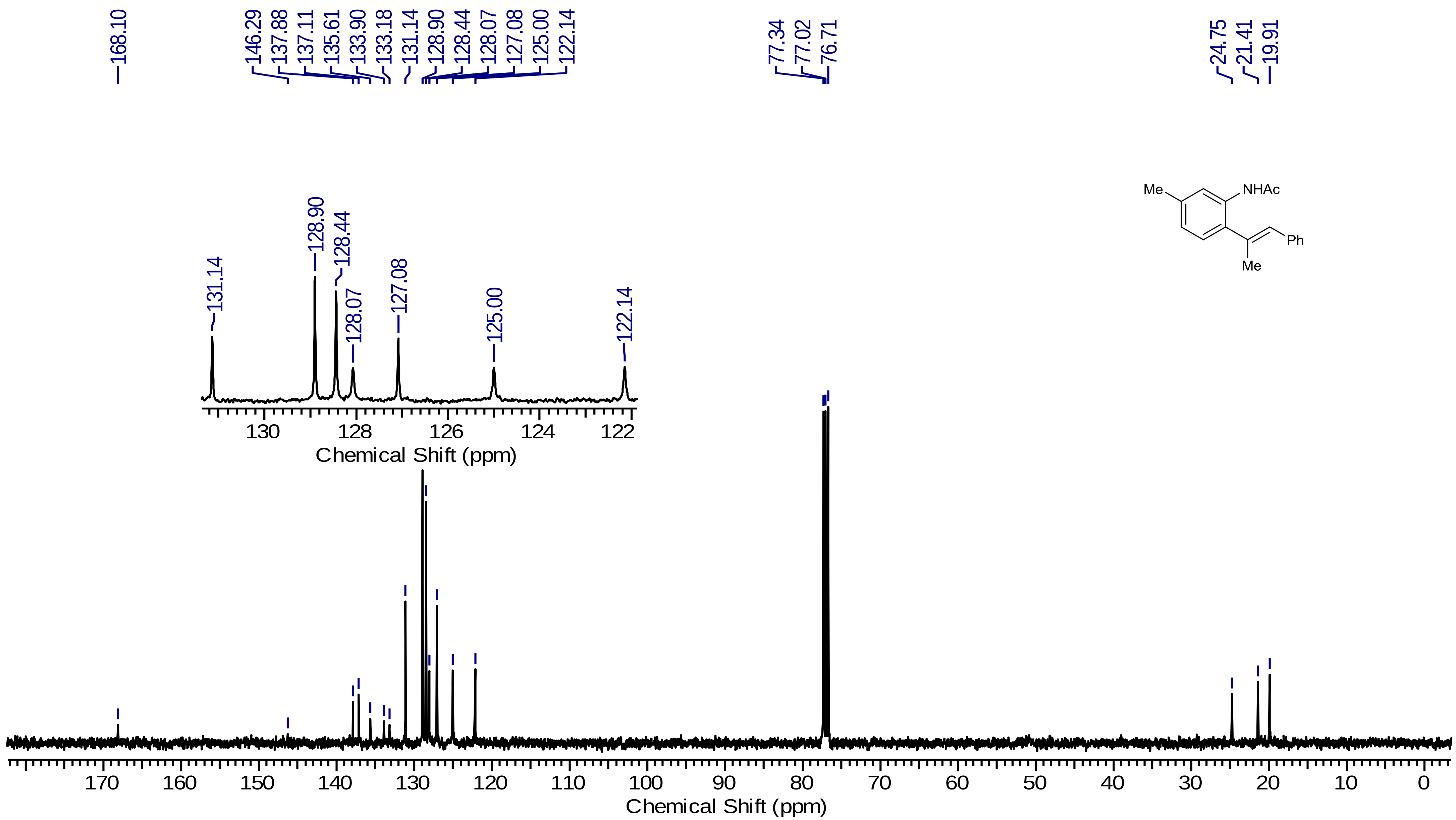

Supplementary Figure 57. ^{13}C NMR of **5c'**

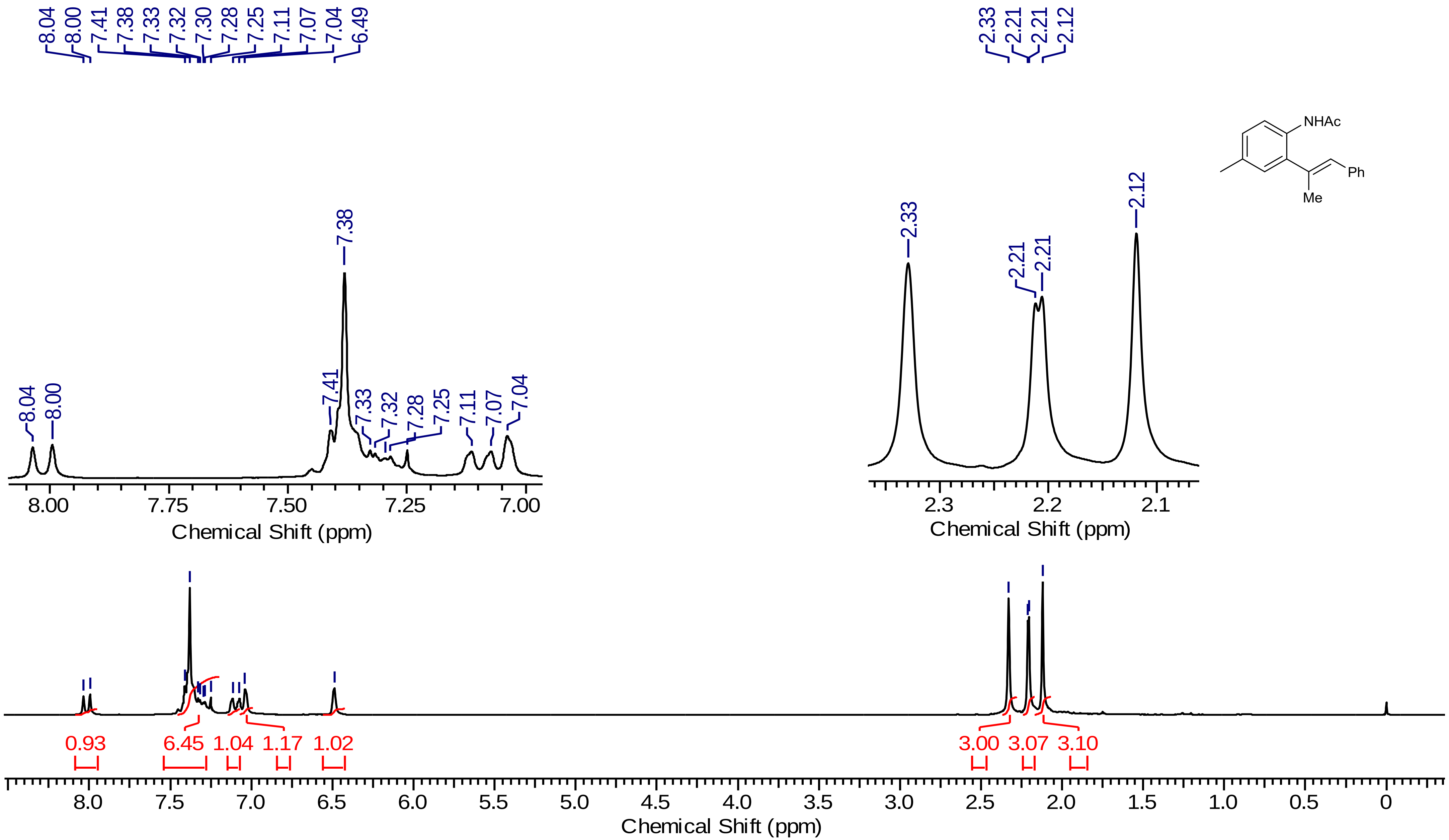

Supplementary Figure 58. ^{1}H NMR of **5d'**

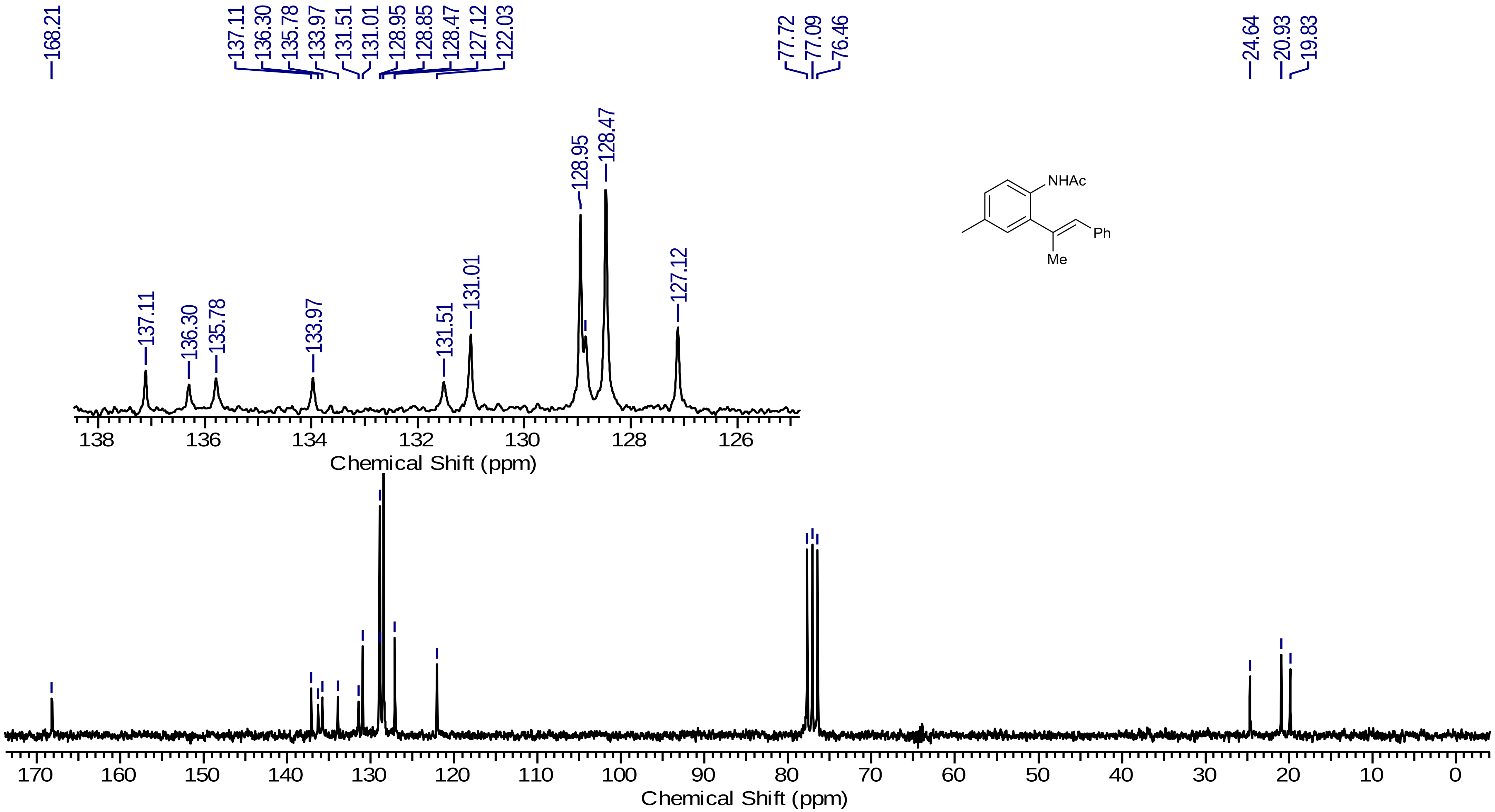

Supplementary Figure 59. ^{13}C NMR of **5d’**

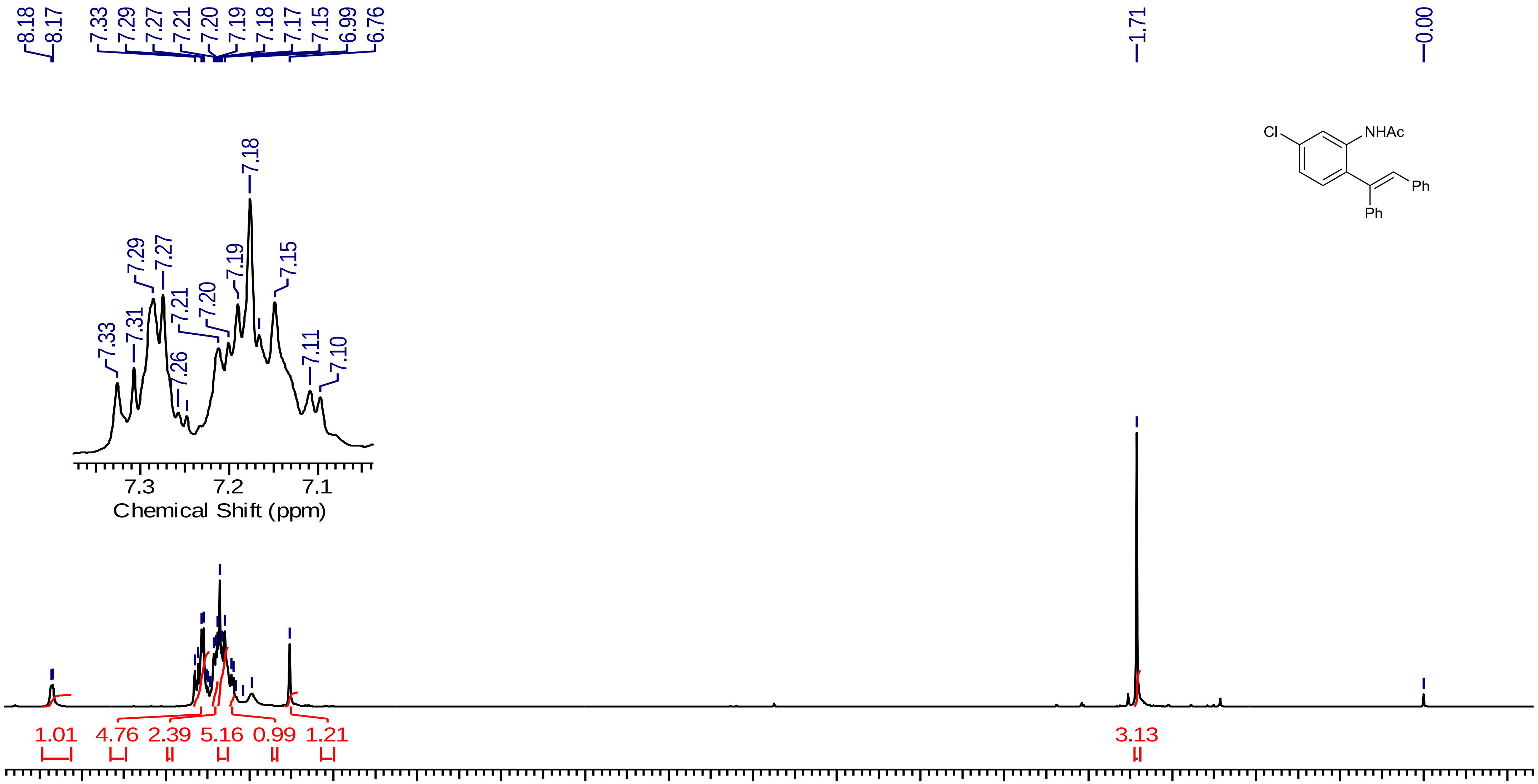

Supplementary Figure 60. ^{1}H NMR of **5e’**

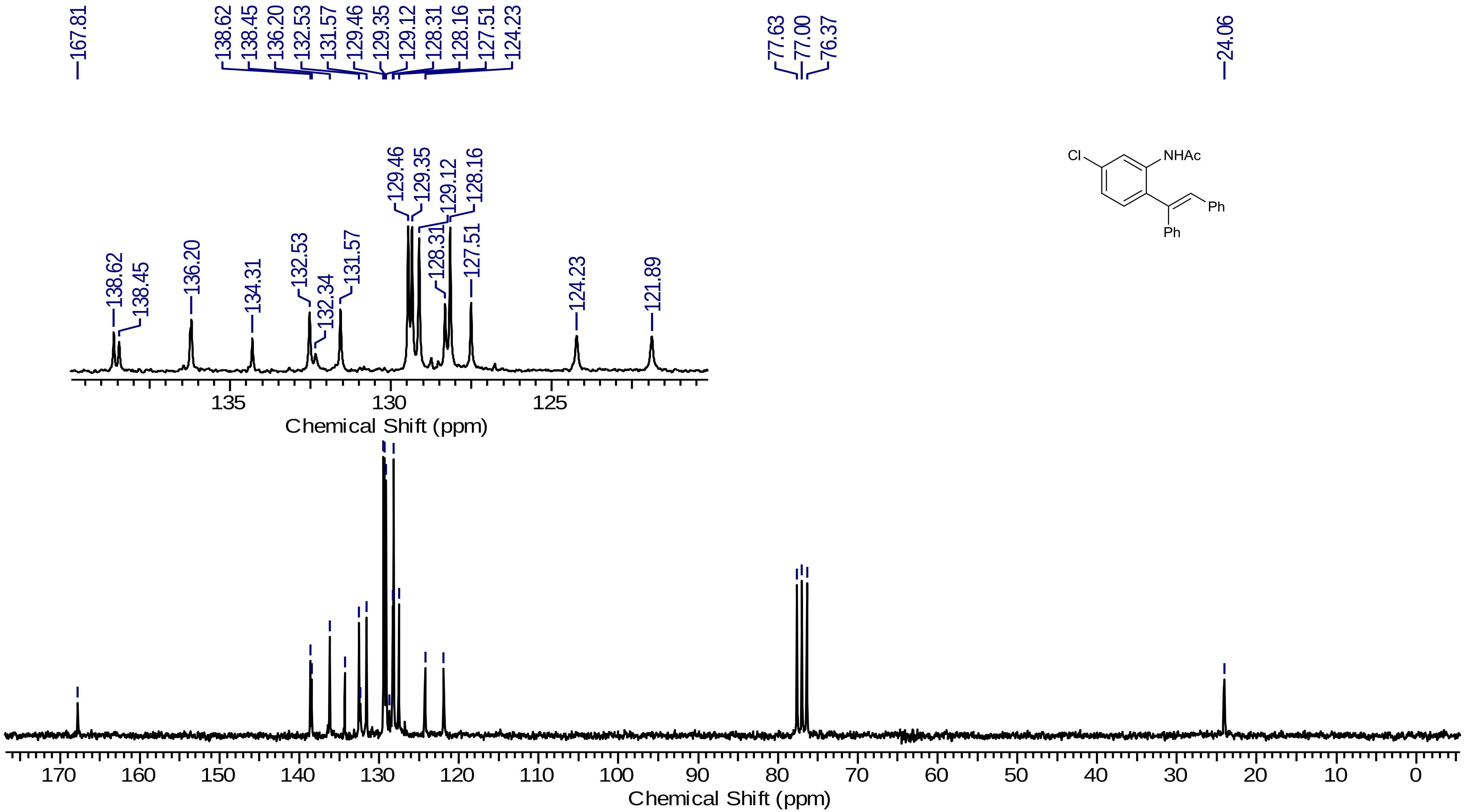

Supplementary Figure 61. ^{13}C NMR of **5e'**

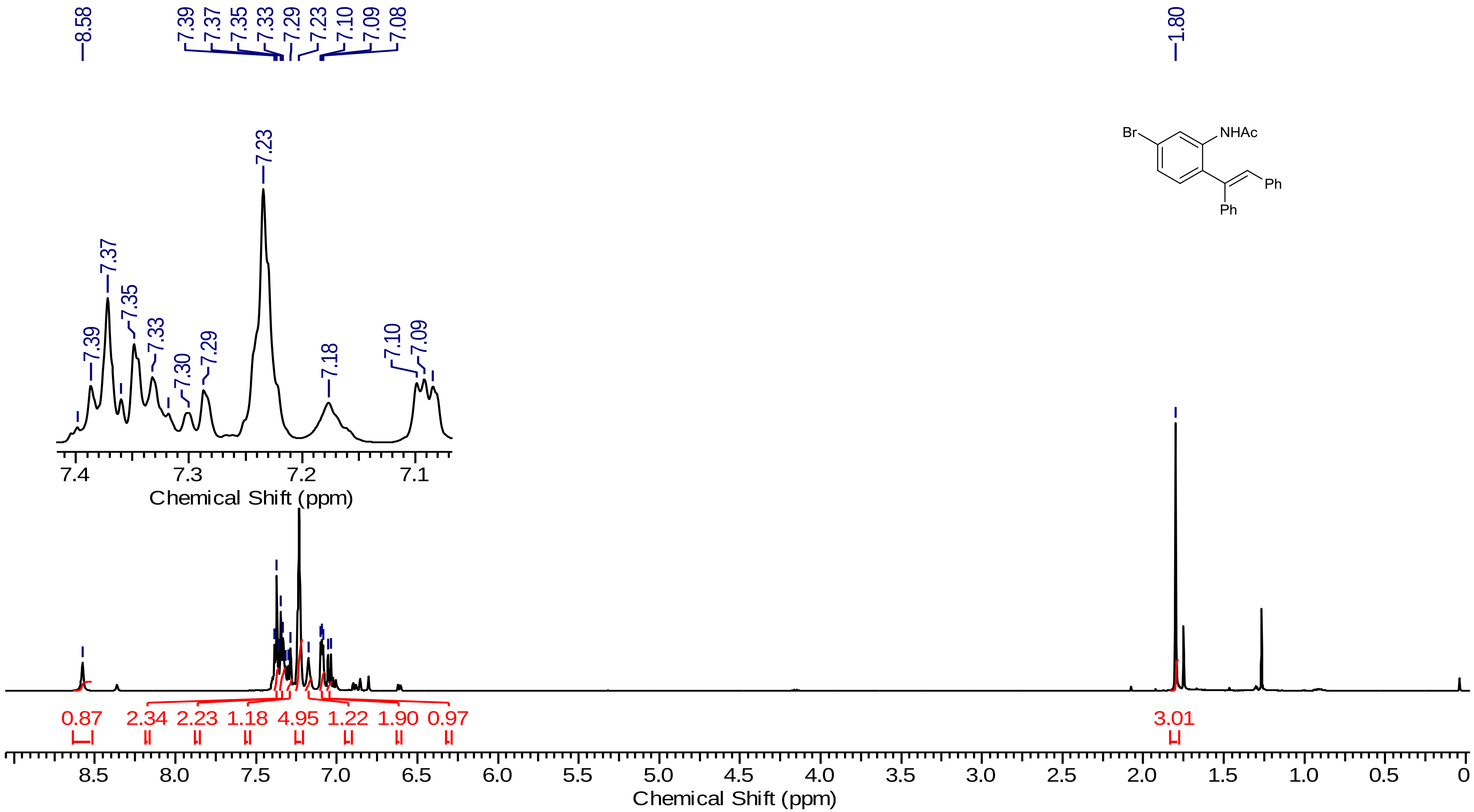

Supplementary Figure 62. ^{1}H NMR of **5f'**

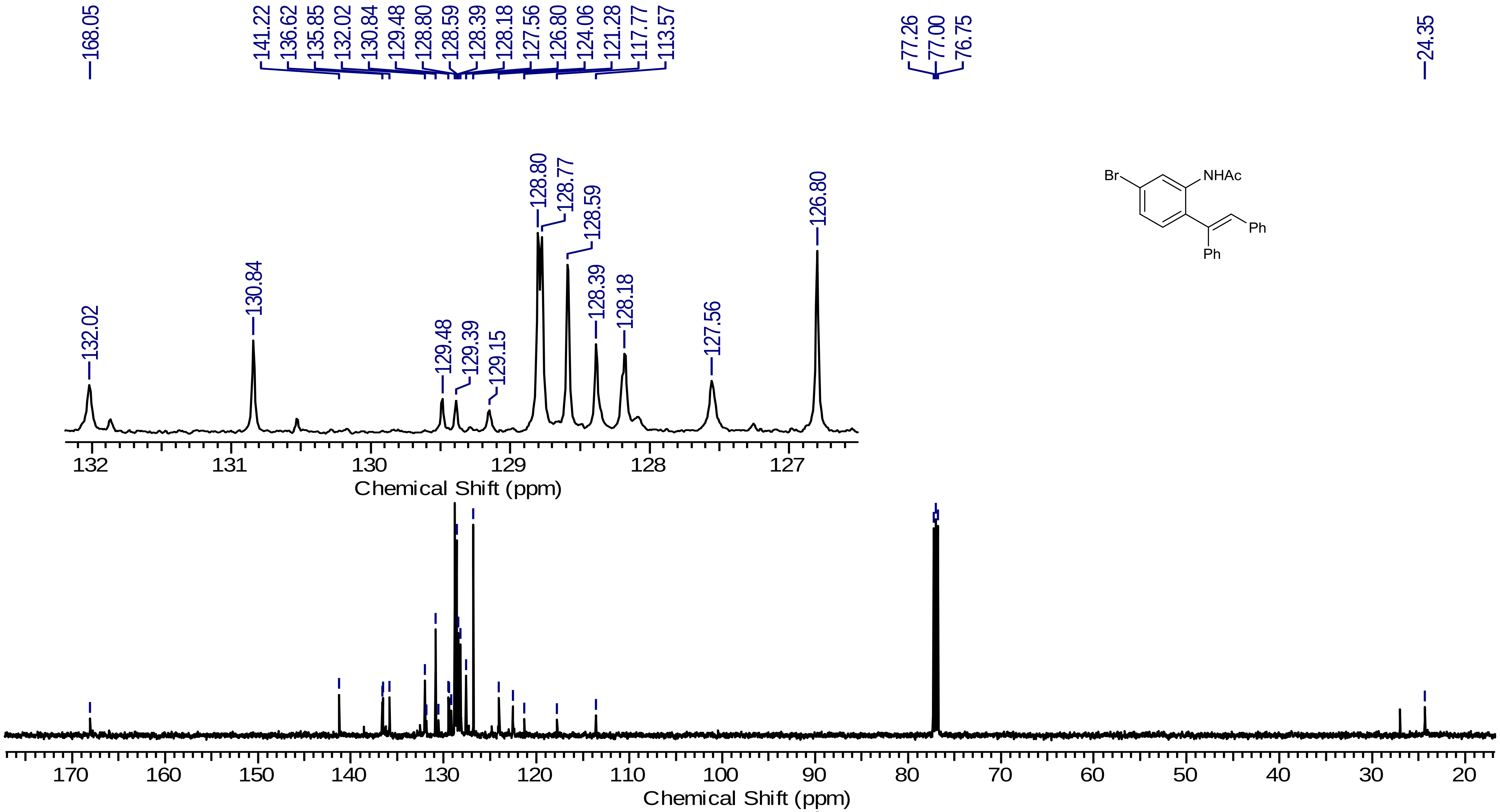

Supplementary Figure 63. ^{13}C NMR of **5f'**

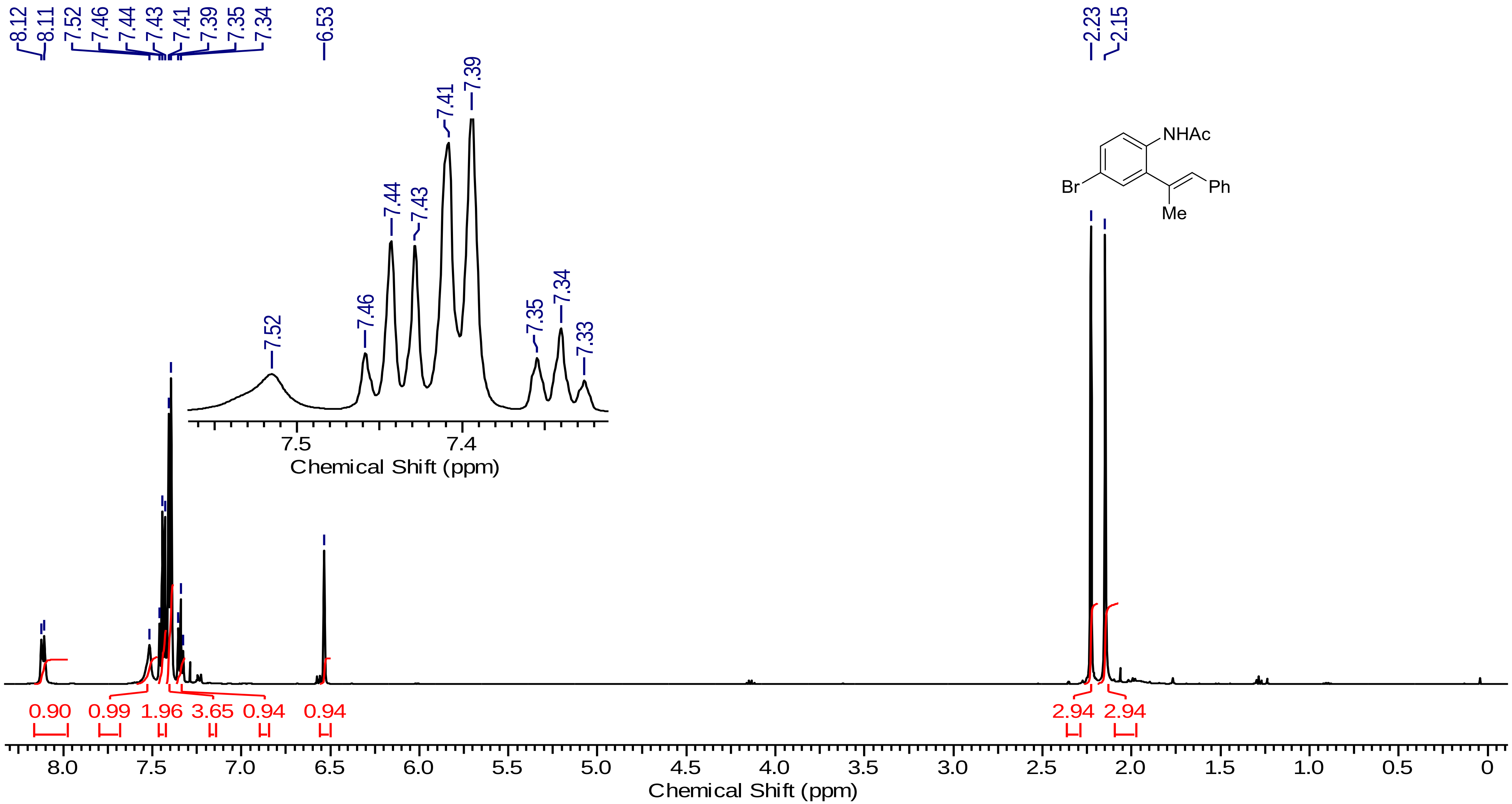

Supplementary Figure 64. ^{13}C NMR of **5g’**

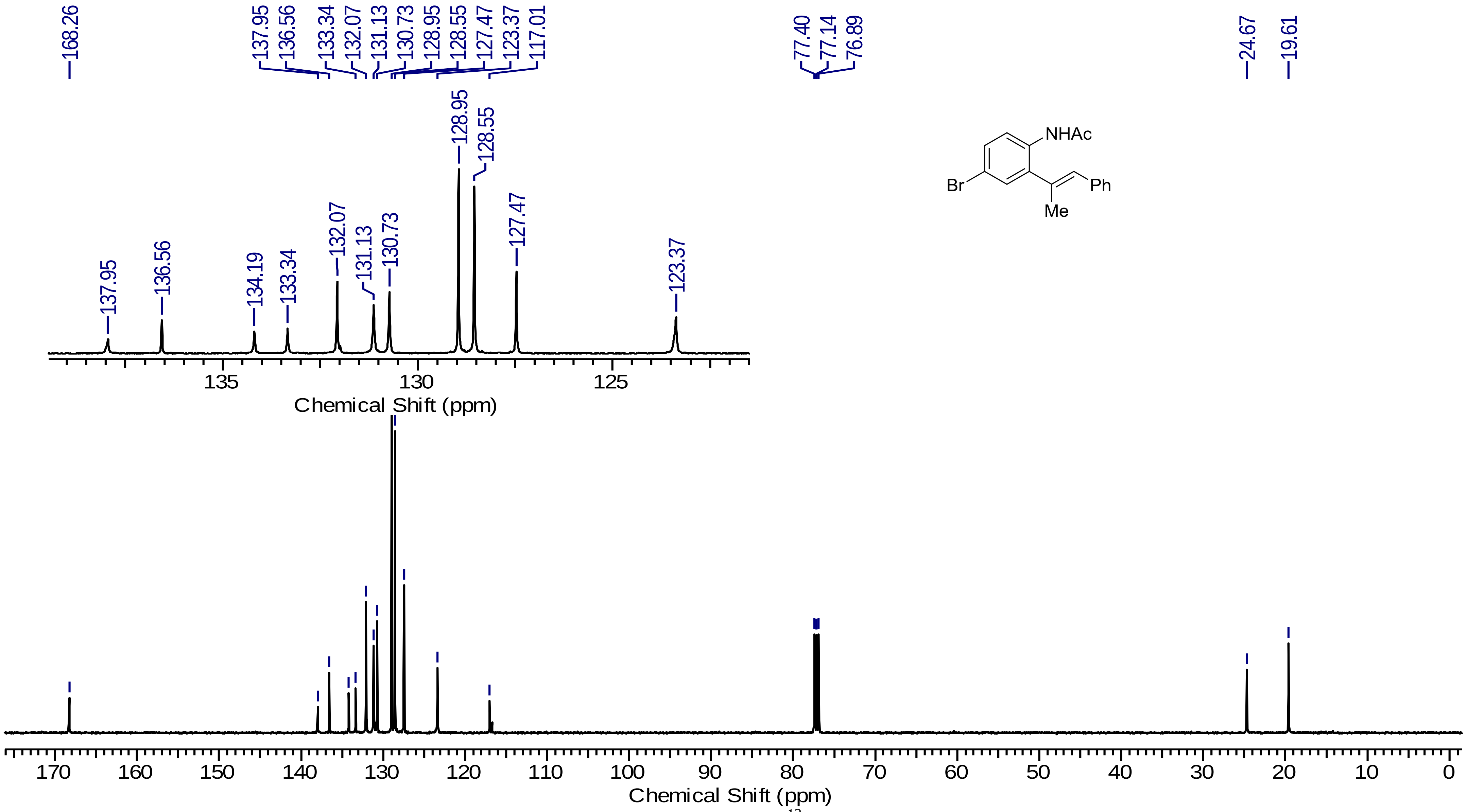

Supplementary Figure 65. ^{13}C NMR of **5g'**

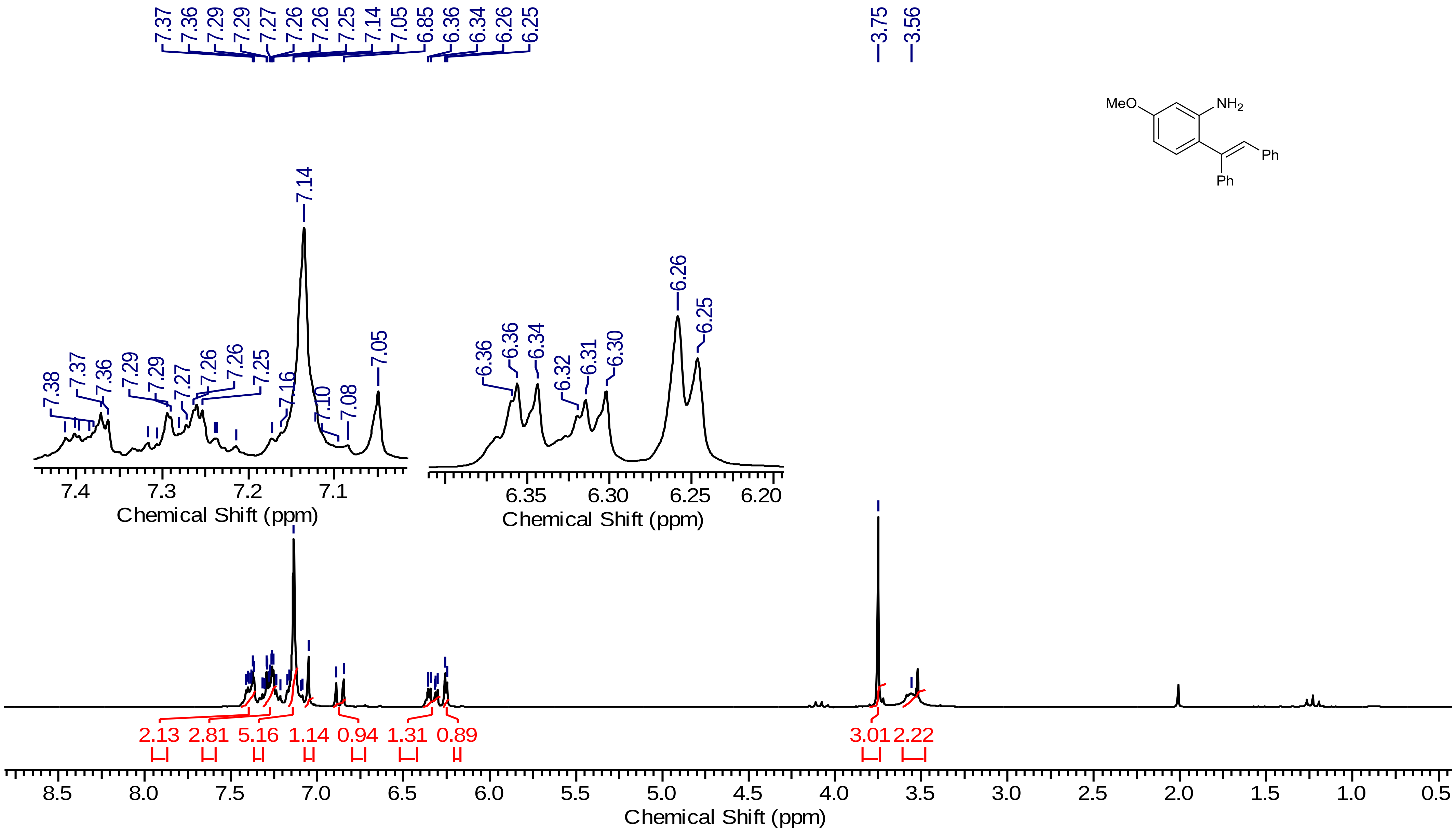

Supplementary Figure 66. ^{1}H NMR of **5a**

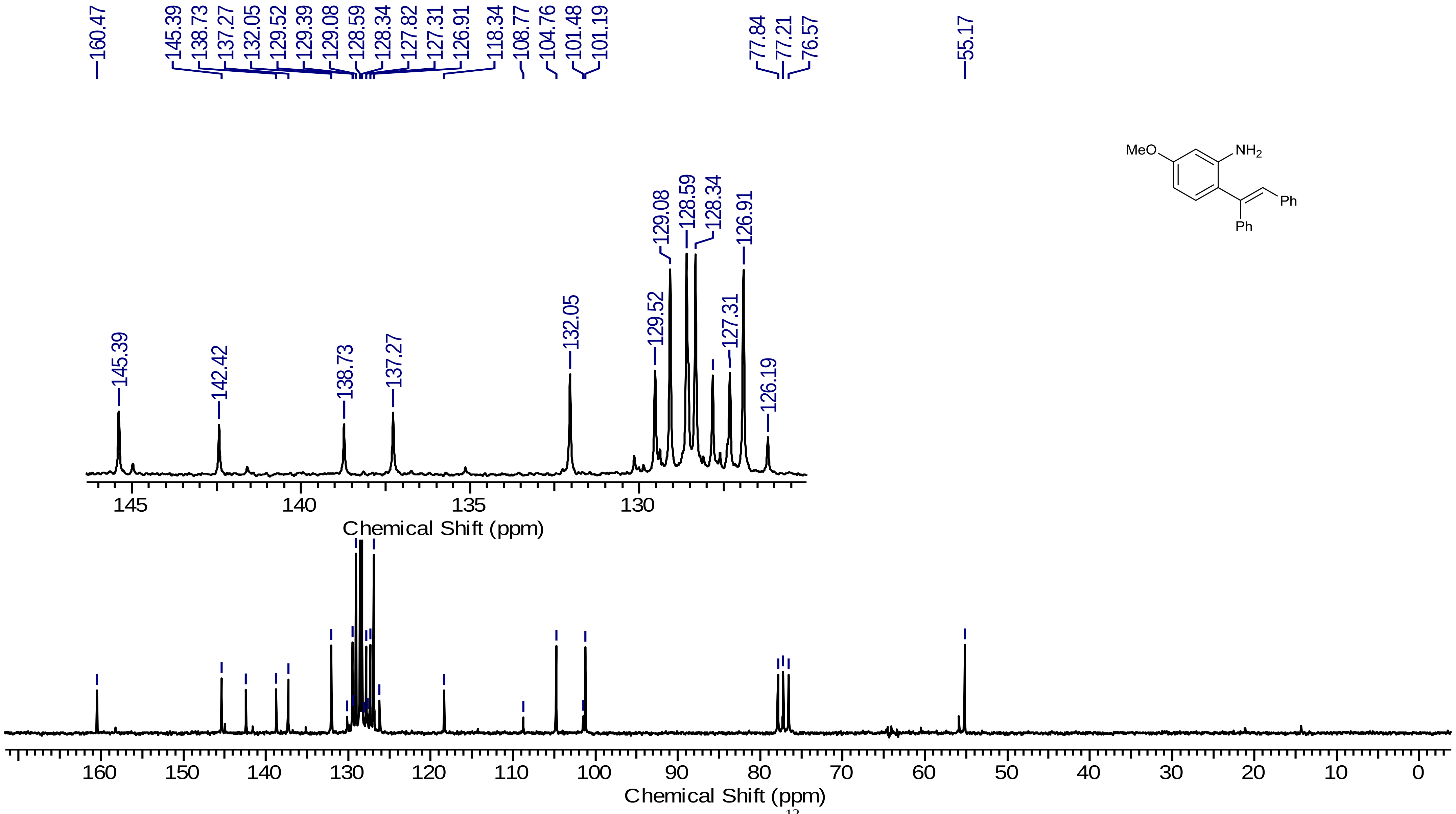

Supplementary Figure 67. ^{13}C NMR of **5a**

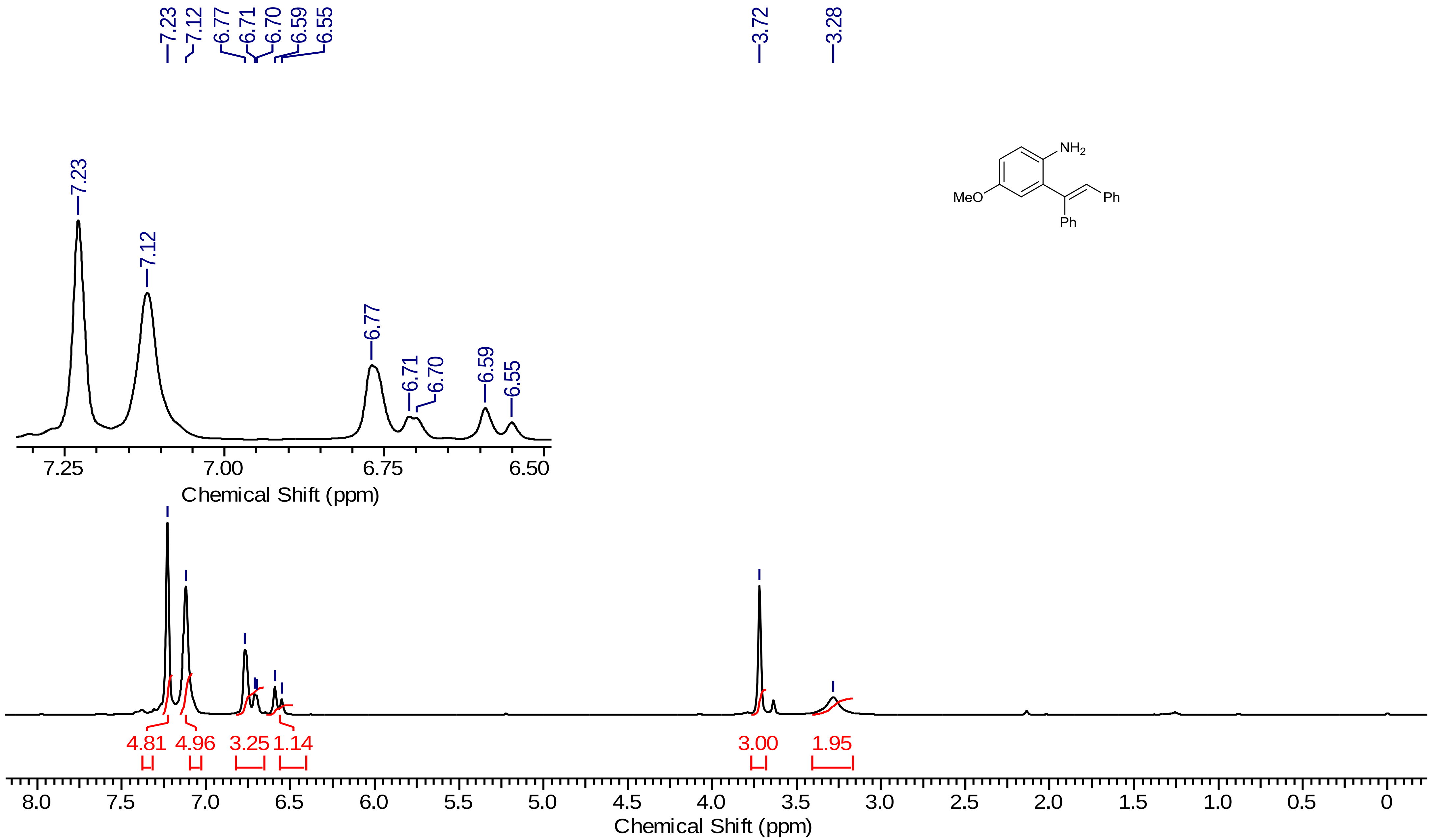

Supplementary Figure 68. ^{1}H NMR of **5b**

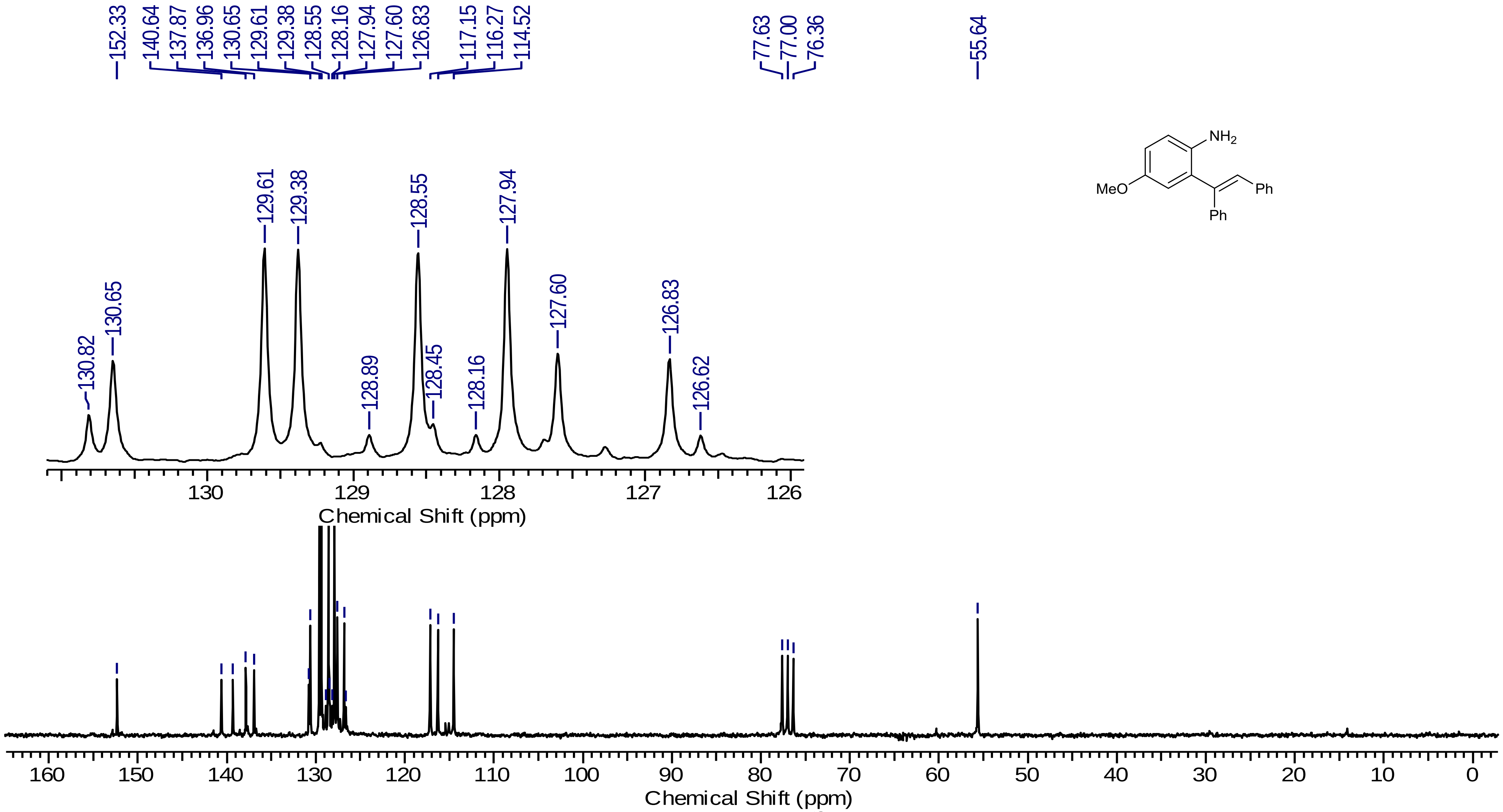

Supplementary Figure 69. ^{13}C NMR of **5b**

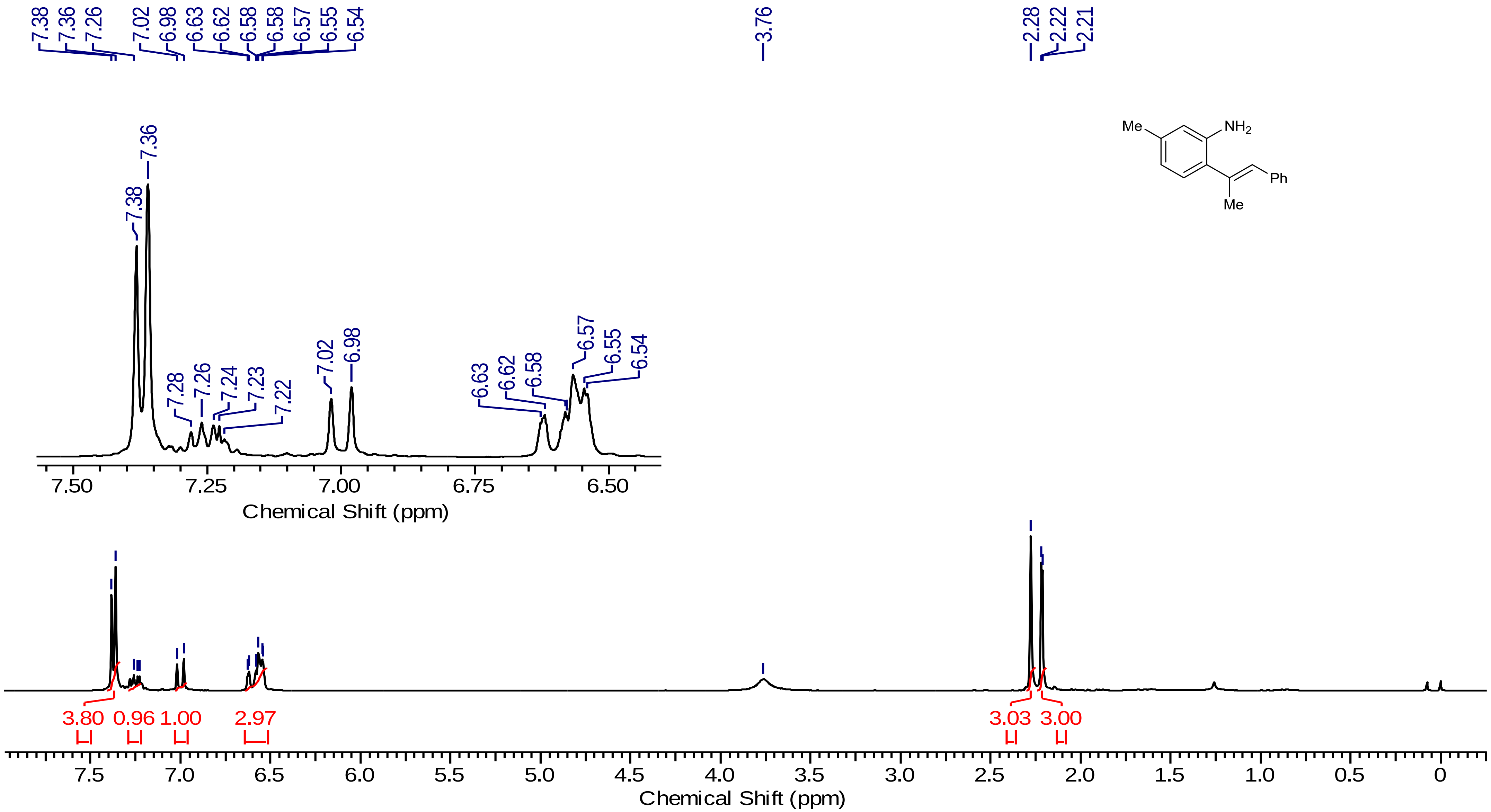

Supplementary Figure 70. ^{1}H NMR of **5c**

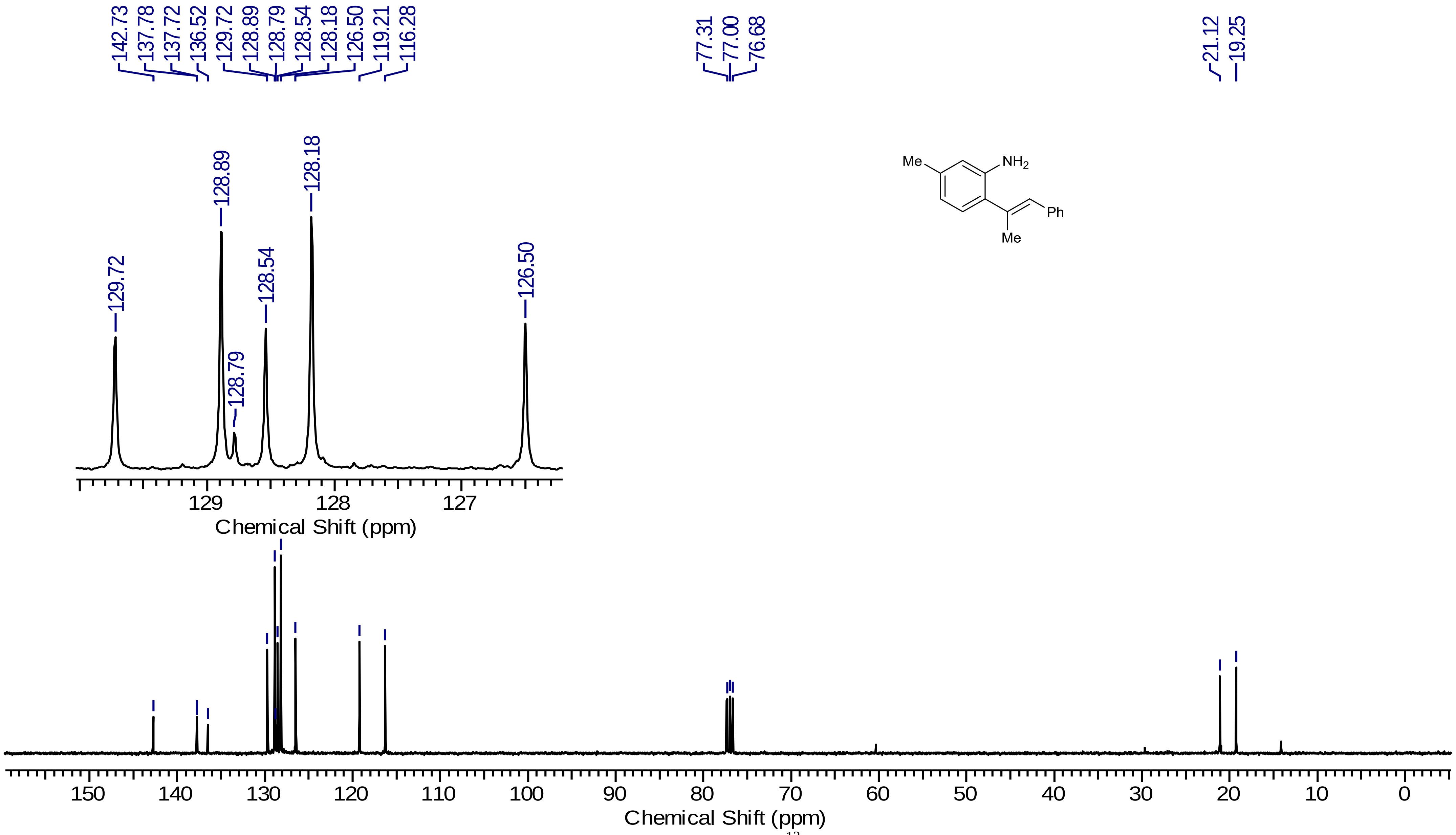

Supplementary Figure 71. ^{13}C NMR of **5c**

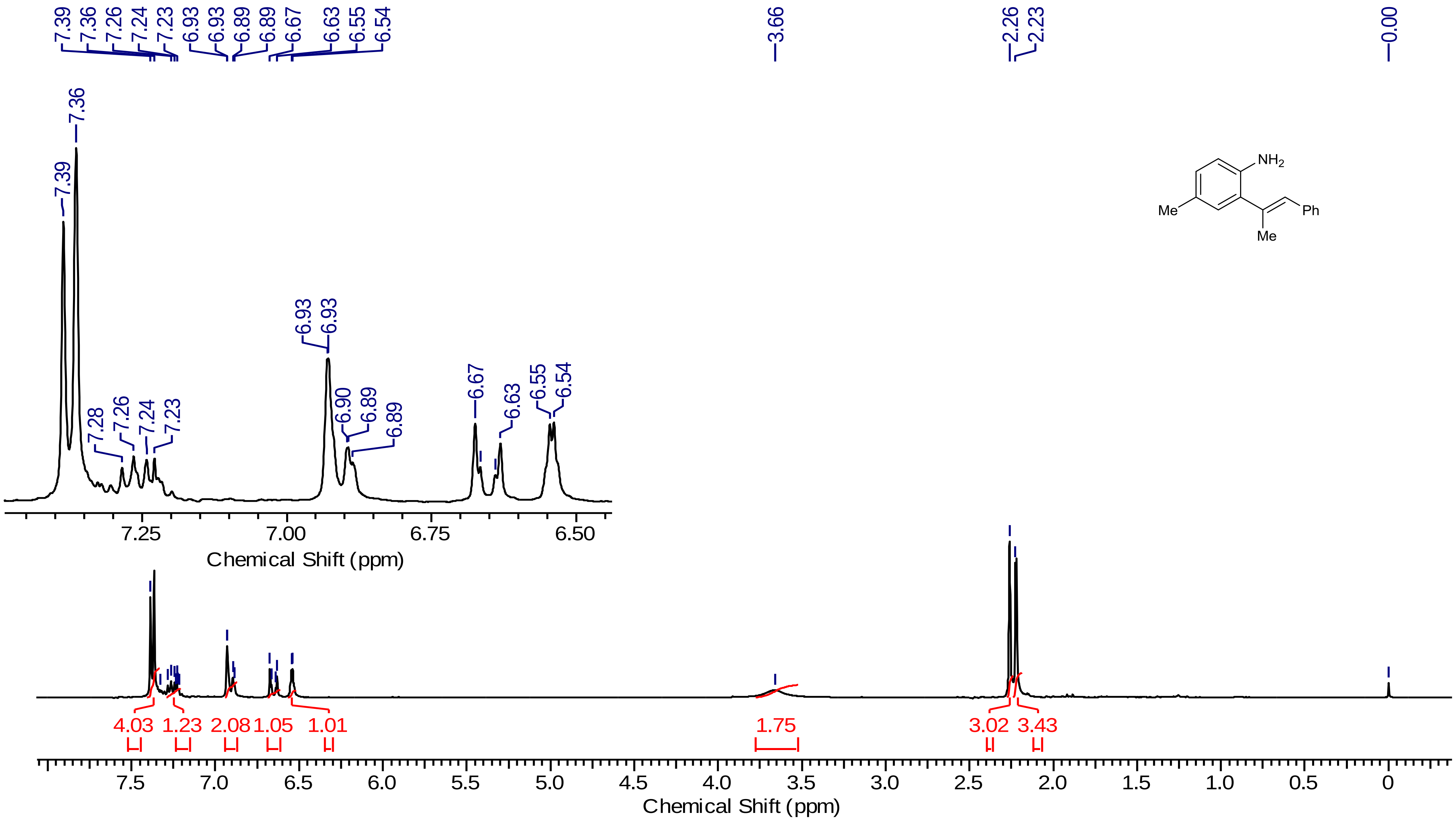

Supplementary Figure 72. ^{1}H NMR of **5d**

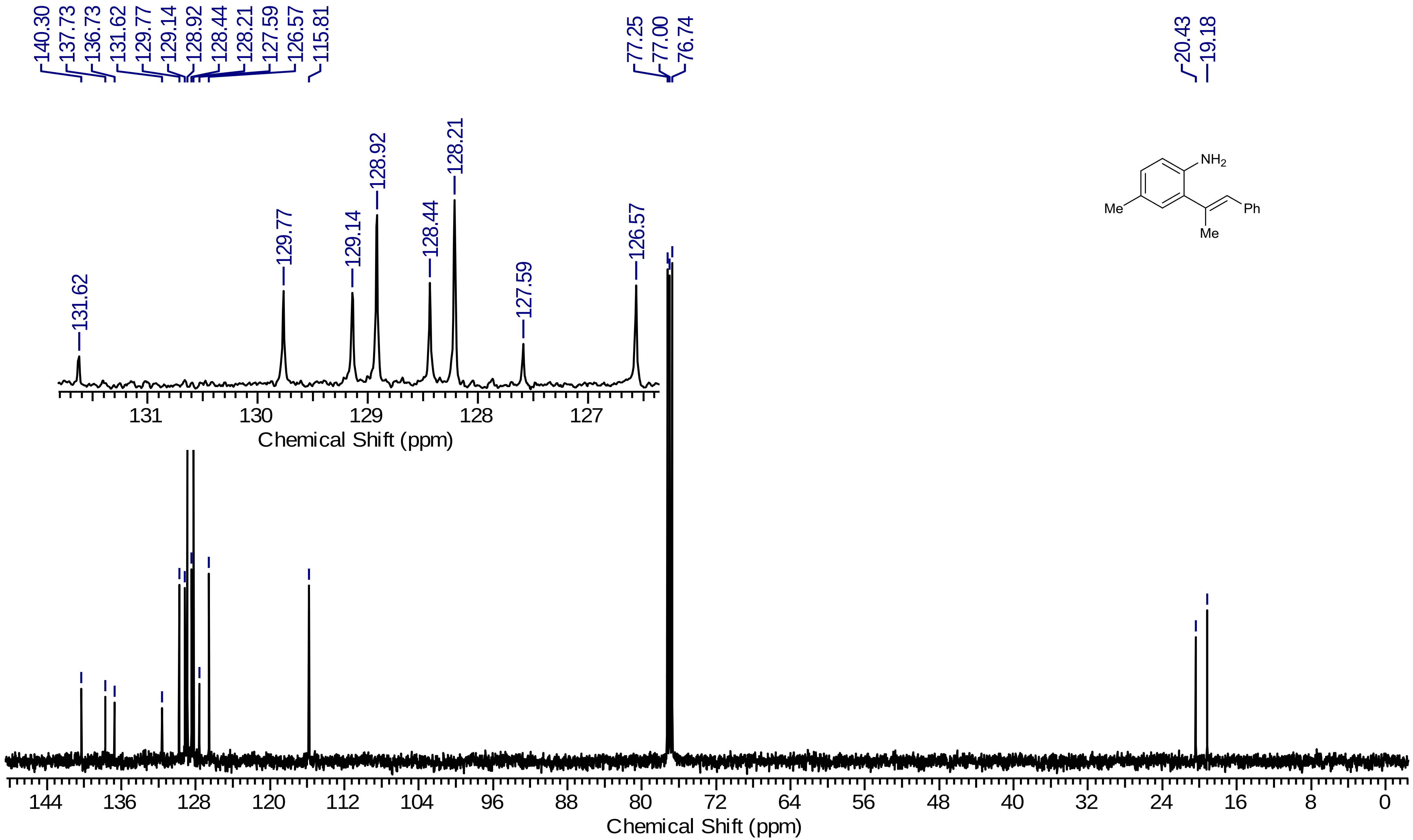

Supplementary Figure 73. ^{13}C NMR of **5d**

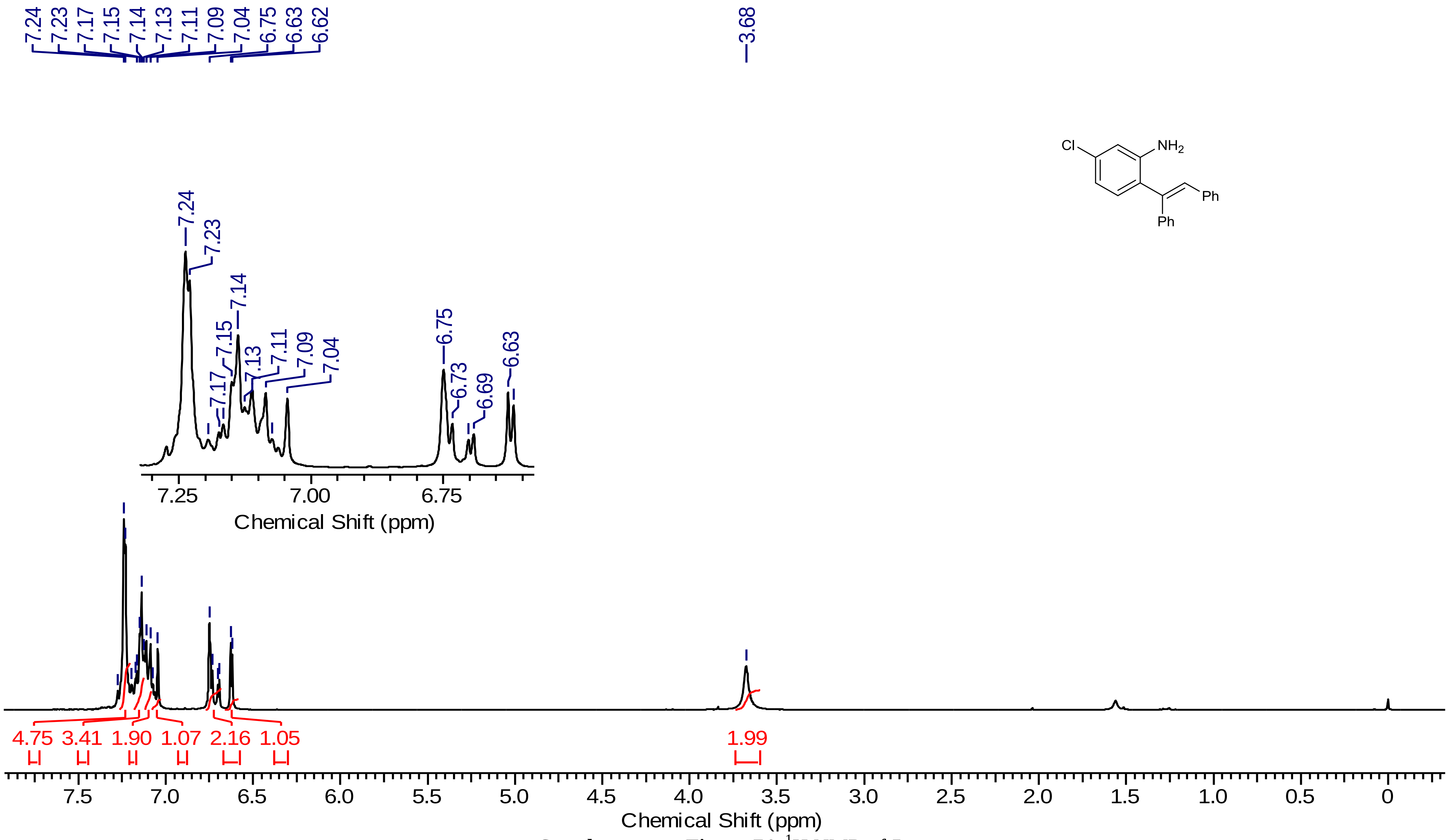

Supplementary Figure 74. ^{1}H NMR of **5e**

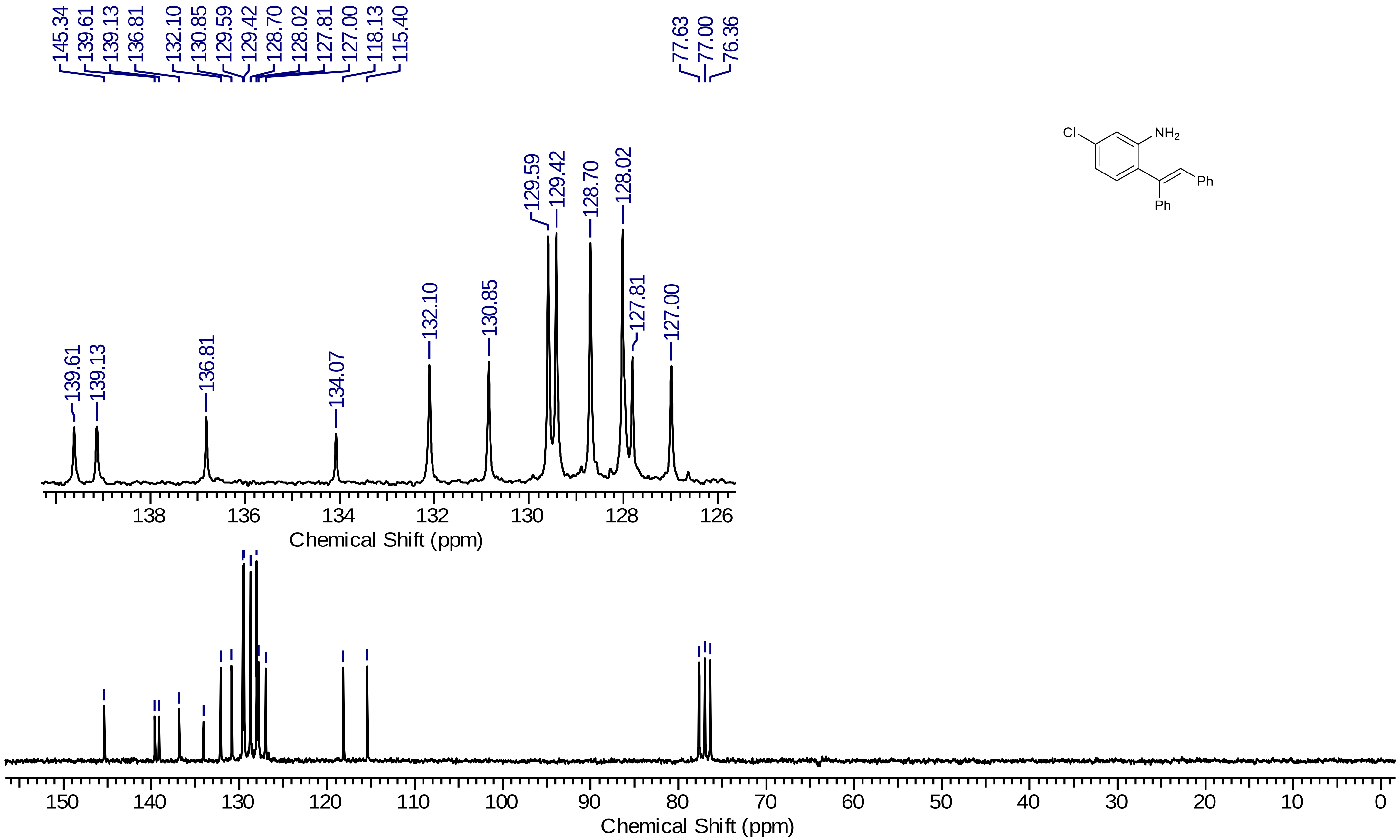

Supplementary Figure 75. ^{13}C NMR of **5e**

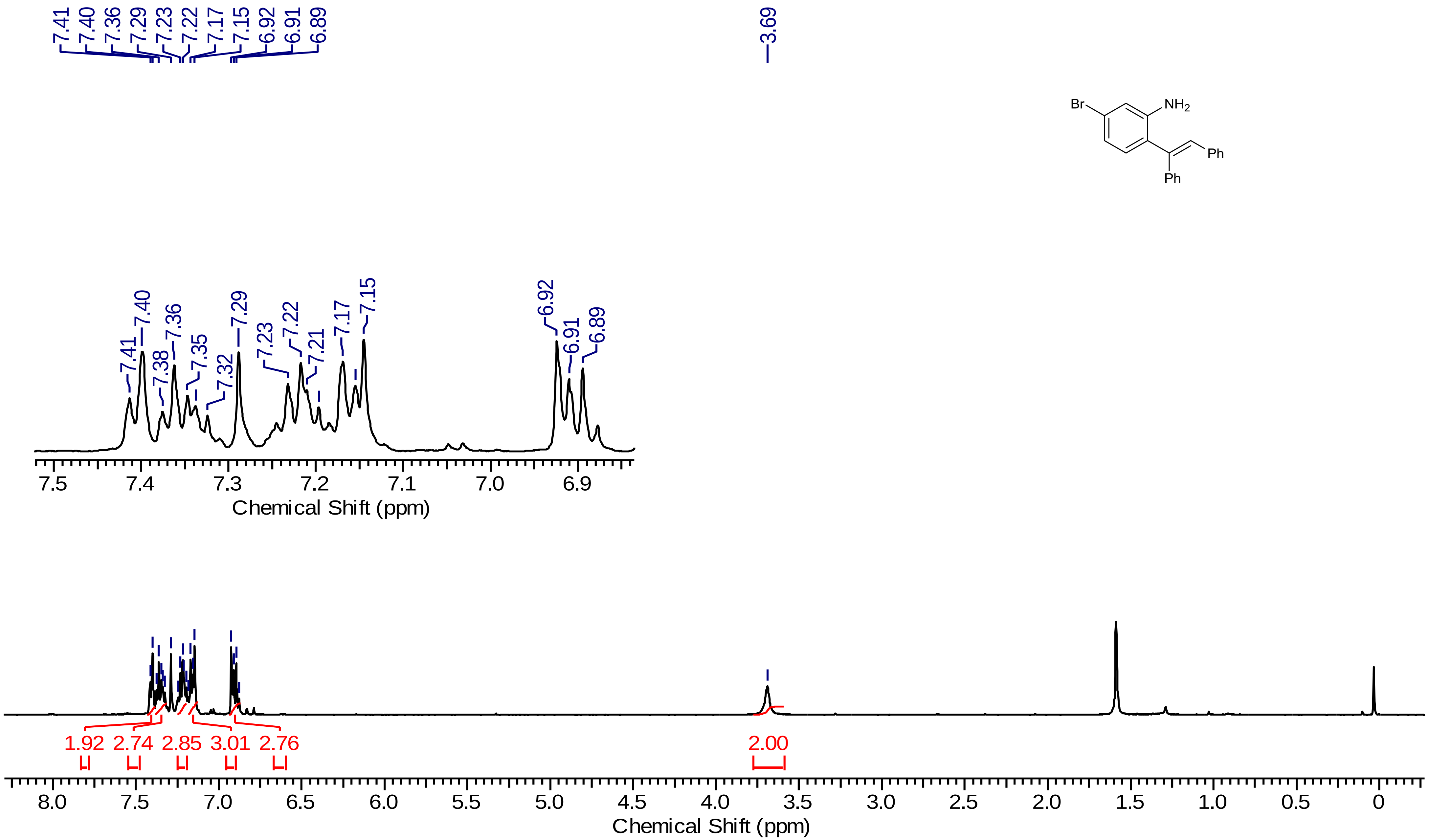

Supplementary Figure 76. ^{1}H NMR of **5f**

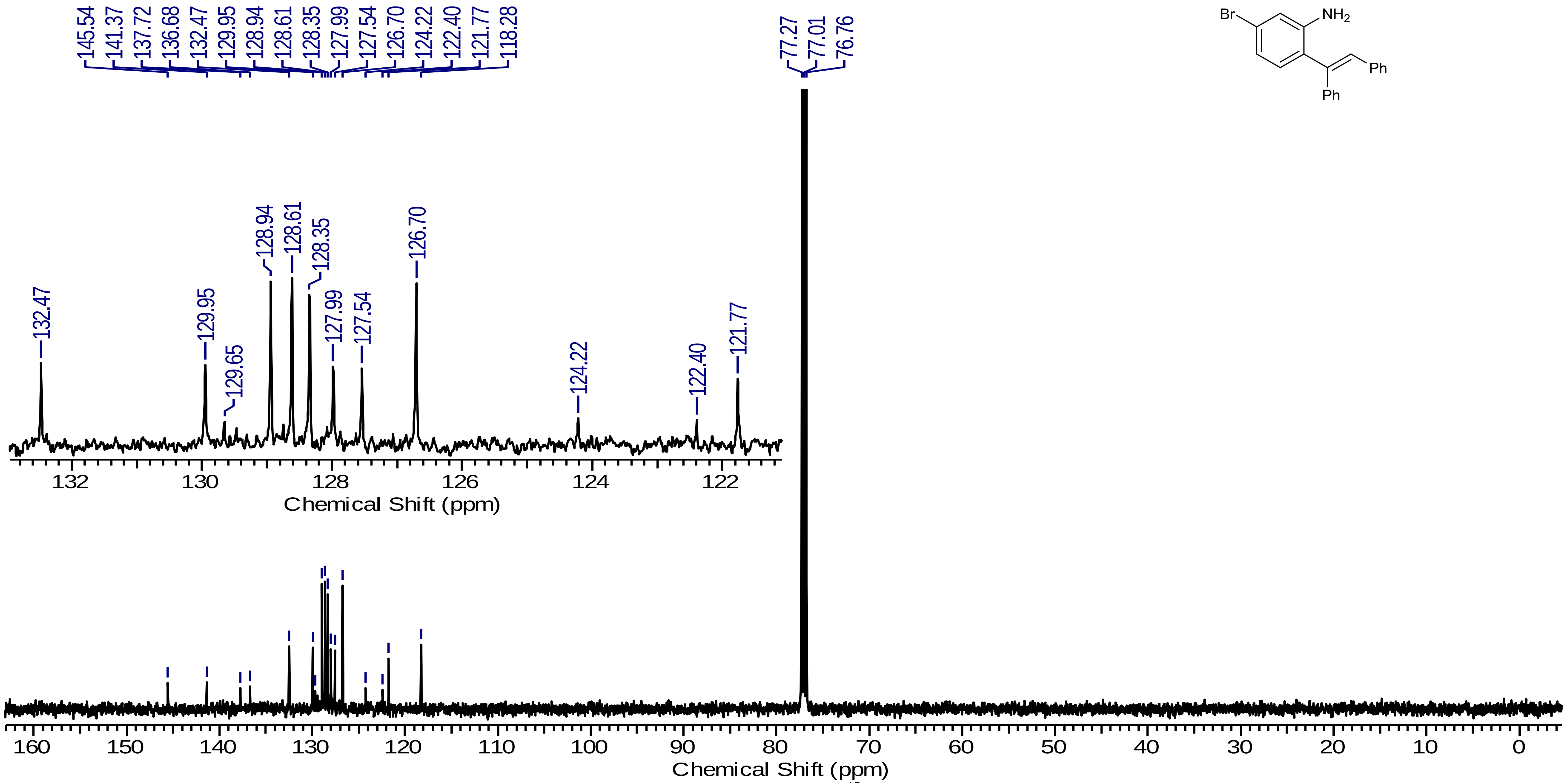

Supplementary Figure 77. ^{13}C NMR of **5f**

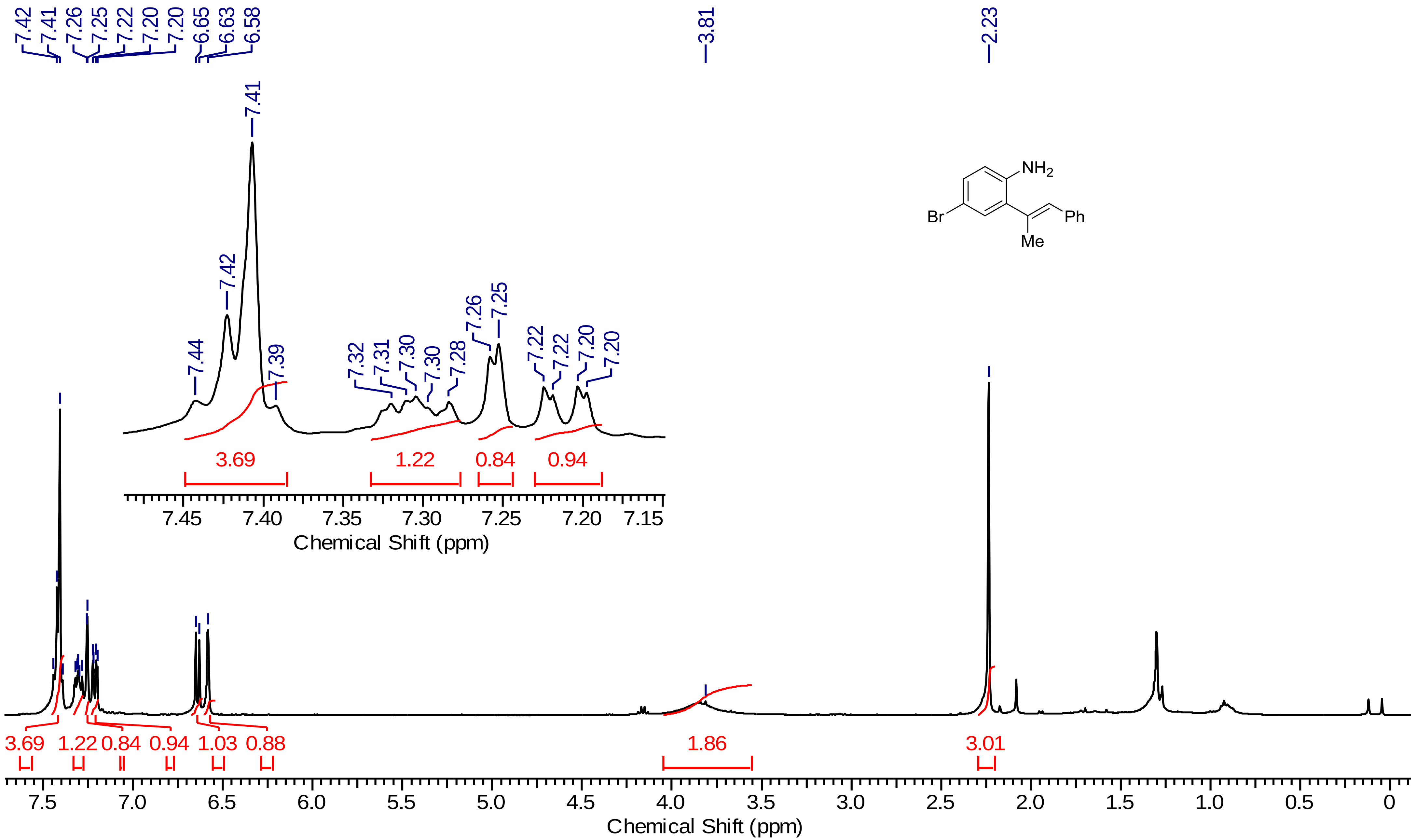

Supplementary Figure 78. ^{1}H NMR of **5g**

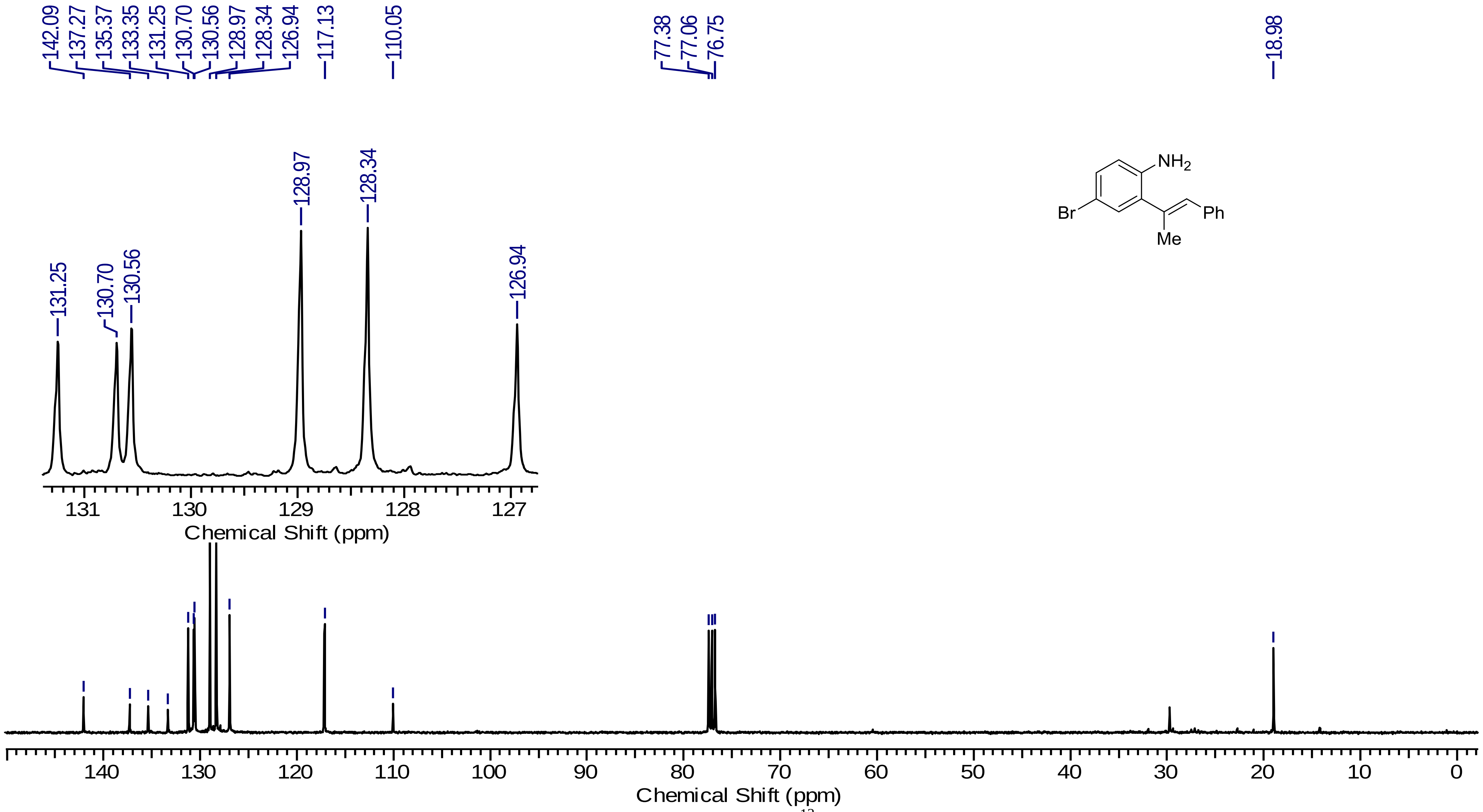

Supplementary Figure 79. ^{13}C NMR of **5g**

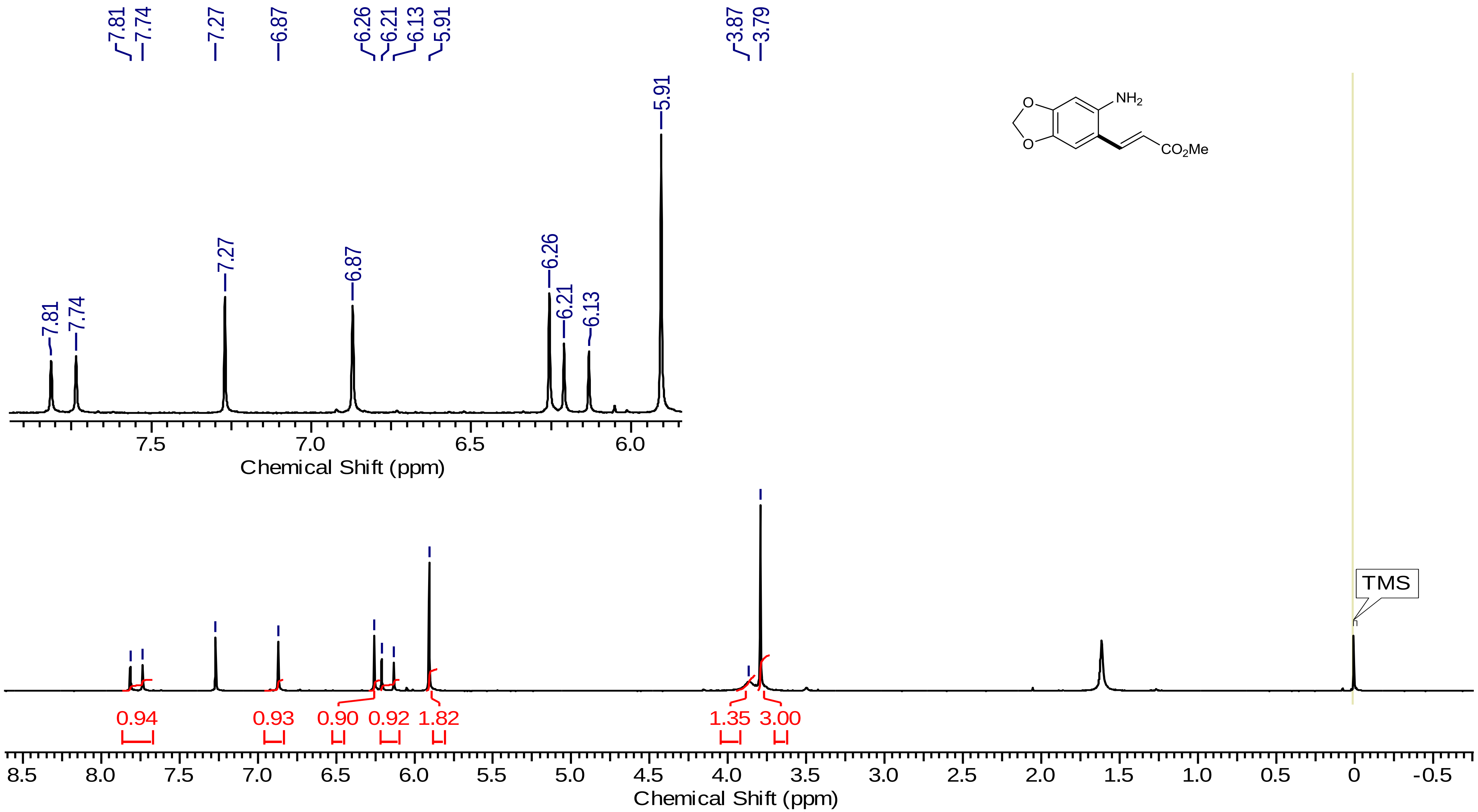

Supplementary Figure 80. ^{1}H NMR of **3a**

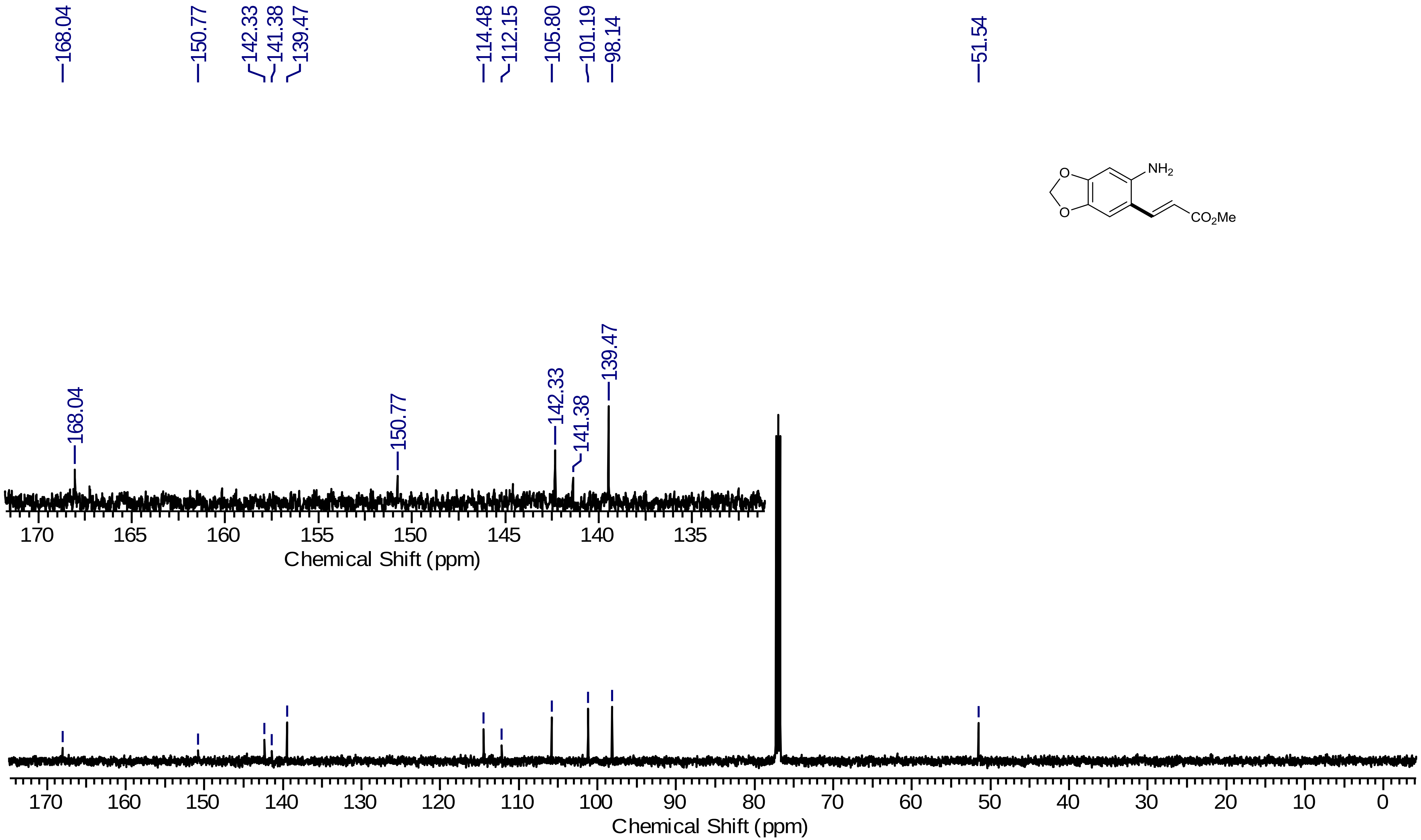

Supplementary Figure 81. ^{13}C NMR of **3a**

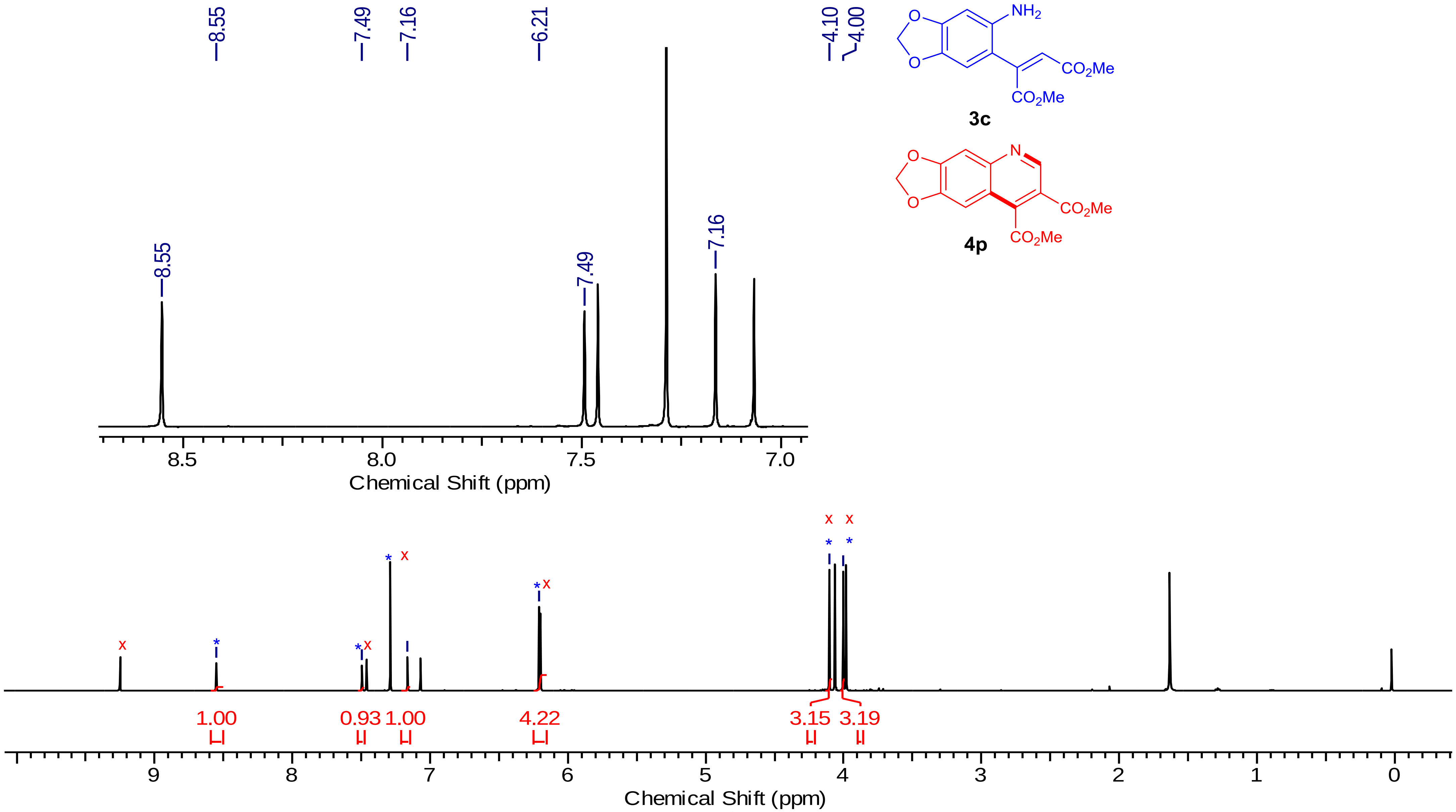

Supplementary Figure 82. ^{1}H NMR of **3c** (contains **4p**)

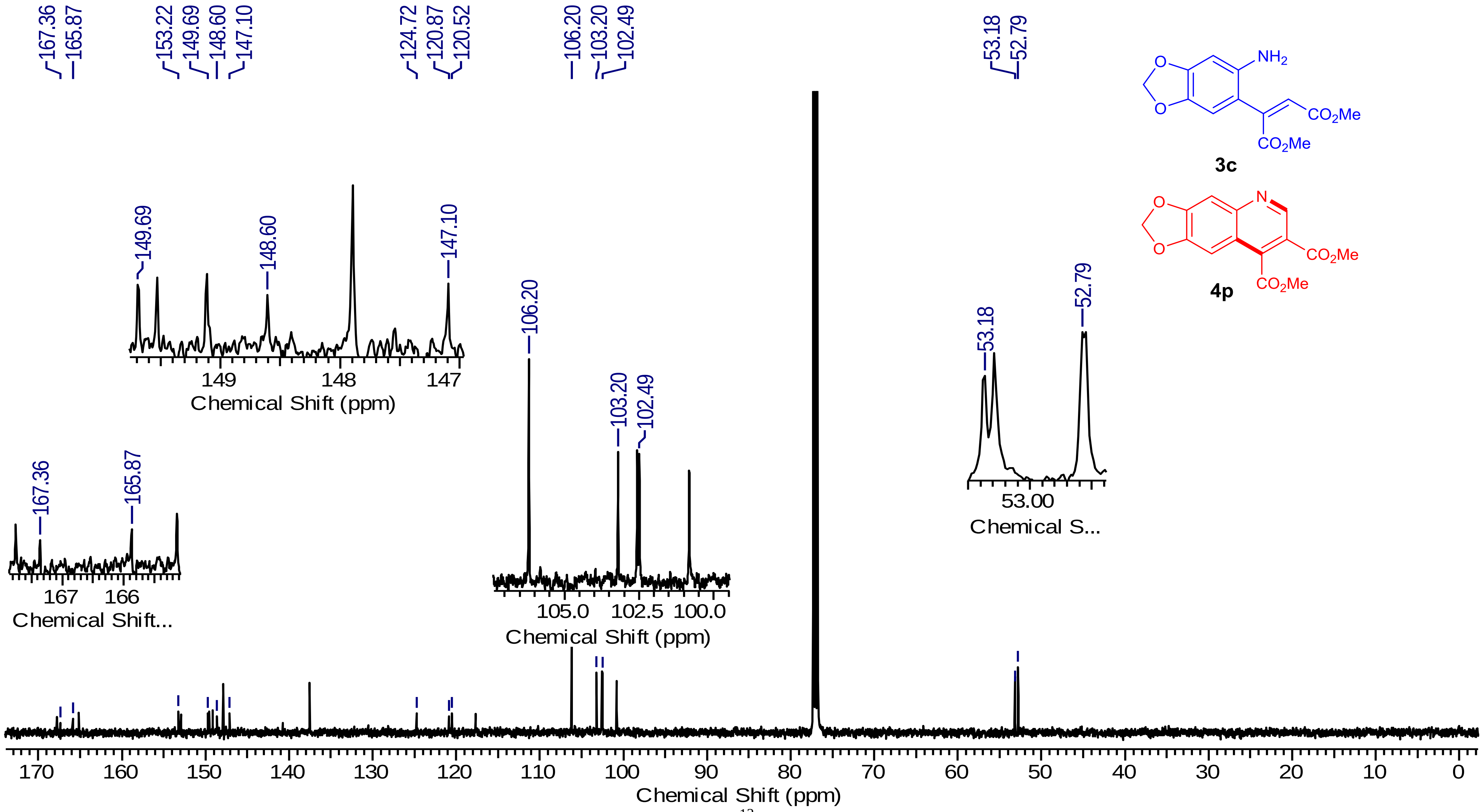

Supplementary Figure 83. ^{13}C NMR of **3c** (contains **4p**)

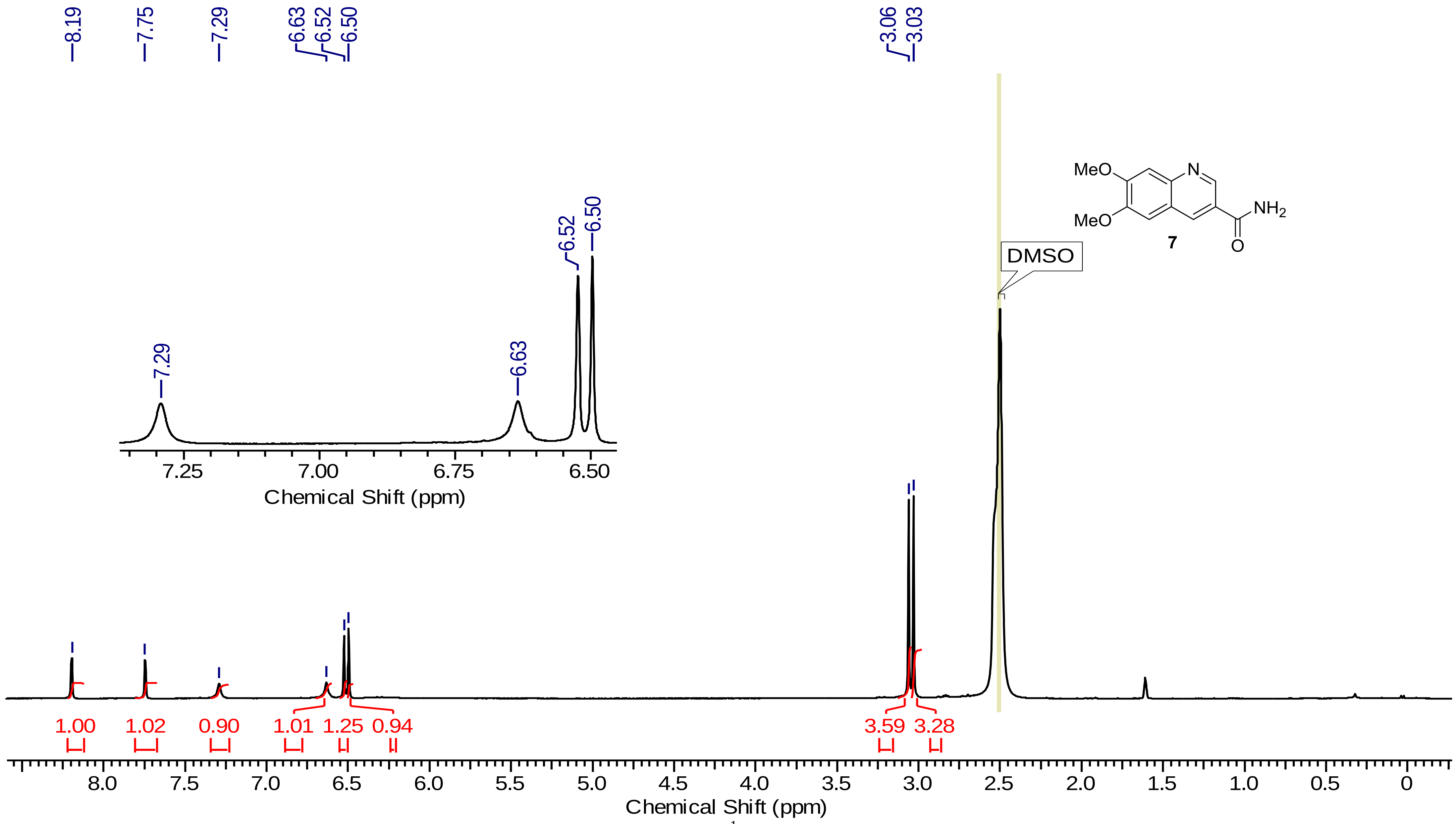

Supplementary Figure 84. ^{1}H NMR of **7** (in DMSO-d_6)

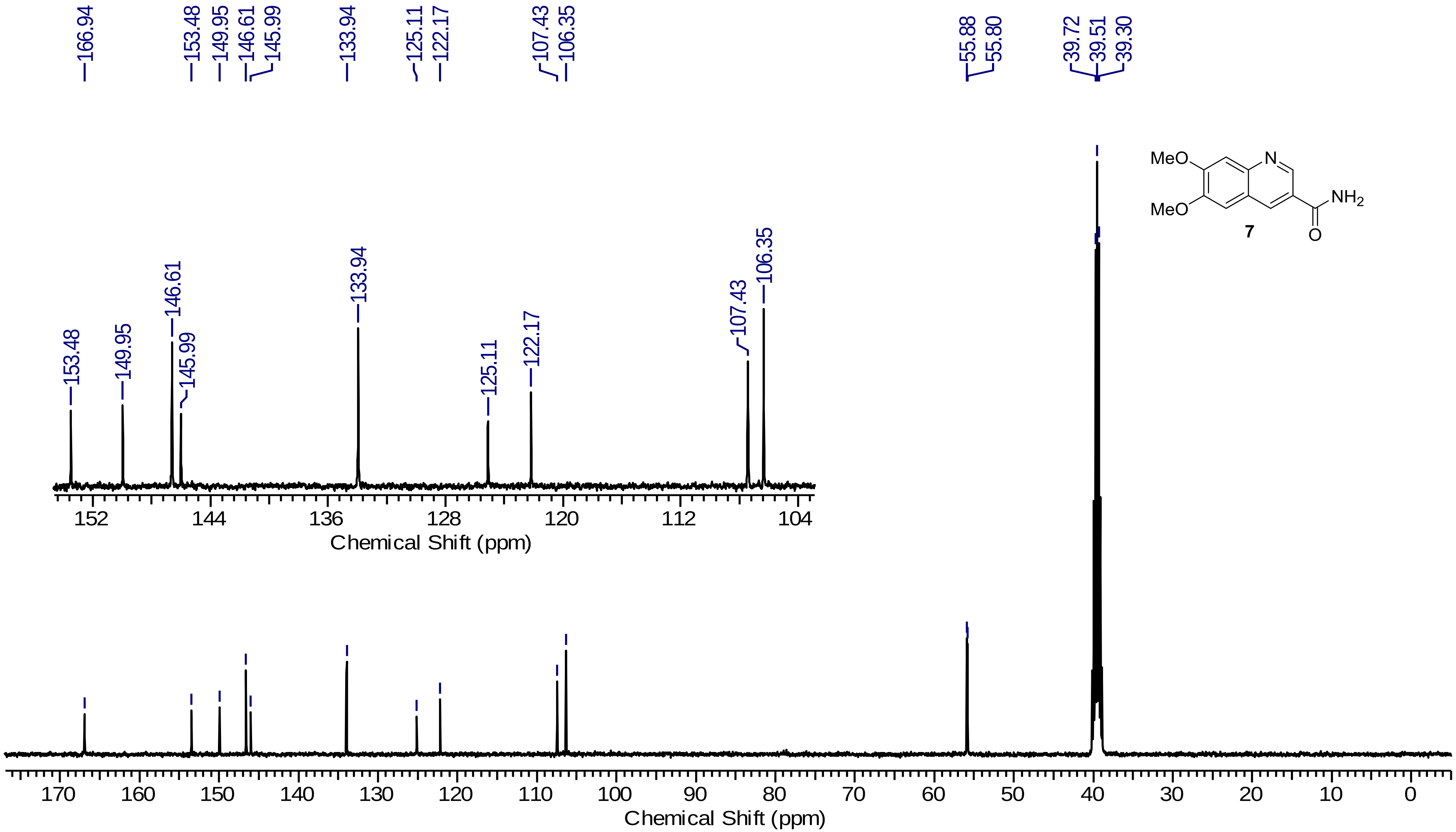

Supplementary Figure 85. ^{13}C NMR of **7** (in DMSO-d_6)

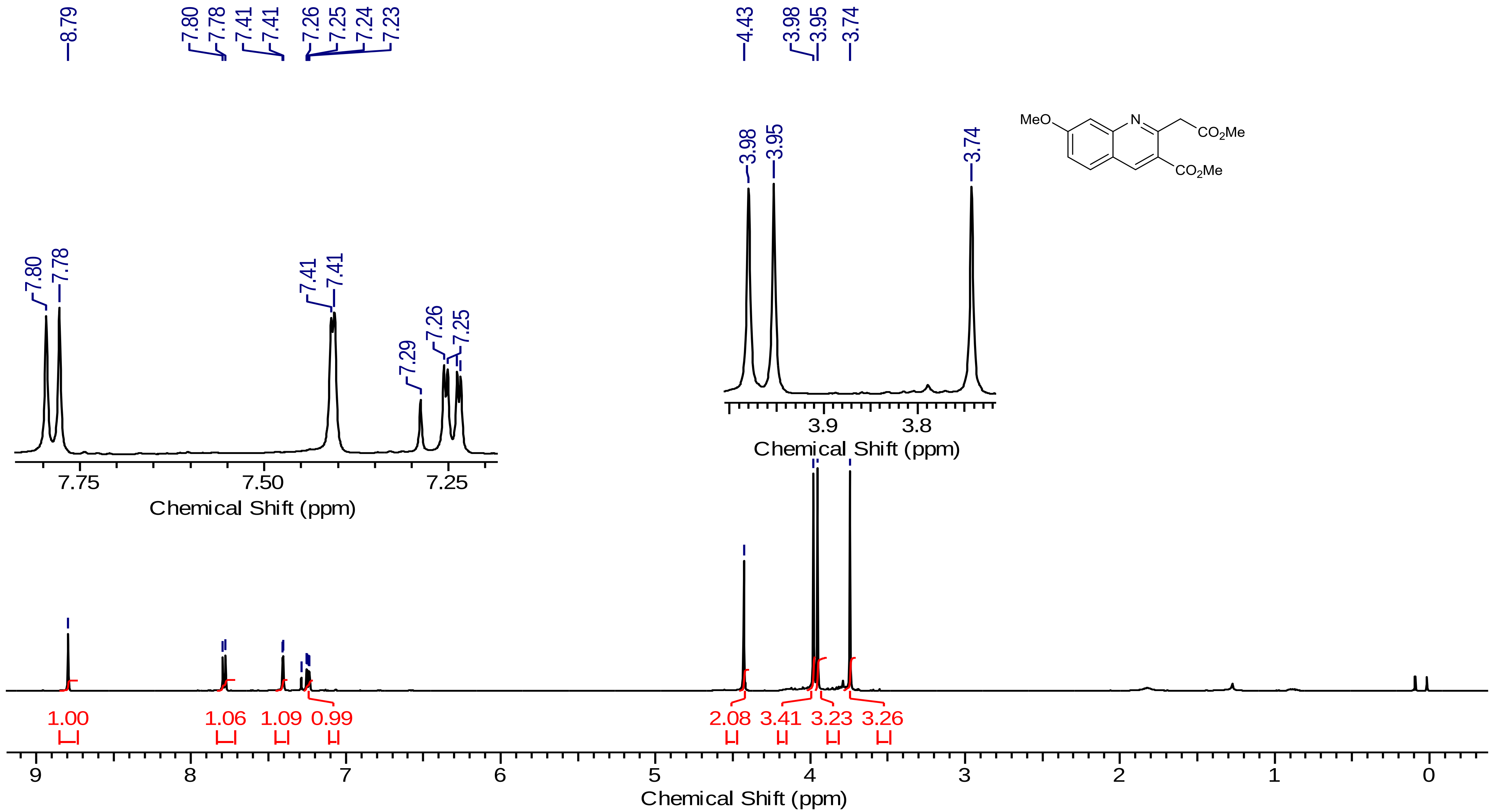

Supplementary Figure 86. ^{1}H NMR of **9**

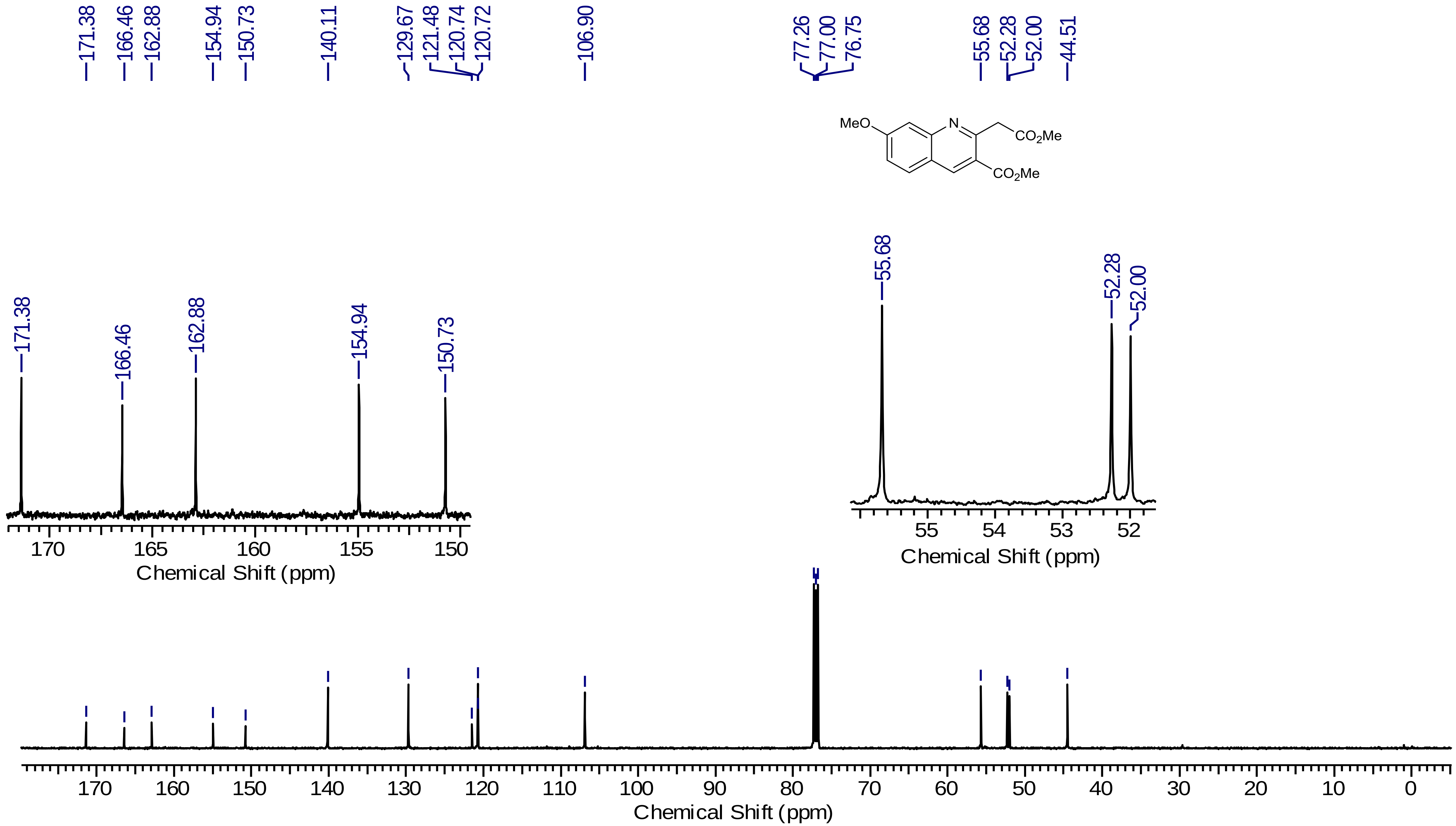

Supplementary Figure 87. ^{13}C NMR of **9**

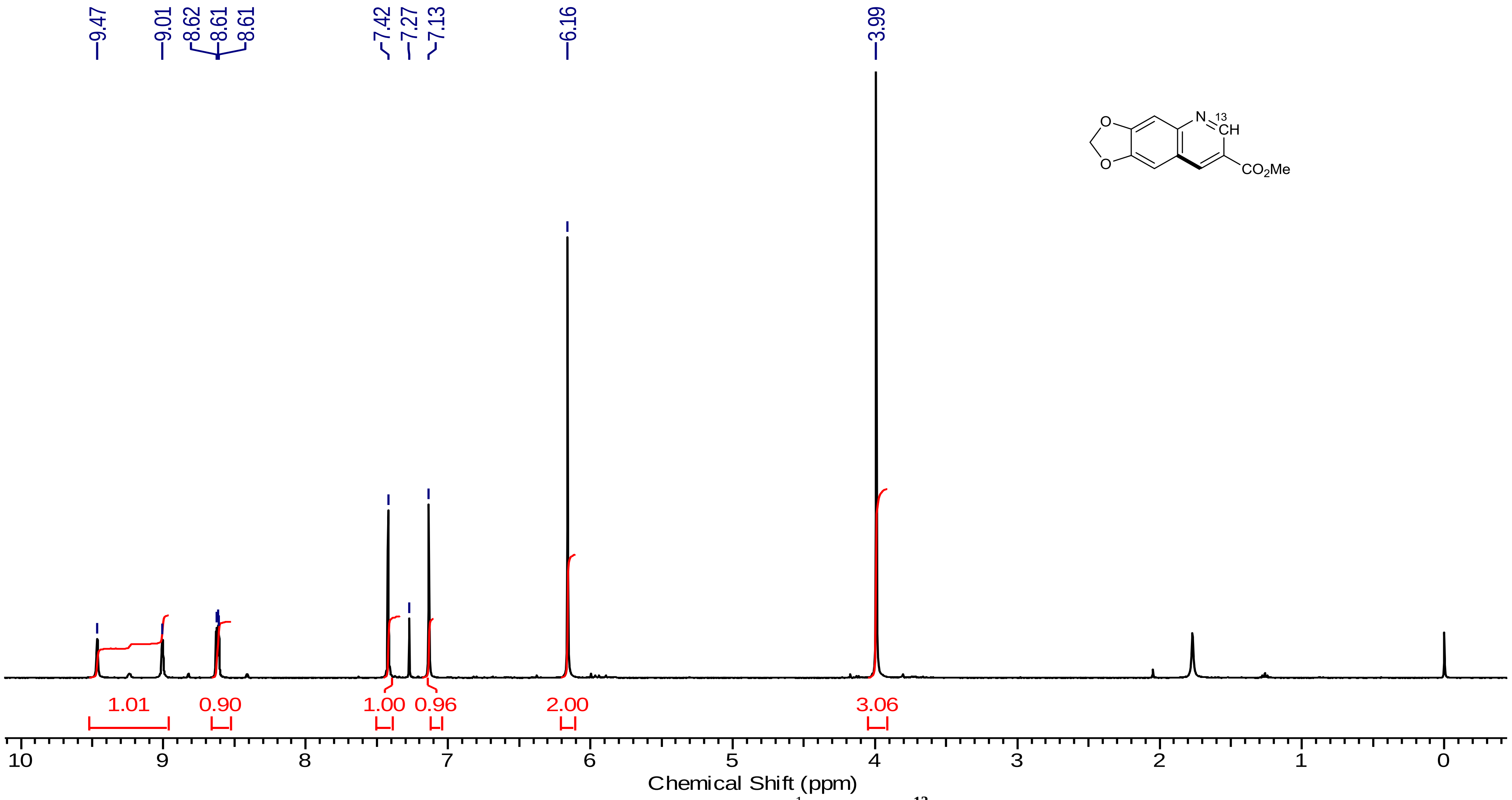

Supplementary Figure 88. ^{1}H NMR of **[^{13}C]4a**

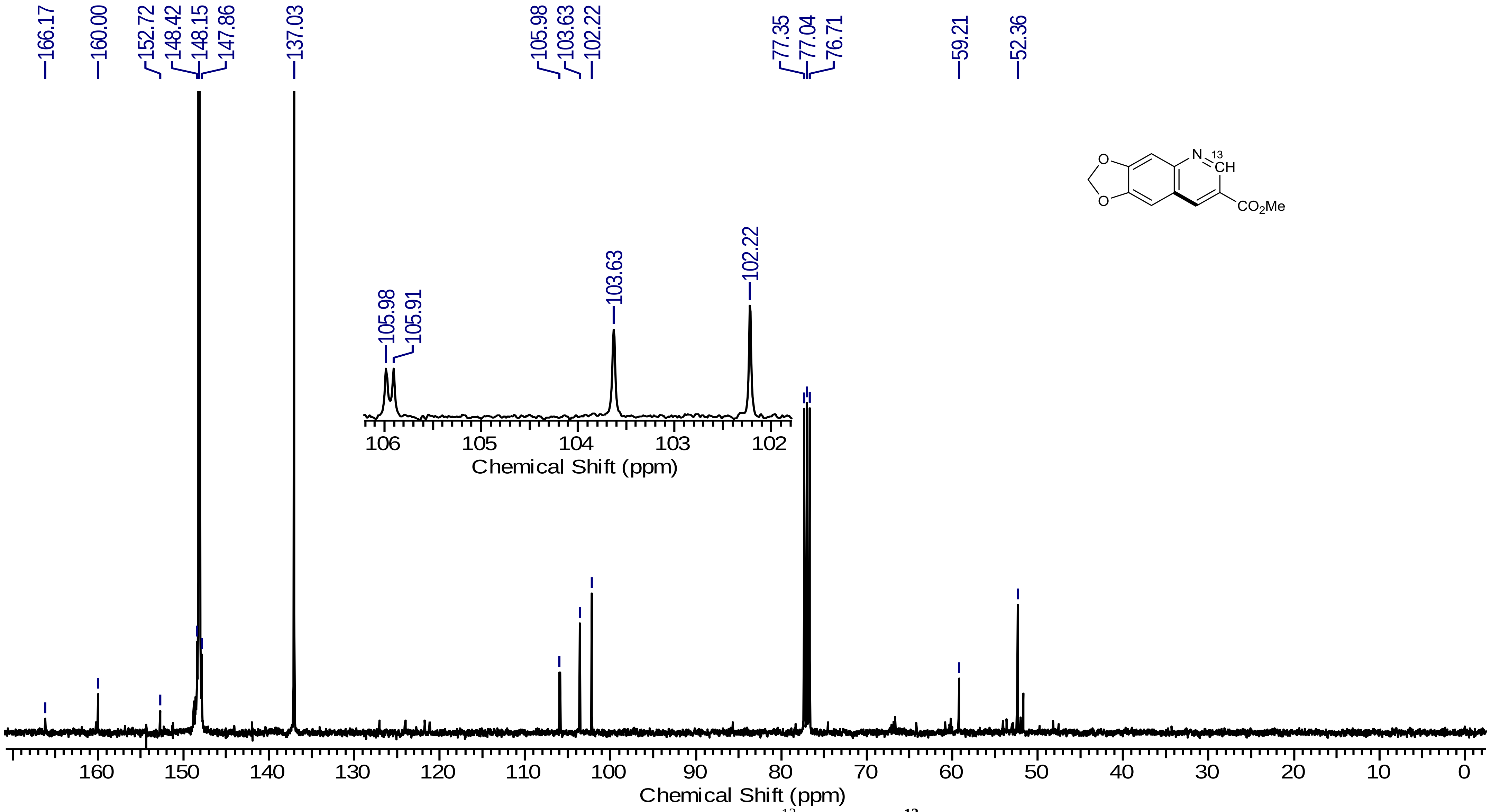

Supplementary Figure 89. ^{13}C NMR of **[^{13}C]4a**

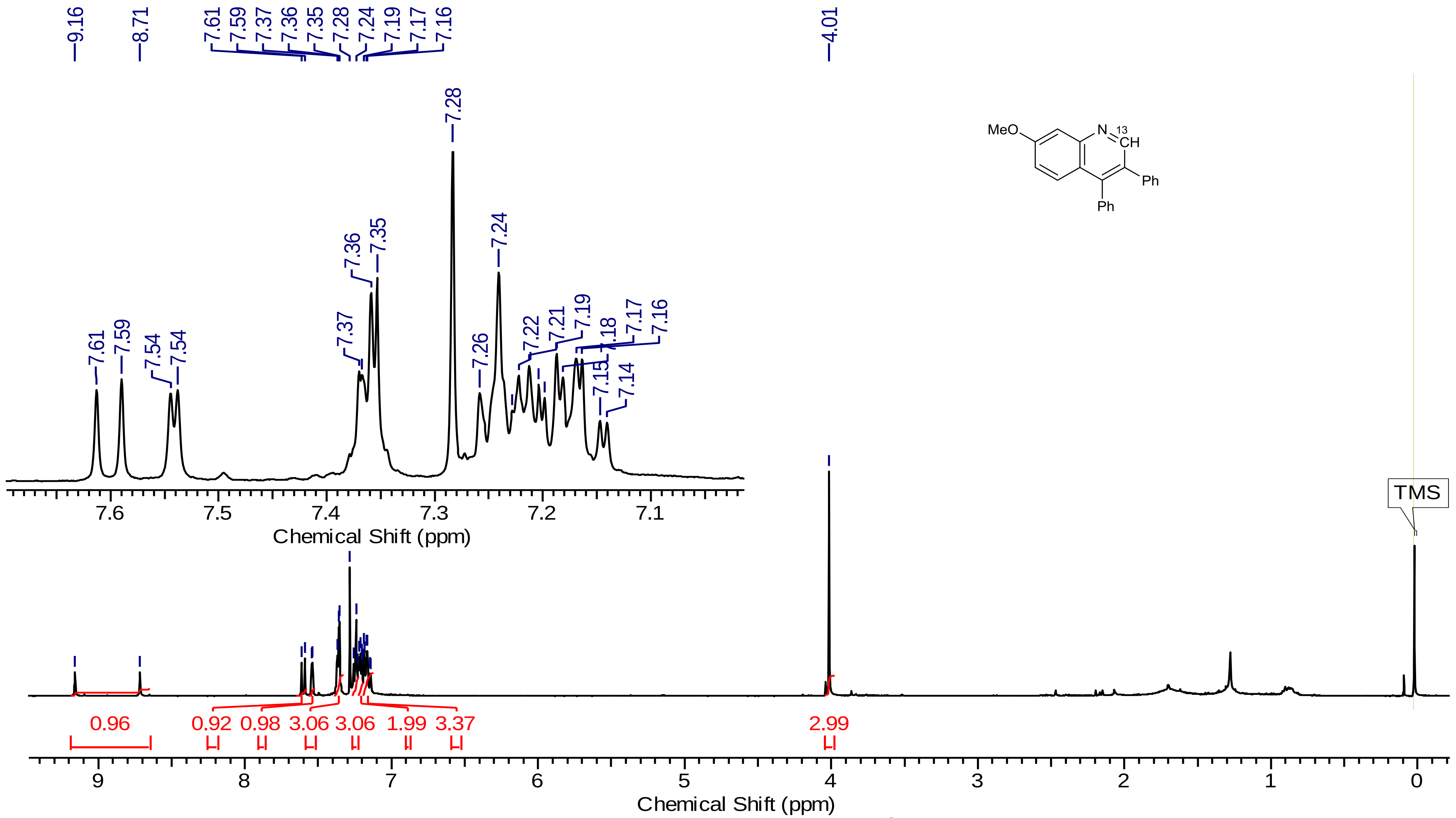

Supplementary Figure 90. ^{1}H NMR of **[^{13}C]6a**

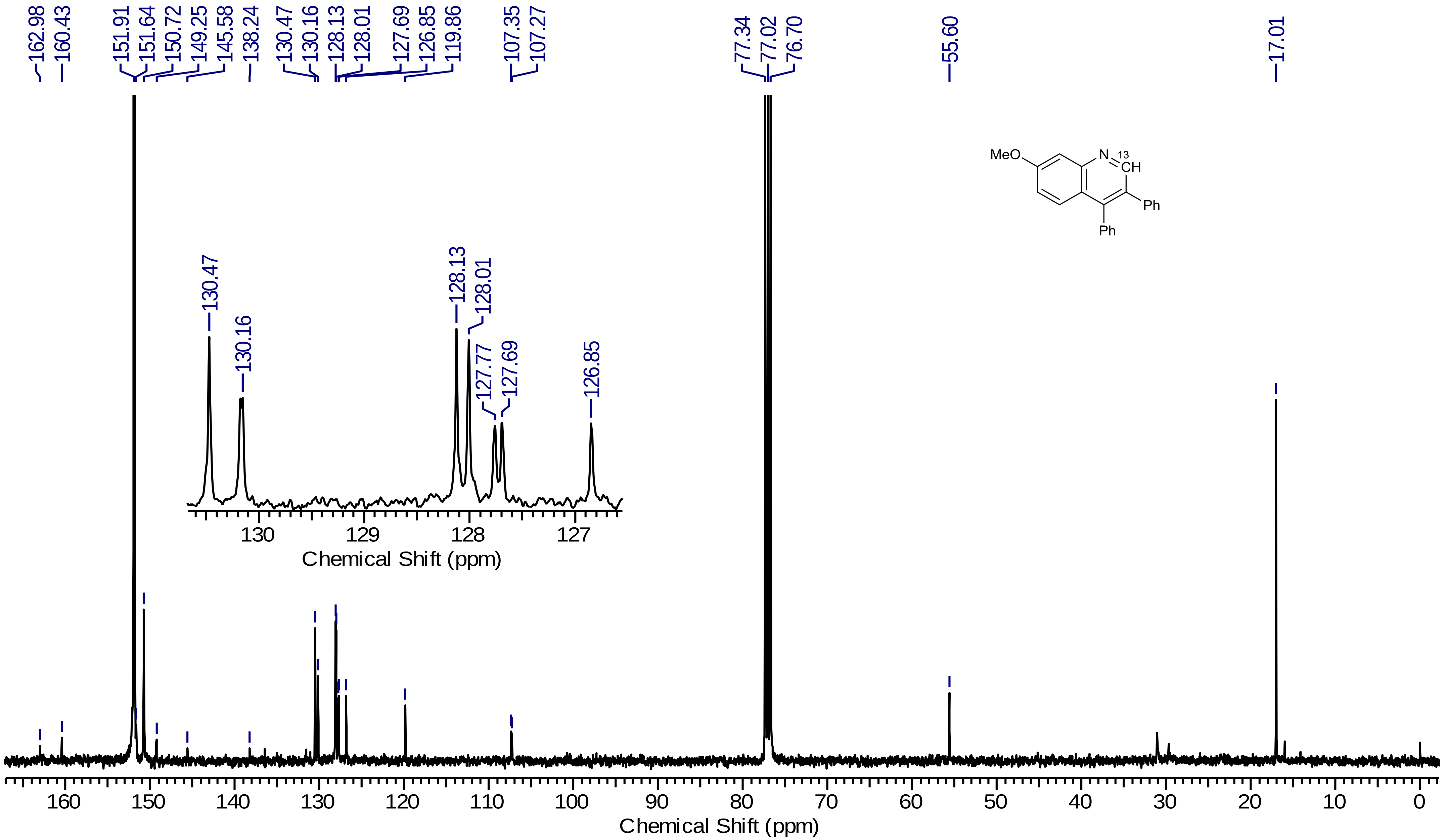

Supplementary Figure 91. ^{13}C NMR of **[^{13}C]6a**

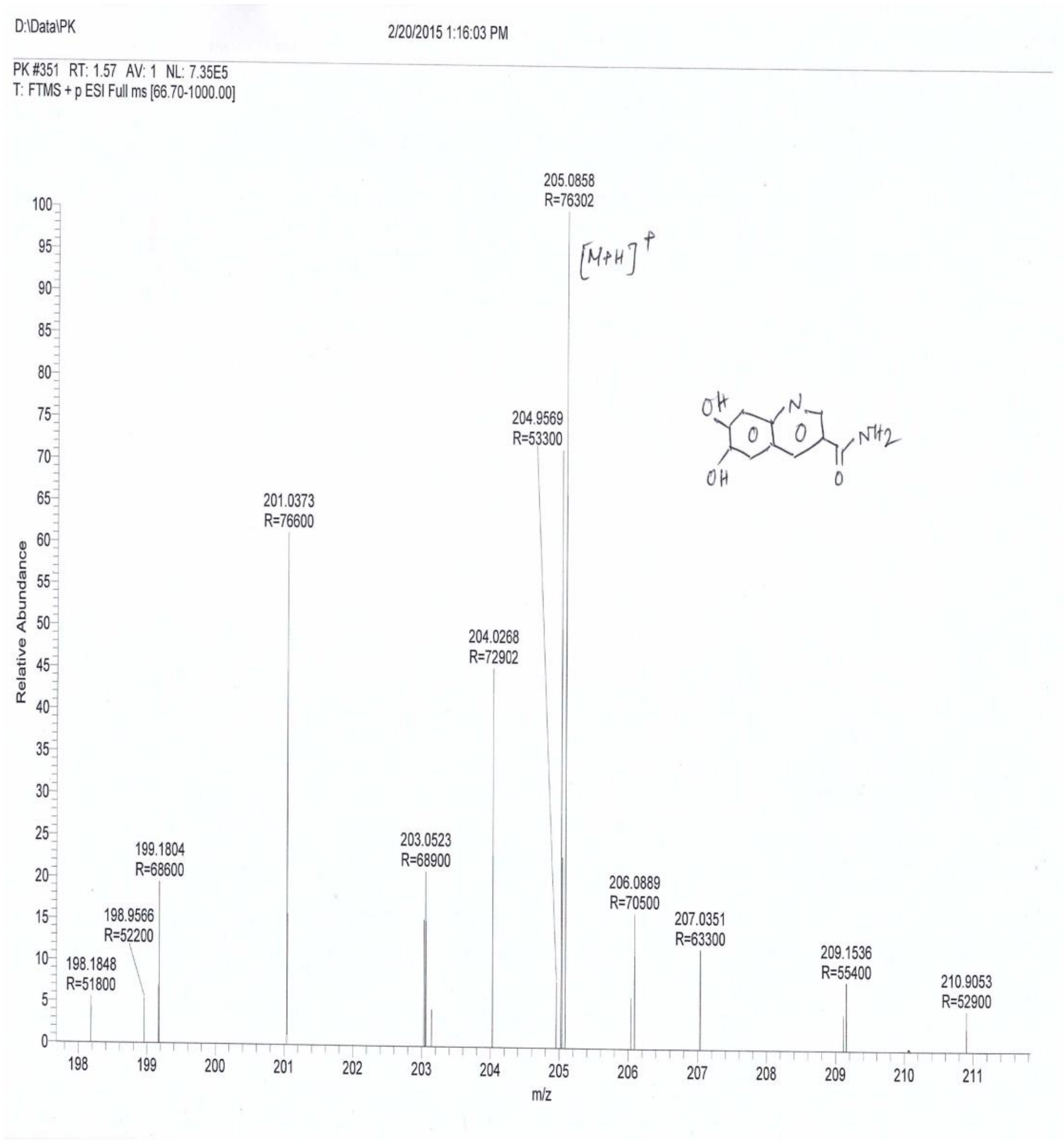

Supplementary Figure 92. HRMS spectra of **8** (reaction mixture)

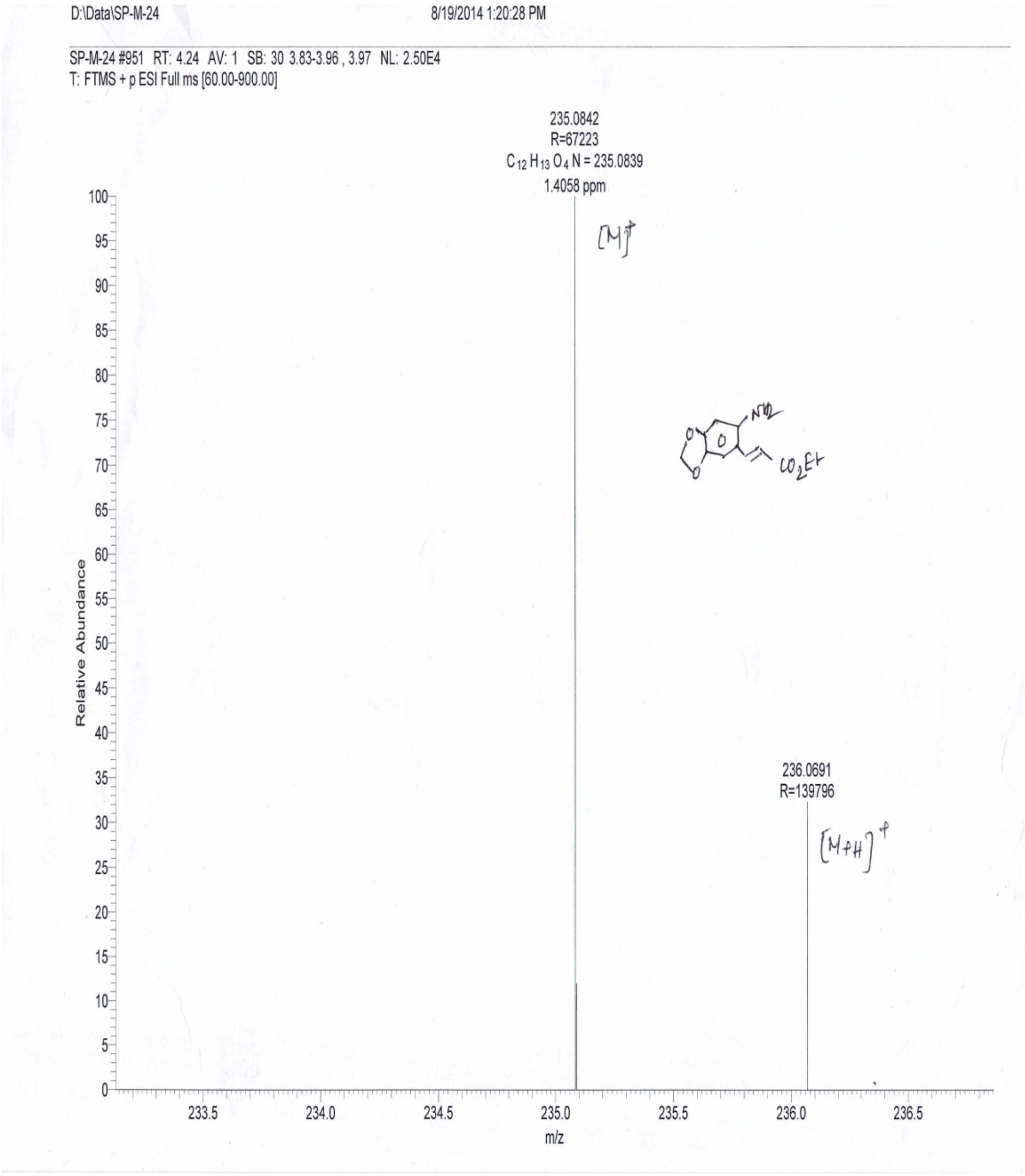

Supplementary Figure 93. HRMS spectra of **3b**

Area % Report

Data File: C:\EZChrom Elite\Enterprise\Projects\Default\Data\SANJAY\160215shiva01.dat
Method: C:\EZChrom Elite\Enterprise\Projects\Default\Method\sleep mode.met
Acquired: 2/16/2015 12:09:41 PM
Printed: 2/20/2015 12:27:14 PM

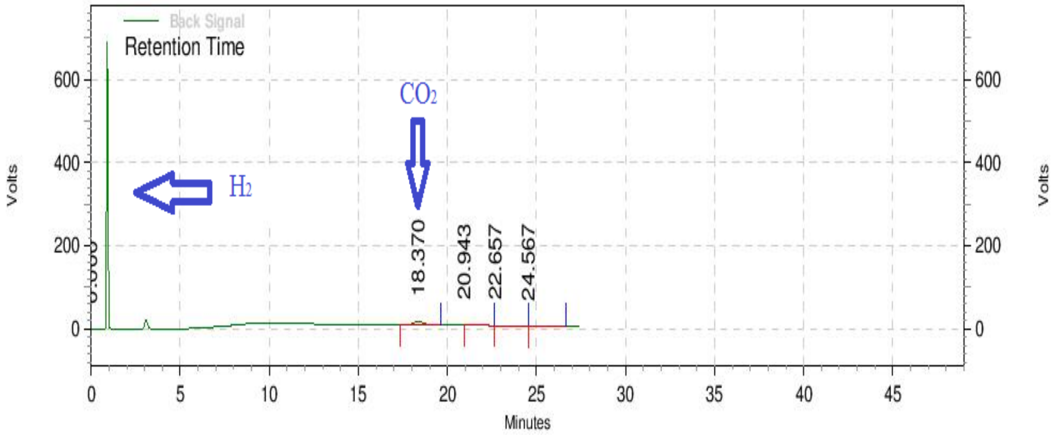

Back Signal
Results

Retention Time	Area	Area %	Height	Height %
0.000	0	0.00	0	0.00
18.370	2848121	85.97	64715	94.68
20.943	149010	4.50	238	0.35
22.657	207821	6.27	2302	3.37
24.567	108045	3.26	1096	1.60
Totals	3312997	100.00	68351	100.00

Supplementary Figure 94. GC Chromatogram for Fig. 8e

```
File        :D:\NCL\DATA\OCD\Year_2015\150343-1.D
Operator    : Dr. BORIKAR
Acquired    : 23 Feb 2015  12:50     using AcqMethod GENERAL.M
Instrument  :    GCMSD
Sample Name:
Misc Info   : CO-A
Vial Number: 0
```

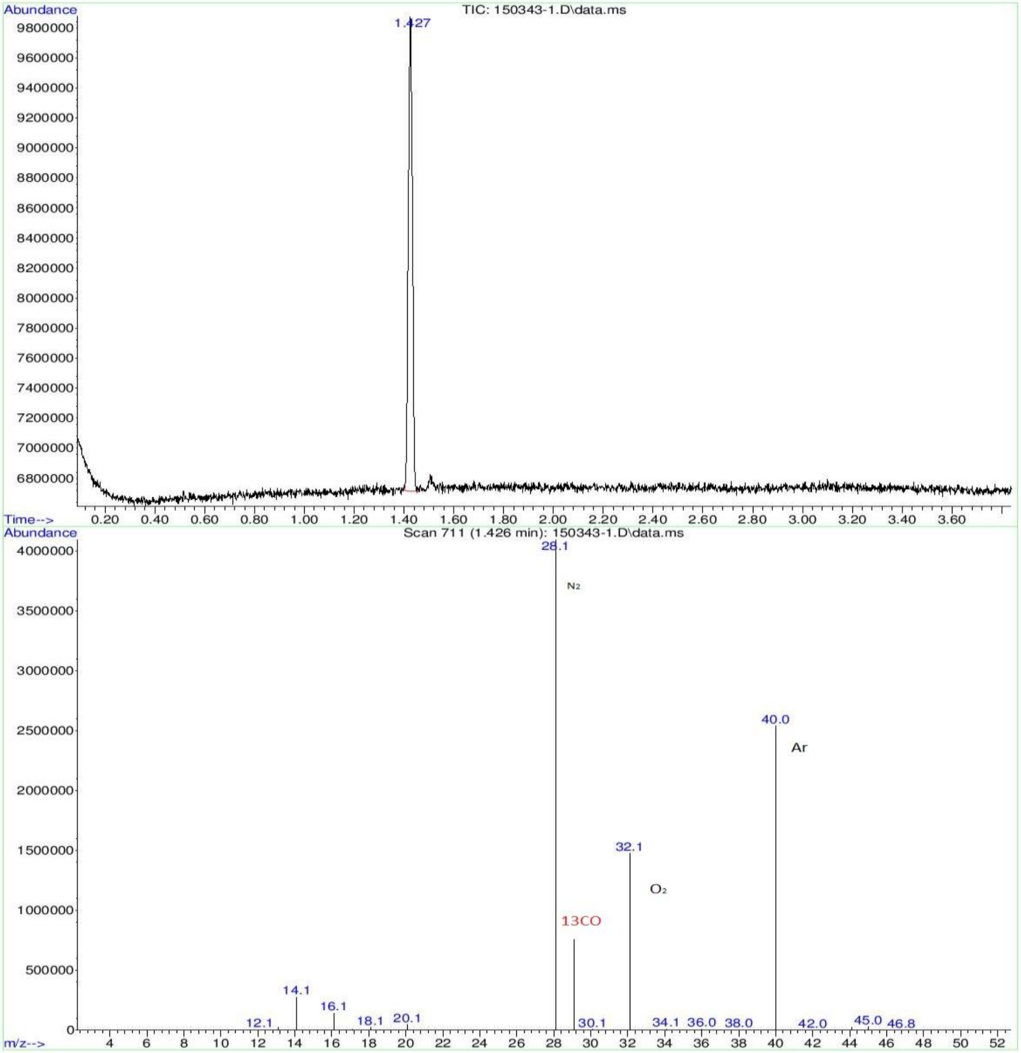

Supplementary Figure 95. GC-MS Chromatogram for Fig. 8e

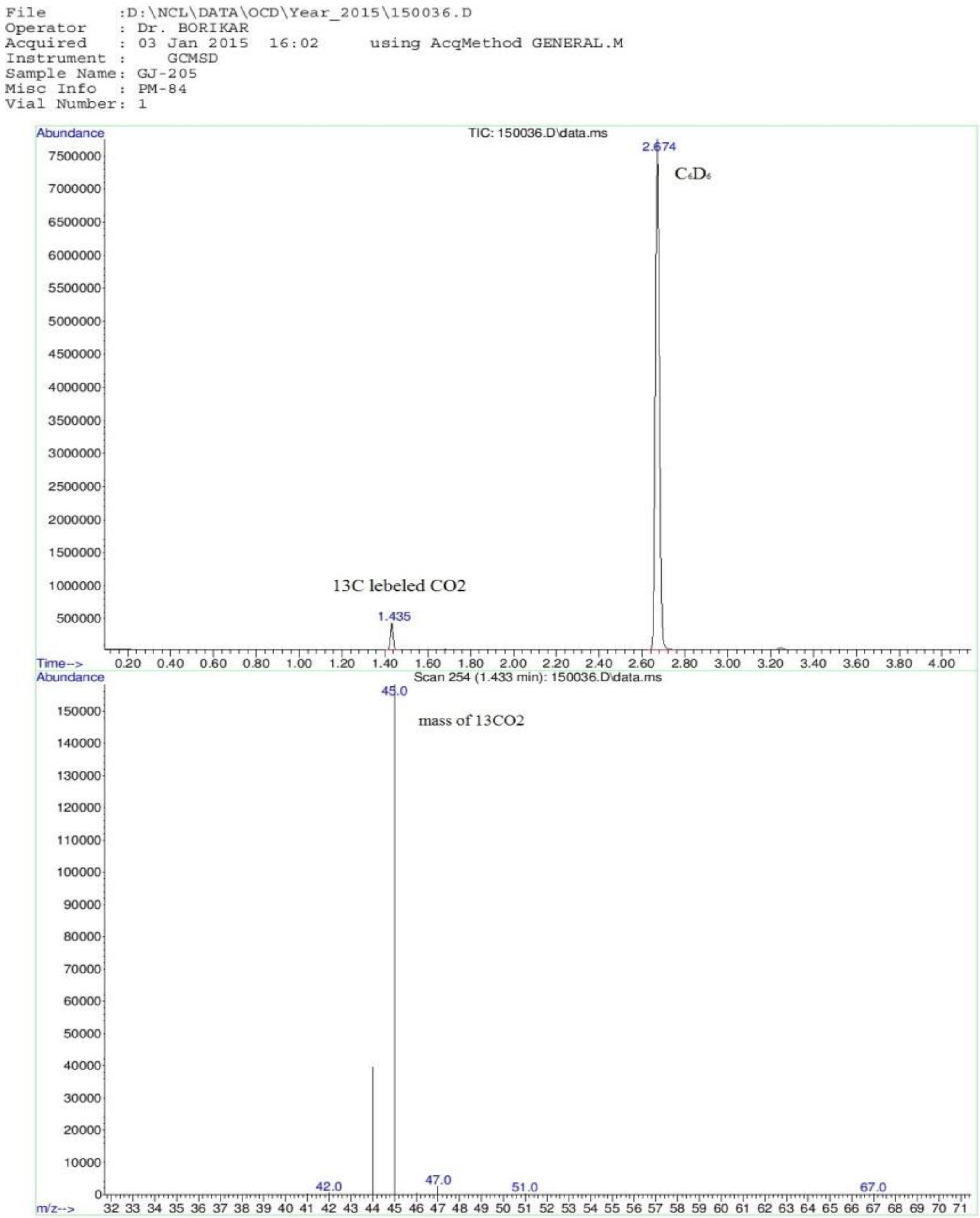

Supplementary Figure 96. GC Chromatogram for Fig. 8e (reaction in water)

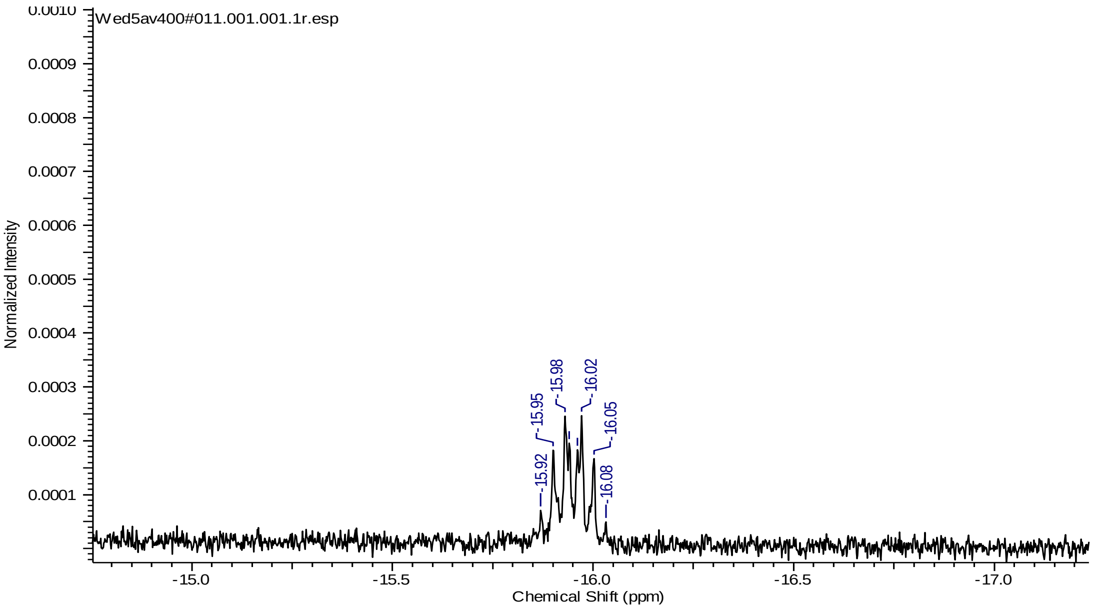

Supplementary Figure 97. ^{1}H NMR of Rh-H intermediate (shown only the hydride region)

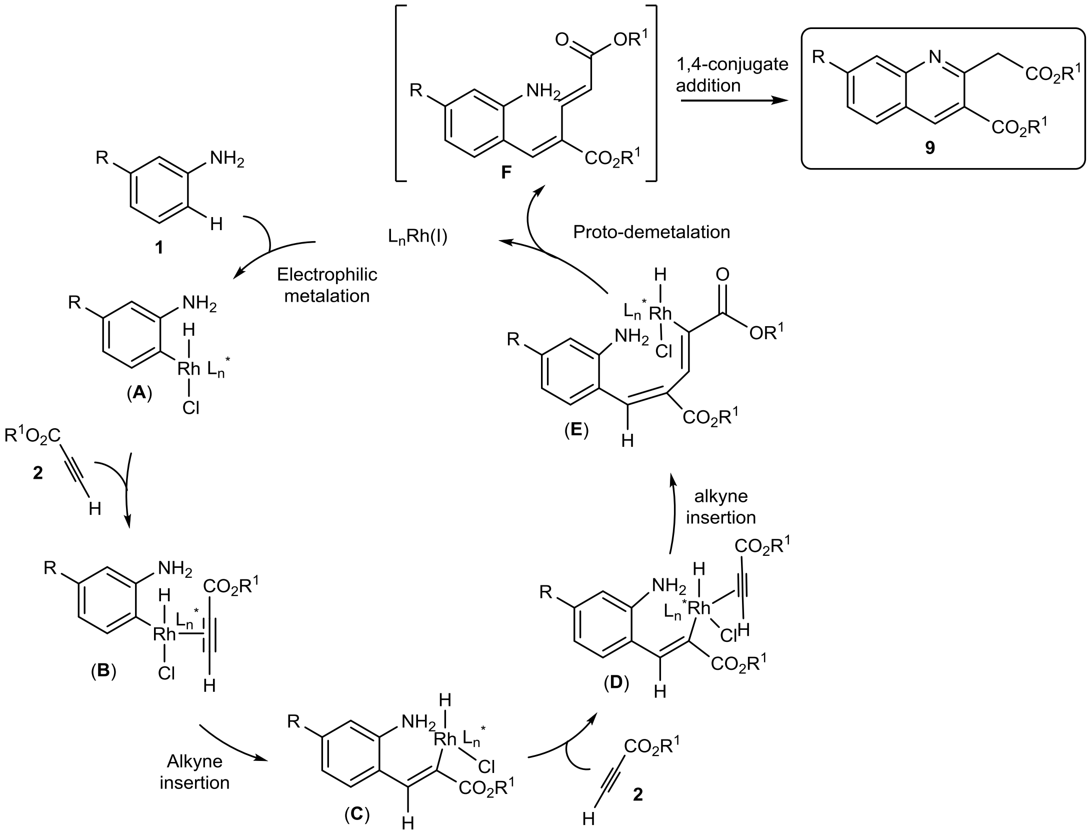

Supplementary Figure 98. A plausible mechanism for the formation of **9**

Supplementary Table 1: Screening of CO surrogate

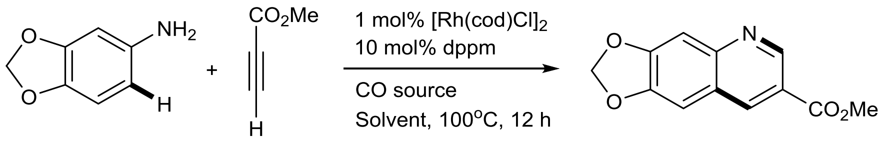

1a 2a 4a

Entry	CO source	Solvent	Yield(%)[a]
1.	CO/H_2 (1:1)	H_2O	18
2.	CO/H_2 (1:1)	THF	11
3.	$(HCHO)_n$	THF	NR[b]
4.	$(HCHO)_n$	THF	62[c,d]
5.	$(HCHO)_n$	H_2O	7[b]
6.	$(HCHO)_n$	DMSO	71[d,e]
7.	$(HCHO)_n$	Toluene	NR[e]

Procedure: 3,4-(Methylenedioxy)aniline **1a** (0.1 mmol), methyl propiolate **2a** (0.11 mmol), $[Rh(cod)Cl]_2$ (1 mol%), dppm (10 mol%), CO source (0.25 mmol in case of HCHO or 3 atm of syngas), 50 µL of solvent were heated at 100 °C in a closed viol for 12 h. [a]Yields of quinoline were determined by ^{1}H NMR using toluene as an internal standard. [b]At room temperature for 24 h. [c]Commercially available (AR grade) THF was used as a solvent. [d]Isolated yields. [e]50 µL of solvent (10:1 mixture of solvent and water).

Supplementary Table 2: Screening of rhodium catalyst

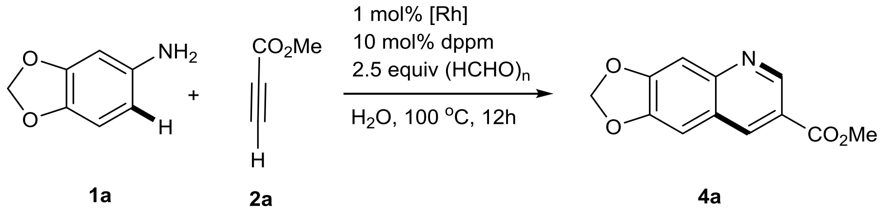

**Entry**	**[Rh] cat.**	**Yield(%)**[a]
1.	$[Rh(coe)_2Cl]_2$	49
2.	$[Cp^*RhCl_2]_2$	18
3.	$Rh_2(OAc)_4$	37
4.	$Ru_3(CO)_{12}$	NR
5.	$RhCl_3.3H_2O$	23
6.	$RhCl(PPh_3)_3$	29
7.	$[Rh(nbd)Cl]_2$	78 (65)[b]

Procedure: 3,4-(methylenedioxy)aniline **1a** (0.1 mmol), methyl propiolate **2a** (0.11 mmol), Rh catalyst (1 mol%), dppm (10 mol%), paraformaldehyde (0.25 mmol) and 50 µL of H_2O were heated at 100 °C in a closed vial for 12 h. [a]Yields of quinoline were determined by ^1H NMR using toluene as an internal standard. [b]Isolated yields.

Supplementary Table 3: Screening of ligand

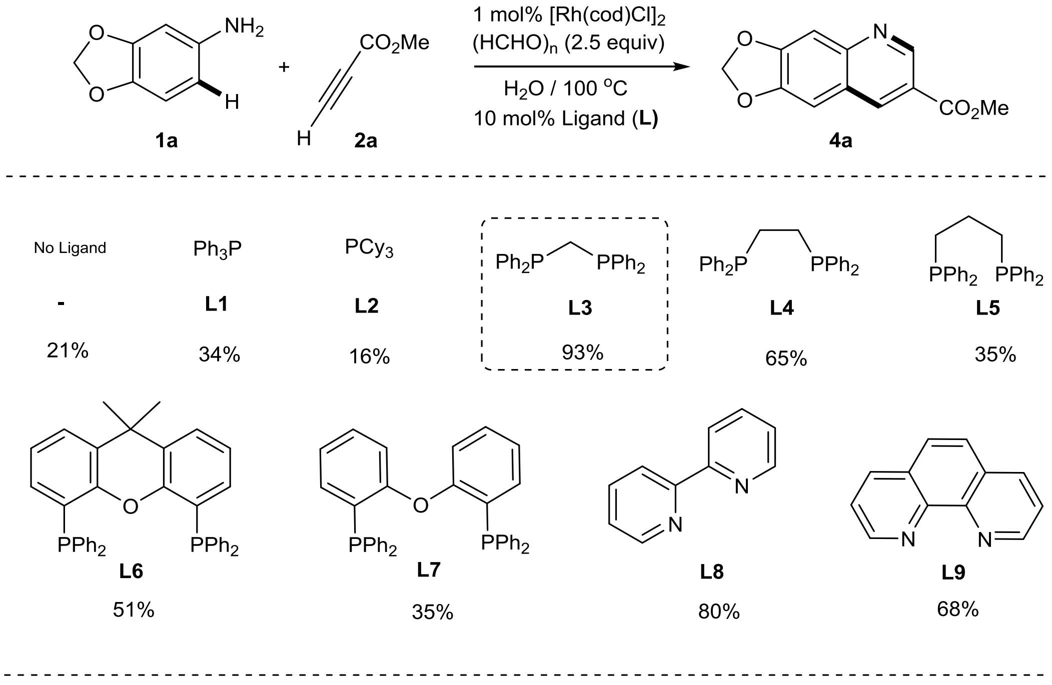

Procedure: 3,4-(Methylenedioxy)aniline **1a** (0.1 mmol), methyl propiolate **2a** (0.11 mmol), $[Rh(cod)Cl]_2$(1 mol%), ligand **L** (10 mol%), $(HCHO)_n$ (0.25 mmol), 50 μL of H_2O were heated at 100 ^{o}C in a closed viol for 12 h and the depicted yields are isolated yields.

When we performed this auto-tandem reaction in the absence of ligand, the reaction proceeded with a lower yield (21%, Supplementary Table 3). Monodentate phosphine ligands showed poorer reactivity and lower yields of the product were obtained (34% for PPh_3 (**L1**), and 16% for PCy_3 (**L2**)) over bidentate ligands. This result indicates the lower ability of monodentate ligands to faster the reductive elimination step in the catalytic cycle. In contrast, bidentate ligand with the increase of their bite angle, the yield of **4a** substantially decreased from 93% to 35% (**L3 - L7**). Under the similar catalytic conditions, nitrogen based bidentate ligands such as 2,2’-bipyridine (**L8)** and 1,10-phenanthroline (**L9)** yielded 80% and 68% of **4a** respectively.

Supplementary Table 4: Screening of rhodium catalyst loading

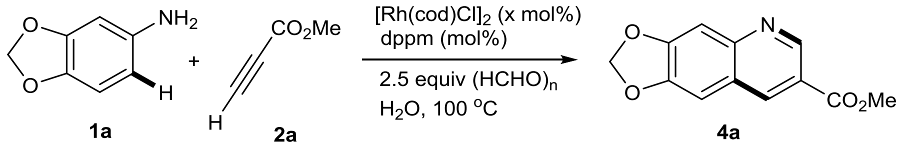

Entry	dppm(mol%)	Yield (%)[a]
1.	0.1	11
2.	0.5	61
3.	1	93
4.	2.5	95
5.	5	73

Procedure: 3,4-(Methylenedioxy)aniline **1a** (0.1 mmol), methyl propiolate **2a** (0.11 mmol), [Rh(cod)Cl]$_2$ (x mol%), dppm (10 mol%), (HCHO)$_n$ (0.25 mmol) and 50 µL of H_2O were heated at 100 ^{o}C in a closed viol for 12 h. [a]Isolated yields.

From the Supplementary Table 4, it is observed that 5 fold excess of ligand (per rhodium atom; 2.5 mol% of Rh atom) is required to obtain excellent yield of the products. This is because in the catalytic reaction water was produced as the by-product and there is a possibility of formation of phosphine oxide (observed by ^{31}P NMR in few cases). Thus, we can hardly believe that 10 mol% of phosphine liagnd (5 fold excess w.r.t per Rh atom) is required for the effective catalytic transformation.

Supplementary Table 5: Screening of ligand loading

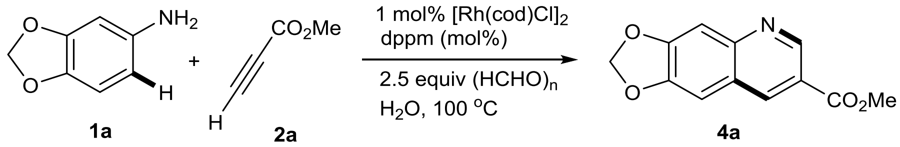

Entry	dppm(mol%)	Yield (%)[a]
1.	2	47
2.	5	69
3.	10	93
4.	20	66
5.	30	42

Procedure: 3,4-(Methylenedioxy)aniline **1a** (0.1 mmol), methyl propiolate **2a** (0.11 mmol), $[Rh(cod)Cl]_2$ (1 mol%), dppm (x mol%), $(HCHO)_n$ (0.25 mmol) and 50 µL of H_2O were heated at 100 °C in a closed viol for 12 h. [a]Isolated yields.

Supplementary Table 6: Screening of temperature

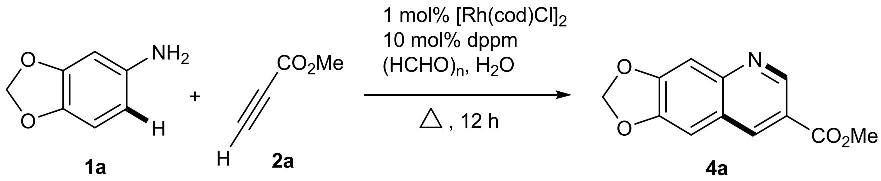

Entry	Temperature(°C)	Yield(%)[a]
1.	RT	11[b,c](85)
2.	RT	7[c]
3.	50	44 (50)
4.	70	55 (34)
5.	100	93

Procedure: 3,4-(Methylenedioxy)aniline **1a** (0.1 mmol), methyl propiolate **2a** (0.11 mmol), $[Rh(cod)Cl]_2$ (1 mol%), dppm (10 mol%), $(HCHO)_n$ (0.25 mmol) and 50 µL of H_2O were heated at specified temperature (^oC) in a closed viol for 12 h. [a]Isolated yields and yields in the parenthesis are recovery of **1a**. [b]Aq. formaldehyde used as a CO source. [c]24 h.

Supplementary Methods

General Information

All catalytic experiments were carried out using standard Schlenk techniques or using J. Young NMR tubes. All solvents were reagent grade or better. Deuterated solvents were used as received. All non-deutrated solvents were dried according to standard procedure.[1] Most of the starting materials (anilines and alkynes), and ligands used in catalytic reactions were purified according to standard procedure.[2] All the reactions were performed in normal reaction tube received from the Fischer brand. Thin layer chromatography (TLC) was performed on Merck 1.05554 aluminum sheets precoated with silica gel 60 F254 and the spots visualized with UV light at 254 nm or under iodine. Column chromatography was performed with SiO_2 (SilicycleSiliaflash F60 (230-400 mesh)). ^1H and ^{13}C spectra were recorded on Bruker DRX-200 (200MHz), DRX-400 (400MHz) and DRX-500 (500MHz) spectrometers with tetramethylsilane or $CHCl_3$ as an internal standard. The peaks were internally referenced to TMS (0.00 ppm) or residual undeuterated solvent signal (77.16 ppm for ^{13}C NMR). Abbreviations used in the NMR follow-up experiments: br, broad; s, singlet; d, doublet; t, triplet; q, quartet; m, multiplet. High resolution mass spectra (HRMS) were recorded at the Centre for Material Characterisation (CMC), CSIR-National Chemical Laboratory. GC analysis were carried out using a Carboxen 1000 column on a HP 690

series GC system or HP-5 cross linked 5% PH ME Siloxane column (30m × 0.32mm × 0.25 µm film thickness, FID) on a HP 6890 series GC system. GC-MS was carried out on HP 6890 (flame ionization detector and thermal conductivity detector) and HP 5973 (MS detector) instruments equipped with a 30 m column (Restek 5MS, 0.32 mm internal diameter) with a 5% phenylmethylsiliconecoating (0.25 mm) and helium as carrier gas. The gaseous CO and hydrogen were analyzed by GC-TCD with a Carbon plot capillary column (J&W Scientific).

Synthesis of Starting Materials

Synthesis of **1h and 1i**

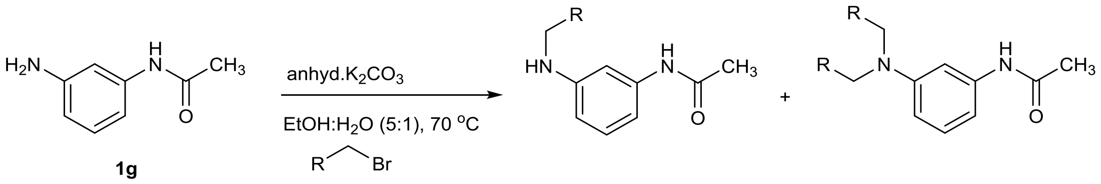

Procedure (Method A): A mixture of **1g** (5 mmol), allyl or propargyl bromide (15 mmol), K_2CO_3 (20 mmol), EtOH (25 mL), and water (5 mL) was added to a 100 mL round bottom flask and stirred at 70°C for the desired time until complete consumption of **1g** as judged by TLC. Then, the solvent was removed under reduced pressure and the product was extracted with ethyl acetate (10 mL × 3), and the combined organic layers were dried over anhydrous Na_2SO_4. The solvent was removed by evaporation under reduced pressure to afford the crude products of both mono- and bis-alkylated derivatives of **1g**. The crude products were subjected for hydrolysis of acetyl group without further purification.

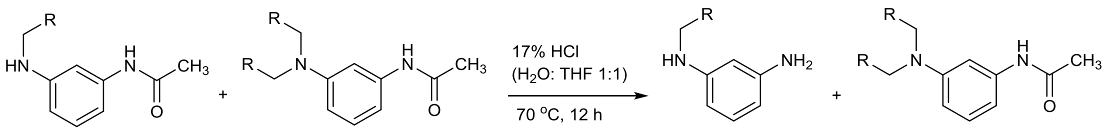

Procedure (Method B): To a 15 mL oven dried screw capped tube were added alkylated products of **1g** and 17% of HCl (H_2O:THF = 1:1by v/v) at room temperature. This reaction mixture was heated to 70°C for 15 h. After cooling to room temperature the reaction mixture was neutralised with saturated $NaHCO_3$ followed by extraction with ethyl acetate (10 mL × 3). The combined organic layers were dried over anhydrous Na_2SO_4 and the solvent was removed by evaporation under reduced pressure. The crude products were isolated by silica gel column chromatography using petroleum ether and ethyl acetate as an eluent.

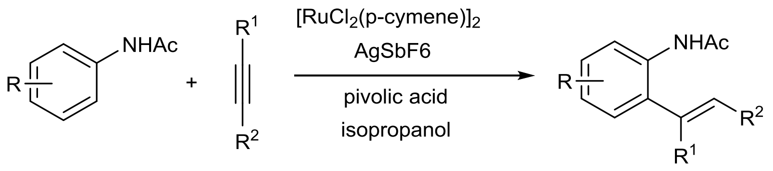

Procedure (Method C):[3] In a 15 mL oven dry screw cap tube [$RuCl_2$(*p*-cymene)]$_2$ (0.25 mmol, 5 mol%) and $AgSbF_6$ (1 mmol, 20 mol%) were added under argon atmosphere. To that tube acetanilide (5 mmol), alkyne (5.5 mmol), pivolic acid (25 mmol, 5 equiv) and isopropanol (2.5 mL) were added. After that the reaction mixture was heated up to 100 ^{o}C for 12 h. Then the reaction mixture was cooling to room temperature and diluted by dichloromethane. Diluted reaction mixture then passed through celite pad and concentrated on rotavapor under reduce pressure. The crude product was purified through silica gel column chromatography by using pet ether and ethyl acetate as an eluent.

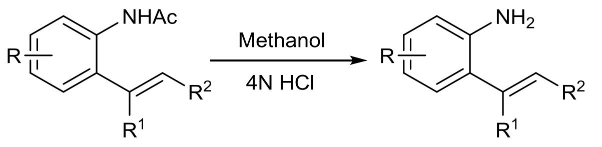

Procedure: Method-B was followed for hydrolysis of acetanilides (**5a'-5g'**) to get *ortho*- alkenylated anilines (**5a-5g**).

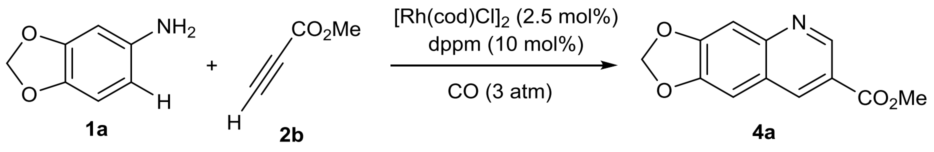

Procedure: A 100 mL Fischer-Porter tube was charged under nitrogen with $[Rh(cod)Cl]_2$ (2.5 mol%), dppm (10 mol%), **1a** (0.1 mmol), methyl propiolate **2a** (0.11 mmol), and 250 µL of solvent (THF or water). The Fischer-Porter tube was purged by three successive cycles of pressurization/venting with CO (5 psi), then pressurized with CO (3 atm). The solution was heated at 100 ºC with stirring for 12 h. After cooling to ~5 ^{o}C (ice/water), the excess CO was vented carefully and the reaction mixture was diluted with water (6 mL) and extracted with ethyl acetate (3 x 5 mL). The combined organic layer was dried over anhydrous Na_2SO_4 and the solvent was evaporated. The crude product was purified by silica gel column chromatography (230-400 mesh size) using petroleum-ether/ethyl acetate as an eluent.

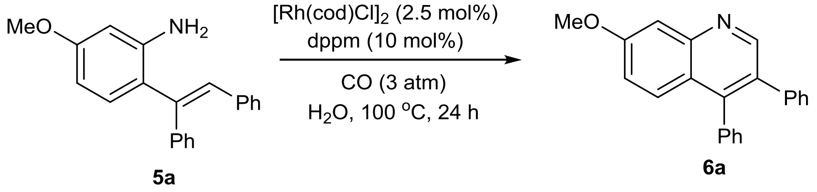

Procedure: A 100 mL Fischer-Porter tube was charged under nitrogen with $[Rh(cod)Cl]_2$ (2.5 mol%), dppm (10 mol%), **5a** (0.25 mmol), and water (500 µL). The

Fischer-Porter tube was purged by three successive cycles of pressurization/venting with CO (5 psi), then pressurized with CO (3atm). The solution was heated at 100 ºC with stirring for 24 h. After cooling to ~5 °C (ice/water), the excess CO was vented carefully and the reaction mixture was diluted with water (6 mL) and extracted with ethyl acetate (3 x 5 mL). The combined organic layer was dried over anhydrous Na_2SO_4 and the solvent was evaporated. The crude product was purified by silica gel column chromatography (230-400 mesh size) using petroleum-ether/ethyl acetate as an eluent.

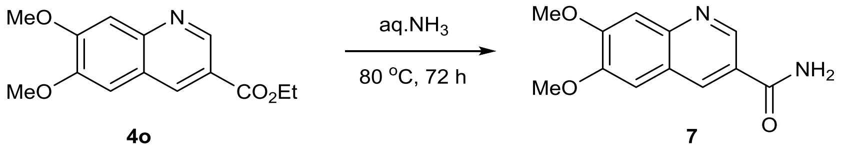

Procedure: To an oven dried 15 mL screw cap reaction vial were added **4o** (264 mg, 1 mmol), NH_4Cl (53 mg, 1 mmol) followed by a saturated solution of NH_3 in methanol. The reaction mixture was heated at 70 °C for 72 h. After cooling to ambient temperature and triturated with water to get analytically pure compound **7**.[4]

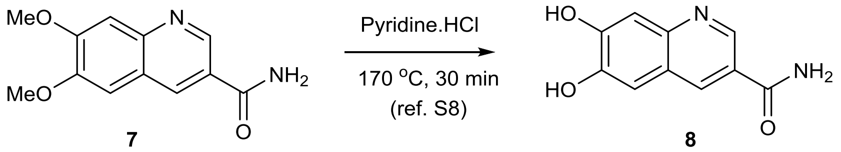

Procedure: To an oven dried 25 mL round bottle flask 87 mg of 6,7-dimethoxyquinoline-3-carboxamide (**7**) and 850 mg of pyridine.HCl were added and then the reaction mixture was kept for heating at 170 °C for 30 minutes. After heating the excess amount of pyridine.HCl was removed under high vacuum and the resulting

solid was washed with ice cold water. The crude mixture was subjected for HRMS analysis (Supplementary Fig 92) to confirm the formation of 6,7-Dihydroxyquinoline-3-carboxamide (**8**).

Mechanistic Studies

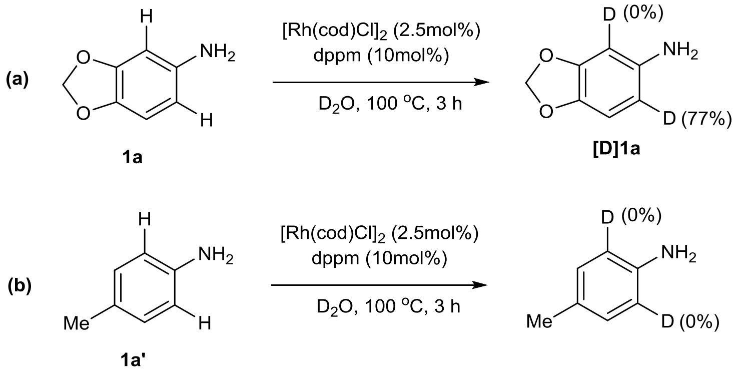

Procedure: To a J.YOUNG NMR tube were added aniline **1a** or **1a'** (0.2 mmol), $[Rh(cod)Cl]_2$ (2.5 mol%), dppm (10 mol%), and D_2O (400 µL) under argon atm. The reaction mixture was heated at 100 °C for 3 h. After cooling to room temperature the ^{1}H NMR confirmed the formation of *ortho*-deuterated product of **1a** with high region control while in the case of **1a'** no deuteration was observed. The residue was purified by column chromatography on silica gel (eluent: pet ether/EtOAc) to afford the desired product **[D]1a**.

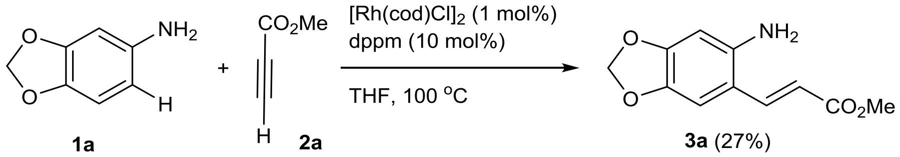

1a **2a** **3a** (27%)

Procedure: To a 10 mL clean, oven-dried screw cap reaction tube was added **1a** (0.25 mmol), $[Rh(cod)Cl]_2$ (1 mol%), dppm (10 mol%), methyl propiolate (**2a**), and THF (250 µL; 10:1 mixture of THF:H_2O) under argon atm. The reaction mixture was kept for heating at 100 °C for 3 h. After cooling to room temperature the product was extracted with EtOAc (3 x 5 mL) and the combined organic layer was dried over anhydrous Na_2SO_4 and concentrated under vacuum. Then the crude product was purified through silica gel column chromatography (230-400 mesh size) by using petroleum-ether/ethyl acetate as an eluting system and yielded 27% of **3a** with the recovery of **1a** (49%).

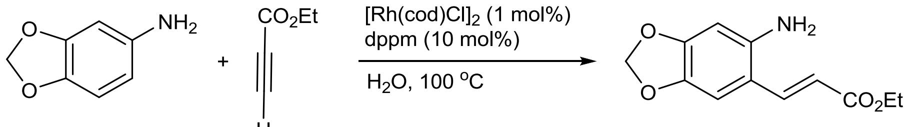

1a **2b** **3b** (identified by HRMS)

Procedure: To a 10 mL clean, oven-dried screw cap reaction tube was added **1a** (0.25 mmol), $[Rh(cod)Cl]_2$ (1 mol%), dppm (10mol%), ethyl propiolate (**2b**), and 250 µL of H_2O under argon atm. The reaction mixture was kept for heating at 100 °C for 3 h. After cooling to room temperature, the crude mixture was subjected for HRMS study to identify the C-C coupled intermediate **3b.** HRMS shows the $[M]^+$peak at 235.0839 and $[M+H]^+$ at 236.03 (Supplementary Fig 93).

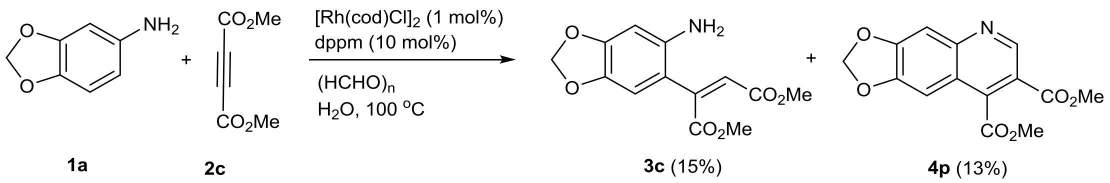

Procedure: To a 10 mL clean, oven-dried screw cap reaction tube was added $[Rh(cod)Cl]_2$ (1 mol%), dppm (10 mol%), **1a** (0.1 mmol), paraformaldehyde (2.5 equiv), dimethyl acetylenedicarboxylate (1.1 equiv) and water (250 µL) under argon atm. The reaction mixture was kept for heating at 100 ^{o}C for 12 h. After cooling at room temperature reaction mixture was diluted with water (6 mL) and extracted with ethyl acetate (3 x 5 mL). The resultant organic layer was dried over anhydrous Na_2SO_4 and the solvent was evaporated. The crude mixture was purified through silica gel column chromatography (230-400 mesh size) using petroleum-ether/ethyl acetate as an eluting system. The same reaction with prolonged time (24 h) selectively yielded **4p** with 30%.

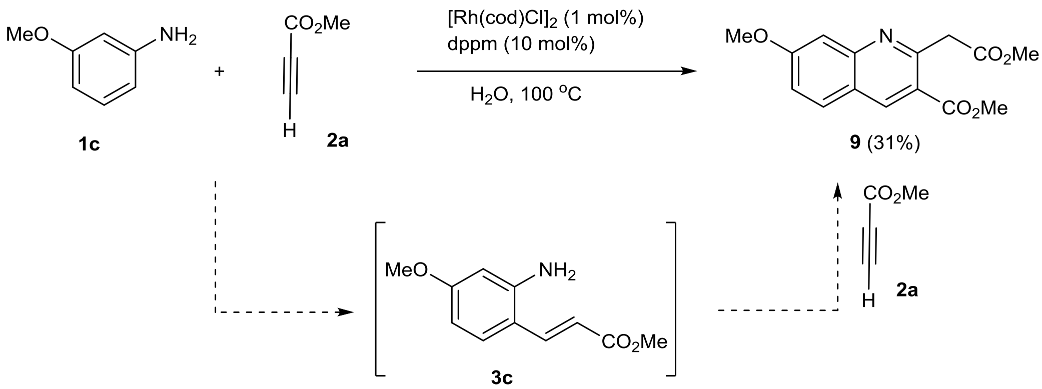

Procedure: To a 10 mL clean, oven-dried screw cap reaction tube was added **1c** (0.1 mmol), $[Rh(cod)Cl]_2$ (1 mol%), dppm (10 mol%), methyl propiolate **2a** (0.25 mmol), and H_2O (250 µL) under argon atm. The reaction mixture was kept for heating at 100 ^{o}C for 16 h. After cooling to room temperature, the reaction mixture was extracted with EtOAc (3 x 5 mL). The resultant organic layer was dried over anhydrous Na_2SO_4

and the solvent was evaporated under vacuum. Then the crude mixture was purified through silica gel column chromatography (230-400 mesh size) using petroleum-ether/ethyl acetate as an eluting system and yielded 31% of **9** with the recovery of **1c** (56%). See Supplementary Fig 98 for plausible mechanism for the formation of **9**.

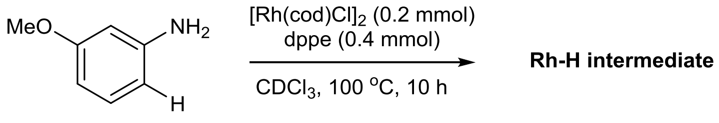

1c

Procedure: In a J.YOUNG NMR tube were added **1c** (0.4 mmol), 0.2 mmol of $[Rh(cod)Cl]_2$, 0.4 mmol of dppe, and $CDCl_3$ (0.5 mL). The mixture was heated at 100° C for about 10 h. After cooling to ambient temperature ^{1}H NMR of this reaction mixture was taken. The hydride ligand appears as a multiplet at -16 ppm ($J_{(Rh,H)}$ = 24.0, $J_{(P,H)}$ = 12.0) which anomalously proved the formation Rh-H intermediate in the reaction (Supplementary Fig 97). The hydride ligand may originated *via ortho* C-H activation of aniline (**1c**) followed by proto-demetallation.

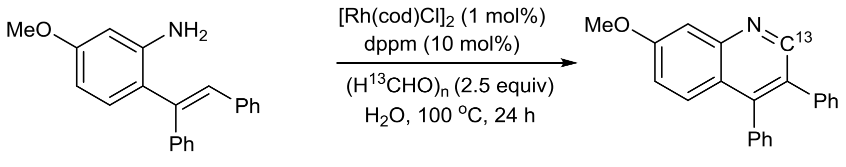

5a **[13C]6a**

Prcedure: To a 10 mL oven-dried screw cap reaction tube were added $[Rh(cod)Cl]_2$ (1 mol%), dppm (10 mol%), **5a** (0.25 mmol), ^{13}C-paraformaldehyde (0.75 mmol, 2.5 equiv) and water (250 µL) under argon atm. The reaction mixture heated at 100 ^{o}C for 24 h. After cooling to room temperature reaction mixture was diluted with water (6

mL) and extracted with ethyl acetate (3 x 5 mL). The combined organic layer was dried over anhydrous Na_2SO_4 and the solvent was evaporated. The crude product was purified by silica gel column chromatography (230-400 mesh size) using petroleum-ether/ethyl acetate as an eluent.

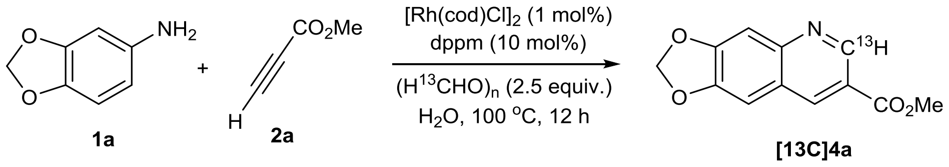

Procedure: To a 10 mL clean, oven-dried screw cap reaction tube was added $[Rh(cod)Cl]_2$ (1 mol%), dppm (10 mol%), **1a** (0.25 mmol), ^{13}C-paraformaldehyde (0.75 mmol), **2a** (0.275 mmol) and water (250 µL) under argon atm. The reaction mixture was kept for heating at 100 ^{o}C for 12 h. After cooling to room temperature reaction mixture was diluted with water (6 mL) and extracted with ethyl acetate (3 x 5 mL). The resultant organic layer was dried over anhydrous Na_2SO_4 and the solvent was evaporated under reduced pressure. The crude mixture was purified by silica gel column chromatography (230-400 mesh size) using petroleum-ether/ethyl acetate as an eluting system.

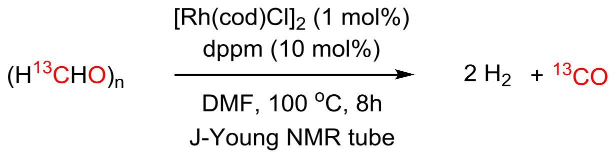

Procedure: A J. YOUNG NMR tube was charged with 0.05 mmol of $[Rh(cod)Cl]_2$, 0.1 mmol of dppm and 1 mmol of $(H^{13}CHO)_n$ and 50 µL of DMF. The NMR tube was heated at 100 ^{o}C for 8 h. After cooling to room temperature, the gaseous ^{13}CO was

qualitatively analyzed by GC-TCD with a Carbon plot capillary column (Supplementary Fig 95).

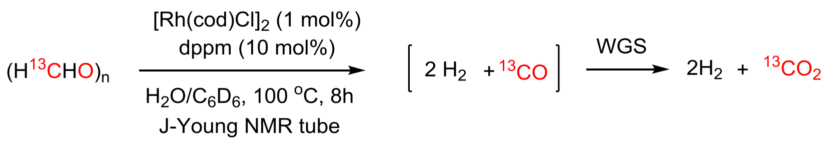

Procedure: A J. YOUNG NMR tube was charged with 0.05 mmol of $[Rh(cod)Cl]_2$, 0.1 mmol of dppm and 1 mmol of $(H^{13}CHO)_n$ and 50 µL of H_2O. The NMR tube was heated at 100 °C for 16 h. After cooling to room temperature, the gaseous samples were analysed on GC and GC-MS. Indeed, after 16 h complete formation of carbon dioxide (*via* water-gas shift reaction)[5] and dihydrogen were observed (Supplementary Fig 94 and 96).

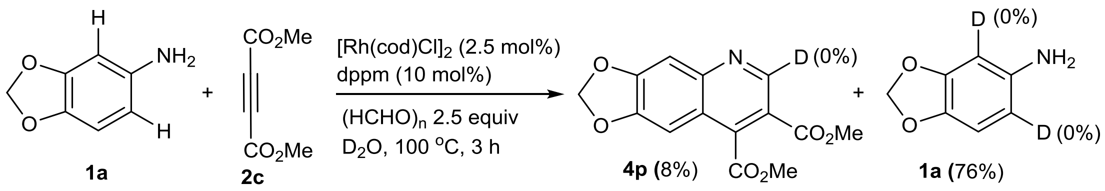

To a 10 mL clean, oven-dried screw cap reaction tube was added $[Rh(cod)Cl]_2$ (2.5 mol%), dppm (10 mol%), **1a** (0.12 mmol), paraformaldehyde (2.5 equiv), **2c** (0.13 mmol) and D_2O (0.3 mL) under argon atm. The reaction mixture was kept for heating at 100 °C for a 3 h. After cooling to room temperature, reaction mixture was extracted with ethyl acetate (3 x 5 mL) and passed through celite pad. The organic layer was concentrated under reduced vacuum. Compounds **1a** (76%) and **4p** (8%) were isolated and their deuterium content was analyzed by ^{1}H NMR. With both recovered compounds, no deuterium incorporation was observed suggesting that cyclometalation is irreversible in presence of **2c**.

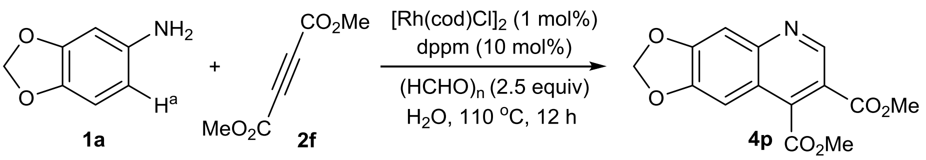

H^a	Yield of **4p**
H	19
D (77%)	6

Procedure: To a 5 mL clean, oven-dried screw cap reaction tube was added $[Rh(cod)Cl]_2$ (1 mol%), dppm (10 mol%), **1a** or [**D]1a** (0.05 mmol), paraformaldehyde (2.5 equiv), dimethyl acetylenedicarboxylate **2c** (1.1 equiv) and water (150 µL) under argon atm. The reaction mixture was kept for heating at 100 ^oC for 8 h. After cooling to room temperature reaction mixture was diluted with DCM. Then transfer to a 10 mL round bottle flask and concentrated through rotary evaporator and isolated through column chromatography. The isolated yields of **4p** were 19% (in case **1a**) and 6% (in case of [**D]1a**) respectively. This result implies that the breaking of *ortho*C-H bond (electrophilic metalation step) may be the rate limiting step.

Disregard of other possible mechanisms

N-formylated 3,4-(methylenedioxy)aniline was prepared by using known literature procedure.[6]

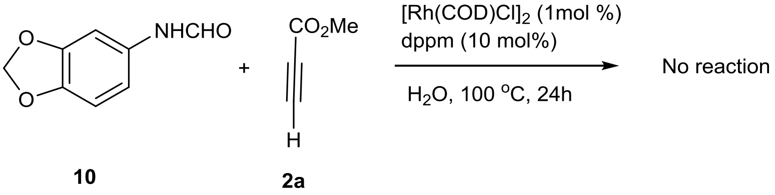

Procedure: To a 10 mL clean, oven-dried screw cap reaction tube was added $[Rh(cod)Cl]_2$ (1 mol%), dppm (10mol%), *N*-(benzo[d][1,3]dioxol-5-yl)formamide **10**

(0.25 mmol) and methyl propiolate **2a** (0.275 mmol) and water (250 µL) under argon atm. The reaction mixture was heated at 100 °C for 24 h. After cooling at room temperature reaction mixture was diluted with 8 mL water and reaction mixture was extracted with ethyl acetate (3 x 5 mL). The combined organic layer was dried over anhydrous Na_2SO_4 and the solvent was evaporated in vacuum. ^1H NMR clearly reveled there is no formation of desired quinoline (**4a**) and the staring material **9** was completely (95%) recovered.

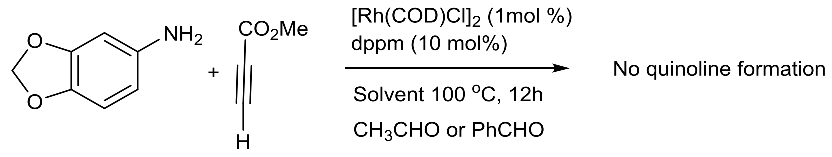

Procedure: To an oven dried 10 mL screw cap reaction tube was added $[Rh(cod)Cl]_2$ (1 mol%), dppm (10mol%), aldehyde (0.25 mmol) and methyl propiolate (0.275 mmol) and solvent (DMF or H_2O, 250 µL) under argon atm. The reaction mixture was heated at 100 °C for 12h. After cooling at room temperature reaction mixture was diluted with 10 mL of ethyl acetate and passed through celite pad. The organic layer was concentrated under reduced vacuum. ^1H NMR reveled there are no formation of desired quinoline derivatives and the staring material **1a** was (reaction in water: 93% in case of acetaldehyde and 89% in case of benzaldehyde; reaction in DMF: 90% in case of acetaldehyde and 81% in case of benzaldehyde) recovered.

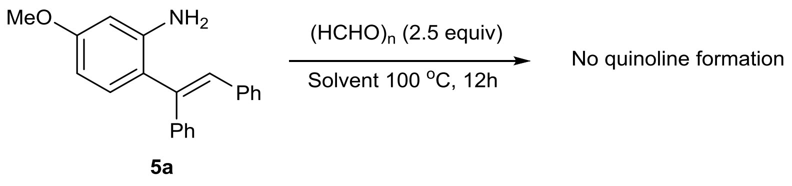

Procedure: To an oven dried 10 mL screw cap reaction tube was added **5a**, paraformaldehyde (2.5 equiv.) and solvent (THF or H_2O, 250 μL) under argon atm. The reaction mixture was heated at 100 ^{o}C for 24 h. After cooling at room temperature reaction mixture was diluted with 10 mL of ethyl acetate and passed through celite pad. The organic layer was concentrated under reduced vacuum. ^{1}H NMR reveled there is no formation of desired quinoline derivative and the staring material **5a** was recovered (90% in water and 95% in THF).

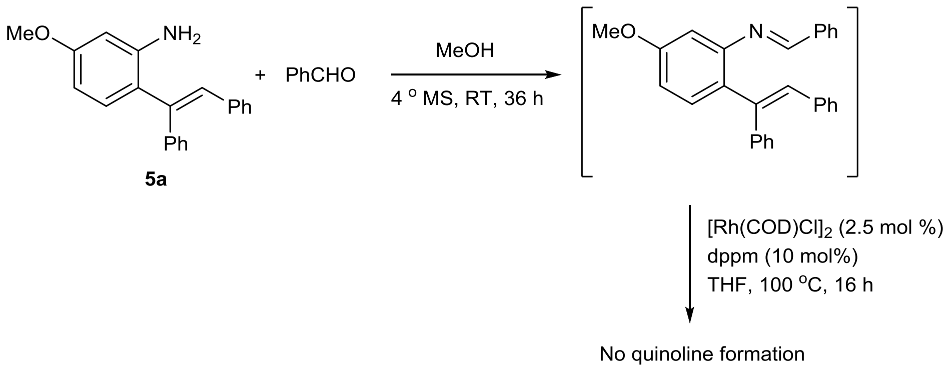

Procedure: To an oven dried 10 mL screw cap reaction tube was added **5a**, PhCHO (0.11 mmol), MeOH (5 mL) and 4° MS under argon atm. The reaction mixture was stirred at room temperature. After 36 h, the solvent was removed under high vacuum and was added $[Rh(cod)Cl]_2$ (2.5 mol%), dppm (10 mol%), and THF (250 μL) under argon atm. The reaction mixture was heated at 100 ^{o}C for 16 h. After cooling at room temperature reaction mixture was concentrated under reduced vacuum. ^{1}H NMR of the reaction mixture reveled there is no formation of desired quinoline derivative (**6a**) and the staring material **5a** was observed (probably due to hydrolysis).

These experiment results implied that auto-tandem approach should proceed through carbonylation path and not *via* either *N*-formylation, imine intermediate, and [3,3] rearrangement.

Characterization of structurally new compounds

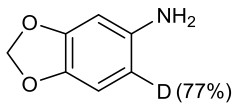

D[1a]

benzo[d][1,3]dioxol-5-amine (D[1a])

Light brown liquid. ^{1}**H NMR** (400 MHz, Chloroform-d) δ 6.60 (s, 1H), 6.27 (s, 1H), 6.11 (dt, *J* = 8.0Hz and 4.0Hz, 0.23H), 5.84 (s, 2H), 3.44 (s, 2H). ^{13}**C NMR** (125.8 MHz) δ 148.14, 141.34, 141.27, 140.30, 108.52, 108.43, 106.82, 100.60, 98.02. **HRMS (ESI)** calcd. for $C_7H_6DNO_2$ $[M]^+$: 138.0539; found: 138.0540.

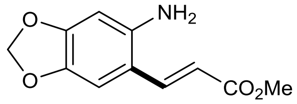

3a

(*E*)-methyl 3-(6-aminobenzo[d][1,3]dioxol-5-yl)acrylate (3a)

Yellow solid. ^{1}**H NMR** (200 MHz, Chloroform-d) δ 7.77 (d, *J* = 14.1 Hz, 1H), 6.87 (s, 1H), 6.26 (s, 1H), 6.17 (d, *J* = 16.0 Hz, 1H), 5.91 (s, 2H), 3.87 (s, 2H), 3.79 (s, 3H). ^{13}**C NMR** (125.8 MHz) δ 168.04, 150.77, 142.33, 141.38, 139.47, 114.48, 112.15, 105.80, 101.19, 98.14, 51.54. **HRMS (ESI)** calcd. for $C_{11}H_{12}NO_4$ $[M+H]^+$: 222.0688; found: 222.0692.

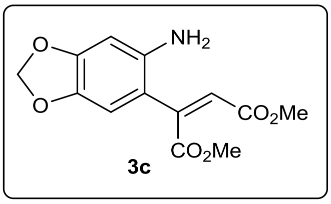

Dimethyl 2-(6-aminobenzo[d][1,3]dioxol-5-yl)maleate (3c)

Compound **3c** was isolated by silica gel column chromatography using petroleum ether/ethyl acetate (v/v 80:20) as eluent (both **4p** and **3c** are having same R_f value). Thus we obtained as an inseparable mixture (**3c** + **4p;** 1:1.16 by ^{1}H NMR).Combined yield: 28%.**^{1}H NMR** (500 MHz, Chloroform-d) δ 8.55 (s, 1H), 7.47 (s, 1H), 7.16 (s, 1H), 6.21 (s, 2H), 4.1 (s, 3H), 4.0 (s, 3H).**^{13}C NMR** (125.8 MHz, Chloroform-d) δ 167.36, 165.87, 153.22, 149.69, 148.60, 147.1, 124.72, 120.87, 120.52, 106.2, 103.2, 102.49, 53.18, 52.79. Due to mixture of **3c** and **4p** HRMS (ESI) was not recorded.

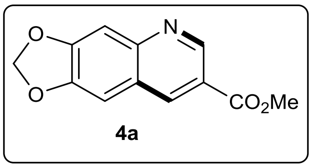

Methyl [1,3]dioxolo[4,5-g]quinoline-7-carboxylate (4a)[7]

Light yellow solid. Yield: 95% (54.8 mg) by procedure (a) and 61% (35.2 mg) by procedure (b). **^{1}H NMR** (400 MHz, Chloroform-d) δ 9.22 (s, 1H), 8.61 (s, 1H), 7.41 (s, 1H), 7.12 (s, 1H), 6.14 (s, 2H), 3.98 (s, 3H). **^{13}C NMR** (100.6 MHz, Chloroform-d) δ 166.10, 152.70, 148.76, 148.56, 148.10, 137.01, 124.02, 121.47, 106.0, 103.59, 102.18, 52.31. **HRMS (ESI)** calcd. for $C_{12}H_{10}NO_4$ $[M+H]^+$: 232.0632; found: 232.0634.

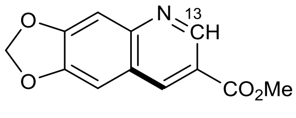
([13C]4a)

Methyl [1,3]dioxolo[4,5-g]quinoline-7-carboxylate ([^{13}C]4a)

Eluent: petroleum ether/ethyl acetate (v/v 85:15). Colourless solid. Yield: 87% (50.4 mg). **^{1}H NMR** (400 MHz, Chloroform-d) δ 9.25 (d, *J* = 200.0 Hz, 1H), 8.64 (s, 1H), 7.43 (s, 1H), 7.13 (s, 1H), 6.17 (s, 2H), 4.01 (s, 3H). **^{13}C NMR** (100.6 MHz, Chloroform-d) δ 166.17, 160.01, 152.72, 148.42, 148.15 (major), 147.86, 137.03, 105.96, 103.36, 102.22, 52.36. **HRMS (ESI)** calcd. for $C_{11}{}^{13}CH_{10}NO_4$ $[M+H]^+$: 233.0523, found: 233.0522.(The ratio of **4a : [^{13}C]4a** = 7 : 93 based on ^{1}H NMR analysis).

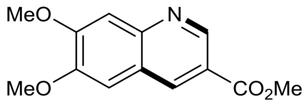
4b

Methyl 6,7-dimethoxyquinoline-3-carboxylate (4b)[7]

Pale yellow solid. Yield: 87% (53.7 mg). **^{1}H NMR** (500 MHz, Chloroform-d) δ 9.29 (s, 1H), 8.71 (s, 1H), 7.50 (s, 1H), 7.16 (s, 1H), 4.09 (s, 3H), 4.06 (s, 3H), 4.02 (s, 3H).**^{13}C NMR** (125.8 MHz, Chloroform-d) δ 166.22, 154.43, 150.42, 148.03, 147.36, 136.58, 122.54, 121.36, 107.88, 105.91, 56.32, 56.13, 52.29. **HRMS (ESI)** calcd. for $C_{13}H_{13}NO_4$ $[M]^+$: 247.0845, found: 247.0849.

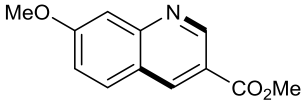
4c

Methyl 7-methoxyquinoline-3-carboxylate (4c)[8]

Yield: 84% (21.8 mg, 0.12 mmol scale). **^{1}H NMR** (200 MHz, Chloroform-d) δ 9.38 (s, 1H), 8.76 (s, 1H), 7.82 (d, *J* = 10.0 Hz,1H), 7.48 (s, 1H), 7.27 (dd, *J* = 10.4 Hz and 4.2 Hz,1H), 4.01 (s, 3H), 3.99 (s, 3H). **^{13}C NMR** (100.6 MHz, Chloroform-d) δ 166.12, 162.73, 151.93, 150.51, 138.24, 130.17, 129.97, 122.05, 120.98, 120.86, 110.41, 107.45, 55.72, 52.33. **HRMS (ESI)** calcd. for $C_{12}H_{11}NO_3$ $[M+H]^+$: 218.0817, found: 218.0823.

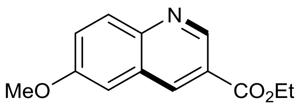

4d

Ethyl 6-methoxyquinoline-3-carboxylate (4d)[9]

Greenish yellow liquid.Yield: 32% (19 mg). **^{1}H NMR** (500 MHz, Chloroform-d) δ 9.33 (s, 1H), 8.77 (s, 1H), 8.08 (d, *J* = 10.1 Hz, 1H), 7.50 (dd, *J* = 10.3 Hz and 3.2 Hz, 1H), 7.20 (s, 1H), 4.50 (q, *J* = 7.2 Hz, 2H), 3.98 (s, 3H), 1.49 (t, *J* = 7.1 Hz, 3H). **^{13}C NMR** (125.8 MHz, Chloroform-d) δ 165.60, 158.32, 147.70, 146.10, 137.26, 130.83, 128.58, 124.72, 123.55, 106.02, 61.46, 55.66, 14.36. **HRMS (ESI)** calcd. for $C_{13}H_{13}NO_3$ $[M]^+$: 231.0895, found: 231.0890.

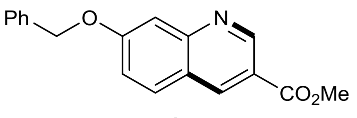

4e

Methyl 7-(benzyloxy)quinoline-3-carboxylate(4e)

Compound **4e** was prepared according to the general procedure and was purified by silica gel column chromatography using petroleum ether/ethyl acetate (v/v 90:10) as an eluent. Yellow solid. Yield: 85% (62.5 mg). **^{1}H NMR** (400 MHz, Chloroform-d) δ

9.40 (s, 1H), 8.78 (s, 1H), 7.85 (d, *J* = 8.0 Hz, 1H), 7.58 (s, 1H), 7.52 (d, *J* = 8.2 Hz, 2H), 7.44 (m, 2H), 7.38 (t, *J* = 8.0 Hz, 2H), 5.27 (s, 2H), 4.03 (s, 3H). **^{13}C NMR** (100.6 MHz, Chloroform-d) δ 166.09, 161.78, 151.83, 150.51, 138.25, 135.96, 130.27, 128.74, 127.73, 122.17, 121.16, 121.09, 108.62, 70.43, 52.34. **HRMS (ESI)** calcd. for $C_{18}H_{16}NO_3$ $[M+H]^+$: 294.1120, found: 294.1125.

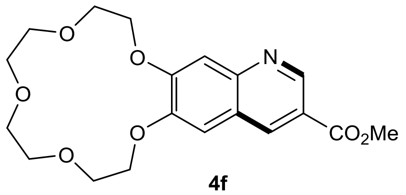

4f

Methyl 2,3,5,6,8,9,11,12-octahydro-[1,4,7,10,13]pentaoxacyclopentadeca[2,3-g] quinoline-17-carboxylate (4f)

Compound **4f** was prepared according to the general procedure and was purified by silica gel column chromatography using petroleum ether/ethyl acetate (v/v 20:80) as an eluent. Gray solid. Yield: 82% (77.4 mg). **^{1}H NMR** (400 MHz, DMSO-d_6) δ 8.16 (s, 1H), 7.12 (s, 1H), 6.80 (s, 1H), 6.63 (s, 1H), 3.33 (s, 2H), 3.27 (s, 2H), 2.97 (s, 2H), 2.91 (s, 4H), 2.71 (s, 8H). **^{13}C NMR** (100.6 MHz, DMSO-d_6) δ 165.66, 153.65, 149.39, 147.12, 146.97, 136.24, 132.01, 130.54, 128.89, 122.29, 120.68, 108.24, 107.79, 70.31, 69.17, 68.15, 52.32. **HRMS (ESI)** calcd. for $C_{19}H_{24}NO_7$ $[M+H]^+$: 378.1538, found: 378.1547.

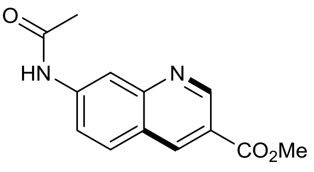

4g

Methyl 7-acetamidoquinoline-3-carboxylate (4g)

Compound **4g** was prepared according to the general procedure and was purified by silica gel column chromatography using petroleum ether/ethyl acetate (v/v 50:50) as an eluent. Pale yellow solid. Yield: 79% (48.2 mg). **^{1}H NMR** (500 MHz, $CDCl_3$/DMSO-d_6 (1:1)) δ 9.48 (s, 1H), 8.39 (s, 1H), 7.92 (s, 1H), 7.62 (s, 1H), 7.10 (d, J = 10.0 Hz, 1H), 6.95 (d, J = 10.0 Hz, 1H), 3.09 (s, 3H), 1.30 (s, 3H). **^{13}C NMR** (125.8 MHz, $CDCl_3$/DMSO-d_6) δ 167.23, 163.47, 148.57, 147.89, 140.60, 135.89, 127.76, 120.84, 119.27, 119.10, 113.76, 50.20, 22.36. **HRMS (ESI)** calcd. for $C_{13}H_{13}N_2O_3$ $[M+H]^+$: 245.0916, found: 245.0921.

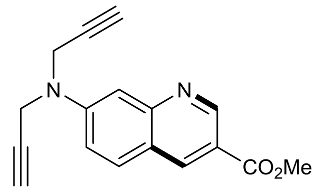

4h

Methyl 7-(diprop-2-ynylamino)quinoline-3-carboxylate (4h)

Compound **4h** was prepared according to the general procedure and was purified by silica gel column chromatography using petroleum ether/ethyl acetate (v/v 50:50) as aneluent. Gummy solid. Yield: 80% (55.7 mg). **^{1}H NMR** (500 MHz, $CDCl_3$) δ 9.35 (s, 1H), 8.71 (s, 1H), 7.87 (d, J = 10.1 Hz, 1H), 7.51 (s, 1H), 7.38 (dd, J = 10.0 and 5.2 Hz, 1H), 4.33 (d, J = 2.0 Hz, 4H), 4.01 (s, 3H), 2.33 (t, J = 2.0 Hz, 2H). **^{13}C NMR** (125.8 MHz, $CDCl_3$) δ 166.24, 151.38, 150.73, 150.21, 138.20, 130.24, 120.57, 120.13, 118.01, 109.78, 78.33, 73.22, 52.25, 40.30. **HRMS (ESI)** calcd. for $C_{17}H_{15}N_2O_2$ $[M+H]^+$: 279.1124, found: 279.1128.

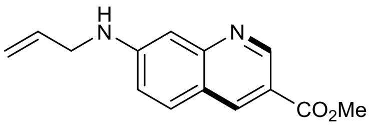

4i

Methyl 7-(allylamino)quinoline-3-carboxylate (4i)

Compound **4i** was prepared according to the general procedure and was purified by silica gel column chromatography using petroleum ether/ethyl acetate (v/v 85:15) as aneluent. Viscous liquid. Yield: 73% (44.2 mg). **^{1}H NMR** (200 MHz, Chloroform-d) δ 9.26 (s, 1H), 8.61 (s, 1H), 7.67 (d, *J* = 8.0 Hz, 1H), 7.08 (s, 1H), 6.97 (d, *J* = 8.2 Hz, 1H), 5.95 (m, 1H), 5.35 (d, *J* = 18.1 Hz, 1H), 5.24 (d, *J* = 8.0 Hz, 1H), 3.97 (s, 3H), 3.75 (m, 3H). **^{13}C NMR** (100.6 MHz, Chloroform-d) δ 166.38, 152.12, 150.97, 150.54, 150.32, 138.17, 133.84, 130.11, 120.24, 119.14, 117.21, 104.93, 52.09, 45.95. **HRMS (ESI)** calcd. for $C_{14}H_{15}N_2O_2$ $[M+H]^+$: 243.1123, found: 243.1128.

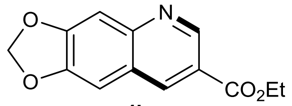

4k

Ethyl [1,3]dioxolo[4,5-g]quinoline-7-carboxylate (4k)

Compound **4k** was prepared according to the general procedure and was purified by silica gel column chromatography using petroleum ether/ethyl acetate (v/v 85:15) as aneluent. White solid. Yield: 90% (55.2 mg). **^{1}H NMR** (400 MHz, Chloroform-d) δ 9.27 (s, 1H), 8.64 (s, 1H), 7.45 (s, 1H), 7.17 (s, 1H), 6.18 (s, 2H), 4.48 (q, *J* = 7.4 Hz, 2H), 1.47 (t, *J* = 8.0 Hz, 3H). **^{13}C NMR** (125.8 MHz, Chloroform-d) δ 165.68, 152.65, 148.79, 148.55, 148.55, 148.23, 124.06, 121.83, 105.96, 103.63, 102.19, 61.33, 14.36. **HRMS (ESI)** calcd. for $C_{13}H_{12}NO_4$ $[M+H]^+$: 246.0763, found: 246.0761.

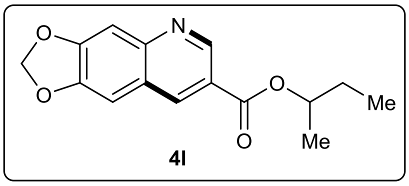

***sec*-butyl [1,3]dioxolo[4,5-g]quinoline-7-carboxylate (4l)**

Compound **4l** was prepared according to the general procedure and was purified by silica gel column chromatography using petroleum ether/ethyl acetate (v/v 85:15) as aneluent. Brown coloured solid. Yield: 85% (58.1 mg). **^{1}H NMR** (200 MHz, Chloroform-d) δ 9.25 (s, 1H), 8.60 (s, 1H), 7.43 (s, 1H), 7.15 (s, 1H), 6.16 (s, 2H), 5.17 (q, *J* = 6.2 Hz, 1H), 1.82-1.67 (m, 2H), 1.38 (d, *J* = 6.0 Hz, 3H), 1.0 (t, *J* = 6.0 Hz, 3H). **^{13}C NMR** (125.8 MHz, Chloroform-d) δ 165.55, 152.83, 148.95, 148.75, 148.49, 137.10, 124.31, 122.45, 106.18, 103.85, 102.40, 73.65, 29.19, 19.84, 10.00. **HRMS (ESI)** calcd. for $C_{15}H_{15}NO_4$ $[M+H]^+$: 273.2839, found: 273.2843.

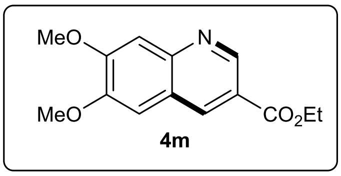

Ethyl 6,7-dimethoxyquinoline-3-carboxylate (4m)[4]

Colourless liquid. Yield: 81% (53 mg). **^{1}H NMR** (200 MHz, Chloroform-d) δ 9.27 (s, 1H), 8.86 (s, 1H), 7.47 (s, 1H), 7.13 (s, 1H), 4.46 (q, *J* = 8.0 Hz, 2H), 4.07 (s, 3H), 4.03 (s, 3H), 1.46 (t, *J* = 8.2 Hz, 3H). **^{13}C NMR** (100.6 MHz, Chloroform-d) δ 165.65, 154.21, 150.25, 148.04, 147.26, 136.32, 122.40, 121.55, 107.82, 105.82, 61.14, 56.20, 56.04, 14.27. **HRMS (ESI)** calcd. for $C_{14}H_{15}NO_4$ $[M]^+$: 261.1001, found: 261.0994.

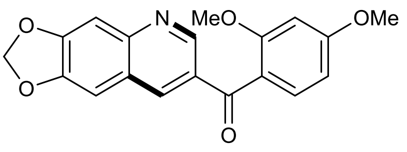

4n

[1,3]dioxolo[4,5-g]quinolin-7-yl(2,4-dimethoxyphenyl)methanone (4n)

Compound **4n** was prepared according to the general procedure (24 h) and was purified by silica gel column chromatography using petroleum ether/ethyl acetate (v/v 85:15) as eluent. Colourless liquid. Yield: 72% (60.7 mg). ^{1}**H NMR** (500 MHz, Chloroform-d) δ 8.98 (s, 1H), 8.46 (s, 1H), 7.57 (d, *J* = 10.0 Hz, 1H), 7.17 (s, 1H), 8.64 (dd, *J* = 10.0 and 5.2 Hz, 1H), 6.55 (s, 1H), 6.19 (s, 2H), 5.88 (d, *J* = 10.0 Hz, 1H), 3.93 (s, 3H), 3.71 (s, 3H). ^{13}**C NMR** (125.8 MHz, Chloroform-d) δ 164.17, 159.70, 137.03, 132.71, 130.27, 105.83, 105.45, 105.28, 103.91, 102.27, 100.94, 98.73, 55.64, 55.57. **HRMS (ESI)** calcd. for $C_{19}H_{16}NO_5$ $[M+H]^+$: 338.1019, found: 338.1023.

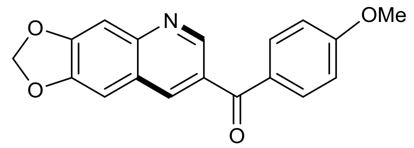

4o

[1,3]dioxolo[4,5-g]quinolin-7-yl(4-methoxyphenyl)methanone (4o)

Compound **4o** was prepared according to the general procedure (20 h) and was purified by silica gel column chromatography using petroleum ether/ethyl acetate (v/v 85:15) as eluent. White solid. Yield: 76% (58.4 mg). ^{1}**H NMR** (200 MHz, Chloroform-d) δ 9.06 (s, 1H), 8.37 (s, 1H), 7.87 (d, *J* = 8.0 Hz, 2H), 7.46 (s, 1H), 7.16 (s, 1H), 7.01 (d, *J* = 8.0 Hz, 2H), 6.17 (s, 2H), 3.92 (s, 3H). ^{13}**C NMR** (125.8 MHz, Chloroform-d) δ 193.57, 163.58, 152.59, 148.68, 148.40, 148.14, 136.94, 132.52,

129.92, 129.40, 124.04, 113.87, 105.84, 105.47, 103.64, 102.22, 100.88, 55.58. **HRMS (ESI)** calcd. for $C_{18}H_{14}NO_4$ $[M+H]^+$: 308.0916, found: 308.0891.

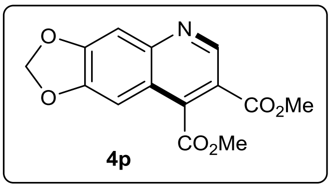

Dimethyl [1,3]dioxolo[4,5-g]quinoline-7,8-dicarboxylate (4p)

Compound **4p** was prepared according to the general procedure (24 h) and was purified by silica gel column chromatography using petroleum ether/ethyl acetate (v/v 80:20) as an eluent. Pale yellow solid. Yield: 69% (50 mg). **^{1}H NMR** (200 MHz, Chloroform-d) δ 9.25 (s, 1H), 7.46 (s, 1H), 7.07 (s, 1H), 6.20 (s, 2H), 4.10 (s, 3H), 4.0 (s, 3H). **^{13}C NMR** (125.8 MHz, Chloroform-d) δ 167.75, 165.13, 152.94, 149.53, 149.11, 147.89, 140.76, 137.52, 117.67, 106.10, 102.57, 100.82, 53.19, 52.78. **HRMS (ESI)** calcd. for $C_{14}H_{12}NO_6$ $[M+H]^+$: 290.0654, found: 290.0659.

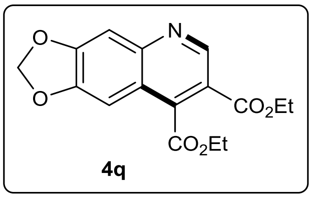

Diethyl [1,3]dioxolo[4,5-g]quinoline-7,8-dicarboxylate (4q)

Compound **4q** was prepared according to the general procedure (24 h) and was purified by silica gel column chromatography using petroleum ether/ethyl acetate (v/v 80:20) as an eluent. Yellow solid. Yield: 72% (57 mg). **^{1}H NMR** (400 MHz, Chloroform-d) δ 9.21 (s, 1H), 7.41 (s, 1H), 7.05 (s, 1H), 4.54 (q, J = 8.0 Hz, 2H), 4.41 (q, J = 8.0 Hz, 2H), 4.13 (t, J = 8.2 Hz, 3H), 4.1 (t, J = 8.0 Hz, 3H). **^{13}C NMR** (125.8 MHz, Chloroform-d) δ 166.98, 164.37, 152.50, 149.14, 148.73, 147.74, 140.53,

120.24, 117.65, 105.89, 102.22, 100.48, 62.01, 61.54, 13.93, 13.81. **HRMS (ESI)** calcd. for $C_{14}H_{12}NO_6$ $[M+H]^+$: 318.0970, found: 318.0972.

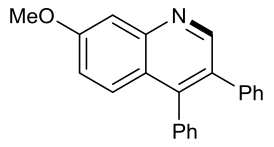

6a

7-methoxy-3,4-diphenylquinoline (6a)

Eluent: petroleum ether/ethyl acetate (v/v 90:10). Colourless solid. Yield: 82% (63.8 mg). **^{1}H NMR** (500 MHz, Chloroform-d) δ 8.94 (s, 1H), 7.60 (d, *J* = 8.0 Hz, 1H), 7.54 (s, 1H), 7.37-7.36 (m, 3H), 7.26-7.24 (m, 3H), 7.23-7.21 (m, 2H), 7.18 (d, *J* = 10.0 Hz, 2H), 7.15-7.17 (m, 1H), 4.02 (s, 3H). **^{13}C NMR** (125.8 MHz, Chloroform-d) δ 160.42, 151.95.149.32, 145.55, 138.26, 136.45, 131.37, 130.48, 130.17, 128.12, 128.01, 127.76, 127.69, 126.84, 122.28, 119.85, 107.36, 55.59. **HRMS (ESI)** calcd. for $C_{22}H_{18}NO$ $[M+H]^+$: 312.1377, found: 312.1383.

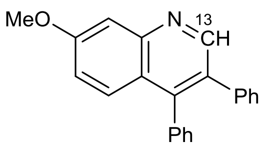

([13C]6a)

7-methoxy-3,4-diphenylquinoline ([^{13}C]6a)

Eluent: petroleum ether/ethyl acetate (v/v 90:10). Colourless solid. Yield: 71% (55 mg). **^{1}H NMR** (400 MHz, Chloroform-d) δ 8.94 (d, *J* = 180.0 Hz, 1H), 7.6 (d, *J* = 8.0 Hz, 1H), 7.54 (s, 1H), 7.37-7.35 (m, 3H), 7.24 (t, *J* = 8.0 Hz, 3H), 7.21 (dd, *J* = 8.0 Hz and 4.0 Hz, 2H), 7.17 (dd, *J* = 10.0 Hz and 4.2 Hz, 3H), 4.01 (s, 3H). **^{13}C NMR** (100.6 MHz, Chloroform-d) δ 162.98, 160.43, 152.91 (major), 150.72, 149.25,

145.58, 138.24, 136.44, 131.6, 131.07, 130.47, 130.17, 128.13, 128.01, 27.77, 27.69, 126.85, 119.86, 107.35, 107.27, 55.60. **HRMS (ESI)** calcd. for $C_{21}{}^{13}CH_{18}NO$ $[M+H]^+$: 313.1416, found: 313.1416. (The ratio of the **6a:[^{13}C]6a** = 6 : 94, based on ^{1}H NMR analysis).

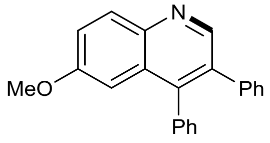

6b

6-methoxy-3,4-diphenylquinoline (6b)

Eluent: petroleum ether/ethyl acetate (v/v 95:5). Colourless solid. Yield: 74% (57.6 mg). **^{1}H NMR** (500 MHz, Chloroform-d) δ 8.88 (s, 1H), 8.12 (d, *J* = 10.1 Hz, 1H), 7.41 (dd, *J* = 10.0 and 5.2 Hz, 1H), 7.38-7.35 (m, 3H), 7.26-7.22 (m, 5H), 7.19 (dd, *J* = 10.0 Hz and 5.2 Hz, 2H), 6.97 (s, 1H), 3.76 (s, 3H). **^{13}C NMR** (125.8 MHz, Chloroform-d) δ 158.06, 149.42, 144.25, 143.71, 138.33, 136.56, 133.43, 130.94, 130.4, 130.13, 128.25, 128, 127.69, 126.98, 121.43, 104.58, 55.38. **HRMS (ESI)** calcd. for $C_{22}H_{18}NO$ $[M+H]^+$: 312.1377, found: 312.1383.

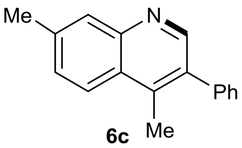

6c

4,7-dimethyl-3-phenylquinoline (6c)

Eluent: petroleum ether/ethyl acetate (v/v 97:3). Yellow liquid. Yield: 72% (42 mg). **^{1}H NMR** (400 MHz, Chloroform-d) δ 8.76 (s, 1H), 7.99 (d, *J* = 8.0 Hz, 1H), 7.92 (s, 1H), 7.52 (d, *J* = 8.2 Hz, 2H), 7.49-7.27 (m, 4H), 2.63 (s, 3H), 2.60 (s, 3H). **^{13}C NMR** (100.6 MHz, Chloroform-d) δ 151.49, 147.26, 140.37, 139.08, 138.80, 133.76,

129.98, 129.00, 128.94, 128.42, 127.45, 125.97, 123.93, 21.64, 15.58. **HRMS (ESI)** calcd. for $C_{17}H_{16}N$ $[M+H]^+$: 234.1274, found: 234.1277.

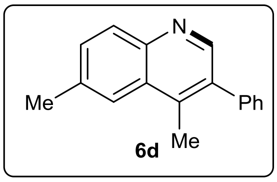

6d

4,6-dimethyl-3-phenylquinoline (6d)

Eluent: petroleum ether/ethyl acetate (v/v 97:3). Yellow liquid. Yield: 71% (41.4 mg). ^{1}**H NMR** (500 MHz, Chloroform-d) δ 8.76 (s, 1H), 8.06 (s, 1H), 7.88 (s, 1H), 7.60 (s, 1H), 7.52 (d, J = 8.3 Hz, 2H), 7.47-7.43 (m, 3H). ^{13}**C NMR** (125.8 MHz, Chloroform-d) δ 150.67, 145.56, 139.84, 138.85, 136.58, 134.46, 131.07, 129.97, 129.71, 128.91, 128.43, 127.92, 127.50, 123.23, 22.02, 15.63. **HRMS (ESI)** calcd. for $C_{17}H_{15}N$ $[M]^+$: 233.1204, found: 233.1204.

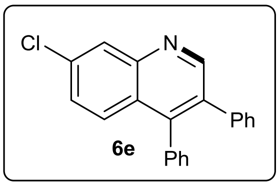

6e

7-chloro-3,4-diphenylquinoline (6e)

Eluent: petroleum ether/ethyl acetate (v/v 97:3). White solid. Yield: 80% (63.2 mg). ^{1}**H NMR** (500 MHz, Chloroform-d) δ 9.02 (s, 1H), 8.21 (s, 1H), 7.67 (s, 1H), 7.46 (s, 1H), 7.44-7.36 (m, 3H), 7.29-7.25 (m, 3H), 7.21-7.17 (m, 4H). ^{13}**C NMR** (125.8 MHz, Chloroform-d) δ 152.87, 147.97, 145.52, 137.69, 135.79, 135.03, 130.39, 130.06, 128.39, 128.29, 128.13, 128.04, 127.96, 127.80, 125.74. **HRMS (ESI)** calcd. for $C_{21}H_{14}ClN$ $[M]^+$: 315.0818, found: 315.0815.

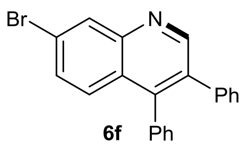

6f

7-bromo-3,4-diphenylquinoline (6f)

Eluent: petroleum ether/ethyl acetate (v/v 97:3). White solid. Yield: 79% (71 mg). **^{1}H NMR** (500 MHz, Chloroform-d) δ 9.01 (s, 1H), 8.39 (s, 1H), 7.58 (s, 2H), 7.38 (d, *J* = 10.1 Hz, 3H), 7.29-7.26 (m, 3H), 7.20-7.17 (m, 4H). **^{13}C NMR** (125.8 MHz, Chloroform-d) δ 152.32, 148.20, 145.62, 137.70, 135.74, 131.73, 130.42, 130.34, 130.06, 128.30, 128.15, 128.11, 127.99, 127.26, 126.03, 123.32. **HRMS (ESI)** calcd. for $C_{21}H_{14}BrN$ $[M]^{+}$: 359.0311, found: 359.031.

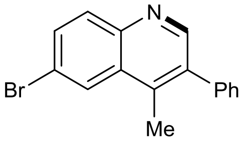

6g

6-bromo-4-methyl-3-phenylquinoline (6g)

Eluent: petroleum ether/ethyl acetate (v/v 95:5). Yield: 58% (from crude mixture by ^{1}H NMR). **^{1}H NMR** (500 MHz, Chloroform-d) δ 8.84 (s, 1H), 8.31 (s, 1H), 8.16 (d, *J* = 10.0 Hz, 1H), 7.87 (d, *J* = 10.0 Hz, 1H), 7.55 (t, *J* = 5.4 Hz, 2H), 7.49 (t, *J* = 10.2 Hz, 1H), 7.42-7.38 (m, 2H), 2.67 (s, 3H). Due to inseparable mixture of (**5g** and product **6g**) **^{13}C NMR** was not recorded. **HRMS (ESI)** calcd. for $C_{16}H_{13}NBr$ $[M+H]^{+}$: 298.0221, found: 298.0226.

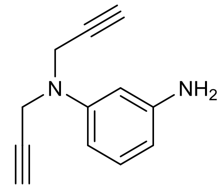

1h

***N,N*'-di(prop-2-ynyl)benzene-1,3-diamine (1h)**

Compound **1h** was prepared according to the general procedure as described above (method A and method B) using proporgyl bromide as an alkylating agent and was purified by silica gel column chromatography using petroleum ether/ethyl acetate (v/v 90:10) as an eluent.

Brown liquid. Yield: 48%. **^{1}H NMR** (400 MHz, Chloroform-d) δ 7.09 (t, *J* = 8.0 Hz, 1H), 6.42 (dd, *J* = 8.4 Hz and 5.0 Hz, 1H), 6.31 (s, 1H), 6.26 (dd, *J* = 8.4 Hz and 5.1 Hz, 1H), 4.11 (s, 4H), 3.67 (s, 2H, NH_2), 2.28 (s, 2H). **^{13}C NMR** (100.6 MHz, Chloroform-d) δ 149.02, 147.39, 130.01, 107.18, 106.41, 102.58, 79.46, 72.59, 40.36. **HRMS (ESI)** calcd. for $C_{12}H_{13}N_2$ $[M+H]^+$: 185.1070; found: 185.1073.

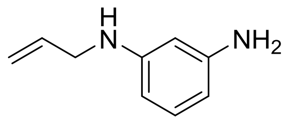

1i

***N*-allylbenzene-1,3-diamine (1i)**

Compound**1i** was prepared according to the general procedure (method A and B) as described above using allyl bromide as an alkylating agent and was purified by silica gel column chromatography using petroleum ether/ethyl acetate (v/v 90:10) as an eluent.

Brown liquid. Yield: 37%. **^{1}H NMR** (500 MHz, Chloroform-d) δ 7.01 (t, *J* = 10.0 Hz, 1H), 6.13 (d, *J* = 10.1 Hz, 2H), 6.01-5.99 (m, 2H), 5.33 (d, *J* = 19.2 Hz, 1H), 5.21 (d, *J* = 10 Hz, 1H), 3.78 (t, *J* = 5,2 Hz, 2H), 3.58 (s, 2H) . **^{13}C NMR** (125.8 MHz,

Chloroform-d) δ 149.15, 147.39, 135.54, 129.88, 115.90, 104.92, 103.99, 99.59, 46.39. **HRMS (ESI)** calcd. for $C_9H_{13}N_2$ $[M+H]^+$: 149.1073; found: 149.1073.

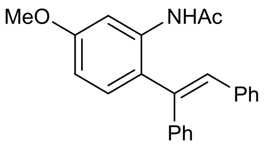

5a'

(*E*)-*N*-(2-(1,2-diphenylvinyl)-5-methoxyphenyl)acetamide (5a')

Compound **5a'** was prepared from 3-methoxy acetanilide and diphenylacetylene by Method C. Light brown solid. **^{1}H NMR** (200 MHz, Chloroform-d) δ 7.99 (s, 1H), 7.36-7.30 (m, 6H), 7.19-7.14 (m, 5H), 7.05 (d, *J* = 10 Hz, 2H), 6.68 (d, *J* = 10.2 Hz, 1H), 3.85 (s, 3H), 1.74 (s, 3H). **^{13}C NMR** (50.3 MHz, Chloroform-d) δ 168.25, 159.97, 142.26, 137.48, 136.58, 136.45, 131.57, 130.27, 128.88, 128.73, 128.54, 128.20, 127.92, 127.02, 121.43, 11.05, 106.2, 55.41, 24.57. **HRMS (ESI)** calcd. for $C_{23}H_{21}NO_2Na$ $[M+Na]^+$: 366.1461; found: 366.1465.

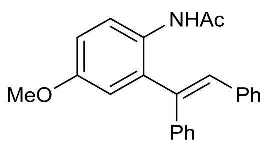

5b'

(*E*)-*N*-(2-(1,2-diphenylvinyl)-4-methoxyphenyl)acetamide (5b')

Compound **5b'**was prepared from 4-methoxy acetanilide and diphenylacetylene by Method C. White solid. **^{1}H NMR** (200 MHz, Chloroform-d) δ 7.77 (d, *J* = 8.0 Hz, 1H), 7.28-7.25 (m, 5H), 7.22-7.16 (m, 5H), 6.96 (s, 1H), 6.89 (dd, *J* = 8.4Hz, 1H), 6.78 (s, 1H), 6.69 (s, 1H), 3.82 (s, 3H), 1.69 (s, 3H). **^{13}C NMR** (100.6 MHz) δ 167.96, 156.59, 139.79, 139.17, 137.06, 136.60, 132.02, 129.56, 129.51, 129.31, 128.42,

128.18, 128.06, 127.38, 125.01, 116.31, 113.58, 55.55, 23.79. **HRMS (ESI)** calcd. for $C_{23}H_{21}NO_2Na$ $[M+Na]^+$: 366.1454; found: 366.1465.

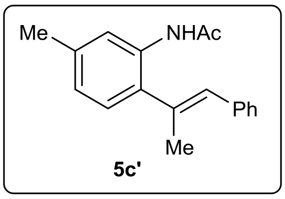

(*E*)-*N*-(5-methyl-2-(1-phenylprop-1-en-2-yl)phenyl)acetamide (5c')

Compound **5c'** was prepared from 3-methyl acetanilide and 1-phenyl-1-propyne by Method C. White solid. ^{1}**H NMR** (200 MHz, Chloroform-d) δ 8.07 (s, 1H), 7.45-7.39 (m, 5H), 7.32 (t, *J* = 8.4 Hz, 1H), 7.14 (d, *J* = 8.4 Hz, 1H), 6.97 (d, *J* = 8.0 Hz, 1H), 6.52 (s, 1H), 2.40 (s, 3H), 2.23 (s, 3H), 2.16 (s, 3H). ^{13}**C NMR** (100.6 MHz) δ 168.10, 137.88, 137.11, 135.61, 133.92, 133.16, 131.14, 128.90, 128.44, 128.07, 127.08, 125.21, 122.14, 24.75, 21.41, 19.91. **HRMS (ESI)** calcd. for $C_{18}H_{19}NONa$ $[M+Na]^+$: 288.1355; found: 288.1359.

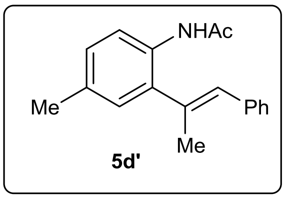

(*E*)-*N*-(4-methyl-2-(1-phenylprop-1-en-2-yl)phenyl)acetamide (5d')

Compound **5d'** was prepared from 4-methyl acetanilide and 1-phenyl-1-propyne by Method C. White solid. ^{1}**H NMR** (200 MHz, Chloroform-d) δ 8.02 (d, *J* = 8.1 Hz, 1H), 7.41-7.25 (m, 6H), 7.09 (d, *J* = 8.0 Hz, 1H), 7.04 (s, 1H), 7.49 (s, 1H), 2.33 (s, 3H), 2.21 (s, 3H), 2.12 (s, 3H). ^{13}**C NMR** (50.3 MHz) δ 168.21, 131.11, 136.30, 135.78, 133.97, 131.51, 131.01, 128.95, 128.47, 127.12, 122.03, 24.64, 20.93, 19.83.

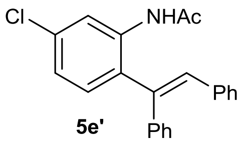

5e'

(*E*)-*N*-(5-chloro-2-(1,2-diphenylvinyl)phenyl)acetamide (5e')

Compound **5e'** was prepared from 3-chloro acetanilide and diphenylacetylene by Method C. White solid. ^{1}**H NMR** (200 MHz, Chloroform-d) δ 8.18 (s, 1H), 7.33-7.25 (m, 5H), 7.20 (d, *J* = 8.0 Hz, 2H), 7.19-7.15 (m, 5H), 7.10 (s, 1H), 6.76 (s, 1H), 1.71 (s, 3H). ^{13}**C NMR** (50.3 MHz) δ 168.81, 138.62, 138.45, 136.20, 134.31, 132.53, 132.34, 131.57, 129.46, 129.35, 129.12, 128.31, 128.16, 127.51, 124.23, 121.89, 14.06.

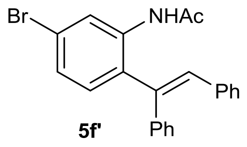

5f'

(*E*)-*N*-(5-bromo-2-(1,2-diphenylvinyl)phenyl)acetamide (5f')

Compound **5f'** was prepared from 3-bromo acetanilide and diphenylacetylene by Method C. Pale white solid. ^{1}**H NMR** (500 MHz, Chloroform-d) δ 8.58 (s, 1H), 7.37 (t, *J* = 10.0 Hz, 2H), 7.34 (d, *J* = 10.2 Hz, 2H), 7.29 (s, 1H), 7.25-7.22 (m, 5H), 7.18 (s, 1H), 7.10-7.08 (m, 2H), 7.04 (d, *J* = 10 Hz, 1H), 1.80 (s, 3H). ^{13}**C NMR** (50.3 MHz) δ 168.05, 141.22, 136.62, 135.85, 132.02, 130.84, 129.84, 129.39, 129.15, 128.80, 128.59, 128.39, 128.18, 127.56, 126.80, 124.06, 121.28, 117.77, 113.57, 24.35.

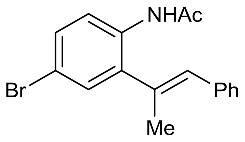

5g'

(*E*)-*N*-(4-bromo-2-(1-phenylprop-1-en-2-yl)phenyl)acetamide (5g')[3]

Compound **5g'** was prepared from 4-bromo acetanilide and 1-Phenyl-1-propyne by Method C. White solid. **^{1}H NMR** (500 MHz, Chloroform-d) δ 8.12 (s, 1H), 7.52 (s, 1H), 7.44 (t, *J* = 10.2 Hz, 2H), 7.40 (d, *J* = 10 Hz, 4H), 7.33 (t, *J* = 6.0 Hz, 1H), 6.53 (s, 1H), 2.23 (s, 3H), 2.15 (s, 3H). **^{13}C NMR** (125.8 MHz) δ 168.26, 137.95, 136.56, 134.19, 133.34, 132.07, 131.13, 130.73, 128.95, 128.55, 127.47, 123.37, 117.01, 24.67, 19.61.

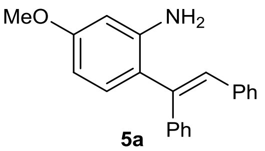

5a

(*E*)-2-(1,2-diphenylvinyl)-5-methoxyaniline (5a)

Gray solid. **^{1}H NMR** (200 MHz, Chloroform-d) δ 7.38 (d, *J* = 8.0 Hz, 2H), 7.29-7.26 (m, 3H), 7.17-7.08 (m, 5H), 7.05 (s, 1H), 6.87 (d, *J* = 8.0 Hz, 1H), 6.32 (dd, *J* = 8.1 Hz, 4 Hz, 1H), 6.26 (s, 1H), 3.75 (s, 3H), 3.56 (s, 2H). **^{13}C NMR** (50.3 MHz) δ 160.47, 145.39, 138.73, 137.22, 132.05, 129.52, 129.39, 129.08, 128.59, 128.34, 127.82, 127.31, 126.91, 126.19, 118.34, 101.48, 101.19, 55.17. **HRMS(ESI)** calcd. for $C_{21}H_{20}NO$ $[M+H]^+$: 302.1536; found: 302.1539.

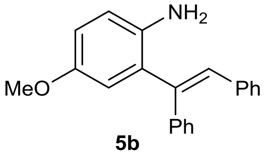

5b

(*E*)-2-(1,2-diphenylvinyl)-4-methoxyaniline (5b)

Gray solid. **^{1}H NMR** (200 MHz, Chloroform-d) δ 7.26-7.20 (m, 5H), 7.17-7.09 (m, 5H), 6.77 (s, 1H), 6.71 (dd, *J* = 8.4 Hz and 3.7 Hz, 2H), 6.57 (dd, *J* = 9.1 Hz and 3.5 Hz, 1H), 3.72 (s, 3H), 3.28 (s, 2H). **^{13}C NMR** (50.3 MHz) δ 152.33, 140.64, 137.87, 136.96, 130.82, 130.65, 129.61, 129.38, 128.89, 128.55, 128.45, 128.16, 127.94, 127.60, 126.83, 126.62, 117.15, 116.27, 114.52, 55.64. **HRMS (ESI)** calcd. for $C_{21}H_{20}NO$ [M+H]$^+$: 302.1467; found: 302.1468.

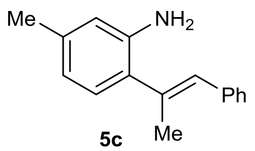

5c

(*E*)-5-methyl-2-(1-phenylprop-1-en-2-yl)aniline (5c)

Brown coloured liquid. **^{1}H NMR** (200 MHz, Chloroform-d) δ 7.38-7.34 (m, 4H), 7.26 (dt, *J* = 8.4 Hz and 3.8 Hz, 1H), 7.0 (d, *J* = 8.4 Hz and 4.0 Hz, 1H), 6.60 (d, *J* = 8.2 Hz and 4.0 Hz, 1H), 6.57-6.54 (m, 2H), 3.76 (s, 2H), 2.28 (s, 3H), 2.22 (s, 3H). **^{13}C NMR** (100.6 MHz) δ 142.73, 137.78, 137.72, 136.52, 129.72, 129.89, 128.79, 128.54, 126.18, 126.50, 119.21, 116.28, 21.12, 19.25. **HRMS (ESI)** calcd. for $C_{18}H_{18}N$ [M+H]$^+$: 224.1430; found: 224.1434.

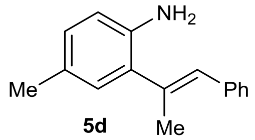

5d

(*E*)-4-methyl-2-(1-phenylprop-1-en-2-yl)aniline (5d)

Brown coloured liquid. **^{1}H NMR** (200 MHz, Chloroform-d) δ 7.39-7.36 (m, 4H), 7.26 (dd, *J* = 10.1 Hz and 3.8 Hz, 1H), 6.91 (d, *J* = 8.0 Hz, 2H), 6.65 (d, *J* = 8.1 Hz, 1H),

6.54 (s, 1H), 3.66 (s, 2H), 2.26 (s, 3H), 2.23 (s, 3H). **^{13}C NMR** (125.8 MHz) δ 140.30, 137.73, 136.73, 131.62, 129.77, 129.14, 128.92, 128.44, 128.21, 127.59126.57, 115.81, 20.43, 19.18. **HRMS (ESI)** calcd. for $C_{16}H_{17}N$ $[M]^+$: 223.1362; found: 223.1361.

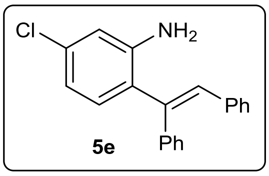

(*E*)-5-chloro-2-(1,2-diphenylvinyl)aniline (5e)

White solid. **^{1}H NMR** (200 MHz, Chloroform-d) δ 7.27-7.21 (m, 5H), 7.17-7.13 (m, 3H), 7.13 (d. *J* = 8.0 Hz, 2H), 7.07-7.04 (m, 1H), 6.75 (s, 1H), 6.72 (dd, *J* = 8.0 Hz, 4.1 Hz, 1H), 6.63 (s, 1H), 3.68 (s, 2H). **^{13}C NMR** (50.3 MHz) δ 145.34, 139.61, 139.13, 136.81, 132.10, 130.35, 129.59, 129.45, 128.70, 128.02, 127.81, 127.00, 118.13, 115.40. **HRMS (ESI)** calcd. for $C_{20}H_{16}ClN$ $[M]^+$: 305.0972; found: 305.0971.

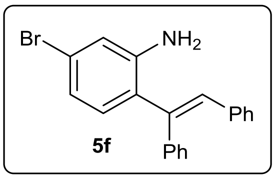

(*E*)-5-bromo-2-(1,2-diphenylvinyl)aniline (5f)

White solid. **^{1}H NMR** (500 MHz, Chloroform-d) δ 7.41 (d, *J* = 10 Hz, 2H), 7.36 (t, *J* = 10.1 Hz, 3H), 7.25-7.20 (m, 3H), 7.16 (d, *J* = 10.0 Hz, 2H), 6.91 (d, *J* = 10.0 Hz, 2H), 6.89 (s, 1H), 3.69 (s, 2H). **^{13}C NMR** (125.8 MHz) δ 145.54, 141.37, 137.72, 136.68, 132.47, 129.95, 129.65, 128.98, 128.61, 128.35, 127.99, 127.54, 126.70, 124.22, 121.77, 118.28. **HRMS (ESI)** calcd. for $C_{20}H_{16}BrN$ $[M]^+$: 349.0466; found: 349.0469.

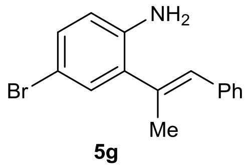

5g

(*E*)-4-bromo-2-(1-phenylprop-1-en-2-yl)aniline (5g)[3]

Semi-solid. **^{1}H NMR** (400 MHz, Chloroform-d) δ 7.44-7.39 (m, 4H), 7.30 (t, *J* = 8.2 Hz, 1H), 7.25 (s, 1H), 7.21 (dd, *J* = 8.2 Hz and 3.8 Hz, 1H), 6.64 (d, *J* = 8.0 Hz, 1H), 6.58 (s, 1H), 3.81 (s, 2H), 2.23 (s, 3H). **^{13}C NMR** (100.6 MHz) δ 142.09, 137.27, 136.37, 133.35, 131.25, 130.72, 130.58, 128.97, 128.34, 126.94, 117.13, 110.15, 18.98.

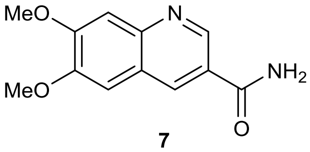

7

6,7-dimethoxyquinoline-3-carboxamide (7)

Yellow solid. Yield: 86%.**^{1}H NMR** (400 MHz, DMSO-d$_6$) δ 8.19 (s, 1H), 7.75 (s, 1H), 7.29 (s, 1H), 6.63 (s, 1H), 6.52 (s, 1H), 6.50 (s, 1H), 3.06 (s, 3H), 3.03 (s, 3H). **^{13}C NMR** (100.6 MHz, DMSO-d$_6$) δ 166.94, 153.48, 149.95, 146.61, 145.99, 133.94, 125.11, 122.17, 107.43, 106.35, 55.88, 55.80. **HRMS (ESI)** calcd. for $C_{12}H_{13}N_2O_3$ $[M+H]^+$: 233.0918, found: 233.0937.

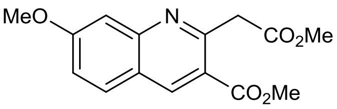

9

Methyl 7-methoxy-2-(2-methoxy-2-oxoethyl)quinoline-3-carboxylate (9)

Yellow solid. Yield: 31%. **^{1}H NMR** (500 MHz, Chloroform-d) δ 8.79 (s, 1H), 7.79 (d, *J* = 10.0 Hz, 1H), 7.41 (s, 1H), 7.24 (dd, *J* = 10.0 Hz and 5.2 Hz, 1H), 4.43 (s, 2H), 3.96 (s, 3H), 3.95 (s, 3H), 3.74 (s, 3H). **^{13}C NMR** (125.8 MHz, Chloroform-d) δ 171.38, 166.46, 162.88, 154.94, 150.73, 140.11, 129.67, 121.48, 120.72, 106.90, 55.68, 52.28, 52.00, 44.51. **HRMS (ESI)** calcd. for $C_{15}H_{16}NO_5$ $[M+H]^+$: 290.1018, found: 290.1023.

Supplementary References

1. Williams, D. B. G. & Lawton, M. Drying of organic solvents: quantitative evaluation of the efficiency of several desiccants. *J. Org. Chem.* **75**, 8351-8354 (2010).

2. Armarego, W. L. F. & Perrin, D. D. *Purification of Laboratory Chemicals* (Pergamon Press, Oxford, 1988) ed 3.

3. Manikandan, R. & Jeganmohan, M. Ruthenium-catalyzed hydroarylation of anilides with alkynes: An efficient route to *ortho*-alkenylated anilines. *Org. Lett.* **16**, 912-915 (2014).

4. Burke, Jr. T. R., Lim, B., Marquez, V. E., Li, Z.-H., Bolen, J. B., Irena Stefanova, I. & Horaks, I. D. Bicyclic compounds as ring-constrained inhibitors of protein-tyrosine kinase p56lck. *J. Med. Chem.* **36**, 425-532 (1993).

5. Newsome, D.S. The water-gas shift reaction. *Catalysis reviews - Science and Engineering* **21**, 275-318 (1980).

6. Becerra-Figueroa, L., Ojeda-Porras, A. & Gamba-Sánchez, D. Transamidation of carboxamides catalyzed by Fe(III) and water. *J. Org. Chem.* **79**, 4544-4552 (2014).

7. O'Del, D. K. & Nicholas, K. M. Synthesis of 3-substituted quinolines via transition-metal-catalyzed reductive cyclization of *o*-nitro Baylis−Hillman acetates. *J. Org. Chem.* **68**, 6427-6430 (2003).

8. Batchu, H., Bhattacharyya, S. & Batra, S. Iodine-mediated intramolecular electrophilic aromatic cyclization in allylamines: A general route to synthesis of quinolines, pyrazolo[4,3-b]pyridines, and thieno[3,2-b]pyridines. *Org. Lett.* **14**, 6330-6333 (2012).

9. Niu, Q., Mao, H., Yuan, G., Gao, J., Liu, H., Tu, Y., Xiaoxia Wang, X. & Lv, X. Copper-catalyzed domino S_N2'/coupling reaction: A versatile and facile synthesis of cyclic compounds from Baylis–Hillman acetates. *Adv. Syn. Catal*. **355**, 1185-1192 (2013).
